# Determinants of self-reported hypertension among women in South Africa: evidence from the population-based survey

**DOI:** 10.1186/s40885-022-00222-5

**Published:** 2022-11-15

**Authors:** Peter Austin Morton Ntenda, Walaa Mamdouh Reyad El-Meidany, Fentanesh Nibret Tiruneh, Mfundi President Sebenele Motsa, Joyce Nyirongo, Gowokani Chijere Chirwa, Arnold Kapachika, Owen Nkoka

**Affiliations:** 1 Malaria Alert Centre, Kamuzu University of Health Sciences, Private Bag 360, Chichiri, Blantyre 3, Malawi; 2 Department of Nutrition, High Institute of Public Health, Alexandria University, Alexandria, Egypt; 3 Department of Applied Human Nutrition, Faculty of Chemical and Food Engineering, Bahir Dar Institute of Technology, Bahir Dar University, Bahir Dar, Ethiopia; 4 Department of Global Health and Health Security, College of Public Health, Taipei Medical University, Taipei City, Taiwan; 5 Department of Economics, Chancellor College, University of Malawi, Zomba, Malawi; 6 Ministry of Health, Machinga District Hospital, Liwonde, Malawi; 7 Institute of Health and Wellbeing, University of Glasgow, Glasgow, UK

**Keywords:** Hypertension, Diabetes mellitus, Hypercholesterolemia, Self report, Overweight, Obesity, Life style, South Africa

## Abstract

**Background:**

Hypertension (HTN), characterized by an elevation of blood pressure, is a serious public health chronic condition that significantly raises the risks of heart, brain, kidney, and other diseases. In South Africa, the prevalence of HTN (measured objectively) was reported at 46.0% in females, nonetheless little is known regarding the prevalence and risks factors of self-reported HTN among the same population. Therefore, the aim of this study was to examine determinants of self-reported HTN among women in South Africa.

**Methods:**

The study used data obtained from the 2016 South African Demographic and Health Survey. In total, 6,027 women aged ≥ 20 years were analyzed in this study. Self-reported HTN was defined as a case in which an individual has not been clinically diagnosed with this chronic condition by a medical doctor, nurse, or health worker. Multiple logistic regression models were employed to examine the independent factors of self-reported HTN while considering the complex survey design.

**Results:**

Overall, self-reported HTN was reported in 23.6% (95% confidence interval [CI], 23.1–24.1) of South African women. Being younger (adjusted odds ratio [aOR], 0.04; 95% CI, 0.03–0.06), never married (aOR, 0.69; 95% CI, 0.56–0.85), and not covered by health insurance (aOR, 0.74; 95% CI, 0.58–0.95) reduced the odds of self-reported HTN. On the other hand, being black/African (aOR, 1.73; 95% CI, 1.17–2.54), perception of being overweight (aOR, 1.72; 95% CI, 1.40–2.11), and perception of having poor health status (aOR, 3.53; 95% CI, 2.53–5.21) and the presence of other comorbidities (aOR, 7.92; 95% CI, 3.63–17.29) increased the odds of self-reported HTN.

**Conclusions:**

Self-reported HTN was largely associated with multiple sociodemographic, health, and lifestyle factors and the presence of other chronic conditions. Health promotion and services aiming at reducing the burden of HTN in South Africa should consider the associated factors reported in this study to ensure healthy aging and quality of life among women.

**Supplementary Information:**

The online version contains supplementary material available at 10.1186/s40885-022-00222-5.

## Background

Hypertension (HTN) characterized by persistently high blood pressure is a serious public health chronic condition that significantly increases the risks of cardiovascular diseases (CVDs), renal failure, blindness, and other diseases [1, 2]. Globally, it is estimated that about 1.3 billion adults aged 30 years and above were diagnosed with HTN where two-thirds of those cases are reported in low-income and middle-income countries [3]. Furthermore, even though it is simple to diagnose HTN and reasonably easy to manage with low-cost drugs, approximately 580 million people with HTN (46%) are unaware of their condition because they were never diagnosed [4]. Globally, HTN is a leading cause of premature death and as part of global targets for noncommunicable diseases, the prevalence of HTN is set to be reduced by 33% between 2010 and 2030 [1, 5].

While the causes of HTN remain a mystery, it is certain that numerous factors may contribute to the pathogenesis of this chronic condition; nonetheless, it is thought that HTN is a result of an interaction of several genetic and environmental factors [6–8]. The World Health Organization (WHO) stated that modifiable risk factors such as unhealthy diets, physical inactivity, tobacco smoking, consumption of a huge amount of alcohol, and being overweight/obese were the risk factors of HTN [9, 10]. Furthermore, WHO stated that nonmodifiable risk factors including genetics, age over 65 years, and comorbidities of other chronic diseases such as diabetes or kidney disease [9, 10] were also the contributing risk factors for HTN. In reaction to the WHO report, a preponderance of previous researchers have reported that age of the respondents [11–14], educational level [13, 15, 16], ethnicity [13, 17], occupation [17], not having health insurance [16], being overweight/obese [11–13, 17, 18], higher waist circumference [19], physical activity [17], smoking status [13], presence of other comorbidities [12, 13, 17, 18, 20], geographical region [11, 20], were significant factors positively associated with HTN. However, most of the studies that were conducted in South Africa, had considered localized areas such as towns and municipalities as well as had focused on the objectively measured HTN (systolic blood pressure level of 140 mmHg or above or a diastolic blood pressure level of 90 mmHg or above) [11, 12, 18, 20]. Nonetheless, there is no study that had addressed the self-reported HTN as an outcome measure in South Africa.

Generally, self-reported HTN is habitually employed in research settings to evaluate the magnitude of this condition in the general population [21–23]. Self-reporting of the specific condition is a simple and low-cost technique that focuses on an individual’s own behaviors, beliefs, attitudes, or intentions [24]. Thus, due to its simplicity and low cost in obtaining health information from individuals, self-reporting has become an integral approach for continuous population monitoring to inform policies aimed to reduce the burden of various diseases [25, 26]. Studies have also reported the validity of self-reports against the objective measurement of the disease. For example, an Iranian study reported a prevalence of 16.8% and 15.7% for self-reported HTN, and HTN based on verified medical history and measurements, respectively [27]. It was, therefore, concluded that self-reported HTN is acceptable, and its method can be used for public health initiatives in the absence of countrywide HTN control and detection programs.

In South Africa, the prevalence of uncontrolled HTN has been estimated at 13.5% to 75.5% [18], while statistics for the controlled HTN ranged from 19.0% to 56.0% [28]. The recent data obtained from the South Africa Demographic and Health Survey (SADHS) indicated that the prevalence of HTN (objectively measured) has nearly doubled since 1998, from 25 to 46% among women [29]. The percentage of women aged 15 years and above with self-reported HTN was reported at 23% in 2016 [29]. These statistics are relatively high and call for urgent attention to reduce the burden of this condition in the general population. HTN is a precursor of stroke and ischemic heart disease, which were the second and fourth leading causes of mortality, respectively, in 2012 in South Africa [30]. Therefore, the objective of this study was to examine the determinants of self-reported HTN among women in South Africa. Such empirical evidence might be required by health policymakers to create targeted strategies for the control of HTN.

## Methods

### Ethical statements

This study was approved by the South Africa Medical Research Council and the Institutional Review Board of International Classification of Functioning (ICF). All methods were conducted in accordance with the Declaration of Helsinki principles. The data were de-identified to prevent the respondents’ identity from being revealed. All individuals who agreed to take part in the survey were asked to provide either written or verbal consent for the interview. Particularly, verbal consent was sought from participants who could not write or read. The Centre for Disease Control and Prevention granted a waiver of written consent per 45CFR46 for respondents who were unable to provide written consent but consented verbally.

### Study design and data sources

The study used cross-sectional data from SADHS 2016. The SADHS 2016 was conducted to provide up-to-date estimates of basic demographic and measures of population health such as fertility levels, awareness and use of contraceptives, childhood and maternal mortality, immunization coverage, and prevalence and treatment of acute respiratory infection, fever, diarrhea, nutrition, etc. [29]. Additionally, the survey was also conducted to provide estimates of health and behavior measures for adults aged 15 years and older.

### Sampling design

The sample for the SADHS 2016 was designed to produce estimates of vital measures of the country as a whole: urban and nonurban areas. Administratively, South Africa is divided into nine provinces namely Eastern Cape, Free State, Gauteng, KwaZulu-Natal, Limpopo, Mpumalanga, Northern Cape, North West, and Western Cape, which vary considerable in size. The Statistics South Africa Master Sample Frame was created using Census 2011 data. Enumeration areas (EAs) was used as the sampling frame for the SADHS 2016. In the Master Sample Frame, EAs of manageable size were treated as primary sampling units (PSUs), whereas small neighboring EAs were combined to form new PSUs, and large EAs were split into conceptual PSUs. Using a two-stage stratified sampling design with a probability proportional to size, PSUs were selected at the first stage while the systematic sampling of residential dwelling units (DUs) was performed at the second stage. Thus, a total of 750 PSUs were selected from the 26 sampling strata. From each PSU, a fixed number of 20 residential DUs were selected using a systematic sampling technique. Of the 15,292 households selected, 13,288 households were occupied, and interviews were successfully conducted in 83% of the occupied households.

### Data collection

Data collection was conducted between June 2016 and November 2016 at the request of the National Department of Health. Data were collected using questionnaires administered by conducting face-to-face interviews. All households were eligible for interviews using the Household Questionnaire where basic demographic indicators on the characteristics of each person listed were collected, including age, sex, marital status, education, and relationship to the head of the household were collected. Further, information on characteristics of the household’s DU, such as the source of drinking water, type of sanitation facility, materials used for the floor, walls, and roof of the DU, and ownership of various durable goods were also collected. Secondly, the woman’s questionnaire was used to collect information from all eligible women aged 15 years and older on background characteristics such as age, education, media, exposure, etc. Additionally, the woman’s questionnaires also included a module on adult health where information on the use of tobacco, alcohol, consumption of fat, salt, sugar, fruit, and vegetables, health care-seeking behaviors, and self-reported prevalence of a variety of noncommunicable diseases including HTN were captured.

### Measures

#### Outcome of interest

The present study considered self-reported HTN (high blood pressure) as the dependent variable. Specifically, respondents were asked, “Has a medical doctor, nurse, or health professional told you that you have high blood pressure?” If the response was “yes” then respondents were classified as having self-rated HTN, and if the response was “no,” then the respondents were considered having no self-reported HTN.

#### Independent variables

Independent variables of this study were selected after a review of the relevant literature and their availability in the SADHS 2016 dataset [11, 17, 31]. In total, 10 sociodemographic variables, seven health and lifestyle-related factors, and three other comorbidities of chronic diseases were included.

Sociodemographic variables included the age of the respondents, education level, ethnicity, household wealth, marital status, employment status, exposure to mass media, place of residence, and geographical region. Lifestyle-related factors included self-reported body mass index (BMI) and perception of own health, type of fruits eaten yesterday, type of vegetables eaten yesterday, frequency of eating processed meat, frequency of eating fried foods, and sugar-sweetened drinks in the last 24 h. Other chronic diseases included comorbidities of self-reported HTN, comorbidities of self-reported diabetes, and comorbidities of self-reported hypercholesterolemia. These variables were categorized as follows: age of the respondents in years (20–24, 25–29, 30–34, 35–39, 40–44, 45–49, 50 + years), educational level (no education/primary, secondary, tertiary education), ethnicity (black/African, white, colored and other), household wealth (poorest, poorer, middle, richer, richest), marital status (never married, currently married, divorced/widowed), current employment (not employed or employed), covered by health insurance (yes or no), the amount of mass media exposure (0, 1, 2, 3), place of residence (urban or rural), and geographical region (Western Cape, Eastern Cape, Northern Cape, Free State, KwaZulu-Natal, North West, Gauteng, Mpumalanga, Limpopo). Household wealth was calculated using household items such as such as televisions and bicycles, materials used for housing construction, and types of water access and sanitation facilities and principal component analysis was used to create scores and these were further divided into quintiles.

Health and lifestyle-related factors included perception of own weight—BMI (underweight, normal weight, overweight/obese), perception of own health, also known as self-rated health status (SRHS) was measured using a single question: “In general, how do would you rate your health?” with the following response options: excellent, good, moderate, or poor; type of fruits eaten yesterday (none, one type, two or more types); type of vegetables eaten yesterday (none, one type, two or more types); frequency of eating processed meat (never, every day, at least once a week, occasionally); frequency of eating fried foods (never, every day, at least once a week, occasionally); and sugar-sweetened drinks yesterday (no or yes). Objectively BMI is calculated by dividing the person’s weight in kilograms by their height in meters squared (kg/m^2^) [32]. For adults over 20 years old, BMI falls into one of the following categories: underweight (below 18.5 kg/m^2^), normal weight (18.5–24.9 kg/m^2^), overweight (25.0–29.9 kg/m^2^), and obesity (30 kg/m^2^ and above).

Comorbidities for self-reported HTN included self-reported diabetes and self-reported hypercholesterolemia. Particularly, respondents were asked; “Has a medical doctor, nurse, or health provider told you that you have high blood pressure?” If the response was “yes” then respondents were classified as having self-reported diabetes/blood sugar or high blood cholesterol/fats in the blood (hypercholesterolemia), and if the response was “no”, then the respondents were considered having no self-reported diabetes or hypercholesterolemia.

### Statistical analysis

All data analyses were conducted using SAS ver. 9.4 (SAS Institute Inc., Cary, NC, USA). Characteristics of the study population were presented with numbers and percentages. Group comparisons between respondents that reported to have HTN and those that did not have HTN were made with Rao-Scott chi-square test. To assess the relationship between the selected variables and self-reported HTN, multivariate logistic analyses were constructed using a generalized estimating equation. The generalized estimating equation models accounted for the clustering effects of the hierarchical SADHS data. The results of the multivariate analysis were reported as adjusted odds ratios (aORs) with their *P*-values and 95% confidence intervals (CIs). A *P*-value of less than 0.05 was considered statistically significant.

## Results

### Baseline characteristics

Table 1 displays the baseline characteristics of the study sample. Of the 6,027 respondents analyzed in this study, self-reported HTN occurred in 23.6% of women (95% CI, 23.1–24.1). Regarding sociodemographic factors, the average age of respondents was 40.7 years, with about 23.4% of them aged 50 years or older. Levels of education, in general, were low, with 26.4% having received no formal/primary education, about 64.3% attained secondary school education, and a majority of them (84.5%) were black or Africans. About half (50.3%) reported that they had never been married and approximately three-quarters of respondents (71.3%) were not currently employed. A majority of respondents (86.3%) were not covered by health insurance and about 42.4% had access to all three forms of media. More than half of the respondents (55.2%) resided in urban areas. Regarding health and lifestyle factors, a majority of respondents (76.7%), reported normal BMI and 13.0% reported their self-rated health as excellent. Intake of fruits and vegetables in general, was low, with about 51.0% and 40.0% of respondents failing to consume any type of fruits and vegetables in the last 24 h, respectively. About one-third of the participants consumed fried foods and processed meat at least once a week. Similarly, one-third of the respondents had taken sugar-sweetened drinks in the last 24 h before the data collection.

**Table 1:** Sociodemographic characteristic of study respondent (*n* = 6,027)

Characteristic	No. (%)
Age (yr)	
Mean ± standard deviation	40.7 ± 18.4
20–24	1,397 (23.2)
25–29	705 (11.7)
30–34	616 (10.7)
35–39	515 (8.5)
40–44	459 (7.6)
45–49	445 (7.4)
50 +	1,890 (31.4)
Education	
No education/primary	1,591 (26.4)
Secondary	3,875 (64.3)
Tertiary	561 (9.3)
Ethnicity	
Black/African	5,092 (84.5)
White	258 (4.3)
Colored and other	677 (11.2)
Wealth	
Poorest	1,209 (20.1)
Poorer	1,239 (20.6)
Middle	1,346 (22.3)
Richer	1,267 (21.0)
Richest	966 (16.0)
Marital status	
Never married	3,034 (50.3)
Currently married	2,018 (33.5)
Divorced/widowed	975 (16.2)
Current employment	
Not employed	4,296 (71.3)
Employed	1,731 (28.7)
Covered by health insurance	
No	5203 (86.3)
Yes	824 (13.7)
Amount of mass media exposure^a^	
0	725 (12.0)
1	1,021 (17.0)
2	1,723 (28.6)
3	2,558 (42.4)
Place of residence	
Urban	3,325 (55.2)
Rural	2,702 (44.8)
Geographical region	
Western Cape	470 (7.8)
Eastern Cape	785 (13.0)
Northern Cape	519 (8.6)
Free State	646 (10.7)
Kwazulu-Natal	962 (16.0)
North West	577 (9.6)
Gauteng	553 (9.2)
Mpumalanga	693 (11.5)
Limpopo	822 (13.6)
Perception of own weight	
Underweight	561 (9.3)
Normal weight	4,620 (76.7)
Overweight/obese	846 (14.0)
Perception of own health	
Poor	766 (12.7)
Average	2,019 (33.5)
Good	2,460 (40.8)
Excellent	782 (13.0)
Type of fruits eaten yesterday	
None	3,061 (50.8)
One	1,730 (28.7)
Two or more	1,236 (20.5)
Type of vegetables eaten yesterday	
None	2,382 (39.5)
One	1,985 (32.9)
Two or more	1,660 (27.5)
Frequency of eating processed meat	
Never	672 (11.2)
Every day	713 (11.8)
At least once a week	1,687 (28.0)
Occasionally	2,955 (49.0)
Frequency of eating fried foods	
Never	469 (7.8)
Every day	513 (8.5)
At least once a week	1,951 (32.4)
Occasionally	3,094 (51.3)
Sugar-sweetened drinks yesterday	
No	4,103 (68.1)
Yes	1,924 (31.9)
Self-reported hypertension	
No	4,603 (76.4)
Yes	1,424 (23.6)

^a^Frequency of viewing television, listening to radio, and reading newspaper

### Comparison of the self-reported hypertension rates among the study respondents

Table 2 compares the prevalence of self-reported HTN among women of different characteristics. In terms of sociodemographic characteristics, respondents of different age (χ^2^ = 1362.1, df = 6, *P* < 0.001), ethnicity (χ^2^ = 19.2, df = 2, *P* < 0.001), education (χ^2^ = 315.3, df = 2, *P* < 0.001), household wealth (χ^2^ = 49.5, df = 4, *P* < 0.001), marital status (χ^2^ = 469.9, df = 2, *P* < 0.001), exposure to mass media (χ^2^ = 31.1, df = 3, *P* < 0.0001), type of place of residence (χ^2^ = 11.0, df = 1, *P* = 0.001), and geographical region (χ^2^ = 121.5, df = 1, *P* < 0.001) had statistically different self-reported hypertensive rates. In terms of health, life styles, and other comorbidities, respondents of different perceived BMI (χ^2^ = 79.8, df = 2, *P* < 0.001), perceived health (χ^2^ = 505.2, df = 3, *P* < 0.001), frequency of eating processed meat (χ^2^ = 76.8, df = 3, *P* < 0.001), frequency of eating fried foods (χ^2^ = 51.5, df = 3, *P* < 0.001), sugar-sweetened drinks (χ^2^ = 24.2, df = 1, *P* < 0.001), presence of other comorbidities (χ2 = 132.3, df = 1, *P* < 0.001) had also significantly reported different hypertensive rates.

**Table 2:** Prevalence of self-reported hypertension by selected factors among women in South Africa

Characteristic	Self-reported hypertension		*P*-value
	No (*n* = 4,603)	Yes (*n* = 1,424)	
Age (yr)			< 0.001
20–24	1,359 (29.5)	38 (2.7)	
25–29	651 (14.1)	54 (3.8)	
30–34	555 (12.1)	61 (4.3)	
35–39	441 (9.6)	74 (5.2)	
40–44	361 (7.8)	98 (6.9)	
45–49	325 (7.1)	120 (8.4)	
50 +	911 (19.8)	979 (68.8)	
Education			< 0.001
No education/primary	957 (20.8)	634 (44.5)	
Secondary	3,182 (69.1)	693 (48.7)	
Tertiary	464 (10.1)	97 (6.8)	
Ethnicity			< 0.001
Black/African	3,941 (85.6)	1,151 (80.8)	
White	180 (3.9)	78 (5.5)	
Colored and other	482 (10.5)	195 (13.7)	
Wealth			< 0.001
Poorest	993 (21.6)	216 (15.2)	
Poorer	985 (21.4)	254 (17.8)	
Middle	1,006 (21.9)	340 (23.9)	
Richer	918 (19.9)	349 (24.5)	
Richest	701 (15.2)	265 (18.6)	
Marital status			< 0.001
Never married	2,627 (57.1)	407 (28.6)	
Currently married	1,450 (31.5)	568 (39.9)	
Divorced/widowed	526 (11.4)	449 (31.5)	
Current employment			0.071
Not employed	3,254 (70.7)	1,042 (73.2)	
Employed	1,349 (29.3)	382 (26.8)	
Covered by health insurance			0.006
No	4,005 (87.0)	1,198 (84.1)	
Yes	598 (13.0)	226 (15.9)	
Amount of mass media exposure^a^			< 0.001
0	548 (11.9)	177 (12.4)	
1	763 (16.6)	258 (18.1)	
2	1,252 (27.2)	471 (33.1)	
3	2,040 (44.3)	518 (36.4)	
Place of residence			0.001
Urban	2,485 (54.0)	840 (59.0)	
Rural	2,118 (46.0)	584 (41.0)	
Geographical region			< 0.001
Western Cape	324 (7.0)	146 (10.3)	
Eastern Cape	560 (12.2)	225 (15.8)	
Northern Cape	370 (8.0)	149 (10.5)	
Free State	451 (9.8)	195 (13.7)	
Kwazulu-Natal	803 (17.5)	159 (11.2)	
North West	408 (8.9)	169 (11.9)	
Gauteng	443 (9.6)	110 (7.7)	
Mpumalanga	552 (12.0)	141 (9.9)	
Limpopo	692 (15.0)	130 (9.1)	
Perception of own weight			< 0.001
Underweight	380 (8.2)	181 (12.7)	
Normal weight	3,653 (79.4)	967 (67.9)	
Overweight/obese	570 (12.4)	276 (19.4)	
Perception of own health			< 0.001
Poor	414 (9.0)	352 (24.7)	
Average	1,374 (29.9)	645 (45.3)	
Good	2,090 (45.4)	370 (26.0)	
Excellent	725 (15.8)	57 (4.0)	
Type of fruits eaten yesterday			0.120
None	2,306 (50.1)	755 (53.0)	
One	1,348 (29.3)	382 (26.8)	
Two or more	949 (20.6)	287 (20.2)	
Type of vegetables eaten yesterday			0.288
None	1,844 (40.1)	538 (37.8)	
One	1,507 (32.7)	478 (33.5)	
Two or more	1,252 (27.2)	408 (28.7)	
Frequency of eating processed meat			< 0.001
Never	444 (9.7)	228 (16.0)	
Everyday	609 (13.2)	104 (7.3)	
At least once a week	1,321 (28.7)	366 (25.7)	
Occasionally	2,229 (48.4)	726 (51.0)	
Frequency of eating fried foods			< 0.001
Never	325 (7.1)	144 (10.1)	
Every day	442 (9.6)	71 (5.0)	
At least once a week	1,532 (33.3)	419 (29.4)	
Occasionally	2,304 (50.0)	790 (55.5)	
Sugar-sweetened drinks yesterday			< 0.001
No	3,058 (66.4)	1,045 (73.4)	
Yes	1,545 (33.6)	379 (26.6)	
Presence of other comorbidities^b^			< 0.001
No	4,593 (99.8)	1,370 (96.2)	
Yes	10 (0.2)	54 (3.8)	

^a^Frequency of viewing television, listening to radio, and reading newspaper; ^b^Other comorbidities for self-reported hypertension (self-reported diabetes or self-reported hypercholesterolemia)

### Factors associated with self-reported hypertension

Table 3 shows factors associated with self-reported HTN among women in South Africa. In this study, the significant predictors of self-reported HTN were the category of the respondent’s age, ethnicity, levels of education, marital status, insurance coverage, geographical region, self-rated body weight, SRHS, and presence of other comorbidities. Compared to respondents of age group 50 years and above, those aged 20 to 24 years (aOR, 0.04; 95% CI, 0.03–0.06) had reduced odds of self-reported HTN. Respondents who had never been married (aOR, 0.69; 95% CI, 0.56–0.85) had also lower odds of self-reported HTN compared to respondents who were formerly married. Furthermore, respondents who had no insurance coverage (aOR, 0.74; 95% CI, 0.58–0.95) had reduced odds of self-reported HTN compared to their counterparts who had insurance coverage. On the other hand, increased odds of self-reported HTN were observed in respondents who perceived their body weight to be overweight (aOR, 1.72; 95% CI, and 1.40–2.11), compared to respondents who perceived their body weight as normal. Being black/African (aOR, 1.73; 95% CI, 1.17–2.54), perception of having poor health status (aOR, 3.53; 95% CI, 2.53–5.211), and the presence of other comorbidities (aOR, 7.92; 95% CI, 3.63–17.29) increased the odds of self-reported HTN. Other predictors that increased the odds of self-reporting HTN included being resident of Western Cape (aOR, 2.70; 95% CI, 1.80–4.05), Eastern Cape (aOR, 2.31; 95% CI, 1.67–3.18), Northern Cape (aOR, 3.01; 95% CI, 2.06–4.40), Free State (aOR, aOR, 2.16; 95% CI, 1.52–3.07), North West (aOR, 2.75; 95% CI, 1.97–3.85), Gauteng (aOR, 1.75; 95% CI, 1.20–2.25), and Mpumalanga (aOR, 1.70; 95% CI, 1.21–2.38) compared to being a Limpopo dweller.

**Table 3:** Predictors of self-reported hypertension among women in South Africa

Characteristic	Self-reported hypertension	
	aOR (95% CI)	*P*-value
Age (yr)		
20–24	0.04 (0.03–0.06)	< 0.001
25–29	0.10 (0.07–0.14)	< 0.001
30–34	0.12 (0.09–0.16)	< 0.001
35–39	0.17 (0.13–0.23)	< 0.001
40–44	0.27 (0.21–0.36)	< 0.001
45–49	0.37 (0.28–0.48)	< 0.001
50 +	1.00	
Education		
No education/Primary	1.38 (0.98–1.95)	0.069
Secondary	1.53 (1.13–2.07)	0.006
Tertiary	1.00	
Ethnicity		
Black/African	1.73 (1.17–2.54)	0.006
Colored and other	1.28 (0.83–1.96)	0.260
White	1.00	
Wealth		
Poorest	0.75 (0.52–1.07)	0.113
Poorer	1.00 (0.71–1.39)	0.973
Middle	1.20 (0.89–1.60)	0.227
Richer	1.15 (0.89–1.49)	0.298
Richest	1.00	
Marital status		
Never married	0.74 (0.62–0.88)	0.001
Divorced/separated	1.07 (0.88–1.30)	0.486
Currently married	1.00	
Current employment		
Not employed	1.12 (0.94–1.33)	0.210
Employed	1.00	
Covered by health insurance		
No	0.74 (0.58–0.95)	0.018
Yes	1.00	
Amount of mass media exposure^a^		
0	0.94 (0.70–1.25)	0.663
1	1.08 (0.85–1.37)	0.529
2	1.11 (0.92–1.34)	0.279
3	1.00	
Place of residence		
Urban	1.13 (0.91–1.40)	0.261
Rural	1.00	
Geographical region		
Western Cape	2.70 (1.80–4.05)	< 0.001
Eastern Cape	2.31 (1.67–3.18)	< 0.001
Northern Cape	3.01 (2.06–4.40)	< 0.001
Free State	2.16 (1.52–3.07)	< 0.001
Kwazulu-Natal	1.33 (0.96–1.85)	0.090
North West	2.75 (1.97–3.85)	< 0.001
Gauteng	1.75 (1.20–2.55)	0.004
Mpumalanga	1.70 (1.21–2.38)	0.002
Limpopo	1.00	
Perception of own weight		
Underweight	0.91 (0.71–1.16)	0.443
Overweight/obese	1.72 (1.40–2.11)	< 0.001
Normal weight	1.00	
Perception of own health		
Poor	3.63 (2.53–5.21)	< 0.001
Average	2.74 (1.99–3.79)	< 0.001
Good	1.53 (1.10–2.11)	0.010
Excellent	1.00	
Type of fruits eaten yesterday		
None	1.04 (0.85–1.27)	0.740
One	0.97 (0.78–1.21)	0.787
Two or more	1.00	
Type of vegetables eaten yesterday		
None	1.08 (0.88–1.31)	0.431
One	1.20 (0.99–1.46)	0.064
Two or more	1.00	
Frequency of eating processed meat		
Never	1.14 (0.90–1.44)	0.392
At least once a week	0.98 (0.74–1.30)	0.632
Occasionally	0.91 (0.76–1.10)	0.888
Every day	1.00	
Frequency of eating fried foods		
Never	0.92 (0.70–1.21)	0.424
At least once a week	0.78 (0.57–1.08)	0.291
Occasionally	0.93 (0.78–1.10)	0.134
Every day	1.00	
Sugar-sweetened drinks yesterday		
No	0.93 (0.79–1.10)	0.386
Yes	1.00	
Presence of other comorbidities^b^		
Yes	7.92 (3.63–17.29)	< 0.001
No	1.00	

^a^Frequency of viewing television, listening to radio, and reading newspaper; ^b^Other comorbidities for self-reported hypertension (self-reported diabetes or self-reported hypercholesterolemia)

## Discussion

The present study revealed a considerable high prevalence (23.6%; 95% CI, 23.1–24.1) of self-reported HTN among women in South Africa. Thus, efforts to reduce the burden of HTN can improve the quality of life among women. Even though the present study was exploratory in nature, it has revealed some important factors that may be worthy of the future investigation. The study found that the age of the respondents, ethnicity, education status, marital status, insurance coverage, geographical region, self-rated body weight, SRHS, and presence of other comorbidities were the significant predictors of self-reported HTN.

In line with previous studies [11, 12, 33–35], the current study revealed that age was associated with the likelihood of the respondents to have a self-reported HTN. The results also revealed a clear dose–response relationship such that the likelihood of respondents having self-reported HTN increased with an increase in age. HTN has been linked with numerous health hazards, and the incidence of this condition is reported to be common among older people [36]. Generally, previous researchers have argued that HTN is a result of arterial stiffness, thickness, loss of elasticity, and stenosis of the aorta and arterial wall, a state that is attributed to aging [37]. Prior researchers have also shown that aging is also associated with inflammation, oxidative stress, and endothelial dysfunction the conditions that jointly influence the health risks of late-life HTN [38–40]. Furthermore, empirical evidence has demonstrated that older people tend to have more functional disorders as physical activity declines [41, 42] a phenomenon that increases the likelihood of reporting HTN. Others have also reported that the risk of HTN is high in older people since they pay less attention to their health and are more likely to have financial constraints that hinder them from accessing health care [43].

In line with prior research [44, 45], lower education levels (secondary education and lower) were associated with the likelihood of having self-reported HTN. Education has been reported to play a vital role in guarding against diseases that are influenced by lifestyle such as diabetes [44]. Thus, higher levels of education may enable attainment of social, psychological, and economic positive skills and assets, and may offer insulation from various adverse stimuli [46]. Furthermore, a number of studies have suggested that educational interventions can improve HTN knowledge, health behaviors, and blood pressure control [47]. Additionally, there is clear evidence on the impact of educational level on lifestyle, nutrition, and physical activity [48], of which the three aforementioned characteristics have an impact on HTN [49].

The likelihood of having self-reported HTN was high in blacks or Africans. Our results are in line with finding from Brazil [13, 50], and the United States [51, 52] where high prevalence rates of self-reported HTN was reported in black women than white women. The probable factors related to the higher prevalence of self-reported HTN in the black population are thought to be genetic predisposition [53], poor economic conditions [54], less access to health services, and stress due to racial discrimination [55, 56]. In line with other studies in South Africa, a survey in Cape Town confirmed very high levels of HTN among black South Africans than whites [57]. Similarly, multiple surveys conducted in urban areas of South Africa had documented higher blood pressures in blacks compared to whites [58, 59].

Having a stable health insurance coverage is often related to improved chronic disease outcomes (such as HTN, diabetes, cancer, and CVD) by ensuring reliable access to outpatient services including detection of disease, disease monitoring, and medications [60–62]. Generally, patients that have health insurance coverage are reported to have better access to health care [63, 64] and a number of previous studies have reported that patients with health insurance coverage had an increased odds of achieving blood pressure control compared to their counterparts without health insurance [60, 65]. Surprisingly, in this study, respondents who had no health insurance coverage exhibited a lower likelihood of having self-reported HTN. The reasons may be that uninsured individuals might be less likely to receive preventive services for chronic conditions such as diabetes, cancer, and CVD, a phenomenon that might make them unaware of their present health conditions. On the other hand, previously, it was reported that residents of Free State, Mpumalanga, and KwaZulu-Natal [11, 20] had higher odds of HTN in South Africa. However, in this study, it was found that additional provinces namely Western Cape, Eastern Cape, Northern Cape, North West, Gauteng, and Mpumalanga exhibited increased odds of having self-reported HTN. Thus, forthcoming studies should consider the probable causes for higher odds of self-reported HTN in those additional provinces.

Consistent with previous studies [66, 67], the current study also revealed that marital status was associated with HTN. In particular, those that were never married had a reduced likelihood of having self-reported HTN. In previous cross-section studies, it was reported that those that were not married (divorced, separated, or widowed) and never married individuals were found to have a higher prevalence of HTN compared to their married counterparts [68]. Furthermore, longitudinal studies have reported somewhat inconsistent results where no associations were found between marital status and change in marital status and HTN [69]. The mechanisms by which marital status influences HTN are poorly understood. However, previous researchers have suggested some reasons for the influences of marital status that include psychopathological factors, health behaviors including physical activities and diet adherence, neuroendocrine pathways, and biological mediators and immune pathways [69]. It is believed that married people have less stress, better sleep, better moods, and have a more healthy diet compared to the never married counterparts [66]. Thus, the results of the current study need further investigation.

In this study, overweight/obesity emerged as a significant predictor associated with self-reported HTN among women. These results are in line with the findings from some of the previous work in different parts of the world i.e., that reported on the HTN which was measured objectively [70–73]. Generally, the specific mechanisms through which overweight/obesity result in HTN are complex and poorly understood. However, previous researchers have documented that neuroendocrine mechanisms and, lately, factors derived from adipose are believed to play a major role [74, 75]. On the other hand, overweight/obesity might induce HTN and other CVD by activating the renin–angiotensin–aldosterone system, increasing sympathetic activity, promoting insulin resistance and leptin resistance, increased procoagulatory activity, and endothelial dysfunction [75]. Additionally, other mechanisms involve an increased renal sodium reabsorption that causes a shift of the pressure natriuresis; thus, causing extracellular fluid volume expansion [76]. Furthermore, overweight/obesity may increase the risk of HTN through subclinical inflammation.

As with previous studies [77–79], the current study found an association between poor SRHS and the likelihood of self-reported HTN. Previous researchers have used this simple and relatively subjective SRHS to predict chronic disease morbidity including CVD [80–82], cardiovascular mortality [83–85], and all‐cause mortality [86–89]. In line with prior research [80, 81], respondents who reported having poor SRHS had high chances of having self-reported HTN in this study. The mechanisms through which SRHS induces HTN are not clearly stated. However, SRHS in previous studies has been reported to be closely associated with social integration and adaptability to psychosocial stress [90, 91]. The probable clarifications for the observed relationship between SRHS and the occurrence of severe HTN may comprise the stated association between poor SRHS and associated negative psychosocial variable gathering to the metabolic syndrome [92], increased markers of inflammation and insulin resistance that result in increasing blood sugar [93, 94], sleep apnea and disordered sleep [95], and elevated levels of cortisol and low levels and adrenal androgen.

In this study, the presence of other chronic comorbidities of diabetes and hypocholesteremia had a high likelihood of having self-reported HTN. Our results are in agreement with similar findings from previous studies [96–99], where dyslipidemia, obesity, and impaired fasting glucose were reported to be the most common comorbidities of hypertensive patients. However, in this study we only considered self-reported diabetes and self-reported hypocholesteremia as comorbidities of self-reported HTN due to the availability of data. Another study reported that seven of the eight chronic conditions were more prevalent in hypertensive patients than in adults without HTN [96]. Generally, HTN, diabetes, and hypercholesterolemia share common pathways and are interrelated [100]. It is established that hypercholesterolemia facilitates HTN by decreasing coronary blood flow reserve and capillary density; and inducing apoptosis of capillary endothelial cells and eventually resulting in impaired left ventricular function [101]. Furthermore, it is believed that hypercholesterolemia may alter membrane lipid bilayer [102], and may alter the regulation of intracellular calcium ions; thus, excess calcium uptake could directly contribute to atherogenesis [103] and eventually, that may lead to the development of atherosclerosis.

Similarly, there is a significant overlap between diabetes and HTN in the causes and disease pathophysiology [104]. It is established that renal sodium control differs in diabetes since there is an upregulation of sodium transporters in the kidneys [105]. Thus, the renin–angiotensin–aldosterone system may be upregulated in diabetes, resulting in increased blood pressure through a direct effect mediated by angiotensin II [106], as well as indirectly through upregulation of sympathetic activity [107]. Another essential connection between HTN and diabetes is the occurrence as well as the progression of diabetic kidney disease [108], the pathophysiology of which is mediated through several pathways including endothelial dysfunction and advanced glycation end-products [109]. Furthermore, obesity and metabolic syndrome, through their effects on various hormones and inflammation pathways, may also influence the occurrence of HTN and diabetes [107].

This study has several strengths. Firstly, the use of a nationally representative sample makes the results of the current study generalizable to the specified population. Secondly, the analysis controlled for a wide range of characteristics, thus strengthening the observed associations. However, we are aware of the potential limitations of the current study. Firstly, this study utilized a cross-sectional design. Therefore, the temporal relationship between self-reported HTN and its associated factors could not be established in this study. Secondly, the outcome measure and other independent variables were self-reported diagnoses; thus, our results are prone to recall bias. Thirdly, it should be noted that chronic conditions such as HTN are frequently under-reported or incorrectly reported by patients, and therefore, the results should be interpreted with caution.

## Conclusions

Self-reported HTN was largely associated with multiple sociodemographic, health, and lifestyle factors as well the presence of other chronic conditions. Health education, promotion, and services aiming at reducing the burden of HTN in South Africa should consider self-reported HTN and the associated factors reported in this study to ensure healthy aging and quality of life among women.

## Supplementary Information

**Additional file 1.** Questionnaire.

## Data Availability

The datasets generated and/or analyzed during the present study are available in the DHS Program repository: https://dhsprogram.com/data/dataset/South-Africa_Standard-DHS_2016.cfm?flag=1.

## Abbreviations

aOR: Adjusted odds ratio
BMI: Body mass index
CI: Confidence interval
CVD: Cardiovascular disease
DU: Dwelling unit
EA: Enumeration area
HTN: Hypertension
PSU: Primary sampling unit
SADHS: South Africa Demographic and Health Survey
SRHS: Self-rated health status
WHO: World Health Organization
