# Supplementary material for: Determinants of self-reported hypertension among women in South Africa: evidence from the population-based survey
Source: Clin Hypertens. 2022 Nov 15;28:39. doi: 10.1186/s40885-022-00222-5 (PMC9664601; doi:10.1186/s40885-022-00222-5)
Supplement: Supplementary file 1 — Additional file 1. Questionnaire. [file 40885_2022_222_MOESM1_ESM.pdf]

QUESTIONNAIRES *Appendix* E

2016 SOUTH AFRICA DEMOGRAPHIC AND HEALTH SURVEY
HOUSEHOLD QUESTIONNAIRE

IDENTIFICATION

PLACE NAME ______

NAME OF HOUSEHOLD HEAD ______

CLUSTER NUMBER . . . . . . [ ][ ][ ][ ]

PSU NUMBER . . . . . . [ ][ ][ ][ ][ ][ ][ ][ ]

DWELLING UNIT NUMBER . . . . . . [ ][ ][ ][ ]

HOUSEHOLD NUMBER . . . . . . [ ][ ][ ][ ]

HOUSEHOLD SELECTED FOR MALE SURVEY AND BIOMARKERS? (YES = 1; NO = 2) . . . . . . [ ]

HOUSEHOLD SELECTED FOR SALT SAMPLE COLLECTION? (YES = 1; NO = 2) . . . . . . [ ]

INTERVIEWER VISITS

	1	2	3	FINAL VISIT
DATE	______	______	______	DAY [ ][ ]<br>MONTH [ ][ ]<br>YEAR 2 0 1 [ ]
INTERVIEWER'S NAME	______	______	______	INT. NO. [ ][ ][ ][ ]
RESULT*	______	______	______	RESULT* [ ]
NEXT VISIT: DATE	______	______		TOTAL NUMBER OF VISITS [ ]
TIME	______	______		

*RESULT CODES:

1 COMPLETED
2 NO HOUSEHOLD MEMBER AT HOME OR NO COMPETENT RESPONDENT AT HOME AT TIME OF VISIT
3 ENTIRE HOUSEHOLD ABSENT FOR EXTENDED PERIOD OF TIME
4 POSTPONED
5 REFUSED
6 DWELLING VACANT OR ADDRESS NOT A DWELLING
7 DWELLING DESTROYED
8 DWELLING NOT FOUND
9 OTHER ______ (SPECIFY)

TOTAL PERSONS IN HOUSEHOLD [ ][ ]

TOTAL ELIGIBLE WOMEN [ ][ ]

TOTAL ELIGIBLE MEN [ ][ ]

TOTAL CHILDREN ELIGIBLE FOR CAREGIVER'S QUEST. [ ][ ]

LINE NO. OF RESPONDENT TO HOUSEHOLD QUESTIONNAIRE [ ][ ]

LANGUAGE OF QUESTIONNAIRE** 0 1 | LANGUAGE OF INTERVIEW** [ ][ ] | HOME LANGUAGE OF RESPONDENT** [ ][ ] | TRANSLATOR USED (YES = 1, NO = 2) [ ]

LANGUAGE OF QUESTIONNAIRE** **ENGLISH**

**LANGUAGE CODES:

01 ENGLISH	05 seSOTHO	09 tshiVENDA
02 AFRIKAANS	06 seTSWANA	10 xiTSONGA
03 isiXHOSA	07 sePEDI	11 isiNDEBELE
04 isiZULU	08 siSWATI	12 OTHER

SUPERVISOR

______ NAME [ ][ ][ ][ ] NUMBER

THIS PAGE IS INTENTIONALLY BLANK

INTRODUCTION

Hello. My name is _______________________________________. I am working with Statistics South Africa. We are conducting a survey about health and other topics all over South Africa. The information we collect will help the government to plan health services. Your household was selected for the survey. I would like to ask you some questions about your household. The questions usually take about 20 minutes. All of the answers you give will be confidential and will not be shared with anyone other than members of our survey team. In case you need more information about the survey, you may contact the person listed on this card.

GIVE CARD WITH CONTACT INFORMATION

Do you have any questions?
May I begin the interview now?

SIGNATURE OF INTERVIEWER ____________________________ DATE ________________

RESPONDENT AGREES TO BE INTERVIEWED . . 1 ↓

RESPONDENT DOES NOT AGREE TO BE INTERVIEWED . . 2 → END

100	RECORD THE TIME.	HOURS . . . . [ ][ ]<br>MINUTES . . . . [ ][ ]

HOUSEHOLD SCHEDULE

								IF AGE 15 OR OLDER
LINE NO.	USUAL RESIDENTS AND VISITORS	RELATIONSHIP TO HEAD OF HOUSEHOLD	SEX	RESIDENCE		DATE OF BIRTH	AGE	MARITAL STATUS
1	2	3	4	5	6	6A	7	8
	Please give me the names of the persons who usually live in your household and guests of the household who stayed here last night, starting with the head of the household.<br><br>AFTER LISTING THE NAMES AND RECORDING THE RELATIONSHIP, SEX RESIDENCE AND AGE FOR EACH PERSON, ASK QUESTIONS 2A-2C TO BE SURE THAT THE LISTING IS COMPLETE.<br><br>THEN ASK APPROPRIATE QUESTIONS IN COLUMNS 8-27 FOR EACH PERSON.	What is the relationship of (NAME) to the head of the household?<br><br>SEE CODES BELOW.	Is (NAME) male or female?	Does (NAME) usually live here?	Did (NAME) stay here last night?	What is (NAME)'s date of birth?<br><br>On what day, month, and year was (NAME) born?<br><br>IF DON'T KNOW DAY, RECORD '98'.<br>IF DON'T KNOW MONTH, RECORD '98'.<br>IF DON'T KNOW YEAR, RECORD '9998.'	How old is (NAME)?<br><br>IF 95 OR MORE, RECORD '95'.<br><br>COMPARE AND CORRECT 6A AND/OR 7 IF INCON-SISTENT.	What is (NAME)'s current marital status?<br><br>1 = MARRIED OR LIVING TOGETHER<br>2 = DIVORCED/ SEPARATED<br>3 = WIDOWED<br>4 = NEVER-MARRIED AND NEVER LIVED TOGETHER
			M F	Y N	Y N	DAY MONTH YEAR	IN YEARS	
01			1 2	1 2	1 2			
02			1 2	1 2	1 2			
03			1 2	1 2	1 2			
04			1 2	1 2	1 2			
05			1 2	1 2	1 2			
06			1 2	1 2	1 2			
07			1 2	1 2	1 2			

TICK HERE IF CONTINUATION SHEET USED ☐

7A) Just to make sure that I have a complete listing: are there any other people such as small children or infants that we have not listed?	YES ☐ → ADD TO TABLE	NO ☐
7B) Are there any other people who may not be members of your family, such as domestic workers, lodgers, or friends who usually live here?	YES ☐ → ADD TO TABLE	NO ☐
7C) Are there any guests or temporary visitors staying here, or anyone else who stayed here last night, who have not been listed?	YES ☐ → ADD TO TABLE	NO ☐

CODES FOR Q. 3: RELATIONSHIP TO HEAD OF HOUSEHOLD

01 = HEAD	07 = PARENT-IN-LAW
02 = WIFE/HUSBAND/PARTNER	08 = BROTHER OR SISTER
03 = SON OR DAUGHTER	09 = OTHER RELATIVE
04 = SON-IN-LAW OR DAUGHTER-IN-LAW	10 = ADOPTED
05 = GRANDCHILD	11 = FOSTER
06 = PARENT	12 = STEPCHILD
	13 = NOT RELATED
	98 = DON'T KNOW

					IF AGE 0-17 YEARS				IF AGE 0-5		IF AGE 5 YEARS OR OLDER		IF AGE 5-24 YEARS	
LINE NO.	ELIGIBILITY				SURVIVORSHIP AND RESIDENCE OF BIOLOGICAL PARENTS				ELIGIBILITY		EVER ATTENDED SCHOOL		CURRENT/RECENT SCHOOL ATTENDANCE	
	9	9A	10	11	12	13	14	15	15A	15B	16	17	18	19
	CIRCLE LINE NUMBER OF ALL WOMEN AGE 15-49 OR, IF HOUSEHOLD SELECTED FOR MALE SURVEY BIO-MARKERS, CIRCLE LINE NUMBER OF ALL WOMEN AGE 15 AND OLDER	CIRCLE LINE NUMBER OF ALL WOMEN AGE 18 AND OLDER	**IF HOUSEHOLD SELECTED FOR MALE SURVEY AND BIO-MARKERS** CIRCLE LINE NUMBER OF ALL MEN AGE 15 AND OLDER	**IF HOUSEHOLD SELECTED FOR MALE SURVEY AND BIO-MARKERS** CIRCLE LINE NUMBER OF ALL CHILDREN AGE 0-5	Is (NAME)'s biological mother alive?	Does (NAME)'s biological mother usually live in this household or was she a guest last night? IF YES: What is her name? RECORD MOTHER'S LINE NUMBER. IF NO, RECORD '00'.	Is (NAME)'s biological father alive?	Does (NAME)'s biological father usually live in this household or was he a guest last night? IF YES: What is his name? RECORD FATHER'S LINE NUMBER. IF NO, RECORD '00'.	CHECK 13: IF MOTHER LIVES IN HOUSEHOLD, SKIP TO 16. IF MOTHER HAS DIED OR DOES NOT LIVE IN THE HOUSEHOLD, CIRCLE LINE NUMBER OF CHILD.	Who is the primary caregiver of (NAME)? RECORD CAREGIVER'S LINE NUMBER.	Has (NAME) ever attended an educational institution?	What is the highest level of education that (NAME) has attended? What is the highest grade (NAME) completed at that level? SEE CODES BELOW.	Did (NAME) attend an educational institution at any time during the 2016 academic year?	During [this/that] academic year, what level and grade [is/was] (NAME) attending? SEE CODES BELOW.
01	01	01	01	01	Y N DK 1 2 8 GO TO 14		Y N DK 1 2 8 GO TO 15A		01	LINE NO.	Y N 1 2 GO TO 20		Y N 1 2 GO TO 20	
02	02	02	02	02	1 2 8 GO TO 14		1 2 8 GO TO 15A		02		1 2 GO TO 20		1 2 GO TO 20	
03	03	03	03	03	1 2 8 GO TO 14		1 2 8 GO TO 15A		03		1 2 GO TO 20		1 2 GO TO 20	
04	04	04	04	04	1 2 8 GO TO 14		1 2 8 GO TO 15A		04		1 2 GO TO 20		1 2 GO TO 20	
05	05	05	05	05	1 2 8 GO TO 14		1 2 8 GO TO 15A		05		1 2 GO TO 20		1 2 GO TO 20	
06	06	06	05	06	1 2 8 GO TO 14		1 2 8 GO TO 15A		06		1 2 GO TO 20		1 2 GO TO 20	
07	07	07	07	07	1 2 8 GO TO 14		1 2 8 GO TO 15A		07		1 2 GO TO 20		1 2 GO TO 20	

CODES FOR Qs. 17 AND 19: EDUCATION

PRE-PRIMARY SCHOOL
00 = LESS THAN 1 YEAR PRE-PRIMARY COMPLETED
(USE '00' FOR Q. 17 ONLY. THIS CODE IS NOT ALLOWED FOR Q. 19.)
01=GRADE R/GRADE 0/RECEPTION

PRIMARY SCHOOL
10=LESS THAN 1 YEAR PRIMARY SCHOOL COMPLETED
(USE '10' FOR Q. 17 ONLY. THIS CODE IS NOT ALLOWED FOR Q. 19
11=GRADE 1/SUB A/CLASS 1
12=GRADE 2/SUB B/CLASS 2
13=GRADE 3/STANDARD 1/AET 1 (KHA RI GUDE, SANLI)
14=GRADE 4/STANDARD 2
15=GRADE 5/STANDARD 3/AET 2
16=GRADE 6 /STANDARD 4
17=GRADE 7/STANDARD 5/AET 3

SECONDARY SCHOOL
20=LESS THAN 1 YEAR SECONDARY SCHOOL COMPLETED
(USE '20' FOR Q. 17 ONLY. THIS CODE IS NOT ALLOWED FOR Q. 19.)
21=GRADE 8/STANDARD 6/FORM 1/NTC 1/N1/NC (V) LEVEL 2
22=GRADE 9/STANDARD 7/FORM 2/AET 4/NTC 2/N2/NC (V) LEVEL 3
23=GRADE 10/STANDARD 8/FORM 3/NTC 3/N3/NC (V) LEVEL 4
24=GRADE 11/STANDARD 9/FORM 4
25=CERTIFICATE OR DIPLOMA WITH LESS THAN GRADE 12/ STANDARD 10 COMPLETED
26=GRADE 12/STANDARD 10/FORM 5/MATRIC
27=N4/NTC4
28=N5/NTC5
29=N6/NTC6

HIGHER EDUCATION
30=FURTHER STUDIES INCOMPLETE OR ONGOING
31=CERTIFICATE OR DIPLOMA WITH GRADE 12/ STANDARD 10 COMPLETED
32=HIGHER DIPLOMA (TECHNIKON/ UNIVERSITY OF TECHNOLOGY)
33=POST HIGHER DIPLOMA (TECHNIKON/ UNIVERSITY OF TECHNOLOGY MASTERS, DOCTORAL)
34=BACHELORS DEGREE/BACHELORS DEGREE AND POST GRADUATE DIPLOMA
35=HONOURS DEGREE
36=HIGHER DEGREE (MASTERS, DOCTORATE)

98 = DON'T KNOW

LINE NO.	IF AGE 5 YEARS OR OLDER							
	PROBLEM OF VISION	PROBLEM OF HEARING	PROBLEM OF WALKING	PROBLEM OF REMEMBERING	PROBLEM WITH SELF-CARE	PROBLEM OF COMMUNICATING	GOVERNMENT GRANTS	
	20	21	22	23	24	25	26	27
	Does (NAME) have difficulty seeing, even if wearing glasses?<br><br>IF NO, CIRCLE "0".<br><br>IF YES, PROBE: With some difficulty, with a lot of difficulty, or cannot see at all?<br><br>IF WITH SOME DIFFICULTY, CIRCLE "1". IF WITH A LOT OF DIFFICULTY, CIRCLE "2". IF CANNOT SEE AT ALL, CIRCLE "3". IF DON'T KNOW CIRCLE "8".	Does (NAME) have difficulty hearing, even if wearing a hearing aid?<br><br>IF NO, CIRCLE "0".<br><br>IF YES, PROBE: With some difficulty, with a lot of difficulty, or cannot hear at all?<br><br>IF WITH SOME DIFFICULTY, CIRCLE "1". IF WITH A LOT OF DIFFICULTY, CIRCLE "2". IF CANNOT HEAR AT ALL, CIRCLE "3". IF DON'T KNOW CIRCLE "8".	Does (NAME) have difficulty walking a kilometre or climbing a flight of steps?<br><br>IF NO, CIRCLE "0".<br><br>IF YES, PROBE: With some difficulty, with a lot of difficulty, or cannot walk or climb steps at all?<br><br>IF WITH SOME DIFFICULTY, CIRCLE "1". IF WITH A LOT OF DIFFICULTY, CIRCLE "2". IF CANNOT WALK OR CLIMB AT ALL, CIRCLE "3". IF DON'T KNOW CIRCLE "8".	Does (NAME) have difficulty remembering or concentrating?<br><br>IF NO, CIRCLE "0".<br><br>IF YES, PROBE: With some difficulty, with a lot of difficulty, or cannot remember or concentrate at all?<br><br>IF WITH SOME DIFFICULTY, CIRCLE "1". IF WITH A LOT OF DIFFICULTY, CIRCLE "2". IF CANNOT REMEMBER OR CONCENTRATE AT ALL, CIRCLE "3". IF DON'T KNOW CIRCLE "8".	Does (NAME) have difficulty with self-care such as washing all over or dressing?<br><br>IF NO, CIRCLE "0".<br><br>IF YES, PROBE: With some difficulty, with a lot of difficulty, or cannot do at all?<br><br>IF WITH SOME DIFFICULTY, CIRCLE "1". IF WITH A LOT OF DIFFICULTY, CIRCLE "2". IF CANNOT DO AT ALL, CIRCLE "3". IF DON'T KNOW CIRCLE "8".	Does (NAME) have difficulty communicating in (his/her) usual language? For example, understanding others or others understanding (him/her)?<br><br>IF NO, CIRCLE "0".<br><br>IF YES, PROBE: With some difficulty, with a lot of difficulty, or cannot communicate at all?<br><br>IF WITH SOME DIFFICULTY, CIRCLE "1". IF WITH A LOT OF DIFFICULTY, CIRCLE "2". IF CANNOT COMMUNICATE AT ALL, CIRCLE "3". IF DON'T KNOW CIRCLE "8".	Does (NAME) receive any social grant, old age grant, or social relief assistance from the government ?	What type of government grant does (NAME) receive?
	N YS YA YT DK	N YS YA YT DK	N YS YA YT DK	N YS YA YT DK	N YS YA YT DK	N YS YA YT DK	Y N	
01	0 1 2 3 8	0 1 2 3 8	0 1 2 3 8	0 1 2 3 8	0 1 2 3 8	0 1 2 3 8	1 2 ↓ NEXT LINE	
02	0 1 2 3 8	0 1 2 3 8	0 1 2 3 8	0 1 2 3 8	0 1 2 3 8	0 1 2 3 8	1 2 ↓ NEXT LINE	
03	0 1 2 3 8	0 1 2 3 8	0 1 2 3 8	0 1 2 3 8	0 1 2 3 8	0 1 2 3 8	1 2 ↓ NEXT LINE	
04	0 1 2 3 8	0 1 2 3 8	0 1 2 3 8	0 1 2 3 8	0 1 2 3 8	0 1 2 3 8	1 2 ↓ NEXT LINE	
05	0 1 2 3 8	0 1 2 3 8	0 1 2 3 8	0 1 2 3 8	0 1 2 3 8	0 1 2 3 8	1 2 ↓ NEXT LINE	
06	0 1 2 3 8	0 1 2 3 8	0 1 2 3 8	0 1 2 3 8	0 1 2 3 8	0 1 2 3 8	1 2 ↓ NEXT LINE	
07	0 1 2 3 8	0 1 2 3 8	0 1 2 3 8	0 1 2 3 8	0 1 2 3 8	0 1 2 3 8	1 2 ↓ NEXT LINE	

CODES FOR Q. 27 : GOVT GRANTS

01 = OLD AGE (60-74; R1500; 75+; R1520)
02 = DISABILITY (18-59; R1500)
03 = CHILD SUPPORT (0-17; R350)
04 = CARE DEPENDENCY (0-17; R1500)
05 = FOSTER CHILD (<22; R890)
06 = WAR VETERAN (60+; R1520)
07 = IN-AID + OLD AGE (60-74; R1850; 75+; R1870)
08 = IN-AID + DISABILITY (18-59; R1850)
09 = IN-AID + WAR VETERAN (60+; R1870)
10 = SOCIAL RELIEF OF DISTRESS
98 = DON'T KNOW

TABLE FOR SELECTION OF WOMEN FOR THE HOUSEHOLD RELATIONS QUESTIONS

LOOK AT THE LAST DIGIT OF THE HOUSEHOLD QUESTIONNAIRE SERIAL NUMBER ON THE COVER PAGE. THIS IS THE ROW NUMBER YOU SHOULD GO TO. CHECK THE TOTAL NUMBER OF ELIGIBLE WOMEN IN COLUMN 9A OF THE HOUSEHOLD SCHEDULE. THIS IS THE COLUMN NUMBER YOU SHOULD GO TO. FOLLOW THE SELECTED ROW AND COLUMN TO THE CELL WHERE THEY MEET AND CIRCLE THE NUMBER IN THE CELL. THIS IS THE NUMBER OF THE WOMAN SELECTED FOR THE HOUSEHOLD RELATIONS QUESTIONS FROM THE LIST OF ELIGIBLE WOMEN IN COLUMN 9A OF THE HOUSEHOLD SCHEDULE. WRITE THE NAME AND LINE NUMBER OF THE SELECTED WOMAN IN THE SPACE BELOW THE TABLE.

EXAMPLE: THE HOUSEHOLD QUESTIONNAIRE SERIAL NUMBER IS '716' AND THE HOUSEHOLD SCHEDULE COLUMN 9A SHOWS THAT THERE ARE THREE ELIGIBLE WOMEN IN THE HOUSEHOLD (LINE NUMBERS 02, 04, AND 05). SINCE THE LAST DIGIT OF THE HOUSEHOLD SERIAL NUMBER IS '6' GO TO ROW '6' AND SINCE THERE ARE THREE ELIGIBLE WOMEN IN THE HOUSEHOLD, GO TO COLUMN '3'. FOLLOW THE ROW AND COLUMN AND FIND THE NUMBER IN THE CELL WHERE THEY MEET ('2') AND CIRCLE THE NUMBER. NOW GO TO THE HOUSEHOLD SCHEDULE AND FIND THE SECOND WOMAN WHO IS ELIGIBLE FOR THE WOMAN'S INTERVIEW (LINE NUMBER '04' IN THIS EXAMPLE). WRITE HER NAME AND LINE NUMBER IN THE SPACE BELOW THE TABLE.

LAST DIGIT OF THE HOUSEHOLD QUESTIONNAIRE SERIAL NUMBER	TOTAL NUMBER OF ELIGIBLE WOMEN IN HOUSEHOLD SCHEDULE COLUMN 9A							
	1	2	3	4	5	6	7	8
0	1	2	2	4	3	6	5	4
1	1	1	3	1	4	1	6	5
2	1	2	1	2	5	2	7	6
3	1	1	2	3	1	3	1	7
4	1	2	3	4	2	4	2	8
5	1	1	1	1	3	5	3	1
6	1	2	2	2	4	6	4	2
7	1	1	3	3	5	1	5	3
8	1	2	1	4	1	2	6	4
9	1	1	2	1	2	3	7	5

NAME OF SELECTED WOMAN ____________________

HOUSEHOLD LINE NUMBER OF SELECTED WOMAN . . ☐☐

NO.	QUESTIONS AND FILTERS	CODING CATEGORIES	SKIP
101	What is the main source of drinking water for members of your household?	**PIPED WATER**<br>PIPED INTO DWELLING/HOUSE . . . . 11<br>PIPED TO YARD/PLOT . . . . 12<br>PIPED TO NEIGHBOUR . . . . 13<br>PUBLIC/COMMUNAL TAP . . . . 14<br><br>BOREHOLE . . . . 21<br>**DUG WELL**<br>PROTECTED WELL . . . . 31<br>UNPROTECTED WELL . . . . 32<br>**WATER FROM SPRING**<br>PROTECTED SPRING . . . . 41<br>UNPROTECTED SPRING . . . . 42<br><br>RAINWATER . . . . 51<br>WATER-CARRIER/TANKER TRUCK . . . . 61<br>CART WITH SMALL TANK/WATER VENDOR . . 71<br>SURFACE WATER (RIVER/DAM/<br>LAKE/POND/STREAM/CANAL/<br>IRRIGATION CHANNEL) . . . . 81<br>BOTTLED WATER . . . . 91<br><br>OTHER ______________________ 96<br>(SPECIFY)	11, 12 → 106<br>14–81 → 103<br>96 → 103
102	What is the main source of water used by your household for other purposes such as cooking and handwashing?	**PIPED WATER**<br>PIPED INTO DWELLING/HOUSE . . . . 11<br>PIPED TO YARD/PLOT . . . . 12<br>PIPED TO NEIGHBOUR . . . . 13<br>PUBLIC/COMMUNAL TAP . . . . 14<br><br>BOREHOLE . . . . 21<br>**DUG WELL**<br>PROTECTED WELL . . . . 31<br>UNPROTECTED WELL . . . . 32<br>**WATER FROM SPRING**<br>PROTECTED SPRING . . . . 41<br>UNPROTECTED SPRING . . . . 42<br><br>RAINWATER . . . . 51<br>WATER-CARRIER/TANKER TRUCK . . . . 61<br>CART WITH SMALL TANK/WATER VENDOR . . 71<br>SURFACE WATER (RIVER/DAM/<br>LAKE/POND/STREAM/CANAL/<br>IRRIGATION CHANNEL) . . . . 81<br><br>OTHER ______________________ 96<br>(SPECIFY)	11, 12 → 106
103	Where is that water source located?	IN OWN DWELLING . . . . 1<br>IN OWN YARD/PLOT . . . . 2<br>ELSEWHERE/OUTSIDE YARD 3	1, 2 → 105
104	How long does it take to go there, get water, and come back?	MINUTES . . . . [ ][ ][ ]<br><br>DON'T KNOW 998	
105	CHECK 101 AND 102: CODE '14' OR '21' CIRCLED?<br>YES [ ] ↓	NO [ ]	→ 107
106	In the past two weeks, was the water from this source not available for at least one full day?	YES . . . . 1<br>NO . . . . 2<br>DON'T KNOW 8	
107	Do you do anything to the water to make it safer to drink?	YES, ALWAYS . . . . 1<br>YES, SOMETIMES . . . . 2<br>NO . . . . 3<br>DON'T KNOW 8	3, 8 → 109

NO.	QUESTIONS AND FILTERS	CODING CATEGORIES		SKIP
108	What do you usually do to make the water safer to drink?<br><br>Anything else?<br><br>RECORD ALL MENTIONED.	BOIL<br>ADD BLEACH/CHLORINE/JIK<br>STRAIN THROUGH A CLOTH<br>USE WATER FILTER (CERAMIC/ SAND/COMPOSITE/ETC)<br>SOLAR DISINFECTION<br>LET IT STAND AND SETTLE<br>OTHER ______ (SPECIFY)<br>DON'T KNOW	A<br>B<br>C<br>D<br>E<br>F<br>X<br>Z	
109	What kind of toilet facility do members of your household usually use?<br><br>IF NOT POSSIBLE TO DETERMINE, ASK PERMISSION TO OBSERVE THE FACILITY.	**FLUSH OR POUR FLUSH TOILET**<br>FLUSH TO PIPED SEWER SYSTEM<br>FLUSH TO SEPTIC TANK<br>FLUSH TO PIT LATRINE<br>FLUSH TO SOMEWHERE ELSE<br>FLUSH, DON'T KNOW WHERE<br>**PIT LATRINE**<br>VENTILATED IMPROVED PIT LATRINE<br>PIT LATRINE WTH VENTILATION PIPE BUT NO GAUZE MESH/NETTING<br>PIT LATRINE WTHOUT VENTILATION PIPE<br>COMPOSTING TOILET/ ECOLOGICAL SANITATION SYSTEM<br>CHEMICAL TOILET<br>BUCKET TOILET<br>NO FACILITY/BUSH/FIELD<br>OTHER ______ (SPECIFY)	 11<br>12<br>13<br>14<br>15<br><br>21<br>22<br>23<br>31<br>41<br>51<br>61<br>96	61 → 113
110	Do you share this toilet facility with other households?	YES<br>NO	1<br>2	2 → 112
111	Including your own household, how many households use this toilet facility?	NO. OF HOUSEHOLDS IF LESS THAN 10<br>10 OR MORE HOUSEHOLDS<br>DON'T KNOW	0 _<br>95<br>98	
112	Where is this toilet facility located?	IN OWN DWELLING<br>IN OWN YARD/PLOT<br>ELSEWHERE/OUTSIDE YARD	1<br>2<br>3	
113	What type of energy/fuel does your household mainly use for cooking?	ELECTRICITY FROM MAINS<br>ELECTRICITY FROM GENERATOR<br>ELECTRICITY FROM OTHER SOURCE<br>SOLAR ENERGY<br>GAS<br>PARAFFIN<br>COAL<br>WOOD<br>STRAW/SHRUBS/GRASS<br>AGRICULTURAL CROP<br>ANIMAL DUNG<br>NO FOOD COOKED IN HOUSEHOLD<br>OTHER ______ (SPECIFY)	01<br>02<br>03<br>04<br>05<br>06<br>07<br>08<br>09<br>10<br>11<br>95<br>96	95 → 116
114	Is the cooking usually done in the house, in a separate building, or outdoors?	IN THE HOUSE<br>IN A SEPARATE BUILDING<br>OUTDOORS<br>OTHER ______ (SPECIFY)	1<br>2<br>3<br>6	3, 6 → 116
115	Do you have a separate room which is used as a kitchen?	YES<br>NO	1<br>2	
116	How many rooms in this household are used for sleeping?	ROOMS	_ _	

HOUSEHOLD CHARACTERISTICS

NO.	QUESTIONS AND FILTERS	CODING CATEGORIES	SKIP
116A	What type of energy/fuel does your household mainly use for heating/warming?	ELECTRICITY FROM MAINS . . . . . 01<br>ELECTRICITY FROM GENERATOR . . . . . 02<br>ELECTRICITY FROM OTHER SOURCE . . . . . 03<br>SOLAR ENERGY . . . . . 04<br>GAS . . . . . 05<br>PARAFFIN . . . . . 06<br>COAL . . . . . 07<br>WOOD . . . . . 08<br>STRAW/SHRUBS/GRASS . . . . . 09<br>AGRICULTURAL CROP . . . . . 10<br>ANIMAL DUNG . . . . . 11<br><br>NO HEATING/WARMING IN HOUSEHOLD . . . . . 95<br><br>OTHER ________ 96<br>(SPECIFY)	
117	Does this household own any livestock, herds, other farm animals, or poultry?	YES . . . . . 1<br>NO 2	 → 120
118	How many of the following animals does this household own?<br>IF NONE, RECORD '00'.<br>IF 95 OR MORE, RECORD '95'.<br>IF UNKNOWN, RECORD '98'.<br><br>a) Cattle?<br>b) Horses, donkeys, or mules?<br>c) Goats?<br>d) Sheep?<br>e) Pigs?<br>f) Chickens or other poultry?	 <br><br><br><br>a) CATTLE . . . . . [ ][ ]<br>b) HORSES/DONKEYS/MULES . . . . . [ ][ ]<br>c) GOATS . . . . . [ ][ ]<br>d) SHEEP . . . . . [ ][ ]<br>e) PIGS . . . . . [ ][ ]<br>f) CHICKENS/POULTRY . . . . . [ ][ ]	
120	CHECK 113 AND 116A: CODE '01' CIRCLED IN EITHER?<br>NO [ ] ↓	YES [ ] →	121
121A	Does your household have electricity that is connected to the mains?	YES . . . . . 1<br>NO 2	
121	Does your household have any of the following in working condition:<br><br>b) A radio?<br>c) A television?<br>d) A landline telephone?<br>e) A desktop or laptop computer?<br>f) A refrigerator?<br>g) A vacuum cleaner or floor polisher?<br>h) A microwave oven?<br>i) An electric or gas stove?<br>j) A washing machine?	YES NO<br><br>b) RADIO . . . . . 1 2<br>c) TELEVISION . . . . . 1 2<br>d) LANDLINE TELEPHONE . . 1 2<br>e) COMPUTER . . . . . 1 2<br>f) REFRIGERATOR . . . . . 1 2<br>g) VACUUM CLEANER . . . . . 1 2<br>h) MICROWAVE OVEN . . . . . 1 2<br>i) STOVE . . . . . 1 2<br>k) WASHING MACHINE 1 2	
122	Does any member of this household own any of the following in working condition:<br><br>a) A watch?<br>b) A cell phone?<br>c) A bicycle?<br>d) A motorcycle or motor scooter?<br>e) An animal-drawn cart?<br>f) A car, bakkie, van or truck?<br>g) A boat with a motor?	YES NO<br><br>a) WATCH . . . . . 1 2<br>b) CELL PHONE . . . . . 1 2<br>c) BICYCLE . . . . . 1 2<br>d) MOTORCYCLE/SCOOTER . . . . . 1 2<br>e) ANIMAL-DRAWN CART . . . . . 1 2<br>f) CAR/BAKKIE/VAN/TRUCK . . 1 2<br>g) BOAT WITH MOTOR 1 2	
124	How often does anyone smoke inside your house? Would you say daily, weekly, monthly, less often than once a month, or never?	DAILY . . . . . 1<br>WEEKLY . . . . . 2<br>MONTHLY . . . . . 3<br>LESS OFTEN THAN ONCE A MONTH . . . . . 4<br>NEVER 5	

NO.	QUESTIONS AND FILTERS	CODING CATEGORIES	SKIP
124A	How is the refuse or rubbish in this household mainly collected or removed?<br><br>PROBE: How often is it removed?	REMOVED BY LOCAL AUTHORITY/PRIVATE COMPANY AT LEAST ONCE A WEEK . . . . . 01<br>REMOVED BY LOCAL AUTHORITY/PRIVATE COMPANY LESS OFTEN THAN ONCE A WEEK 02<br>REMOVED BY COMMUNITY MEMBERS, CONTRACTED BY THE MUNICIPALITY AT LEAST ONCE A WEEK . . . . 03<br>REMOVED BY COMMUNITY MEMBERS, CONTRACTED BY THE MUNICIPALITY LESS OFTEN THAN ONCE A WEEK . . . . 04<br>REMOVED BY COMMUNITY MEMBERS AT LEAST ONCE A WEEK . . . . 05<br>REMOVED BY COMMUNITY MEMBERS LESS OFTEN THAN ONCE A WEEK . . . . 06<br>COMMUNAL REFUSE DUMP . . . . 07<br>COMMUNAL CONTAINER/CENTRAL COLLECTION POINT . . . . 08<br>OWN REFUSE DUMP . . . . 09<br>OWN REFUSE BURNED . . . . 10<br><br>NO RUBBISH DISPOSAL/DUMP OR LEAVE ANYWHERE . . . . 11<br><br>OTHER ______________________ 96<br>(SPECIFY)	
124B	Do you know where you can get forms to apply for a government grant such as a child or old-age grant?	YES . . . . 1<br>NO 2	 → 124D
124C	Where can you obtain forms?<br><br>RECORD ALL MENTIONED.	POST OFFICE . . . . A<br>BANK . . . . B<br>MAGISTRATE'S COURT . . . . C<br>SASSA/DEPARTMENT OF WELFARE/SOCIAL DEVELOPMENT OFFICE . . . . D<br>PAY POINT . . . . E<br><br>OTHER ______________________ X<br>(SPECIFY)<br>DON'T KNOW/UNSURE . . . . Z	
124D	In the past 12 months, did any adult (18 years and above) in this household go hungry because there wasn't enough food?	NEVER . . . . 1<br>SELDOM . . . . 2<br>SOMETIMES . . . . 3<br>OFTEN . . . . 4<br>ALWAYS . . . . 5<br><br>NOT APPLICABLE/NO ADULTS IN HOUSEHOLD 6	
124E	In the past 12 months, did any child (17 years or younger) in this household go hungry because there wasn't enough food?	NEVER . . . . 1<br>SELDOM . . . . 2<br>SOMETIMES . . . . 3<br>OFTEN . . . . 4<br>ALWAYS . . . . 5<br><br>NOT APPLICABLE/NO CHILDREN IN HOUSEHOLD 6	

NO.	QUESTIONS AND FILTERS	CODING CATEGORIES	SKIP
139	We would like to learn about the places that households use to wash their hands. Can you please show me where members of your household most often wash their hands?	OBSERVED, FIXED PLACE . . . . 1<br>OBSERVED, MOBILE . . . . 2<br>NOT OBSERVED, NOT IN DWELLING/YARD/PLOT 3<br>NOT OBSERVED, NO PERMISSION TO SEE . . 4<br>NOT OBSERVED, OTHER REASON 5	3, 4, 5 → 141A
140	OBSERVE PRESENCE OF WATER AT THE PLACE FOR HANDWASHING.<br><br>RECORD OBSERVATION.	WATER IS AVAILABLE . . . . 1<br>WATER IS NOT AVAILABLE 2	
141	OBSERVE PRESENCE OF SOAP, DETERGENT, OR OTHER CLEANSING AGENT AT THE PLACE FOR HANDWASHING.<br><br>RECORD OBSERVATION.	SOAP OR DETERGENT<br>(BAR, LIQUID, POWDER, PASTE) . . . . A<br>ASH, MUD, SAND . . . . B<br>NONE . . . . Y	
141A	OBSERVE TYPE OF DWELLING<br><br>RECORD OBSERVATION.	DWELLING/HOUSE OR BRICK/CONCRETE<br>BLOCK STRUCTURE ON A SEPARATE STAND/<br>YARD/FARM . . . . 01<br>TRADITIONAL DWELLING/HUT STRUCTURE<br>MADE OF TRADITIONAL MATERIALS . . . . . 02<br>FLAT OR APARTMENT IN BLOCK OF FLATS . . 03<br>CLUSTER HOUSE IN COMPLEX . . . . 04<br>TOWN HOUSE/SEMI-DETACHED HOUSE<br>IN COMPLEX . . . . 05<br>SEMI-DETACHED HOUSE . . . . 06<br>DWELLING/HOUSE/FLAT/ROOM IN BACKYARD 07<br>INFORMAL DWELLING/SHACK IN BACKYARD . . 08<br>INFORMAL DWELLING/SHACK NOT IN BACKYARD<br>(E.G., IN AN INFORMAL/SQUATTER<br>SETTLEMENT OR ON FARM) . . . . 09<br>ROOM/FLATLET ON A PROPERTY OR LARGER<br>DWELLING/SERVANTS' QUARTERS/<br>GRANNY FLAT . . . . 10<br>CARAVAN OR TENT . . . . 11<br><br>OTHER ______________________ 96<br>(SPECIFY)	
142	OBSERVE MAIN MATERIAL OF THE FLOOR OF THE DWELLING.<br><br>RECORD OBSERVATION.	**NATURAL FLOOR**<br>EARTH/SAND . . . . 11<br>DUNG . . . . 12<br>**RUDIMENTARY FLOOR**<br>WOOD PLANKS . . . . 21<br>**FINISHED FLOOR**<br>LAMINATED OR POLISHED WOOD . . . . 31<br>VINYL/ASPHALT STRIPS . . . . 32<br>CERAMIC TILES . . . . 33<br>CEMENT . . . . 34<br>CARPET . . . . 35<br><br>OTHER ______________________ 96<br>(SPECIFY)	
143	OBSERVE MAIN MATERIAL OF THE ROOF OF THE DWELLING.<br><br>RECORD OBSERVATION.	**NATURAL ROOFING**<br>NO ROOF . . . . 11<br>THATCHING/GRASS . . . . 12<br>MUD/SOD . . . . 13<br>**RUDIMENTARY ROOFING**<br>PLASTIC . . . . 21<br>WATTLE AND DAUB . . . . 22<br>MUD WITH CEMENT MIX . . . . 23<br>BRICKS . . . . 24<br>WOOD PLANKS . . . . 25<br>CARDBOARD . . . . 26<br>**FINISHED ROOFING**<br>CORRUGATED IRON/ZINC . . . . 31<br>WOOD . . . . 32<br>ASBESTOS . . . . 33<br>TILES . . . . 34<br>CEMENT . . . . 35<br><br>OTHER ______________________ 96<br>(SPECIFY)	

ADDITIONAL HOUSEHOLD CHARACTERISTICS

NO.	QUESTIONS AND FILTERS	CODING CATEGORIES	SKIP
144	OBSERVE MAIN MATERIAL OF THE EXTERIOR WALLS OF THE DWELLING.<br><br>RECORD OBSERVATION.	**NATURAL WALLS**<br>NO WALLS . . . . 11<br>DIRT/MUD . . . . 13<br>**RUDIMENTARY WALLS**<br>PLASTIC . . . . 21<br>WATTLE AND DAUB . . . . 22<br>STONE WITH MUD . . . . 23<br>MUD WITH CEMENT MIX . . . . 24<br>CARDBOARD . . . . 25<br>REUSED WOOD . . . . 26<br>**FINISHED WALLS**<br>CEMENT . . . . 31<br>STONE WITH LIME/CEMENT . . . . 32<br>BRICKS . . . . 33<br>CEMENT BLOCK/CONCRETE . . . . 34<br>WOOD PLANKS . . . . 36<br>CORRUGATED IRON/ZINC . . . . 37<br><br>OTHER ______ 96<br>(SPECIFY)	
144A	CHECK COVER PAGE: HOUSEHOLD SELECTED FOR SALT COLLECTION?<br>YES ☐ ↓	NO ☐ →	146
145	We would like to check whether the salt used in your household is adequately iodised. May I have a sample of the salt used to cook meals in your household?<br><br>RECORD BAR CODE NUMBER FROM FIRST BAR CODE LABEL IN BOXES. PLACE THE 1ST BAR CODE LABEL ON THE SALT SAMPLE AND THE 2ND ON THE TRANSMITTAL FORM.	BARCODE NUMBER<br>☐☐☐☐☐<br><br>NO SALT IN HOUSEHOLD . . . . 99994<br>REFUSED . . . . 99995<br>OTHER 99996	
146	RECORD THE TIME.	HOURS . . . . ☐☐<br>MINUTES . . . . ☐☐	

INTERVIEWER'S OBSERVATIONS

TO BE FILLED IN AFTER COMPLETING INTERVIEW

COMMENTS ABOUT INTERVIEW:

COMMENTS ON SPECIFIC QUESTIONS:

ANY OTHER COMMENTS:

SUPERVISOR'S OBSERVATIONS

2016 SOUTH AFRICA DEMOGRAPHIC AND HEALTH SURVEY
BIOMARKER QUESTIONNAIRE

IDENTIFICATION

PLACE NAME ____________________

NAME OF HOUSEHOLD HEAD ____________________

CLUSTER NUMBER . . . . [ ][ ][ ][ ]

HOUSEHOLD NUMBER . . . . [ ][ ][ ][ ]

HOUSEHOLD SELECTED FOR MALE SURVEY AND BIOMARKERS? (YES = 1; NO = 2) . . . . [1]

FIELDWORKER VISITS

	1	2	3	FINAL VISIT
DATE<br>FIELDWORKER'S NAME	______<br>______	______<br>______	______<br>______	DAY [ ][ ]<br>MONTH [ ][ ]<br>YEAR [2][0][1][ ]
NEXT VISIT: DATE<br>TIME	______<br>______	______<br>______		TOTAL NUMBER OF VISITS [ ]

NOTES:

____
____
____
____
____

TOTAL ELIGIBLE WOMEN [ ][ ]

TOTAL ELIGIBLE MEN [ ][ ]

TOTAL ELIGIBLE CHILDREN [ ][ ]

LANGUAGE OF QUESTIONNAIRE** [0][1]

LANGUAGE OF INTERVIEW** [ ][ ]

HOME LANGUAGE OF RESPONDENT** [ ][ ]

TRANSLATOR USED (YES = 1, NO = 2) [ ]

LANGUAGE OF QUESTIONNAIRE** ENGLISH

**LANGUAGE CODES:

01 ENGLISH	05 seSOTHO	09 tshiVENDA
02 AFRIKAANS	06 seTSWANA	10 xiTSONGA
03 isiXHOSA	07 sePEDI	11 isiNDEBELE
04 isiZULU	08 siSWATI	12 OTHER

SUPERVISOR

______ NAME

[ ][ ][ ][ ] NUMBER

WEIGHT, HEIGHT AND HAEMOGLOBIN MEASUREMENT FOR CHILDREN AGE 0-5

101	FROM THE LIST OF PERSONS ELIGIBLE FOR BIOMARKERS, RECORD THE LINE NUMBER AND NAME OF ELIGIBLE CHILDREN AGE 0-5 IN THE SAME ORDER THEY APPEAR. IF THERE ARE MORE THAN SIX CHILDREN, USE ADDITIONAL QUESTIONNAIRE(S).			
		CHILD 1	CHILD 2	CHILD 3
102	CHECK LIST OF CHILDREN ELIGIBLE FOR BIOMARKERS:<br>RECORD LINE NUMBER AND NAME.	LINE NUMBER . . . . . . [ ][ ]<br>NAME ________	LINE NUMBER . . . . . . [ ][ ]<br>NAME ________	LINE NUMBER . . . . . . [ ][ ]<br>NAME ________
103	What is (NAME)'s date of birth?	DAY . . . . [ ][ ]<br>MONTH . . . . [ ][ ]<br>YEAR . . . [ ][ ][ ][ ]	DAY . . . . [ ][ ]<br>MONTH . . . . [ ][ ]<br>YEAR . . . [ ][ ][ ][ ]	DAY . . . . [ ][ ]<br>MONTH . . . . [ ][ ]<br>YEAR . . . [ ][ ][ ][ ]
104	CHECK 103: CHILD BORN BETWEEN 2011-2016?	YES . . . . 1<br>NO . . . . 2<br>(SKIP TO 114)	YES . . . . 1<br>NO . . . . 2<br>(SKIP TO 114)	YES . . . . 1<br>NO . . . . 2<br>(SKIP TO 114)
104A	RECORD NAME OF PARENT/OTHER ADULT RESPONSIBLE FOR THE CHILD.	NAME________	NAME________	NAME________
104B	ASK CONSENT FOR ANTHROPOMETRY FROM PARENT/OTHER ADULT.	PROVIDE PARENT/RESPONSIBLE ADULT WITH PARENTAL CONSENT FORM.		
104C	CIRCLE THE CODE AND SIGN YOUR NAME.	GRANTED . . . . 1<br>________ (SIGN)<br>REFUSED . . . . 2<br>NOT PRESENT/OTHER . 3	GRANTED . . . . 1<br>________ (SIGN)<br>REFUSED . . . . 2<br>NOT PRESENT/OTHER . 3	GRANTED . . . . 1<br>________ (SIGN)<br>REFUSED . . . . 2<br>NOT PRESENT/OTHER . 3
105	WEIGHT IN KILOGRAMS.	KG. . . . [ ][ ].[ ][0]<br>NOT PRESENT . . . . 9994<br>REFUSED . . . . 9995<br>OTHER 9996	KG. . . . [ ][ ].[ ][0]<br>NOT PRESENT . . . . 9994<br>REFUSED . . . . 9995<br>OTHER 9996	KG. . . . [ ][ ].[ ][0]<br>NOT PRESENT . . . . 9994<br>REFUSED . . . . 9995<br>OTHER 9996
106	HEIGHT IN CENTIMETRES.	CM. . . . [ ][ ][ ].[ ]<br>NOT PRESENT . . . . 9994<br>REFUSED . . . . 9995<br>OTHER . . . . 9996<br>(SKIP TO 108)	CM. . . . [ ][ ][ ].[ ]<br>NOT PRESENT . . . . 9994<br>REFUSED . . . . 9995<br>OTHER . . . . 9996<br>(SKIP TO 108)	CM. . . . [ ][ ][ ].[ ]<br>NOT PRESENT . . . . 9994<br>REFUSED . . . . 9995<br>OTHER . . . . 9996<br>(SKIP TO 108)
107	MEASURED LYING DOWN OR STANDING UP?	LYING DOWN . . . . . . 1<br>STANDING UP 2	LYING DOWN . . . . . . 1<br>STANDING UP 2	LYING DOWN . . . . . . 1<br>STANDING UP 2
108	MEASURER: ENTER YOUR FIELDWORKER NUMBER.	[ ][ ][ ][ ]<br>FIELDWORKER NUMBER	[ ][ ][ ][ ]<br>FIELDWORKER NUMBER	[ ][ ][ ][ ]<br>FIELDWORKER NUMBER
109	CHECK 103: CHILD AGE 0-5 MONTHS, I.E., WAS CHILD BORN IN MONTH OF INTERVIEW OR 5 PREVIOUS MONTHS?	0-5 MONTHS . . . . 1<br>(SKIP TO 114)<br>OLDER 2	0-5 MONTHS . . . . 1<br>(SKIP TO 114)<br>OLDER 2	0-5 MONTHS . . . . 1<br>(SKIP TO 114)<br>OLDER 2
111	ASK CONSENT FOR ANAEMIA TEST FROM PARENT/OTHER ADULT.	PROVIDE PARENT/RESPONSIBLE ADULT WITH PARENTAL CONSENT FORM.		
112	CIRCLE THE CODE AND SIGN YOUR NAME.	GRANTED . . . . 1<br>________ (SIGN)<br>REFUSED . . . . 2<br>NOT PRESENT/OTHER . 3	GRANTED . . . . 1<br>________ (SIGN)<br>REFUSED . . . . 2<br>NOT PRESENT/OTHER . 3	GRANTED . . . . 1<br>________ (SIGN)<br>REFUSED . . . . 2<br>NOT PRESENT/OTHER . 3
113	RECORD HAEMOGLOBIN LEVEL HERE AND IN THE CHILD HEALTH INFORMATIONAL BROCHURE.	G/DL . . . . [ ][ ].[ ]<br>NOT PRESENT 994<br>REFUSED . . . . 995<br>OTHER 996	G/DL . . . . [ ][ ].[ ]<br>NOT PRESENT 994<br>REFUSED . . . . 995<br>OTHER 996	G/DL . . . . [ ][ ].[ ]<br>NOT PRESENT 994<br>REFUSED . . . . 995<br>OTHER 996
114	GO BACK TO 103 IN NEXT COLUMN OF THIS QUESTIONNAIRE OR IN THE FIRST COLUMN OF THE NEXT PAGE; IF NO MORE CHILDREN, GO TO 201.			

		CHILD 4	CHILD 5	CHILD 6
102	CHECK LIST OF CHILDREN ELIGIBLE FOR BIOMARKERS:<br><br>RECORD LINE NUMBER AND NAME.	LINE NUMBER . . . . . . [ ][ ]<br>NAME ________	LINE NUMBER . . . . . . [ ][ ]<br>NAME ________	LINE NUMBER . . . . . . [ ][ ]<br>NAME ________
103	What is (NAME)'s date of birth?	DAY . . . . [ ][ ]<br>MONTH . . . . [ ][ ]<br>YEAR . . . [ ][ ][ ][ ]	DAY . . . . [ ][ ]<br>MONTH . . . . [ ][ ]<br>YEAR . . . [ ][ ][ ][ ]	DAY . . . . [ ][ ]<br>MONTH . . . . [ ][ ]<br>YEAR . . . [ ][ ][ ][ ]
104	CHECK 103: CHILD BORN BETWEEN 2011-2016?	YES . . . . 1<br>NO . . . . 2<br>(SKIP TO 114)	YES . . . . 1<br>NO . . . . 2<br>(SKIP TO 114)	YES . . . . 1<br>NO . . . . 2<br>(SKIP TO 114)
104A	RECORD NAME OF PARENT/OTHER ADULT RESPONSIBLE FOR THE CHILD.	NAME________	NAME________	NAME________
104B	ASK CONSENT FOR ANTHROPOMETRY FROM PARENT/OTHER ADULT.	PROVIDE PARENT/RESPONSIBLE ADULT WITH PARENTAL CONSENT FORM.		
104C	CIRCLE THE CODE AND SIGN YOUR NAME.	GRANTED . . . . 1<br>________ (SIGN)<br>REFUSED . . . . 2<br>NOT PRESENT/OTHER . 3	GRANTED . . . . 1<br>________ (SIGN)<br>REFUSED . . . . 2<br>NOT PRESENT/OTHER . 3	GRANTED . . . . 1<br>________ (SIGN)<br>REFUSED . . . . 2<br>NOT PRESENT/OTHER . 3
105	WEIGHT IN KILOGRAMS.	KG. . . . [ ][ ].[ ][0]<br>NOT PRESENT . . . . 9994<br>REFUSED . . . . 9995<br>OTHER 9996	KG. . . . [ ][ ].[ ][0]<br>NOT PRESENT . . . . 9994<br>REFUSED . . . . 9995<br>OTHER 9996	KG. . . . [ ][ ].[ ][0]<br>NOT PRESENT . . . . 9994<br>REFUSED . . . . 9995<br>OTHER 9996
106	HEIGHT IN CENTIMETRES.	CM. . . . [ ][ ][ ].[ ]<br>NOT PRESENT . . . . 9994<br>REFUSED . . . . 9995<br>OTHER . . . . 9996<br>(SKIP TO 108)	CM. . . . [ ][ ][ ].[ ]<br>NOT PRESENT . . . . 9994<br>REFUSED . . . . 9995<br>OTHER . . . . 9996<br>(SKIP TO 108)	CM. . . . [ ][ ][ ].[ ]<br>NOT PRESENT . . . . 9994<br>REFUSED . . . . 9995<br>OTHER . . . . 9996<br>(SKIP TO 108)
107	MEASURED LYING DOWN OR STANDING UP?	LYING DOWN . . . . . . 1<br>STANDING UP 2	LYING DOWN . . . . . . 1<br>STANDING UP 2	LYING DOWN . . . . . . 1<br>STANDING UP 2
108	MEASURER: ENTER YOUR FIELDWORKER NUMBER.	[ ][ ][ ][ ]<br>FIELDWORKER NUMBER	[ ][ ][ ][ ]<br>FIELDWORKER NUMBER	[ ][ ][ ][ ]<br>FIELDWORKER NUMBER
109	CHECK 103: CHILD AGE 0-5 MONTHS, I.E., WAS CHILD BORN IN MONTH OF INTERVIEW OR 5 PREVIOUS MONTHS?	0-5 MONTHS . . . . 1<br>(SKIP TO 114)<br>OLDER 2	0-5 MONTHS . . . . 1<br>(SKIP TO 114)<br>OLDER 2	0-5 MONTHS . . . . 1<br>(SKIP TO 114)<br>OLDER 2
111	ASK CONSENT FOR ANAEMIA TEST FROM PARENT/OTHER ADULT.	PROVIDE PARENT/RESPONSIBLE ADULT WITH PARENTAL CONSENT FORM.		
112	CIRCLE THE CODE AND SIGN YOUR NAME.	GRANTED . . . . 1<br>________ (SIGN)<br>REFUSED . . . . 2<br>NOT PRESENT/OTHER . 3	GRANTED . . . . 1<br>________ (SIGN)<br>REFUSED . . . . 2<br>NOT PRESENT/OTHER . 3	GRANTED . . . . 1<br>________ (SIGN)<br>REFUSED . . . . 2<br>NOT PRESENT/OTHER . 3
113	RECORD HAEMOGLOBIN LEVEL HERE AND IN THE CHILD HEALTH INFORMATIONAL BROCHURE.	G/DL . . . . [ ][ ].[ ]<br>NOT PRESENT 994<br>REFUSED . . . . 995<br>OTHER 996	G/DL . . . . [ ][ ].[ ]<br>NOT PRESENT 994<br>REFUSED . . . . 995<br>OTHER 996	G/DL . . . . [ ][ ].[ ]<br>NOT PRESENT 994<br>REFUSED . . . . 995<br>OTHER 996
114	GO BACK TO 103 IN NEXT COLUMN OF THIS QUESTIONNAIRE OR IN THE FIRST COLUMN OF AN ADDITIONAL QUESTIONNAIRE; IF NO MORE CHILDREN, GO TO 201.			

WEIGHT, HEIGHT, WAIST, BLOOD PRESSURE, HAEMOGLOBIN MEASUREMENT, BLOOD COLLECTION FOR HBA1C AND HIV TESTING, AND RECORDING OF MEDICINES FOR WOMEN AGE 15-95

201	FROM THE LIST OF PERSONS ELIGIBLE FOR BIOMARKERS, RECORD THE LINE NUMBER, NAME, AGE, AND MARITAL STATUS FOR ALL ELIGIBLE WOMEN IN 202. WRITE THE NAME OF EACH WOMAN AT THE TOP OF THE FOLLOWING PAGES.<br>IF THERE ARE MORE THAN THREE WOMEN, USE ADDITIONAL QUESTIONNAIRE(S).			
		WOMAN 1	WOMAN 2	WOMAN 3
202	CHECK LIST OF WOMEN ELIGIBLE FOR BIOMARKERS:<br><br>RECORD LINE NUMBER, NAME, AND AGE.<br><br>RECORD MARITAL STATUS.	LINE NUMBER . . . . [ ][ ]<br>NAME ____________<br>AGE . . . . [ ][ ]<br>NEVER IN UNION . . . . 1<br>OTHER 2	LINE NUMBER . . . . [ ][ ]<br>NAME ____________<br>AGE . . . . [ ][ ]<br>NEVER IN UNION . . . . 1<br>OTHER 2	LINE NUMBER . . . . [ ][ ]<br>NAME ____________<br>AGE . . . . [ ][ ]<br>NEVER IN UNION . . . . 1<br>OTHER 2

202A	CHECK 202: AGE	15-17 YEARS . . . . 1<br>18-95 YEARS . . . . 2<br>(SKIP TO 202C)	15-17 YEARS . . . . 1<br>18-95 YEARS . . . . 2<br>(SKIP TO 202C)	15-17 YEARS . . . . 1<br>18-95 YEARS . . . . 2<br>(SKIP TO 202C)
202B	CHECK 202: MARITAL STATUS	NEVER IN UNION . . . . 1<br>(SKIP TO 202E)<br>OTHER 2	NEVER IN UNION . . . . 1<br>(SKIP TO 202E)<br>OTHER 2	NEVER IN UNION . . . . 1<br>(SKIP TO 202E)<br>OTHER 2

ADULT RESP CONSENT

ADULT RESPONDENT CONSENT FOR ANTHROPOMETRY

202C	ASK CONSENT FOR ANTHROPOMETRY.	PROVIDE ADULT RESPONDENT WITH CONSENT FORM.		
202D	CIRCLE THE CODE AND SIGN YOUR NAME.	GRANTED . . . . 1<br>RESPONDENT REFUSED . . . 2<br>____________<br>(SIGN AND SKIP TO 205)<br>NOT PRESENT/OTHER . . . 3<br>(SKIP TO 205)	GRANTED . . . . 1<br>RESPONDENT REFUSED . . . 2<br>____________<br>(SIGN AND SKIP TO 205)<br>NOT PRESENT/OTHER . . . 3<br>(SKIP TO 205)	GRANTED . . . . 1<br>RESPONDENT REFUSED . . . 2<br>____________<br>(SIGN AND SKIP TO 205)<br>NOT PRESENT/OTHER . . . 3<br>(SKIP TO 205)

202E	RECORD NAME OF PARENT/ADULT RESPONSIBLE FOR MINOR.	NAME ____________	NAME ____________	NAME ____________

PARENT—RESP ADULT CONSENT

PARENTAL/RESPONSIBLE ADULT CONSENT FOR ANTHROPOMETRY

202F	ASK CONSENT FOR ANTHROPOMETRY.	PROVIDE PARENT/RESPONSIBLE ADULT WITH PARENTAL CONSENT FORM.		
202G	CIRCLE THE CODE AND SIGN YOUR NAME.	GRANTED . . . . 1<br>PARENT/OTHER RESPONSIBLE ADULT REFUSED . . . . . 2<br>____________<br>(SIGN)<br>(IF REFUSED, SKIP TO 205)<br>NOT PRESENT/OTHER . . . 3<br>(SKIP TO 205)	GRANTED . . . . 1<br>PARENT/OTHER RESPONSIBLE ADULT REFUSED . . . . . 2<br>____________<br>(SIGN)<br>(IF REFUSED, SKIP TO 205)<br>NOT PRESENT/OTHER . . . 3<br>(SKIP TO 205)	GRANTED . . . . 1<br>PARENT/OTHER RESPONSIBLE ADULT REFUSED . . . . . 2<br>____________<br>(SIGN)<br>(IF REFUSED, SKIP TO 205)<br>NOT PRESENT/OTHER . . . 3<br>(SKIP TO 205)

MINOR RESP CONSENT

MINOR RESPONDENT CONSENT FOR ANTHROPOMETRY

202H	ASK CONSENT FOR ANTHROPOMETRY.	PROVIDE MINOR RESPONDENT WITH CONSENT FORM.		
202I	CIRCLE THE CODE AND SIGN YOUR NAME.	GRANTED . . . . 1<br>MINOR RESPONDENT REFUSED . . . . 2<br>____________<br>(SIGN)<br>NOT PRESENT/OTHER . . . 3	GRANTED . . . . 1<br>MINOR RESPONDENT REFUSED . . . . 2<br>____________<br>(SIGN)<br>NOT PRESENT/OTHER . . . 3	GRANTED . . . . 1<br>MINOR RESPONDENT REFUSED . . . . 2<br>____________<br>(SIGN)<br>NOT PRESENT/OTHER . . . 3

WEIGHT, HEIGHT, WAIST, BLOOD PRESSURE, HAEMOGLOBIN MEASUREMENT, BLOOD COLLECTION FOR HBA1C AND HIV TESTING, AND RECORDING OF MEDICINES FOR WOMEN AGE 15-95

		WOMAN 1	WOMAN 2	WOMAN 3
	NAME FROM LIST.	NAME ____________	NAME ____________	NAME ____________
205	WEIGHT IN KILOGRAMS.	KG. . . . [ ][ ][ ].[0]<br>NOT PRESENT . . . . . . 99994<br>REFUSED . . . . 99995<br>OTHER 99996	KG. . . . [ ][ ][ ].[0]<br>NOT PRESENT . . . . . . 99994<br>REFUSED . . . . 99995<br>OTHER 99996	KG. . . . [ ][ ][ ].[0]<br>NOT PRESENT . . . . . . 99994<br>REFUSED . . . . 99995<br>OTHER 99996
206	HEIGHT IN CENTIMETRES.	CM. . . . . . . [ ][ ][ ].[ ]<br>NOT PRESENT . . . . 9994<br>REFUSED . . . . 9995<br>OTHER 9996	CM. . . . . . . [ ][ ][ ].[ ]<br>NOT PRESENT . . . . 9994<br>REFUSED . . . . 9995<br>OTHER 9996	CM. . . . . . . [ ][ ][ ].[ ]<br>NOT PRESENT . . . . 9994<br>REFUSED . . . . 9995<br>OTHER 9996
206A	WAIST CIRCUMFERENCE IN CENTIMETRES.	CM. . . . . . . [ ][ ][ ].[ ]<br>NOT PRESENT . . . . 9994<br>REFUSED . . . . 9995<br>OTHER 9996	CM. . . . . . . [ ][ ][ ].[ ]<br>NOT PRESENT . . . . 9994<br>REFUSED . . . . 9995<br>OTHER 9996	CM. . . . . . . [ ][ ][ ].[ ]<br>NOT PRESENT . . . . 9994<br>REFUSED . . . . 9995<br>OTHER 9996
207	MEASURER: ENTER YOUR FIELDWORKER NUMBER.	[ ][ ][ ][ ]<br>FIELDWORKER NUMBER	[ ][ ][ ][ ]<br>FIELDWORKER NUMBER	[ ][ ][ ][ ]<br>FIELDWORKER NUMBER
208	CHECK 202: AGE	15-17 YEARS . . . . 1<br>18-95 YEARS . . . . 2<br>(SKIP TO 210)	15-17 YEARS . . . . 1<br>18-95 YEARS . . . . 2<br>(SKIP TO 210)	15-17 YEARS . . . . 1<br>18-95 YEARS . . . . 2<br>(SKIP TO 210)
209	CHECK 202: MARITAL STATUS	NEVER IN UNION . . . . 1<br>(SKIP TO 213)<br>OTHER 2	NEVER IN UNION . . . . 1<br>(SKIP TO 213)<br>OTHER 2	NEVER IN UNION . . . . 1<br>(SKIP TO 213)<br>OTHER 2

		WOMAN 1	WOMAN 2	WOMAN 3
	NAME FROM LIST.	NAME ________	NAME ________	NAME ________

ADULT RESPONDENT CONSENT FOR BLOOD PRESSURE MEASUREMENT

ADULT RESPONDENT CONSENT

210	ASK CONSENT FOR BLOOD PRESSURE MEASUREMENT.	PROVIDE ADULT RESPONDENT WITH CONSENT FORM.		
211	CIRCLE THE CODE AND SIGN YOUR NAME.	GRANTED . . . . 1<br>RESPONDENT REFUSED . . . 2<br>________<br>(SIGN)<br>(IF GRANTED, SKIP TO 217;<br>IF REFUSED, SKIP TO 247)<br>NOT PRESENT/OTHER . . . 3<br>(SKIP TO 247)	GRANTED . . . . 1<br>RESPONDENT REFUSED . . . 2<br>________<br>(SIGN)<br>(IF GRANTED, SKIP TO 217;<br>IF REFUSED, SKIP TO 247)<br>NOT PRESENT/OTHER . . . 3<br>(SKIP TO 247)	GRANTED . . . . 1<br>RESPONDENT REFUSED . . . 2<br>________<br>(SIGN)<br>(IF GRANTED, SKIP TO 217;<br>IF REFUSED, SKIP TO 247)<br>NOT PRESENT/OTHER . . . 3<br>(SKIP TO 247)

PARENTAL/RESPONSIBLE ADULT CONSENT FOR BLOOD PRESSURE MEASUREMENT

PARENT—RESP ADULT CONSENT

213	ASK CONSENT FOR BLOOD PRESSURE MEASUREMENT.	PROVIDE PARENT/RESPONSIBLE ADULT WITH PARENTAL CONSENT FORM.		
214	CIRCLE THE CODE AND SIGN YOUR NAME.	GRANTED . . . . 1<br>PARENT/OTHER RESPONSIBLE ADULT REFUSED . . . . . 2<br>________<br>(SIGN)<br>(IF REFUSED, SKIP TO 255)<br>NOT PRESENT/OTHER . . . 3<br>(SKIP TO 255)	GRANTED . . . . 1<br>PARENT/OTHER RESPONSIBLE ADULT REFUSED . . . . . 2<br>________<br>(SIGN)<br>(IF REFUSED, SKIP TO 255)<br>NOT PRESENT/OTHER . . . 3<br>(SKIP TO 255)	GRANTED . . . . 1<br>PARENT/OTHER RESPONSIBLE ADULT REFUSED . . . . . 2<br>________<br>(SIGN)<br>(IF REFUSED, SKIP TO 255)<br>NOT PRESENT/OTHER . . . 3<br>(SKIP TO 255)

MINOR RESPONDENT CONSENT FOR BLOOD PRESSURE MEASUREMENT

MINOR RESPONDENT CONSENT

215	ASK CONSENT FOR BLOOD PRESSURE MEASUREMENT.	PROVIDE MINOR RESPONDENT WITH CONSENT FORM.		
216	CIRCLE THE CODE AND SIGN YOUR NAME.	GRANTED . . . . 1<br>MINOR RESPONDENT REFUSED . . . . 2<br>________<br>(SIGN)<br>(IF REFUSED, SKIP TO 255)<br>NOT PRESENT/OTHER . . . 3<br>(SKIP TO 255)	GRANTED . . . . 1<br>MINOR RESPONDENT REFUSED . . . . 2<br>________<br>(SIGN)<br>(IF REFUSED, SKIP TO 255)<br>NOT PRESENT/OTHER . . . 3<br>(SKIP TO 255)	GRANTED . . . . 1<br>MINOR RESPONDENT REFUSED . . . . 2<br>________<br>(SIGN)<br>(IF REFUSED, SKIP TO 255)<br>NOT PRESENT/OTHER . . . 3<br>(SKIP TO 255)

		WOMAN 1	WOMAN 2	WOMAN 3
	NAME FROM LIST.	NAME ________	NAME ________	NAME ________
217	Before taking your blood pressure, I would like to ask a few questions about things that may affect these measurements. Have you done any of the following within the past 30 minutes:	YES NO	YES NO	YES NO
a)	Eaten anything?	EATEN 1 2	EATEN 1 2	EATEN 1 2
b)	Had coffee, tea, cola or other drink that has caffeine?	HAD CAFFEINATED DRINK 1 2	HAD CAFFEINATED DRINK 1 2	HAD CAFFEINATED DRINK 1 2
c)	Smoked any tobacco product?	SMOKED 1 2	SMOKED 1 2	SMOKED 1 2
d)	Used any other type of tobacco such as chewing tobacco or snuff?	OTHER TOBACCO 1 2	OTHER TOBACCO 1 2	OTHER TOBACCO 1 2
218	May I begin the process of measuring your blood pressure? I will begin by measuring the circumference of your arm to make sure that I use the right equipment.	MEASURE THE CIRCUMFERENCE OF THE RESPONDENT'S ARM MIDWAY BETWEEN THE ELBOW AND THE SHOULDER. RECORD THE MEASUREMENT IN CENTIMETRES.<br>ARM CIRCUMFERENCE (IN CENTIMETRES). [ ][ ]	MEASURE THE CIRCUMFERENCE OF THE RESPONDENT'S ARM MIDWAY BETWEEN THE ELBOW AND THE SHOULDER. RECORD THE MEASUREMENT IN CENTIMETRES.<br>ARM CIRCUMFERENCE (IN CENTIMETRES). [ ][ ]	MEASURE THE CIRCUMFERENCE OF THE RESPONDENT'S ARM MIDWAY BETWEEN THE ELBOW AND THE SHOULDER. RECORD THE MEASUREMENT IN CENTIMETRES.<br>ARM CIRCUMFERENCE (IN CENTIMETRES). [ ][ ]
219	USE THE ARM CIRCUMFERENCE MEASUREMENT TO SELECT THE APPROPRIATE BLOOD PRESSURE MONITOR CUFF SIZE. CIRCLE THE CODE FOR THE CUFF SIZE.	SMALL: 17 CM – 22 CM . . . . . 1<br>MEDIUM: 23 CM – 31 CM . . . . . 2<br>LARGE: 32 CM – 42 CM . . . . . 3<br>EXTRA LARGE: ≥43 CM 4	SMALL: 17 CM – 22 CM . . . . . 1<br>MEDIUM: 23 CM – 31 CM . . . . . 2<br>LARGE: 32 CM – 42 CM . . . . . 3<br>EXTRA LARGE: ≥43 CM 4	SMALL: 17 CM – 22 CM . . . . . 1<br>MEDIUM: 23 CM – 31 CM . . . . . 2<br>LARGE: 32 CM – 42 CM . . . . . 3<br>EXTRA LARGE: ≥43 CM 4
220	RECORD TIME OF FIRST BP READING	HOURS MINUTES<br>[ ][ ] : [ ][ ]	HOURS MINUTES<br>[ ][ ] : [ ][ ]	HOURS MINUTES<br>[ ][ ] : [ ][ ]
221	TAKE THE FIRST BLOOD PRESSURE READING. RECORD THE SYSTOLIC AND DIASTOLIC PRESSURE AND PULSE (HEART RATE).	**FIRST BP MEASURE**<br>SYSTOLIC . . . . [ ][ ][ ]<br>DIASTOLIC . . . . . . [ ][ ][ ]<br>PULSE . . . . [ ][ ][ ]<br>TECHNICAL PROBLEMS . 994<br>REFUSED . . . . 995<br>OTHER . . . . 996<br>(IF NOT MEASURED, GO TO 245)	**FIRST BP MEASURE**<br>SYSTOLIC . . . . [ ][ ][ ]<br>DIASTOLIC . . . . . . [ ][ ][ ]<br>PULSE . . . . [ ][ ][ ]<br>TECHNICAL PROBLEMS . 994<br>REFUSED . . . . 995<br>OTHER . . . . 996<br>(IF NOT MEASURED, GO TO 245)	**FIRST BP MEASURE**<br>SYSTOLIC . . . . [ ][ ][ ]<br>DIASTOLIC . . . . . . [ ][ ][ ]<br>PULSE . . . . [ ][ ][ ]<br>TECHNICAL PROBLEMS . 994<br>REFUSED . . . . 995<br>OTHER . . . . 996<br>(IF NOT MEASURED, GO TO 245)

		WOMAN 1	WOMAN 2	WOMAN 3
	NAME FROM LIST.	NAME ______	NAME ______	NAME ______
222	Before this survey, has your blood pressure ever been checked?	YES . . . . 1<br>NO 2	YES . . . . 1<br>NO 2	YES . . . . 1<br>NO 2
223	Were you told on two or more different occasions by a doctor or other health professional that you had hypertension or high blood pressure?	YES . . . . 1<br>NO 2	YES . . . . 1<br>NO 2	YES . . . . 1<br>NO 2
224	To lower your blood pressure, are you now taking a prescribed medicine?	YES . . . . 1<br>NO 2	YES . . . . 1<br>NO 2	YES . . . . 1<br>NO 2
225	**CHECK THAT IT HAS BEEN AT LEAST 3 MINUTES BEFORE TAKING THE SECOND BLOOD PRESSURE MEASUREMENT**			
226	May I take your blood pressure at this time?	YES . . . . 1<br>NO . . . . 2<br>(GO TO 243)	YES . . . . 1<br>NO . . . . 2<br>(GO TO 243)	YES . . . . 1<br>NO . . . . 2<br>(GO TO 243)
227	RECORD TIME OF SECOND BP READING.	HOURS [ ][ ] : MINUTES [ ][ ]	HOURS [ ][ ] : MINUTES [ ][ ]	HOURS [ ][ ] : MINUTES [ ][ ]
228	TAKE THE SECOND BLOOD PRESSURE READING. RECORD THE SYSTOLIC AND DIASTOLIC PRESSURE AND PULSE (HEART RATE).	**SECOND BP MEASURE**<br>SYSTOLIC . . . . [ ][ ][ ]<br>DIASTOLIC . . . . [ ][ ][ ]<br>PULSE . . . . [ ][ ][ ]<br>TECHNICAL PROBLEMS . 994<br>REFUSED . . . . 995<br>OTHER . . . . 996<br>(IF NOT MEASURED, GO TO 243)	**SECOND BP MEASURE**<br>SYSTOLIC . . . . [ ][ ][ ]<br>DIASTOLIC . . . . [ ][ ][ ]<br>PULSE . . . . [ ][ ][ ]<br>TECHNICAL PROBLEMS . 994<br>REFUSED . . . . 995<br>OTHER . . . . 996<br>(IF NOT MEASURED, GO TO 243)	**SECOND BP MEASURE**<br>SYSTOLIC . . . . [ ][ ][ ]<br>DIASTOLIC . . . . [ ][ ][ ]<br>PULSE . . . . [ ][ ][ ]<br>TECHNICAL PROBLEMS . 994<br>REFUSED . . . . 995<br>OTHER . . . . 996<br>(IF NOT MEASURED, GO TO 243)

		WOMAN 1	WOMAN 2	WOMAN 3
	NAME FROM LIST.	NAME ______________	NAME ______________	NAME ______________
229	**CHECK THAT IT HAS BEEN AT LEAST 3 MINUTES BEFORE TAKING THE THIRD BLOOD PRESSURE MEASUREMENT**			
230	May I take your blood pressure at this time?	YES . . . . 1<br>NO . . . . 2<br>(GO TO 243)	YES . . . . 1<br>NO . . . . 2<br>(GO TO 243)	YES . . . . 1<br>NO . . . . 2<br>(GO TO 243)
231	RECORD TIME OF THIRD BP READING	HOURS [ ][ ] : MINUTES [ ][ ]	HOURS [ ][ ] : MINUTES [ ][ ]	HOURS [ ][ ] : MINUTES [ ][ ]
232	TAKE THE THIRD BLOOD PRESSURE READING. RECORD THE SYSTOLIC AND DIASTOLIC PRESSURE AND PULSE (HEART RATE).	**THIRD BP MEASURE**<br>SYSTOLIC . . . . . . [ ][ ][ ]<br>DIASTOLIC . . . . . . [ ][ ][ ]<br>PULSE . . . . [ ][ ][ ]<br>TECHNICAL PROBLEMS . 994<br>REFUSED . . . . 995<br>OTHER 996	**THIRD BP MEASURE**<br>SYSTOLIC . . . . . . [ ][ ][ ]<br>DIASTOLIC . . . . . . [ ][ ][ ]<br>PULSE . . . . [ ][ ][ ]<br>TECHNICAL PROBLEMS . 994<br>REFUSED . . . . 995<br>OTHER 996	**THIRD BP MEASURE**<br>SYSTOLIC . . . . . . [ ][ ][ ]<br>DIASTOLIC . . . . . . [ ][ ][ ]<br>PULSE . . . . [ ][ ][ ]<br>TECHNICAL PROBLEMS . 994<br>REFUSED . . . . 995<br>OTHER 996

243 CIRCLE THE SINGLE NUMBER WHERE THE FINAL READING OF THE DIASTOLIC AND SYSTOLIC MEASURES MEET.

	**FINAL DIASTOLIC** (WOMAN 1)						**FINAL DIASTOLIC** (WOMAN 2)						**FINAL DIASTOLIC** (WOMAN 3)					
**FINAL SYSTOLIC**	<80	<85	85-89	90-99	100-109	≥110	<80	<85	85-89	90-99	100-109	≥110	<80	<85	85-89	90-99	100-109	≥110
<120	1	2	3	4	5	6	1	2	3	4	5	6	1	2	3	4	5	6
<130	2	2	3	4	5	6	2	2	3	4	5	6	2	2	3	4	5	6
130-139	3	3	3	4	5	6	3	3	3	4	5	6	3	3	3	4	5	6
140-159	4	4	4	4	5	6	4	4	4	4	5	6	4	4	4	4	5	6
160-179	5	5	5	5	5	6	5	5	5	5	5	6	5	5	5	5	5	6
≥180	6	6	6	6	6	6	6	6	6	6	6	6	6	6	6	6	6	6

244 LOCATE THE NUMBER YOU CIRCLED IN 243 IN THE CHART BELOW. THEN USE THE INSTRUCTIONS TO THE RIGHT OF THAT NUMBER TO COMPLETE A BLOOD PRESSURE REPORT AND REFERRAL FORM FOR THE RESPONDENT. GIVE THE FORM TO THE RESPONDENT AND ANSWER ANY QUESTIONS.

NUMBER CIRCLED IN 243	RESPONDENT'S BLOOD PRESSURE CATEGORY	CONSULT HEALTH PROVIDER TO CHECK BLOOD PRESSURE WITHIN:
1	NORMAL (OPTIMAL)	1 YEAR
2	NORMAL (MILDLY HIGH)	1 YEAR
3	NORMAL (MODERATELY HIGH)	2 MONTHS
4	ABNORMAL (MILDLY ELEVATED)	1 MONTH
5	ABNORMAL (MODERATELY ELEVATED)	1 DAY/IMMEDIATELY
6	ABNORMAL (SEVERELY ELEVATED)	IMMEDIATELY

		WOMAN 1	WOMAN 2	WOMAN 3
245	CHECK 202: AGE	15-17 YEARS . . . . 1<br>18-95 YEARS . . . . 2<br>(SKIP TO 247)	15-17 YEARS . . . . 1<br>18-95 YEARS . . . . 2<br>(SKIP TO 247)	15-17 YEARS . . . . 1<br>18-95 YEARS . . . . 2<br>(SKIP TO 247)
246	CHECK 202: MARITAL STATUS	NEVER IN UNION . . . . 1<br>(SKIP TO 255)<br>OTHER 2	NEVER IN UNION . . . . 1<br>(SKIP TO 255)<br>OTHER 2	NEVER IN UNION . . . . 1<br>(SKIP TO 255)<br>OTHER 2

		WOMAN 1	WOMAN 2	WOMAN 3
	NAME FROM LIST.	NAME ______	NAME ______	NAME ______
**ADULT RESPONDENT CONSENT FOR ANAEMIA TEST**				
247	ASK CONSENT FOR ANAEMIA TEST.	PROVIDE ADULT RESPONDENT WITH CONSENT FORM.		
248	CIRCLE THE CODE AND SIGN YOUR NAME.	GRANTED . . . . . 1<br>RESPONDENT REFUSED . . . 2<br>______<br>(SIGN)<br>(IF REFUSED, SKIP TO 249)<br>NOT PRESENT/OTHER . . . 3<br>(SKIP TO 249)	GRANTED . . . . . 1<br>RESPONDENT REFUSED . . . 2<br>______<br>(SIGN)<br>(IF REFUSED, SKIP TO 249)<br>NOT PRESENT/OTHER . . . 3<br>(SKIP TO 249)	GRANTED . . . . . 1<br>RESPONDENT REFUSED . . . 2<br>______<br>(SIGN)<br>(IF REFUSED, SKIP TO 249)<br>NOT PRESENT/OTHER . . . 3<br>(SKIP TO 249)
248A	CHECK 202: AGE	15-49 YEARS . . . . 1<br>50-95 YEARS . . . . 2<br>(SKIP TO 249)	15-49 YEARS . . . . 1<br>50-95 YEARS . . . . 2<br>(SKIP TO 249)	15-49 YEARS . . . . 1<br>50-95 YEARS . . . . 2<br>(SKIP TO 249)
248B	Are you pregnant?	YES . . . . . 1<br>NO . . . . . 2<br>DON'T KNOW 8	YES . . . . . 1<br>NO . . . . . 2<br>DON'T KNOW 8	YES . . . . . 1<br>NO . . . . . 2<br>DON'T KNOW 8
**ADULT RESPONDENT CONSENT FOR HBA1C TESTING**				
249	ASK CONSENT FOR HBA1C TESTING.	PROVIDE ADULT RESPONDENT WITH CONSENT FORM.		
250	CIRCLE THE CODE, SIGN YOUR NAME, AND ENTER YOUR FIELDWORKER NUMBER.	GRANTED . . . . . 1<br>RESPONDENT REFUSED . . . 2<br>______<br>SIGN<br>NOT PRESENT/OTHER . . . 3<br>(SKIP TO 271)	GRANTED . . . . . 1<br>RESPONDENT REFUSED . . . 2<br>______<br>SIGN<br>NOT PRESENT/OTHER . . . 3<br>(SKIP TO 271)	GRANTED . . . . . 1<br>RESPONDENT REFUSED . . . 2<br>______<br>SIGN<br>NOT PRESENT/OTHER . . . 3<br>(SKIP TO 271)
**ADULT RESPONDENT CONSENT FOR HIV TESTING**				
251	ASK CONSENT FOR HIV TESTING.	PROVIDE ADULT RESPONDENT WITH CONSENT FORM.		
252	CIRCLE THE CODE AND SIGN YOUR NAME.	GRANTED . . . . . 1<br>RESPONDENT REFUSED . . . 2<br>______<br>(SIGN AND ENTER YOUR FIELDWORKER NUMBER)<br>[ ][ ][ ][ ]<br>(IF REFUSED, SKIP TO 271)	GRANTED . . . . . 1<br>RESPONDENT REFUSED . . . 2<br>______<br>(SIGN AND ENTER YOUR FIELDWORKER NUMBER)<br>[ ][ ][ ][ ]<br>(IF REFUSED, SKIP TO 271)	GRANTED . . . . . 1<br>RESPONDENT REFUSED . . . 2<br>______<br>(SIGN AND ENTER YOUR FIELDWORKER NUMBER)<br>[ ][ ][ ][ ]<br>(IF REFUSED, SKIP TO 271)
**ADULT RESPONDENT CONSENT FOR ADDITIONAL TESTING**				
253	ASK CONSENT FOR ADDITIONAL TESTING.	PROVIDE ADULT RESPONDENT WITH CONSENT FORM.		
254	CIRCLE THE CODE AND SIGN YOUR NAME.	GRANTED . . . . . 1<br>RESPONDENT REFUSED . . . 2<br>______<br>(SIGN AND SKIP TO 271)	GRANTED . . . . . 1<br>RESPONDENT REFUSED . . . 2<br>______<br>(SIGN AND SKIP TO 271)	GRANTED . . . . . 1<br>RESPONDENT REFUSED . . . 2<br>______<br>(SIGN AND SKIP TO 271)

ADULT RESP CONSENT

WEIGHT, HEIGHT, WAIST, BLOOD PRESSURE, HAEMOGLOBIN MEASUREMENT, BLOOD COLLECTION FOR HBA1C AND HIV TESTING, AND RECORDING OF MEDICINES FOR WOMEN AGE 15-95

		WOMAN 1	WOMAN 2	WOMAN 3
	NAME FROM LIST.	NAME ____________	NAME ____________	NAME ____________
**PARENTAL/RESPONSIBLE ADULT CONSENT FOR ANAEMIA TEST**				
255	ASK CONSENT FOR ANAEMIA TEST FROM PARENT/ADULT.	PROVIDE PARENT/RESPONSIBLE ADULT WITH CONSENT FORM.		
256	CIRCLE THE CODE AND SIGN YOUR NAME.	GRANTED . . . . . 1<br>PARENT/OTHER RESPONSIBLE ADULT REFUSED . . . . . 2<br>____________ (SIGN)<br>(IF REFUSED, SKIP TO 259)<br>NOT PRESENT/OTHER . . . 3 (SKIP TO 259)	GRANTED . . . . . 1<br>PARENT/OTHER RESPONSIBLE ADULT REFUSED . . . . . 2<br>____________ (SIGN)<br>(IF REFUSED, SKIP TO 259)<br>NOT PRESENT/OTHER . . . 3 (SKIP TO 259)	GRANTED . . . . . 1<br>PARENT/OTHER RESPONSIBLE ADULT REFUSED . . . . . 2<br>____________ (SIGN)<br>(IF REFUSED, SKIP TO 259)<br>NOT PRESENT/OTHER . . . 3 (SKIP TO 259)
**MINOR RESPONDENT CONSENT FOR ANAEMIA TEST**				
257	ASK CONSENT FOR ANAEMIA TEST FROM MINOR RESPONDENT.	PROVIDE MINOR RESPONDENT WITH CONSENT FORM.		
258	CIRCLE THE CODE AND SIGN YOUR NAME.	GRANTED . . . . . 1<br>MINOR RESPONDENT REFUSED . . . . . 2<br>____________ (SIGN)<br>(IF REFUSED, SKIP TO 259)<br>NOT PRESENT/OTHER . . . 3 (SKIP TO 259)	GRANTED . . . . . 1<br>MINOR RESPONDENT REFUSED . . . . . 2<br>____________ (SIGN)<br>(IF REFUSED, SKIP TO 259)<br>NOT PRESENT/OTHER . . . 3 (SKIP TO 259)	GRANTED . . . . . 1<br>MINOR RESPONDENT REFUSED . . . . . 2<br>____________ (SIGN)<br>(IF REFUSED, SKIP TO 259)<br>NOT PRESENT/OTHER . . . 3 (SKIP TO 259)
258A	Are you pregnant?	YES . . . . . 1<br>NO . . . . . 2<br>DON'T KNOW 8	YES . . . . . 1<br>NO . . . . . 2<br>DON'T KNOW 8	YES . . . . . 1<br>NO . . . . . 2<br>DON'T KNOW 8
**PARENTAL/RESPONSIBLE ADULT CONSENT FOR HBA1C TESTING**				
259	ASK CONSENT FOR HBA1C TESTING FROM PARENT/ADULT.	PROVIDE PARENT/RESPONSIBLE ADULT WITH CONSENT FORM.		
260	CIRCLE THE CODE AND SIGN YOUR NAME.	GRANTED . . . . . 1<br>PARENT/OTHER RESPONSIBLE ADULT REFUSED . . . . . 2<br>____________ (SIGN)<br>(IF REFUSED, SKIP TO 263)<br>NOT PRESENT/OTHER . . . 3 (SKIP TO 271)	GRANTED . . . . . 1<br>PARENT/OTHER RESPONSIBLE ADULT REFUSED . . . . . 2<br>____________ (SIGN)<br>(IF REFUSED, SKIP TO 263)<br>NOT PRESENT/OTHER . . . 3 (SKIP TO 271)	GRANTED . . . . . 1<br>PARENT/OTHER RESPONSIBLE ADULT REFUSED . . . . . 2<br>____________ (SIGN)<br>(IF REFUSED, SKIP TO 263)<br>NOT PRESENT/OTHER . . . 3 (SKIP TO 271)
**MINOR RESPONDENT CONSENT FOR HBA1C TESTING**				
261	ASK CONSENT FOR HBA1C TESTING FROM MINOR RESPONDENT.	PROVIDE MINOR RESPONDENT WITH CONSENT FORM.		
262	CIRCLE THE CODE, SIGN YOUR NAME, AND ENTER YOUR FIELDWORKER NUMBER.	GRANTED . . . . . 1<br>MINOR RESPONDENT REFUSED . . . . . 2<br>____________ (SIGN)<br>NOT PRESENT/OTHER . . . 3 (SKIP TO 271)	GRANTED . . . . . 1<br>MINOR RESPONDENT REFUSED . . . . . 2<br>____________ (SIGN)<br>NOT PRESENT/OTHER . . . 3 (SKIP TO 271)	GRANTED . . . . . 1<br>MINOR RESPONDENT REFUSED . . . . . 2<br>____________ (SIGN)<br>NOT PRESENT/OTHER . . . 3 (SKIP TO 271)

		WOMAN 1	WOMAN 2	WOMAN 3
	NAME FROM LIST.	NAME ________	NAME ________	NAME ________

PARENTAL/RESPONSIBLE ADULT CONSENT FOR HIV TESTING (PARENT—RESP ADULT CONSENT)

		WOMAN 1	WOMAN 2	WOMAN 3
263	ASK CONSENT FOR HIV TESTING FROM PARENT/ADULT.	PROVIDE PARENT/RESPONSIBLE ADULT WITH CONSENT FORM.		
264	CIRCLE THE CODE AND SIGN YOUR NAME.	GRANTED . . . . 1<br>PARENT/OTHER RESPONSIBLE ADULT REFUSED . . . . . . 2<br>________<br>(SIGN AND ENTER YOUR FIELDWORKER NUMBER)<br>[ ][ ][ ][ ]<br>(IF REFUSED, SKIP TO 271)	GRANTED . . . . 1<br>PARENT/OTHER RESPONSIBLE ADULT REFUSED . . . . . . 2<br>________<br>(SIGN AND ENTER YOUR FIELDWORKER NUMBER)<br>[ ][ ][ ][ ]<br>(IF REFUSED, SKIP TO 271)	GRANTED . . . . 1<br>PARENT/OTHER RESPONSIBLE ADULT REFUSED . . . . . . 2<br>________<br>(SIGN AND ENTER YOUR FIELDWORKER NUMBER)<br>[ ][ ][ ][ ]<br>(IF REFUSED, SKIP TO 271)

MINOR RESPONDENT CONSENT FOR HIV TESTING (MINOR RESP CONSENT)

		WOMAN 1	WOMAN 2	WOMAN 3
265	ASK CONSENT FOR HIV TESTING FROM MINOR RESPONDENT.	PROVIDE MINOR RESPONDENT WITH CONSENT FORM.		
266	CIRCLE THE CODE AND SIGN YOUR NAME.	GRANTED . . . . 1<br>MINOR RESPONDENT REFUSED . . . . 2<br>________<br>(SIGN)<br>(IF REFUSED, SKIP TO 271)	GRANTED . . . . 1<br>MINOR RESPONDENT REFUSED . . . . 2<br>________<br>(SIGN)<br>(IF REFUSED, SKIP TO 271)	GRANTED . . . . 1<br>MINOR RESPONDENT REFUSED . . . . 2<br>________<br>(SIGN)<br>(IF REFUSED, SKIP TO 271)

PARENTAL/RESPONSIBLE ADULT CONSENT FOR ADDITIONAL TESTING (PARENT—RESP ADULT CONSENT)

		WOMAN 1	WOMAN 2	WOMAN 3
267	ASK CONSENT FOR ADDITIONAL TESTING FROM PARENT/ADULT.	PROVIDE PARENT/RESPONSIBLE ADULT WITH CONSENT FORM.		
268	CIRCLE THE CODE AND SIGN YOUR NAME.	GRANTED . . . . 1<br>PARENT/OTHER RESPONSIBLE ADULT REFUSED . . . . . . 2<br>________<br>(SIGN)<br>(IF REFUSED, SKIP TO 271)	GRANTED . . . . 1<br>PARENT/OTHER RESPONSIBLE ADULT REFUSED . . . . . . 2<br>________<br>(SIGN)<br>(IF REFUSED, SKIP TO 271)	GRANTED . . . . 1<br>PARENT/OTHER RESPONSIBLE ADULT REFUSED . . . . . . 2<br>________<br>(SIGN)<br>(IF REFUSED, SKIP TO 271)

MINOR RESPONDENT CONSENT FOR ADDITIONAL TESTING (MINOR RESP CONSENT)

		WOMAN 1	WOMAN 2	WOMAN 3
269	ASK CONSENT FOR ADDITIONAL TESTING FROM MINOR RESPONDENT.	PROVIDE MINOR RESPONDENT WITH CONSENT FORM.		
270	CIRCLE THE CODE AND SIGN YOUR NAME.	GRANTED . . . . 1<br>MINOR RESPONDENT REFUSED . . . . 2<br>________<br>(SIGN)	GRANTED . . . . 1<br>MINOR RESPONDENT REFUSED . . . . 2<br>________<br>(SIGN)	GRANTED . . . . 1<br>MINOR RESPONDENT REFUSED . . . . 2<br>________<br>(SIGN)

		WOMAN 1	WOMAN 2	WOMAN 3
	NAME FROM LIST.	NAME ______	NAME ______	NAME ______
271	PREPARE EQUIPMENT AND SUPPLIES ONLY FOR THE TEST(S) FOR WHICH CONSENT HAS BEEN OBTAINED AND PROCEED WITH THE TEST(S).			
272	ADDITIONAL TESTS.	IF ADULT RESPONDENT, CHECK 254; IF MINOR RESPONDENT, CHECK 268 AND 270.<br>IF CONSENT HAS NOT BEEN GRANTED, WRITE "NO ADDITIONAL TESTS" ON THE FILTER PAPER.	IF ADULT RESPONDENT, CHECK 254; IF MINOR RESPONDENT, CHECK 268 AND 270.<br>IF CONSENT HAS NOT BEEN GRANTED, WRITE "NO ADDITIONAL TESTS" ON THE FILTER PAPER.	IF ADULT RESPONDENT, CHECK 254; IF MINOR RESPONDENT, CHECK 268 AND 270.<br>IF CONSENT HAS NOT BEEN GRANTED, WRITE "NO ADDITIONAL TESTS" ON THE FILTER PAPER.
273	RECORD HAEMOGLOBIN LEVEL HERE AND IN THE ADULT HEALTH INFORMATIONAL BROCHURE.	G/DL . . . . [ ][ ].[ ]<br>NOT PRESENT . . . . 994<br>REFUSED . . . . 995<br>OTHER 996	G/DL . . . . [ ][ ].[ ]<br>NOT PRESENT . . . . 994<br>REFUSED . . . . 995<br>OTHER 996	G/DL . . . . [ ][ ].[ ]<br>NOT PRESENT . . . . 994<br>REFUSED . . . . 995<br>OTHER 996
274	HBA1C TESTING: PLACE BAR CODE LABEL.	PUT THE 1ST BAR CODE LABEL HERE.<br>NOT PRESENT . . . . . . 99994<br>REFUSED . . . . 99995<br>OTHER 99996	PUT THE 1ST BAR CODE LABEL HERE.<br>NOT PRESENT . . . . . . 99994<br>REFUSED . . . . 99995<br>OTHER 99996	PUT THE 1ST BAR CODE LABEL HERE.<br>NOT PRESENT . . . . . . 99994<br>REFUSED . . . . 99995<br>OTHER 99996
275	HIV TESTING: PLACE BAR CODE LABEL.	PUT THE 2ND BAR CODE LABEL HERE.<br>NOT PRESENT . . . . . . 99994<br>REFUSED . . . . 99995<br>OTHER 99996	PUT THE 2ND BAR CODE LABEL HERE.<br>NOT PRESENT . . . . . . 99994<br>REFUSED . . . . 99995<br>OTHER 99996	PUT THE 2ND BAR CODE LABEL HERE.<br>NOT PRESENT . . . . . . 99994<br>REFUSED . . . . 99995<br>OTHER 99996
275A	OFFER HIV SELF-TEST KIT TO RESPONDENT WHO CONSENTED TO HIV TESTING.	TEST KIT ACCEPTED . . . . . 1<br>TEST KIT REFUSED . . . . . . 2<br>TEST KIT NOT OFFERRED 3<br>NOT PRESENT . . . . 4<br>OTHER 6	TEST KIT ACCEPTED . . . . . 1<br>TEST KIT REFUSED . . . . . . 2<br>TEST KIT NOT OFFERRED 3<br>NOT PRESENT . . . . 3<br>OTHER 6	TEST KIT ACCEPTED . . . . . 1<br>TEST KIT REFUSED . . . . . . 2<br>TEST KIT NOT OFFERRED 3<br>NOT PRESENT . . . . 3<br>OTHER 6
276	CHECK 274 AND 275: AT LEAST ONE BAR CODE LABEL PRESENT?	IF CONSENT GRANTED FOR EITHER TEST, PUT 3RD BAR CODE LABEL ON THE RESPONDENT'S FILTER PAPER CARD AND THE 4TH BAR CODE ON THE TRANSMITTAL FORM.	IF CONSENT GRANTED FOR EITHER TEST, PUT 3RD BAR CODE LABEL ON THE RESPONDENT'S FILTER PAPER CARD AND THE 4TH BAR CODE ON THE TRANSMITTAL FORM.	IF CONSENT GRANTED FOR EITHER TEST, PUT 3RD BAR CODE LABEL ON THE RESPONDENT'S FILTER PAPER CARD AND THE 4TH BAR CODE ON THE TRANSMITTAL FORM.

		WOMAN 1	WOMAN 2	WOMAN 3
	NAME FROM LIST.	NAME ______	NAME ______	NAME ______
277	Please show me all the prescribed medicines that you take regularly or daily.	MEDICINES SEEN ...... 1<br>NONE .... 2<br>NOT PRESENT .... 3<br>REFUSED .... 4<br>OTHER 6	MEDICINES SEEN ...... 1<br>NONE .... 2<br>NOT PRESENT .... 3<br>REFUSED .... 4<br>OTHER 6	MEDICINES SEEN ...... 1<br>NONE .... 2<br>NOT PRESENT .... 3<br>REFUSED .... 4<br>OTHER 6
		DRUG NAME ______	DRUG NAME ______	DRUG NAME ______
	RECORD ALL MEDICATION/DRUG NAMES.	DRUG NAME ______	DRUG NAME ______	DRUG NAME ______
		DRUG NAME ______	DRUG NAME ______	DRUG NAME ______
		DRUG NAME ______	DRUG NAME ______	DRUG NAME ______
		DRUG NAME ______	DRUG NAME ______	DRUG NAME ______
		DRUG NAME ______	DRUG NAME ______	DRUG NAME ______
		DRUG NAME ______	DRUG NAME ______	DRUG NAME ______
		DRUG NAME ______	DRUG NAME ______	DRUG NAME ______
		DRUG NAME ______	DRUG NAME ______	DRUG NAME ______
		DRUG NAME ______	DRUG NAME ______	DRUG NAME ______
		DRUG NAME ______	DRUG NAME ______	DRUG NAME ______
		DRUG NAME ______	DRUG NAME ______	DRUG NAME ______
278	GO BACK TO 202 IN NEXT COLUMN OF THIS QUESTIONNAIRE OR IN THE FIRST COLUMN OF AN ADDITIONAL QUESTIONNAIRE; IF NO MORE WOMEN, GO TO 301.			

WEIGHT, HEIGHT, WAIST, BLOOD PRESSURE, HAEMOGLOBIN MEASUREMENT, BLOOD COLLECTION FOR HBA1C AND HIV TESTING, AND RECORDING OF MEDICINES FOR MEN AGE 15-95

301	FROM THE LIST OF PERSONS ELIGIBLE FOR BIOMARKERS, RECORD THE LINE NUMBER, NAME, AGE, AND MARITAL STATUS FOR ALL ELIGIBLE MEN IN 302. WRITE THE NAME OF EACH MAN AT THE TOP OF THE FOLLOWING PAGES. IF THERE ARE MORE THAN THREE MEN, USE ADDITIONAL QUESTIONNAIRE(S).			
		MAN 1	MAN 2	MAN 3
302	CHECK LIST OF MEN ELIGIBLE FOR BIOMARKERS:<br><br>RECORD LINE NUMBER, NAME, AND AGE.<br><br>RECORD MARITAL STATUS.	LINE NUMBER . . . . [ ][ ]<br>NAME ________<br>AGE . . . . [ ][ ]<br>NEVER IN UNION . . . . 1<br>OTHER 2	LINE NUMBER . . . . [ ][ ]<br>NAME ________<br>AGE . . . . [ ][ ]<br>NEVER IN UNION . . . . 1<br>OTHER 2	LINE NUMBER . . . . [ ][ ]<br>NAME ________<br>AGE . . . . [ ][ ]<br>NEVER IN UNION . . . . 1<br>OTHER 2
302A	CHECK 302: AGE	15-17 YEARS . . . . 1<br>18-95 YEARS . . . . 2<br>(SKIP TO 302C)	15-17 YEARS . . . . 1<br>18-95 YEARS . . . . 2<br>(SKIP TO 302C)	15-17 YEARS . . . . 1<br>18-95 YEARS . . . . 2<br>(SKIP TO 302C)
302B	CHECK 302: MARITAL STATUS	NEVER IN UNION . . . . 1<br>(SKIP TO 302E)<br>OTHER 2	NEVER IN UNION . . . . 1<br>(SKIP TO 302E)<br>OTHER 2	NEVER IN UNION . . . . 1<br>(SKIP TO 302E)<br>OTHER 2

ADULT RESPONDENT CONSENT FOR ANTHROPOMETRY

(ADULT RESP CONSENT)

302C	ASK CONSENT FOR ANTHROPOMETRY.	PROVIDE ADULT RESPONDENT WITH CONSENT FORM.		
302D	CIRCLE THE CODE AND SIGN YOUR NAME.	GRANTED . . . . 1<br>RESPONDENT REFUSED . . . 2<br>________<br>(SIGN AND SKIP TO 305)<br>NOT PRESENT/OTHER . . . 3<br>(SKIP TO 305)	GRANTED . . . . 1<br>RESPONDENT REFUSED . . . 2<br>________<br>(SIGN AND SKIP TO 305)<br>NOT PRESENT/OTHER . . . 3<br>(SKIP TO 305)	GRANTED . . . . 1<br>RESPONDENT REFUSED . . . 2<br>________<br>(SIGN AND SKIP TO 305)<br>NOT PRESENT/OTHER . . . 3<br>(SKIP TO 305)

302E	RECORD NAME OF PARENT/ADULT RESPONSIBLE FOR MINOR.	NAME ________	NAME ________	NAME ________

PARENTAL/RESPONSIBLE ADULT CONSENT FOR ANTHROPOMETRY

(PARENT/RESP ADULT CONSENT)

302F	ASK CONSENT FOR ANTHROPOMETRY.	PROVIDE PARENT/RESPONSIBLE ADULT WITH PARENTAL CONSENT FORM.		
302G	CIRCLE THE CODE AND SIGN YOUR NAME.	GRANTED . . . . 1<br>PARENT/OTHER RESPONSIBLE ADULT REFUSED . . . . 2<br>________<br>(SIGN)<br>(IF REFUSED, SKIP TO 305)<br>NOT PRESENT/OTHER . . . 3<br>(SKIP TO 305)	GRANTED . . . . 1<br>PARENT/OTHER RESPONSIBLE ADULT REFUSED . . . . 2<br>________<br>(SIGN)<br>(IF REFUSED, SKIP TO 305)<br>NOT PRESENT/OTHER . . . 3<br>(SKIP TO 305)	GRANTED . . . . 1<br>PARENT/OTHER RESPONSIBLE ADULT REFUSED . . . . 2<br>________<br>(SIGN)<br>(IF REFUSED, SKIP TO 305)<br>NOT PRESENT/OTHER . . . 3<br>(SKIP TO 305)

MINOR RESPONDENT CONSENT FOR ANTHROPOMETRY

(MINOR RESP CONSENT)

302H	ASK CONSENT FOR ANTHROPOMETRY.	PROVIDE MINOR RESPONDENT WITH CONSENT FORM.		
302I	CIRCLE THE CODE AND SIGN YOUR NAME.	GRANTED . . . . 1<br>MINOR RESPONDENT REFUSED . . . . 2<br>________<br>(SIGN)<br>NOT PRESENT/OTHER . . . 3	GRANTED . . . . 1<br>MINOR RESPONDENT REFUSED . . . . 2<br>________<br>(SIGN)<br>NOT PRESENT/OTHER . . . 3	GRANTED . . . . 1<br>MINOR RESPONDENT REFUSED . . . . 2<br>________<br>(SIGN)<br>NOT PRESENT/OTHER . . . 3

		MAN 1	MAN 2	MAN 3
	NAME FROM LIST.	NAME ______	NAME ______	NAME ______
305	WEIGHT IN KILOGRAMS.	KG. . . . [ ][ ][ ].[ ][0]<br>NOT PRESENT . . . . . . 99994<br>REFUSED . . . . 99995<br>OTHER 99996	KG. . . . [ ][ ][ ].[ ][0]<br>NOT PRESENT . . . . . . 99994<br>REFUSED . . . . 99995<br>OTHER 99996	KG. . . . [ ][ ][ ].[ ][0]<br>NOT PRESENT . . . . . . 99994<br>REFUSED . . . . 99995<br>OTHER 99996
306	HEIGHT IN CENTIMETRES.	CM. . . . . . . [ ][ ][ ].[ ]<br>NOT PRESENT . . . . 9994<br>REFUSED . . . . 9995<br>OTHER 9996	CM. . . . . . . [ ][ ][ ].[ ]<br>NOT PRESENT . . . . 9994<br>REFUSED . . . . 9995<br>OTHER 9996	CM. . . . . . . [ ][ ][ ].[ ]<br>NOT PRESENT . . . . 9994<br>REFUSED . . . . 9995<br>OTHER 9996
306A	WAIST CIRCUMFERENCE IN CENTIMETRES.	CM. . . . . . . [ ][ ][ ].[ ]<br>NOT PRESENT . . . . 9994<br>REFUSED . . . . 9995<br>OTHER 9996	CM. . . . . . . [ ][ ][ ].[ ]<br>NOT PRESENT . . . . 9994<br>REFUSED . . . . 9995<br>OTHER 9996	CM. . . . . . . [ ][ ][ ].[ ]<br>NOT PRESENT . . . . 9994<br>REFUSED . . . . 9995<br>OTHER 9996
307	MEASURER: ENTER YOUR FIELDWORKER NUMBER.	[ ][ ][ ][ ]<br>FIELDWORKER NUMBER	[ ][ ][ ][ ]<br>FIELDWORKER NUMBER	[ ][ ][ ][ ]<br>FIELDWORKER NUMBER
308	CHECK 302: AGE	15-17 YEARS . . . . 1<br>18-95 YEARS . . . . 2<br>(SKIP TO 310)	15-17 YEARS . . . . 1<br>18-95 YEARS . . . . 2<br>(SKIP TO 310)	15-17 YEARS . . . . 1<br>18-95 YEARS . . . . 2<br>(SKIP TO 310)
309	CHECK 302: MARITAL STATUS	NEVER IN UNION . . . . 1<br>(SKIP TO 313)<br>OTHER 2	NEVER IN UNION . . . . 1<br>(SKIP TO 313)<br>OTHER 2	NEVER IN UNION . . . . 1<br>(SKIP TO 313)<br>OTHER 2

WEIGHT, HEIGHT, WAIST, BLOOD PRESSURE, HAEMOGLOBIN MEASUREMENT, BLOOD COLLECTION FOR HBA1C AND HIV TESTING, AND RECORDING OF MEDICINES FOR MEN AGE 15-95

			MAN 1	MAN 2	MAN 3
		NAME FROM LIST.	NAME ____________	NAME ____________	NAME ____________
ADULT RESPONDENT CONSENT		**ADULT RESPONDENT CONSENT FOR BLOOD PRESSURE MEASUREMENT**			
	310	ASK CONSENT FOR BLOOD PRESSURE MEASUREMENT.	PROVIDE ADULT RESPONDENT WITH CONSENT FORM.		
	311	CIRCLE THE CODE AND SIGN YOUR NAME.	GRANTED . . . . 1<br>RESPONDENT REFUSED . . . 2<br>____________<br>(SIGN)<br>(IF GRANTED, SKIP TO 317; IF REFUSED, SKIP TO 347)<br>NOT PRESENT/OTHER . . . 3<br>(SKIP TO 347)	GRANTED . . . . 1<br>RESPONDENT REFUSED . . . 2<br>____________<br>(SIGN)<br>(IF GRANTED, SKIP TO 317; IF REFUSED, SKIP TO 347)<br>NOT PRESENT/OTHER . . . 3<br>(SKIP TO 347)	GRANTED . . . . 1<br>RESPONDENT REFUSED . . . 2<br>____________<br>(SIGN)<br>(IF GRANTED, SKIP TO 317; IF REFUSED, SKIP TO 347)<br>NOT PRESENT/OTHER . . . 3<br>(SKIP TO 347)

PARENT—RESP ADULT CONSENT		**PARENTAL/RESPONSIBLE ADULT CONSENT FOR BLOOD PRESSURE MEASUREMENT**			
	313	ASK CONSENT FOR BLOOD PRESSURE MEASUREMENT.	PROVIDE PARENT/RESPONSIBLE ADULT WITH PARENTAL CONSENT FORM.		
	314	CIRCLE THE CODE AND SIGN YOUR NAME.	GRANTED . . . . 1<br>PARENT/OTHER RESPONSIBLE ADULT REFUSED . . . . 2<br>____________<br>(SIGN)<br>(IF REFUSED, SKIP TO 355)<br>NOT PRESENT/OTHER . . . 3<br>(SKIP TO 355)	GRANTED . . . . 1<br>PARENT/OTHER RESPONSIBLE ADULT REFUSED . . . . 2<br>____________<br>(SIGN)<br>(IF REFUSED, SKIP TO 355)<br>NOT PRESENT/OTHER . . . 3<br>(SKIP TO 355)	GRANTED . . . . 1<br>PARENT/OTHER RESPONSIBLE ADULT REFUSED . . . . 2<br>____________<br>(SIGN)<br>(IF REFUSED, SKIP TO 355)<br>NOT PRESENT/OTHER . . . 3<br>(SKIP TO 355)

MINOR RESPONDENT CONSENT		**MINOR RESPONDENT CONSENT FOR BLOOD PRESSURE MEASUREMENT**			
	315	ASK CONSENT FOR BLOOD PRESSURE MEASUREMENT.	PROVIDE MINOR RESPONDENT WITH CONSENT FORM.		
	316	CIRCLE THE CODE AND SIGN YOUR NAME.	GRANTED . . . . 1<br>MINOR RESPONDENT REFUSED . . . . 2<br>____________<br>(SIGN)<br>(IF REFUSED, SKIP TO 355)<br>NOT PRESENT . . . . 3<br>(SKIP TO 355)	GRANTED . . . . 1<br>MINOR RESPONDENT REFUSED . . . . 2<br>____________<br>(SIGN)<br>(IF REFUSED, SKIP TO 355)<br>NOT PRESENT . . . . 3<br>(SKIP TO 355)	GRANTED . . . . 1<br>MINOR RESPONDENT REFUSED . . . . 2<br>____________<br>(SIGN)<br>(IF REFUSED, SKIP TO 355)<br>NOT PRESENT . . . . 3<br>(SKIP TO 355)

		MAN 1	MAN 2	MAN 3
	NAME FROM LIST.	NAME ________	NAME ________	NAME ________
317	Before taking your blood pressure, I would like to ask a few questions about things that may affect these measurements. Have you done any of the following within the past 30 minutes:	YES NO	YES NO	YES NO
a)	Eaten anything?	EATEN 1 2	EATEN 1 2	EATEN 1 2
b)	Had coffee, tea, cola or other drink that has caffeine?	HAD CAFFEINATED DRINK 1 2	HAD CAFFEINATED DRINK 1 2	HAD CAFFEINATED DRINK 1 2
c)	Smoked any tobacco product?	SMOKED 1 2	SMOKED 1 2	SMOKED 1 2
d)	Used any other type of tobacco such as chewing tobacco or snuff?	OTHER TOBACCO 1 2	OTHER TOBACCO 1 2	OTHER TOBACCO 1 2
318	May I begin the process of measuring your blood pressure? I will begin by measuring the circumference of your arm to make sure that I use the right equipment.	MEASURE THE CIRCUMFERENCE OF THE RESPONDENT'S ARM MIDWAY BETWEEN THE ELBOW AND THE SHOULDER. RECORD THE MEASUREMENT IN CENTIMETRES.<br>ARM CIRCUMFERENCE (IN CENTIMETRES). ☐☐	MEASURE THE CIRCUMFERENCE OF THE RESPONDENT'S ARM MIDWAY BETWEEN THE ELBOW AND THE SHOULDER. RECORD THE MEASUREMENT IN CENTIMETRES.<br>ARM CIRCUMFERENCE (IN CENTIMETRES). ☐☐	MEASURE THE CIRCUMFERENCE OF THE RESPONDENT'S ARM MIDWAY BETWEEN THE ELBOW AND THE SHOULDER. RECORD THE MEASUREMENT IN CENTIMETRES.<br>ARM CIRCUMFERENCE (IN CENTIMETRES). ☐☐
319	USE THE ARM CIRCUMFERENCE MEASUREMENT TO SELECT THE APPROPRIATE BLOOD PRESSURE MONITOR CUFF SIZE. CIRCLE THE CODE FOR THE CUFF SIZE.	SMALL: 17 CM – 22 CM . . . . . 1<br>MEDIUM: 23 CM – 31 CM . . . . . 2<br>LARGE: 32 CM – 42 CM . . . . . 3<br>EXTRA LARGE: ≥43 CM 4	SMALL: 17 CM – 22 CM . . . . 1<br>MEDIUM: 23 CM – 31 CM . . . . 2<br>LARGE: 32 CM – 42 CM . . . . 3<br>EXTRA LARGE: ≥43 CM 4	SMALL: 17 CM – 22 CM . . . . 1<br>MEDIUM: 23 CM – 31 CM . . . . 2<br>LARGE: 32 CM – 42 CM . . . . 3<br>EXTRA LARGE: ≥43 CM 4
320	RECORD TIME OF FIRST BP READING	HOURS MINUTES<br>☐☐ : ☐☐	HOURS MINUTES<br>☐☐ : ☐☐	HOURS MINUTES<br>☐☐ : ☐☐
321	TAKE THE FIRST BLOOD PRESSURE READING. RECORD THE SYSTOLIC AND DIASTOLIC PRESSURE AND PULSE (HEART RATE).	**FIRST BP MEASURE**<br>SYSTOLIC. . . . . ☐☐☐<br>DIASTOLIC . . . . . . ☐☐☐<br>PULSE . . . . ☐☐☐<br>TECHNICAL PROBLEMS . 994<br>REFUSED . . . . 995<br>OTHER . . . . 996<br>(IF NOT MEASURED, GO TO 345)	**FIRST BP MEASURE**<br>SYSTOLIC. . . . . ☐☐☐<br>DIASTOLIC . . . . . . ☐☐☐<br>PULSE . . . . ☐☐☐<br>TECHNICAL PROBLEMS . 994<br>REFUSED . . . . 995<br>OTHER . . . . 996<br>(IF NOT MEASURED, GO TO 345)	**FIRST BP MEASURE**<br>SYSTOLIC. . . . . ☐☐☐<br>DIASTOLIC . . . . . . ☐☐☐<br>PULSE . . . . ☐☐☐<br>TECHNICAL PROBLEMS . 994<br>REFUSED . . . . 995<br>OTHER . . . . 996<br>(IF NOT MEASURED, GO TO 345)

		MAN 1	MAN 2	MAN 3
	NAME FROM LIST.	NAME ____________	NAME ____________	NAME ____________
322	Before this survey, has your blood pressure ever been checked?	YES . . . . . 1<br>NO 2	YES . . . . . 1<br>NO 2	YES . . . . . 1<br>NO 2
323	Were you told on two or more different occasions by a doctor or other health professional that you had hypertension or high blood pressure?	YES . . . . . 1<br>NO 2	YES . . . . . 1<br>NO 2	YES . . . . . 1<br>NO 2
324	To lower your blood pressure, are you now taking a prescribed medicine?	YES . . . . . 1<br>NO 2	YES . . . . . 1<br>NO 2	YES . . . . . 1<br>NO 2
325	**CHECK THAT IT HAS BEEN AT LEAST 3 MINUTES BEFORE TAKING THE SECOND BLOOD PRESSURE MEASUREMENT**			
326	May I take your blood pressure at this time?	YES . . . . . 1<br>NO . . . . . 2<br>(GO TO 343)	YES . . . . . 1<br>NO . . . . . 2<br>(GO TO 343)	YES . . . . . 1<br>NO . . . . . 2<br>(GO TO 343)
327	RECORD TIME OF SECOND BP READING.	HOURS MINUTES<br>[ ][ ] : [ ][ ]	HOURS MINUTES<br>[ ][ ] : [ ][ ]	HOURS MINUTES<br>[ ][ ] : [ ][ ]
328	TAKE THE SECOND BLOOD PRESSURE READING. RECORD THE SYSTOLIC AND DIASTOLIC PRESSURE AND PULSE (HEART RATE).	**SECOND BP MEASURE**<br>SYSTOLIC . . . . . [ ][ ][ ]<br>DIASTOLIC . . . . . [ ][ ][ ]<br>PULSE . . . . . [ ][ ][ ]<br>TECHNICAL PROBLEMS . 994<br>REFUSED . . . . . 995<br>OTHER . . . . . 996<br>(IF NOT MEASURED, GO TO 343)	**SECOND BP MEASURE**<br>SYSTOLIC . . . . . [ ][ ][ ]<br>DIASTOLIC . . . . . [ ][ ][ ]<br>PULSE . . . . . [ ][ ][ ]<br>TECHNICAL PROBLEMS . 994<br>REFUSED . . . . . 995<br>OTHER . . . . . 996<br>(IF NOT MEASURED, GO TO 343)	**SECOND BP MEASURE**<br>SYSTOLIC . . . . . [ ][ ][ ]<br>DIASTOLIC . . . . . [ ][ ][ ]<br>PULSE . . . . . [ ][ ][ ]<br>TECHNICAL PROBLEMS . 994<br>REFUSED . . . . . 995<br>OTHER . . . . . 996<br>(IF NOT MEASURED, GO TO 343)

		MAN 1	MAN 2	MAN 3
	NAME FROM LIST.	NAME ______	NAME ______	NAME ______
329	**CHECK THAT IT HAS BEEN AT LEAST 3 MINUTES BEFORE TAKING THE THIRD BLOOD PRESSURE MEASUREMENT**			
330	May I take your blood pressure at this time?	YES . . . 1<br>NO . . . 2 (GO TO 343)	YES . . . 1<br>NO . . . 2 (GO TO 343)	YES . . . 1<br>NO . . . 2 (GO TO 343)
331	RECORD TIME OF THIRD BP READING	HOURS [ ][ ] : MINUTES [ ][ ]	HOURS [ ][ ] : MINUTES [ ][ ]	HOURS [ ][ ] : MINUTES [ ][ ]
332	TAKE THE THIRD BLOOD PRESSURE READING. RECORD THE SYSTOLIC AND DIASTOLIC PRESSURE AND PULSE (HEART RATE).	**THIRD BP MEASURE**<br>SYSTOLIC . . . [ ][ ][ ]<br>DIASTOLIC . . . [ ][ ][ ]<br>PULSE . . . [ ][ ][ ]<br>TECHNICAL PROBLEMS . 994<br>REFUSED . . . 995<br>OTHER . . . 996	**THIRD BP MEASURE**<br>SYSTOLIC . . . [ ][ ][ ]<br>DIASTOLIC . . . [ ][ ][ ]<br>PULSE . . . [ ][ ][ ]<br>TECHNICAL PROBLEMS . 994<br>REFUSED . . . 995<br>OTHER . . . 996	**THIRD BP MEASURE**<br>SYSTOLIC . . . [ ][ ][ ]<br>DIASTOLIC . . . [ ][ ][ ]<br>PULSE . . . [ ][ ][ ]<br>TECHNICAL PROBLEMS . 994<br>REFUSED . . . 995<br>OTHER . . . 996

343 CIRCLE THE SINGLE NUMBER WHERE THE FINAL READING OF THE DIASTOLIC AND SYSTOLIC MEASURES MEET.

**FINAL SYSTOLIC**	MAN 1 **FINAL DIASTOLIC** <80	<85	85-89	90-99	100-109	≥110	MAN 2 **FINAL DIASTOLIC** <80	<85	85-89	90-99	100-109	≥110	MAN 3 **FINAL DIASTOLIC** <80	<85	85-89	90-99	100-109	≥110
<120	1	2	3	4	5	6	1	2	3	4	5	6	1	2	3	4	5	6
<130	2	2	3	4	5	6	2	2	3	4	5	6	2	2	3	4	5	6
130-139	3	3	3	4	5	6	3	3	3	4	5	6	3	3	3	4	5	6
140-159	4	4	4	4	5	6	4	4	4	4	5	6	4	4	4	4	5	6
160-179	5	5	5	5	5	6	5	5	5	5	5	6	5	5	5	5	5	6
≥180	6	6	6	6	6	6	6	6	6	6	6	6	6	6	6	6	6	6

344 LOCATE THE NUMBER YOU CIRCLED IN 343 IN THE CHART BELOW. THEN USE THE INSTRUCTIONS TO THE RIGHT OF THAT NUMBER TO COMPLETE A BLOOD PRESSURE REPORT AND REFERRAL FORM FOR THE RESPONDENT. GIVE THE FORM TO THE RESPONDENT AND ANSWER ANY QUESTIONS.

**NUMBER CIRCLED IN 343**	**RESPONDENT'S BLOOD PRESSURE CATEGORY**	**CONSULT HEALTH PROVIDER TO CHECK BLOOD PRESSURE WITHIN:**
**1**	**NORMAL (OPTIMAL)**	**1 YEAR**
**2**	**NORMAL (MILDLY HIGH)**	**1 YEAR**
**3**	**NORMAL (MODERATELY HIGH)**	**2 MONTHS**
**4**	**ABNORMAL (MILDLY ELEVATED)**	**1 MONTH**
**5**	**ABNORMAL (MODERATELY ELEVATED)**	**1 DAY/IMMEDIATELY**
**6**	**ABNORMAL (SEVERELY ELEVATED)**	**IMMEDIATELY**

		MAN 1	MAN 2	MAN 3
345	CHECK 302: AGE	15-17 YEARS . . . 1<br>18-95 YEARS . . . 2 (SKIP TO 347)	15-17 YEARS . . . 1<br>18-95 YEARS . . . 2 (SKIP TO 347)	15-17 YEARS . . . 1<br>18-95 YEARS . . . 2 (SKIP TO 347)
346	CHECK 302: MARITAL STATUS	NEVER IN UNION . . . 1 (SKIP TO 355)<br>OTHER . . . 2	NEVER IN UNION . . . 1 (SKIP TO 355)<br>OTHER . . . 2	NEVER IN UNION . . . 1 (SKIP TO 355)<br>OTHER . . . 2

WEIGHT, HEIGHT, WAIST, BLOOD PRESSURE, HAEMOGLOBIN MEASUREMENT, BLOOD COLLECTION FOR HBA1C AND HIV TESTING, AND RECORDING OF MEDICINES FOR MEN AGE 15-95

		MAN 1	MAN 2	MAN 3
	NAME FROM LIST.	NAME ______	NAME ______	NAME ______

ADULT RESPONDENT CONSENT FOR ANAEMIA TEST

ADULT RESP CONSENT

347	ASK CONSENT FOR ANAEMIA TEST.	PROVIDE ADULT RESPONDENT WITH CONSENT FORM.		
348	CIRCLE THE CODE AND SIGN YOUR NAME.	GRANTED . . . . 1<br>RESPONDENT REFUSED . . . 2<br>______<br>(SIGN)<br>(IF REFUSED, SKIP TO 349)<br>NOT PRESENT/OTHER . . . 3	GRANTED . . . . 1<br>RESPONDENT REFUSED . . . 2<br>______<br>(SIGN)<br>(IF REFUSED, SKIP TO 349)<br>NOT PRESENT/OTHER . . . 3	GRANTED . . . . 1<br>RESPONDENT REFUSED . . . 2<br>______<br>(SIGN)<br>(IF REFUSED, SKIP TO 349)<br>NOT PRESENT/OTHER . . . 3

ADULT RESPONDENT CONSENT FOR HBA1C TESTING

ADULT RESP CONSENT

349	ASK CONSENT FOR HBA1C TESTING.	PROVIDE ADULT RESPONDENT WITH CONSENT FORM.		
350	CIRCLE THE CODE, SIGN YOUR NAME, AND ENTER YOUR FIELDWORKER NUMBER.	GRANTED . . . . 1<br>RESPONDENT REFUSED . . . 2<br>______<br>SIGN<br>NOT PRESENT/OTHER . . . 3<br>(SKIP TO 371)	GRANTED . . . . 1<br>RESPONDENT REFUSED . . . 2<br>______<br>SIGN<br>NOT PRESENT/OTHER . . . 3<br>(SKIP TO 371)	GRANTED . . . . 1<br>RESPONDENT REFUSED . . . 2<br>______<br>SIGN<br>NOT PRESENT/OTHER . . . 3<br>(SKIP TO 371)

ADULT RESPONDENT CONSENT FOR HIV TESTING

ADULT RESP CONSENT

351	ASK CONSENT FOR HIV TESTING.	PROVIDE ADULT RESPONDENT WITH CONSENT FORM.		
352	CIRCLE THE CODE AND SIGN YOUR NAME.	GRANTED . . . . 1<br>RESPONDENT REFUSED . . . 2<br>______<br>(SIGN AND ENTER YOUR FIELDWORKER NUMBER)<br>[ ][ ][ ][ ]<br>(IF REFUSED, SKIP TO 371)	GRANTED . . . . 1<br>RESPONDENT REFUSED . . . 2<br>______<br>(SIGN AND ENTER YOUR FIELDWORKER NUMBER)<br>[ ][ ][ ][ ]<br>(IF REFUSED, SKIP TO 371)	GRANTED . . . . 1<br>RESPONDENT REFUSED . . . 2<br>______<br>(SIGN AND ENTER YOUR FIELDWORKER NUMBER)<br>[ ][ ][ ][ ]<br>(IF REFUSED, SKIP TO 371)

ADULT RESPONDENT CONSENT FOR ADDITIONAL TESTING

ADULT RESP CONSENT

353	ASK CONSENT FOR ADDITIONAL TESTING.	PROVIDE ADULT RESPONDENT WITH CONSENT FORM.		
354	CIRCLE THE CODE AND SIGN YOUR NAME.	GRANTED . . . . 1<br>RESPONDENT REFUSED . . . 2<br>______<br>(SIGN AND SKIP TO 371)	GRANTED . . . . 1<br>RESPONDENT REFUSED . . . 2<br>______<br>(SIGN AND SKIP TO 371)	GRANTED . . . . 1<br>RESPONDENT REFUSED . . . 2<br>______<br>(SIGN AND SKIP TO 371)

		MAN 1	MAN 2	MAN 3
	NAME FROM LIST.	NAME ________	NAME ________	NAME ________
	**PARENTAL/RESPONSIBLE ADULT CONSENT FOR ANAEMIA TEST**			
355	ASK CONSENT FOR ANAEMIA TEST FROM PARENT/ADULT.	PROVIDE PARENT/RESPONSIBLE ADULT WITH CONSENT FORM.		
356	CIRCLE THE CODE AND SIGN YOUR NAME.	GRANTED . . . . . . 1<br>PARENT/OTHER RESPONSIBLE ADULT REFUSED . . . . . . 2<br>________ (SIGN)<br>(IF REFUSED, SKIP TO 359)<br>NOT PRESENT/OTHER . . . 3 (SKIP TO 359)	GRANTED . . . . . . 1<br>PARENT/OTHER RESPONSIBLE ADULT REFUSED . . . . . . 2<br>________ (SIGN)<br>(IF REFUSED, SKIP TO 359)<br>NOT PRESENT/OTHER . . . 3 (SKIP TO 359)	GRANTED . . . . . . 1<br>PARENT/OTHER RESPONSIBLE ADULT REFUSED . . . . . . 2<br>________ (SIGN)<br>(IF REFUSED, SKIP TO 359)<br>NOT PRESENT/OTHER . . . 3 (SKIP TO 359)
	**MINOR RESPONDENT CONSENT FOR ANAEMIA TEST**			
357	ASK CONSENT FOR ANAEMIA TEST FROM MINOR RESPONDENT.	PROVIDE MINOR RESPONDENT WITH CONSENT FORM.		
358	CIRCLE THE CODE AND SIGN YOUR NAME.	GRANTED . . . . . . 1<br>MINOR RESPONDENT REFUSED . . . . . . 2<br>________ (SIGN)<br>(IF REFUSED, SKIP TO 359)<br>NOT PRESENT/OTHER . . . 3	GRANTED . . . . . . 1<br>MINOR RESPONDENT REFUSED . . . . . . 2<br>________ (SIGN)<br>(IF REFUSED, SKIP TO 359)<br>NOT PRESENT/OTHER . . . 3	GRANTED . . . . . . 1<br>MINOR RESPONDENT REFUSED . . . . . . 2<br>________ (SIGN)<br>(IF REFUSED, SKIP TO 359)<br>NOT PRESENT/OTHER . . . 3
	**PARENTAL/RESPONSIBLE ADULT CONSENT FOR HBA1C TESTING**			
359	ASK CONSENT FOR HBA1C TESTING FROM PARENT/ADULT.	PROVIDE PARENT/RESPONSIBLE ADULT WITH CONSENT FORM.		
360	CIRCLE THE CODE AND SIGN YOUR NAME.	GRANTED . . . . . . 1<br>PARENT/OTHER RESPONSIBLE ADULT REFUSED . . . . . . 2<br>________ (SIGN)<br>(IF REFUSED, SKIP TO 363)<br>NOT PRESENT/OTHER . . . 3 (SKIP TO 371)	GRANTED . . . . . . 1<br>PARENT/OTHER RESPONSIBLE ADULT REFUSED . . . . . . 2<br>________ (SIGN)<br>(IF REFUSED, SKIP TO 363)<br>NOT PRESENT/OTHER . . . 3 (SKIP TO 371)	GRANTED . . . . . . 1<br>PARENT/OTHER RESPONSIBLE ADULT REFUSED . . . . . . 2<br>________ (SIGN)<br>(IF REFUSED, SKIP TO 363)<br>NOT PRESENT/OTHER . . . 3 (SKIP TO 371)
	**MINOR RESPONDENT CONSENT FOR HBA1C TESTING**			
361	ASK CONSENT FOR HBA1C TESTING FROM MINOR RESPONDENT.	PROVIDE MINOR RESPONDENT WITH CONSENT FORM.		
362	CIRCLE THE CODE, SIGN YOUR NAME, AND ENTER YOUR FIELDWORKER NUMBER.	GRANTED . . . . . . 1<br>MINOR RESPONDENT REFUSED . . . . . . 2<br>________ (SIGN)<br>NOT PRESENT/OTHER . . . 3 (SKIP TO 371)	GRANTED . . . . . . 1<br>MINOR RESPONDENT REFUSED . . . . . . 2<br>________ (SIGN)<br>NOT PRESENT/OTHER . . . 3 (SKIP TO 371)	GRANTED . . . . . . 1<br>MINOR RESPONDENT REFUSED . . . . . . 2<br>________ (SIGN)<br>NOT PRESENT/OTHER . . . 3 (SKIP TO 371)

Side labels: PARENT/RESP ADULT CONSENT; MINOR RESP CONSENT; PARENT/RESP ADULT CONSENT; MINOR RESP CONSENT

		MAN 1	MAN 2	MAN 3
	NAME FROM LIST.	NAME ____________	NAME ____________	NAME ____________

PARENTAL/RESPONSIBLE ADULT CONSENT FOR HIV TESTING (PARENT/RESP ADULT CONSENT)

		MAN 1	MAN 2	MAN 3
363	ASK CONSENT FOR HIV TESTING FROM PARENT/ADULT.	PROVIDE PARENT/RESPONSIBLE ADULT WITH CONSENT FORM.		
364	CIRCLE THE CODE AND SIGN YOUR NAME.	GRANTED . . . . 1<br>PARENT/OTHER RESPONSIBLE ADULT REFUSED . . . . . . 2<br>____________<br>(SIGN AND ENTER YOUR FIELDWORKER NUMBER)<br>[ ][ ][ ][ ]<br>(IF REFUSED, SKIP TO 371)	GRANTED . . . . 1<br>PARENT/OTHER RESPONSIBLE ADULT REFUSED . . . . . . 2<br>____________<br>(SIGN AND ENTER YOUR FIELDWORKER NUMBER)<br>[ ][ ][ ][ ]<br>(IF REFUSED, SKIP TO 371)	GRANTED . . . . 1<br>PARENT/OTHER RESPONSIBLE ADULT REFUSED . . . . . . 2<br>____________<br>(SIGN AND ENTER YOUR FIELDWORKER NUMBER)<br>[ ][ ][ ][ ]<br>(IF REFUSED, SKIP TO 371)

MINOR RESPONDENT CONSENT FOR HIV TESTING (MINOR RESP CONSENT)

		MAN 1	MAN 2	MAN 3
365	ASK CONSENT FOR HIV TESTING FROM MINOR RESPONDENT.	PROVIDE MINOR RESPONDENT WITH CONSENT FORM.		
366	CIRCLE THE CODE AND SIGN YOUR NAME.	GRANTED . . . . 1<br>MINOR RESPONDENT REFUSED . . . . 2<br>____________<br>(SIGN)<br>(IF REFUSED, SKIP TO 371)	GRANTED . . . . 1<br>MINOR RESPONDENT REFUSED . . . . 2<br>____________<br>(SIGN)<br>(IF REFUSED, SKIP TO 371)	GRANTED . . . . 1<br>MINOR RESPONDENT REFUSED . . . . 2<br>____________<br>(SIGN)<br>(IF REFUSED, SKIP TO 371)

PARENTAL/RESPONSIBLE ADULT CONSENT FOR ADDITIONAL TESTING (PARENT/RESP ADULT CONSENT)

		MAN 1	MAN 2	MAN 3
367	ASK CONSENT FOR ADDITIONAL TESTING FROM PARENT/ADULT.	PROVIDE PARENT/RESPONSIBLE ADULT WITH CONSENT FORM.		
368	CIRCLE THE CODE AND SIGN YOUR NAME.	GRANTED . . . . 1<br>PARENT/OTHER RESPONSIBLE ADULT REFUSED . . . . . . 2<br>____________<br>(SIGN)<br>(IF REFUSED, SKIP TO 371)	GRANTED . . . . 1<br>PARENT/OTHER RESPONSIBLE ADULT REFUSED . . . . . . 2<br>____________<br>(SIGN)<br>(IF REFUSED, SKIP TO 371)	GRANTED . . . . 1<br>PARENT/OTHER RESPONSIBLE ADULT REFUSED . . . . . . 2<br>____________<br>(SIGN)<br>(IF REFUSED, SKIP TO 371)

MINOR RESPONDENT CONSENT FOR ADDITIONAL TESTING (MINOR RESP CONSENT)

		MAN 1	MAN 2	MAN 3
369	ASK CONSENT FOR ADDITIONAL TESTING FROM MINOR RESPONDENT.	PROVIDE MINOR RESPONDENT WITH CONSENT FORM.		
370	CIRCLE THE CODE AND SIGN YOUR NAME.	GRANTED . . . . 1<br>MINOR RESPONDENT REFUSED . . . . 2<br>____________<br>(SIGN)	GRANTED . . . . 1<br>MINOR RESPONDENT REFUSED . . . . 2<br>____________<br>(SIGN)	GRANTED . . . . 1<br>MINOR RESPONDENT REFUSED . . . . 2<br>____________<br>(SIGN)

		MAN 1	MAN 2	MAN 3
	NAME FROM LIST.	NAME ______	NAME ______	NAME ______
371	PREPARE EQUIPMENT AND SUPPLIES ONLY FOR THE TEST(S) FOR WHICH CONSENT HAS BEEN OBTAINED AND PROCEED WITH THE TEST(S).			
372	ADDITIONAL TESTS.	IF ADULT RESPONDENT, CHECK 354; IF MINOR RESPONDENT, CHECK 368 AND 370.<br>IF CONSENT HAS NOT BEEN GRANTED, WRITE "NO ADDITIONAL TESTS" ON THE FILTER PAPER.	IF ADULT RESPONDENT, CHECK 354; IF MINOR RESPONDENT, CHECK 368 AND 370.<br>IF CONSENT HAS NOT BEEN GRANTED, WRITE "NO ADDITIONAL TESTS" ON THE FILTER PAPER.	IF ADULT RESPONDENT, CHECK 354; IF MINOR RESPONDENT, CHECK 368 AND 370.<br>IF CONSENT HAS NOT BEEN GRANTED, WRITE "NO ADDITIONAL TESTS" ON THE FILTER PAPER.
373	RECORD HAEMOGLOBIN LEVEL HERE AND IN THE ADULT HEALTH INFORMATIONAL BROCHURE.	G/DL . . . . ☐☐.☐<br>NOT PRESENT . . . . 994<br>REFUSED . . . . 995<br>OTHER 996	G/DL . . . . ☐☐.☐<br>NOT PRESENT . . . . 994<br>REFUSED . . . . 995<br>OTHER 996	G/DL . . . . ☐☐.☐<br>NOT PRESENT . . . . 994<br>REFUSED . . . . 995<br>OTHER 996
374	HBA1C TESTING: PLACE BAR CODE LABEL.	PUT THE 1ST BAR CODE LABEL HERE.<br>NOT PRESENT . . . . . . 99994<br>REFUSED . . . . 99995<br>OTHER 99996	PUT THE 1ST BAR CODE LABEL HERE.<br>NOT PRESENT . . . . . . 99994<br>REFUSED . . . . 99995<br>OTHER 99996	PUT THE 1ST BAR CODE LABEL HERE.<br>NOT PRESENT . . . . . . 99994<br>REFUSED . . . . 99995<br>OTHER 99996
375	HIV TESTING: PLACE BAR CODE LABEL.	PUT THE 2ND BAR CODE LABEL HERE.<br>NOT PRESENT . . . . . . 99994<br>REFUSED . . . . 99995<br>OTHER 99996	PUT THE 2ND BAR CODE LABEL HERE.<br>NOT PRESENT . . . . . . 99994<br>REFUSED . . . . 99995<br>OTHER 99996	PUT THE 2ND BAR CODE LABEL HERE.<br>NOT PRESENT . . . . . . 99994<br>REFUSED . . . . 99995<br>OTHER 99996
375A	OFFER HIV SELF-TEST KIT TO RESPONDENT WHO CONSENTED TO HIV TESTING.	TEST KIT ACCEPTED . . . . 1<br>TEST KIT REFUSED . . . . . . 2<br>TEST KIT NOT OFFERRED 3<br>NOT PRESENT . . . . 4<br>OTHER 6	TEST KIT ACCEPTED . . . . 1<br>TEST KIT REFUSED . . . . . . 2<br>TEST KIT NOT OFFERRED 3<br>NOT PRESENT . . . . 3<br>OTHER 6	TEST KIT ACCEPTED . . . . 1<br>TEST KIT REFUSED . . . . . . 2<br>TEST KIT NOT OFFERRED 3<br>NOT PRESENT . . . . 3<br>OTHER 6
376	CHECK 374 AND 375: AT LEAST ONE BAR CODE LABEL PRESENT?	IF CONSENT GRANTED FOR EITHER TEST, PUT 3RD BAR CODE LABEL ON THE RESPONDENT'S FILTER PAPER CARD AND THE 4TH BAR CODE ON THE TRANSMITTAL FORM.	IF CONSENT GRANTED FOR EITHER TEST, PUT 3RD BAR CODE LABEL ON THE RESPONDENT'S FILTER PAPER CARD AND THE 4TH BAR CODE ON THE TRANSMITTAL FORM.	IF CONSENT GRANTED FOR EITHER TEST, PUT 3RD BAR CODE LABEL ON THE RESPONDENT'S FILTER PAPER CARD AND THE 4TH BAR CODE ON THE TRANSMITTAL FORM.

		MAN 1	MAN 2	MAN 3
	NAME FROM LIST.	NAME ______	NAME ______	NAME ______
377	Please show me all the prescribed medicines that you take regularly or daily.<br><br>RECORD ALL MEDICATION/DRUG NAMES.	MEDICINES SEEN . . . . . . 1<br>NONE . . . . 2<br>NOT PRESENT . . . . 3<br>REFUSED . . . . 4<br>OTHER 6	MEDICINES SEEN . . . . . . 1<br>NONE . . . . 2<br>NOT PRESENT . . . . 3<br>REFUSED . . . . 4<br>OTHER 6	MEDICINES SEEN . . . . . . 1<br>NONE . . . . 2<br>NOT PRESENT . . . . 3<br>REFUSED . . . . 4<br>OTHER 6
		DRUG NAME______	DRUG NAME______	DRUG NAME______
		DRUG NAME______	DRUG NAME______	DRUG NAME______
		DRUG NAME______	DRUG NAME______	DRUG NAME______
		DRUG NAME______	DRUG NAME______	DRUG NAME______
		DRUG NAME______	DRUG NAME______	DRUG NAME______
		DRUG NAME______	DRUG NAME______	DRUG NAME______
		DRUG NAME______	DRUG NAME______	DRUG NAME______
		DRUG NAME______	DRUG NAME______	DRUG NAME______
		DRUG NAME______	DRUG NAME______	DRUG NAME______
		DRUG NAME______	DRUG NAME______	DRUG NAME______
		DRUG NAME______	DRUG NAME______	DRUG NAME______
		DRUG NAME______	DRUG NAME______	DRUG NAME______
378	GO BACK TO 302 IN NEXT COLUMN OF THIS QUESTIONNAIRE OR IN THE FIRST COLUMN OF AN ADDITIONAL QUESTIONNAIRE; IF NO MORE MEN, END INTERVIEW.			

FIELDWORKER'S OBSERVATIONS

TO BE FILLED IN AFTER COMPLETING BIOMARKERS

SUPERVISOR'S OBSERVATIONS

2016 SOUTH AFRICA DEMOGRAPHIC AND HEALTH SURVEY
WOMAN'S QUESTIONNAIRE

IDENTIFICATION

PLACE NAME ______

NAME OF HOUSEHOLD HEAD ______

CLUSTER NUMBER . . . . [ ][ ][ ][ ]

HOUSEHOLD NUMBER . . . . [ ][ ][ ][ ]

NAME AND LINE NUMBER OF WOMAN ______ [ ][ ]

HOUSEHOLD SELECTED FOR MALE SURVEY AND FULL BIOMARKERS? (YES = 1; NO = 2) . . . . . . [ ]

RESPONDENT SELECTED FOR THE HOUSEHOLD RELATIONS MODULE? (YES = 1; NO = 2) . . . . . . [ ]

INTERVIEWER VISITS

	1	2	3	FINAL VISIT
DATE	______	______	______	DAY [ ][ ]<br>MONTH [ ][ ]<br>YEAR 2 0 1 [ ]
INTERVIEWER'S NAME	______	______	______	INT. NO. [ ][ ][ ][ ]
RESULT*	______	______	______	RESULT* [ ]
NEXT VISIT: DATE	______	______		TOTAL NUMBER OF VISITS [ ]
TIME	______	______		

*RESULT CODES:
1 COMPLETED
2 NOT AT HOME
3 POSTPONED
4 REFUSED
5 PARTLY COMPLETED
6 INCAPACITATED
7 OTHER ______ (SPECIFY)

LANGUAGE OF QUESTIONNAIRE** 0 1

LANGUAGE OF INTERVIEW** [ ][ ]

HOME LANGUAGE OF RESPONDENT** [ ][ ]

TRANSLATOR USED (YES = 1, NO = 2) [ ]

LANGUAGE OF QUESTIONNAIRE** **ENGLISH**

**LANGUAGE CODES:

01 ENGLISH	05 seSOTHO	09 tshiVENDA
02 AFRIKAANS	06 seTSWANA	10 xiTSONGA
03 isiXHOSA	07 sePEDI	11 isiNDEBELE
04 isiZULU	08 siSWATI	12 OTHER

SUPERVISOR

______ NAME

[ ][ ][ ][ ] NUMBER

100A	CHECK RESPONDENT'S AGE AND MARITAL STATUS IN HOUSEHOLD QUESTIONNAIRE.	
	AGE 15-17 AND NEVER IN UNION ☐ ↓ AGE 18 AND ABOVE OR AGE 15-17 AND EVER IN UNION ☐	→ 100C

INTRODUCTION AND CONSENT (PARENT/GUARDIAN)

100B
Hello. My name is _______________________. I am working with Statistics South Africa. We are conducting a survey about health and other topics all over South Africa. The information we collect will help the government to plan health services. Your household was selected for the survey. I would like to talk to (NAME OF MINOR) about her health and well-being. The questions usually take about 45 to 60 minutes. All of the answers (NAME OF MINOR) gives will be confidential and will not be shared with anyone other than members of our survey team. (NAME OF MINOR) doesn't have to be in the survey, but we hope you will agree to allow (NAME OF MINOR) to answer the questions since (NAME OF MINOR)'s views are important.

In case you need more information about the survey, you may contact the person listed on the card that has already been given to your household.

Do you have any questions?
May I begin the interview with (NAME OF MINOR) now?

SIGNATURE OF INTERVIEWER _______________________ DATE _______________

PARENT/GUARDIAN AGREES MINOR MAY BE INTERVIEWED . . 1 ↓   PARENT/GUARDIAN DOES NOT AGREE TO ALLOW MINOR TO BE INTERVIEWED . . 2 → END

INTRODUCTION AND CONSENT (RESPONDENT)

100C
Hello. My name is _______________________. I am working with Statistics South Africa. We are conducting a survey about health and other topics all over South Africa. The information we collect will help the government to plan health services. Your household was selected for the survey. The questions usually take about 45 to 60 minutes. All of the answers you give will be confidential and will not be shared with anyone other than members of our survey team. You don't have to be in the survey, but we hope you will agree to answer the questions since your views are important. If I ask you any question you don't want to answer, just let me know and I will go on to the next question or you can stop the interview at any time.

In case you need more information about the survey, you may contact the person listed on this information sheet.

GIVE INFORMATION SHEET.

Do you have any questions?
May I begin the interview now?

SIGNATURE OF INTERVIEWER _______________________ DATE _______________

RESPONDENT AGREES TO BE INTERVIEWED . . 1 ↓   RESPONDENT DOES NOT AGREE TO BE INTERVIEWED . . 2 → END

SECTION 1. RESPONDENT'S BACKGROUND

NO.	QUESTIONS AND FILTERS	CODING CATEGORIES	SKIP
101	RECORD THE TIME.	HOURS . . . . ☐☐<br>MINUTES . . . . ☐☐	
102	How long have you been living continuously in (NAME OF CURRENT CITY, TOWN OR VILLAGE OF RESIDENCE)?<br><br>IF LESS THAN ONE YEAR, RECORD '00' YEARS.	YEARS . . . . ☐☐<br>ALWAYS . . . . 95<br>VISITOR 96	95, 96 → 105
103	Just before you moved here, where did you live?<br><br>PROBE: Is that a city, a town, a rural area, a farm, a tribal area, or an informal settlement?	CITY . . . . 1<br>TOWN . . . . 2<br>RURAL AREA . . . . 3<br>FARM . . . . 4<br>TRIBAL AREA . . . . 5<br>INFORMAL SETTLEMENT 6	
104	Before you moved here, which province did you live in?	WESTERN CAPE . . . . 01<br>EASTERN CAPE . . . . 02<br>NORTHERN CAPE . . . . 03<br>FREE STATE . . . . 04<br>KWAZULU-NATAL . . . . 05<br>NORTH WEST . . . . 06<br>GAUTENG . . . . 07<br>MPUMALANGA . . . . 08<br>LIMPOPO . . . . 09<br>SADC COUNTRY . . . . 16<br>OTHER COUNTRY 26	

SECTION 1. RESPONDENT'S BACKGROUND

NO.	QUESTIONS AND FILTERS	CODING CATEGORIES	SKIP
105	On what day, month, and year were you born?	DAY . . . . [ ][ ]<br>DON'T KNOW DAY . . . . 98<br>MONTH . . . . [ ][ ]<br>DON'T KNOW MONTH . . . . 98<br>YEAR . . . . [ ][ ][ ][ ]<br>DON'T KNOW YEAR 9998	
106	How old were you at your last birthday?<br><br>COMPARE AND CORRECT 105 AND/OR 106 IF INCONSISTENT. IF AGE 95 OR OLDER, RECORD 95.	AGE IN COMPLETED YEARS . . . . [ ][ ]	
106A	Which population group do you consider yourself: black, white, coloured, Indian or something else?	BLACK/AFRICAN . . . . 1<br>WHITE . . . . 2<br>COLOURED . . . . 3<br>INDIAN/ASIAN . . . . 4<br>OTHER ______________________ 6<br>(SPECIFY)	
107	Have you ever attended an educational institution?	YES . . . . 1<br>NO 2	 → 111
108	What is the highest level you attended: primary, secondary, or higher than secondary?	PRIMARY . . . . 1<br>SECONDARY . . . . 2<br>HIGHER THAN SECONDARY 3	
109	What is the highest grade or form you completed at that level?	**PRIMARY SCHOOL**<br>LESS THAN 1 YEAR COMPLETED . . . . 00<br>GRADE 1/SUB A/CLASS 1 . . . . 11<br>GRADE 2/SUB B/CLASS 2 . . . . 12<br>GRADE 3/STANDARD 1/<br>AET 1 (KHA RI GUDE, SANLI) . . . . 13<br>GRADE 4/STANDARD 2 . . . . 14<br>GRADE 5/STANDARD 3/AET 2 . . . . 15<br>GRADE 6 /STANDARD 4 . . . . 16<br>GRADE 7/STANDARD 5/AET 3 . . . . 17<br><br>**SECONDARY SCHOOL**<br>LESS THAN 1 YEAR COMPLETED . . . . 20<br>GRADE 8/STANDARD 6/FORM 1/NTC 1/<br>N1/NC (V) LEVEL 2 . . . . 21<br>GRADE 9/STANDARD 7/FORM 2/AET 4/NTC 2/<br>N2/NC (V) LEVEL 3 . . . . 22<br>GRADE 10/STANDARD 8/FORM 3/NTC 3/<br>N3/NC (V) LEVEL 4 . . . . 23<br>GRADE 11/STANDARD 9/FORM 4 . . . . 24<br>CERTIFICATE OR DIPLOMA WITH LESS THAN<br>GRADE 12/STANDARD 10 COMPLETED . . 25<br>GRADE 12/STANDARD 10/FORM 5/MATRIC . . 26<br>N4/NTC4 . . . . 27<br>N5/NTC5 . . . . 28<br>N6/NTC6 . . . . 29<br><br>**HIGHER EDUCATION**<br>FURTHER STUDIES INCOMPLETE OR ONGOII 30<br>CERTIFICATE OR DIPLOMA WITH GRADE 12/<br>STANDARD 10 COMPLETED . . . . 31<br>HIGHER DIPLOMA (TECHNIKON/<br>U. OF TECHNOLOGY) . . . . 32<br>POST HIGHER DIPLOMA (TECHNIKON/<br>U. TECHNOLOGY MASTERS, DOCTORAL) 33<br>BACHELORS DEGREE/BACHELORS DEGREE<br>AND POST GRADUATE DIPLOMA . . . . . 34<br>HONOURS DEGREE . . . . 35<br>HIGHER DEGREE (MASTERS, DOCTORATI . . 36	
110	CHECK 108:<br>PRIMARY OR SECONDARY [ ] ↓	HIGHER [ ] →	113

SECTION 1. RESPONDENT'S BACKGROUND

NO.	QUESTIONS AND FILTERS	CODING CATEGORIES	SKIP
111	Now I would like you to read this sentence to me.<br><br>SHOW CARD TO RESPONDENT.<br><br>IF RESPONDENT CANNOT READ WHOLE SENTENCE, PROBE: Can you read any part of the sentence to me?	CANNOT READ AT ALL . . . . 1<br>ABLE TO READ ONLY PART OF THE SENTENCE . . . . 2<br>ABLE TO READ WHOLE SENTENCE . . . . 3<br>NO CARD WITH REQUIRED LANGUAGE ______ 4<br>(SPECIFY LANGUAGE)<br>BLIND/VISUALLY IMPAIRED 5	
112	CHECK 111:<br>CODE '2', '3' OR '4' CIRCLED ☐ ↓ CODE '1' OR '5' CIRCLED ☐ →		114
113	Do you read a newspaper or magazine at least once a week, less than once a week or not at all?	AT LEAST ONCE A WEEK . . . . 1<br>LESS THAN ONCE A WEEK . . . . 2<br>NOT AT ALL 3	
114	Do you listen to the radio at least once a week, less than once a week or not at all?	AT LEAST ONCE A WEEK . . . . 1<br>LESS THAN ONCE A WEEK . . . . 2<br>NOT AT ALL 3	
115	Do you watch television at least once a week, less than once a week or not at all?	AT LEAST ONCE A WEEK . . . . 1<br>LESS THAN ONCE A WEEK . . . . 2<br>NOT AT ALL 3	
116	Do you own a cell phone?	YES . . . . 1<br>NO 2	 → 118
117	Do you use your cell phone for any financial transactions?	YES . . . . 1<br>NO 2	
118	Do you have an account in a bank or other financial institution that you yourself use?	YES . . . . 1<br>NO 2	
119	Have you ever used the internet?	YES . . . . 1<br>NO 2	 → 124
120	In the last 12 months, have you used the internet?<br><br>IF NECESSARY, PROBE FOR USE FROM ANY LOCATION, WITH ANY DEVICE.	YES . . . . 1<br>NO 2	 → 124
121	During the last one month, how often did you use the internet: almost every day, at least once a week, less than once a week, or not at all?	ALMOST EVERY DAY . . . . 1<br>AT LEAST ONCE A WEEK . . . . 2<br>LESS THAN ONCE A WEEK . . . . 3<br>NOT AT ALL 4	
124	In the last 12 months, how many times have you been away from home for one or more nights?	NUMBER OF TIMES . . . . ☐☐<br><br>NONE 00	 <br>→ 126
125	In the last 12 months, have you been away from home for more than one month at a time?	YES . . . . 1<br>NO 2	
126	CHECK 106: AGE OF RESPONDENT<br>AGE 15-49 ☐ ↓ AGE 50 AND ABOVE ☐ →		701

SECTION 2. REPRODUCTION

NO.	QUESTIONS AND FILTERS	CODING CATEGORIES	SKIP
201	Now I would like to ask about all the births you have had during your life. Have you ever given birth?	YES . . . 1<br>NO . . . 2	 → 206
202	Do you have any sons or daughters to whom you have given birth who are now living with you?	YES . . . 1<br>NO . . . 2	 → 204
203	a) How many sons live with you?<br>b) And how many daughters live with you?<br>IF NONE, RECORD '00'.	a) SONS AT HOME . . . [ ][ ]<br>b) DAUGHTERS AT HOME . . . [ ][ ]	
204	Do you have any sons or daughters to whom you have given birth who are alive but do not live with you?	YES . . . 1<br>NO . . . 2	 → 206
205	a) How many sons are alive but do not live with you?<br>b) And how many daughters are alive but do not live with you?<br>IF NONE, RECORD '00'.	a) SONS ELSEWHERE . . . [ ][ ]<br>b) DAUGHTERS ELSEWHERE . . . [ ][ ]	
206	Have you ever given birth to a boy or girl who was born alive but later died?<br>IF NO, PROBE: Any baby who cried, who made any movement, sound, or effort to breathe, or who showed any other signs of life even if for a very short time?	YES . . . 1<br>NO . . . 2	 → 208
207	a) How many boys have died?<br>b) And how many girls have died?<br>IF NONE, RECORD '00'.	a) BOYS DEAD . . . [ ][ ]<br>b) GIRLS DEAD . . . [ ][ ]	
208	SUM ANSWERS TO 203, 205, AND 207, AND ENTER TOTAL. IF NONE, RECORD '00'.	TOTAL BIRTHS . . . [ ][ ]	
209	CHECK 208:<br>Just to make sure that I have this right: you have had in TOTAL _____ births during your life. Is that correct?<br>YES [ ] ↓ NO [ ] → PROBE AND CORRECT 201-208 AS NECESSARY.		
210	CHECK 208:<br>ONE OR MORE BIRTHS [ ] ↓ NO BIRTHS [ ] →		→ 226

211	Now I would like to record the names of all your births, whether still alive or not, starting with the first one you had.<br>RECORD NAMES OF ALL THE BIRTHS IN 212. RECORD TWINS AND TRIPLETS ON SEPARATE ROWS. IF THERE ARE MORE THAN 6 BIRTHS, USE AN ADDITIONAL QUESTIONNAIRE, STARTING WITH THE SECOND ROW.

212	213	214	215	215A	216	217	218	219	220	220A	221
What name was given to your (first/ next) baby?<br><br>RECORD NAME. BIRTH HISTORY NUMBER.	Is (NAME) a boy or a girl?	Were any of these births twins?	On what day, month, and year was (NAME) born?	IF BIRTH SINCE JANUARY 2011:<br>How many months were you pregnant before the birth of (NAME)?<br><br>ENTER 'B' IN THE MONTH OF BIRTH IN THE CALENDAR. WRITE THE NAME OF THE CHILD TO THE LEFT OF THE 'B' CODE. PLACE A 'P' IN EACH OF THE PRECEDING MONTHS ACCORDING TO THE DURATION OF THE PREGNANCY. (NOTE: THE NUMBER OF 'P's MUST BE ONE LESS THAN THE NUMBER OF MONTHS THAT THE PREGNANCY LASTED.)<br><br>C	Is (NAME) still alive?	IF ALIVE:<br>How old was (NAME) at (NAME)'s last birthday?<br><br>RECORD AGE IN COMP- LETED YEARS.	IF ALIVE:<br>Is (NAME) living with you?	IF ALIVE:<br>RECORD HOUSEHOLD LINE NUMBER OF CHILD. RECORD '00' IF CHILD NOT LISTED IN HOUSEHOLD.	IF DEAD:<br>How old was (NAME) when (he/she) died?<br><br>IF '12 MONTHS' OR '1 YR', ASK: Did (NAME) have (his/her) first birthday?<br><br>THEN ASK: Exactly how many months old was (NAME) when (he/she) died?<br><br>RECORD DAYS IF LESS THAN 1 MONTH; MONTHS IF LESS THAN TWO YEARS; OR YEARS.	IF DEAD:<br>IF BIRTH SINCE JANUARY 2011:<br>Where did (NAME) die?<br><br>At a health facility, at home, or somewhere else?	Were there any other live births between (NAME OF PREVIOUS BIRTH) and (NAME), including any children who died after birth?
01	BOY 1<br>GIRL 2	SING 1<br>MULT 2	DAY<br>MONTH<br>YEAR	MONTHS	YES 1<br>NO 2<br>(SKIP TO 220)	AGE IN YEARS	YES 1<br>NO 2	HOUSEHOLD LINE NUMBER<br>(NEXT BIRTH)	DAYS 1<br>MONTHS 2<br>YEARS 3	HEALTH FACILITY ... 1<br>HOME .... 2<br>ELSEWHERE . 3	
02	BOY 1<br>GIRL 2	SING 1<br>MULT 2	DAY<br>MONTH<br>YEAR	MONTHS	YES 1<br>NO 2<br>(SKIP TO 220)	AGE IN YEARS	YES 1<br>NO 2	HOUSEHOLD LINE NUMBER<br>(SKIP TO 221)	DAYS 1<br>MONTHS 2<br>YEARS 3	HEALTH FACILITY ... 1<br>HOME .... 2<br>ELSEWHERE . 3	YES 1 (ADD BIRTH)<br>NO 2 (NEXT BIRTH)
03	BOY 1<br>GIRL 2	SING 1<br>MULT 2	DAY<br>MONTH<br>YEAR	MONTHS	YES 1<br>NO 2<br>(SKIP TO 220)	AGE IN YEARS	YES 1<br>NO 2	HOUSEHOLD LINE NUMBER<br>(SKIP TO 221)	DAYS 1<br>MONTHS 2<br>YEARS 3	HEALTH FACILITY ... 1<br>HOME .... 2<br>ELSEWHERE . 3	YES 1 (ADD BIRTH)<br>NO 2 (NEXT BIRTH)
04	BOY 1<br>GIRL 2	SING 1<br>MULT 2	DAY<br>MONTH<br>YEAR	MONTHS	YES 1<br>NO 2<br>(SKIP TO 220)	AGE IN YEARS	YES 1<br>NO 2	HOUSEHOLD LINE NUMBER<br>(SKIP TO 221)	DAYS 1<br>MONTHS 2<br>YEARS 3	HEALTH FACILITY ... 1<br>HOME .... 2<br>ELSEWHERE . 3	YES 1 (ADD BIRTH)<br>NO 2 (NEXT BIRTH)
05	BOY 1<br>GIRL 2	SING 1<br>MULT 2	DAY<br>MONTH<br>YEAR	MONTHS	YES 1<br>NO 2<br>(SKIP TO 220)	AGE IN YEARS	YES 1<br>NO 2	HOUSEHOLD LINE NUMBER<br>(SKIP TO 221)	DAYS 1<br>MONTHS 2<br>YEARS 3	HEALTH FACILITY ... 1<br>HOME .... 2<br>ELSEWHERE . 3	YES 1 (ADD BIRTH)<br>NO 2 (NEXT BIRTH)
06	BOY 1<br>GIRL 2	SING 1<br>MULT 2	DAY<br>MONTH<br>YEAR	MONTHS	YES 1<br>NO 2<br>(SKIP TO 220)	AGE IN YEARS	YES 1<br>NO 2	HOUSEHOLD LINE NUMBER<br>(SKIP TO 221)	DAYS 1<br>MONTHS 2<br>YEARS 3	HEALTH FACILITY ... 1<br>HOME .... 2<br>ELSEWHERE . 3	YES 1 (ADD BIRTH)<br>NO 2 (NEXT BIRTH)

SECTION 2. REPRODUCTION

NO.	QUESTIONS AND FILTERS	CODING CATEGORIES	SKIP
222	Have you had any live births since the birth of (NAME OF LAST BIRTH)?	YES . . . . . 1 (RECORD BIRTH(S) IN TABLE)<br>NO 2	
223	COMPARE 208 WITH NUMBER OF BIRTHS IN BIRTH HISTORY<br>NUMBERS ARE SAME ☐<br>NUMBERS ARE DIFFERENT ☐ (PROBE AND RECONCILE)		
224	CHECK 215: ENTER THE NUMBER OF BIRTHS IN 2011-2016	NUMBER OF BIRTHS . . . . . ☐<br>NONE 0	
226	Are you pregnant now?	YES . . . . . 1<br>NO . . . . . 2<br>UNSURE 8	2, 8 → 230
227	How many months pregnant are you?<br>RECORD NUMBER OF COMPLETED MONTHS.<br>**C** ENTER 'P's IN THE CALENDAR, BEGINNING WITH THE MONTH OF INTERVIEW AND FOR THE TOTAL NUMBER OF COMPLETED MONTHS.	MONTHS . . . . . ☐☐	
228	When you got pregnant, did you want to get pregnant at that time?	YES . . . . . 1<br>NO 2	1 → 230
229	CHECK 208: TOTAL NUMBER OF BIRTHS<br>ONE OR MORE ☐ → a) Did you want to have a baby later on or did you not want any more children?<br>NONE ☐ → b) Did you want to have a baby later on or did you not want any children?	LATER . . . . . 1<br>NO MORE/NONE 2	
230	Have you ever had a pregnancy that miscarried, was terminated, or ended in a stillbirth?	YES . . . . . 1<br>NO 2	2 → 239
231	When did the last such pregnancy end?	MONTH . . . . . ☐☐<br>YEAR . . . . . ☐☐☐☐	
232	CHECK 231:<br>LAST PREGNANCY ENDED IN 2011-2016 ☐<br>LAST PREGNANCY ENDED IN 2010 OR EARLIER ☐		→ 233A<br>→ 239

NO.	QUESTIONS AND FILTERS		CODING CATEGORIES		SKIP
LINE NO.	233<br>In what month and year did the preceding such pregnancy end?	233A<br>Did that pregnancy end in a spontaneous miscarriage, an induced abortion, or a stillbirth?	234<br>How many months pregnant were you when that pregnancy ended?	235<br>Since January 2011, have you had any other pregnancies that did not result in a live birth?	
01		MISCARRIAGE 1<br>ABORTION 2<br>STILLBIRTH 3	MONTHS	YES 1<br>NO 2	1 → NEXT LINE<br>2 → 236
02	MONTH YEAR	MISCARRIAGE 1<br>ABORTION 2<br>STILLBIRTH 3	MONTHS	YES 1<br>NO 2	1 → NEXT LINE<br>2 → 236
03	MONTH YEAR	MISCARRIAGE 1<br>ABORTION 2<br>STILLBIRTH 3	MONTHS	YES 1<br>NO 2	1 → NEXT LINE<br>2 → 236
04	MONTH YEAR	MISCARRIAGE 1<br>ABORTION 2<br>STILLBIRTH 3	MONTHS	YES 1<br>NO 2	→ 236

NO.	QUESTIONS AND FILTERS	CODING CATEGORIES	SKIP
236	C FOR EACH PREGNANCY THAT DID NOT END IN A LIVE BIRTH IN 2011-2016 OR LATER, ENTER 'C' FOR MISCARRIAGE, 'A' FOR INDUCED ABORTION, OR 'S' FOR STILLBIRTH IN THE CALENDAR IN THE MONTH THAT THE PREGNANCY TERMINATED AND 'P' FOR THE REMAINING NUMBER OF COMPLETED MONTHS OF PREGNANCY.IF THERE ARE MORE THAN FOUR PREGNANCIES THAT DID NOT END IN A LIVE BIRTH, USE AN ADDITIONAL QUESTIONNAIRE STARTING ON THE SECOND LINE.		
236A	CHECK 233A: HAD INDUCED ABORTION SINCE JANUARY 2011?<br>YES ↓ NO		NO → 237
236B	The most recent time you had an induced abortion, what method was used?	SURGICAL ABORTION . . . 11<br>MEDICAL ABORTION . . . 21<br>SURGICAL AND MEDICAL . . . 31<br>SELF-INDUCED . . . 41<br>DON'T KNOW . . . 98	21 → 236D<br>41, 98 → 236E
236C	Where was the procedure done?<br><br>PROBE TO IDENTIFY THE TYPE OF SOURCE.<br><br>IF UNABLE TO DETERMINE IF PUBLIC OR PRIVATE SECTOR, RECORD 96 AND WRITE THE NAME OF THE PLACE.	**PUBLIC SECTOR**<br>GOVT. HOSPITAL . . . 11<br>GOVT. CLINIC/COMMUNITY HEALTH CENTRE 12<br>OTHER PUBLIC SECTOR<br>_____ 16<br>(SPECIFY)<br>**PRIVATE MEDICAL SECTOR**<br>PRIVATE HOSPITAL/CLINIC . . . 21<br>MARIE STOPES CLINIC . . . 22<br>PRIVATE DOCTOR . . . 23<br>OTHER PRIVATE MEDICAL SECTOR<br>_____ 26<br>(SPECIFY)<br>**OTHER SOURCE**<br>BACKSTREET ABORTION . . . 31<br>OTHER _____ 96<br>(SPECIFY)	→ 236E

NO.	QUESTIONS AND FILTERS	CODING CATEGORIES	SKIP
236D	Where did you get the drug?<br><br>PROBE TO IDENTIFY THE TYPE OF SOURCE.<br><br>IF UNABLE TO DETERMINE IF PUBLIC OR PRIVATE SECTOR, RECORD 96 AND WRITE THE NAME OF THE PLACE.	**PUBLIC SECTOR**<br>GOVT. HOSPITAL . . . . . 11<br>GOVT. CLINIC/COMMUNITY HEALTH CENTRE 12<br>COMMUNITY HEALTH WORKER . . . . . 13<br>OTHER PUBLIC SECTOR<br>_____ 16<br>(SPECIFY)<br><br>**PRIVATE MEDICAL SECTOR**<br>PRIVATE HOSPITAL/CLINIC . . . . . 21<br>CHEMIST/PHARMACY . . . . . 22<br>PRIVATE DOCTOR . . . . . 23<br>OTHER PRIVATE MEDICAL SECTOR<br>_____ 26<br>(SPECIFY)<br><br>**OTHER SOURCE**<br>BACKSTREET ABORTION . . . . . 31<br>TRADITIONAL HEALER . . . . . 32<br><br>OTHER _____ 96<br>(SPECIFY)	
236E	We have spoken about pregnancy losses that occurred since 2011. Did you have any miscarriages, terminations, or stillbirths that ended before 2011?	YES . . . . . 1<br>NO 2	→ 238<br>→ 239
237	Did you have any miscarriages, terminations or stillbirths that ended before 2011?	YES . . . . . 1<br>NO 2	 → 239
238	When did the last such pregnancy that terminated before 2011 end?	MONTH . . . . . [ ][ ]<br>YEAR . . . . . [ ][ ][ ][ ]	
239	When did your last menstrual period start?<br><br>_____<br>(DATE, IF GIVEN)	DAYS AGO . . . . . 1 [ ][ ]<br>WEEKS AGO . . . . . 2 [ ][ ]<br>MONTHS AGO . . . . . 3 [ ][ ]<br>YEARS AGO . . . . . 4 [ ][ ]<br><br>IN MENOPAUSE/<br>HAS HAD HYSTERECTOMY . . . . . 994<br><br>BEFORE LAST BIRTH . . . . . 995<br><br>NEVER MENSTRUATED 996	
240	From one menstrual period to the next, are there certain days when a woman is more likely to become pregnant?	YES . . . . . 1<br>NO . . . . . 2<br>DON'T KNOW 8	 } → 242
241	Is this time just before her period begins, during her period, right after her period has ended, or halfway between two periods?	JUST BEFORE HER PERIOD<br>BEGINS . . . . . 1<br>DURING HER PERIOD . . . . . 2<br>RIGHT AFTER HER<br>PERIOD HAS ENDED . . . . . 3<br>HALFWAY BETWEEN<br>TWO PERIODS . . . . . 4<br><br>OTHER _____ 6<br>(SPECIFY)<br>DON'T KNOW 8	
242	After the birth of a child, can a woman become pregnant before her menstrual period has returned?	YES . . . . . 1<br>NO . . . . . 2<br>DON'T KNOW 8	

SECTION 3. CONTRACEPTION

301	Now I would like to talk about family planning - the various ways or methods that a couple can use to delay or avoid a pregnancy. Which ways or methods have you heard about? MARK ALL METHODS DECLARED BY THE RESPONDENT.<br><br>FOR METHODS NOT MENTIONED SPONTANEOUSLY, ASK: Have you ever heard of (METHOD)?	
01	Female Sterilisation/Tubal Ligation/Tubes Cut/Tubes Binded.<br>PROBE: Women can have an operation to avoid having any more children.	YES . . . . . 1<br>NO 2
02	Male Sterilisation/Vasectomy/Tubes Cut/Tubes Binded.<br>PROBE: Men can have an operation to avoid having any more children.	YES . . . . . 1<br>NO 2
03	IUD.<br>PROBE: Women can have a loop or coil placed inside them by a doctor or a nurse which can prevent pregnancy for one or more years.	YES . . . . . 1<br>NO 2
04	Injectables/Depo.<br>PROBE: Women can have an injection by a health provider that stops them from becoming pregnant for one or more months.	YES . . . . . 1<br>NO 2
05	Implants/Norplant/Jadelle.<br>PROBE: Women can have one or more small rods placed in their upper arm by a doctor or nurse which can prevent pregnancy for one or more years.	YES . . . . . 1<br>NO 2
06	Pill.<br>PROBE: Women can take a pill every day to avoid becoming pregnant.	YES . . . . . 1<br>NO 2
07	Male Condom.<br>PROBE: Men can put a rubber sheath on their penis before sexual intercourse.	YES . . . . . 1<br>NO 2
08	Female Condom.<br>PROBE: Women can place a sheath in their vagina before sexual intercourse.	YES . . . . . 1<br>NO 2
09	Emergency Contraception.<br>PROBE: As an emergency measure, within three days after they have unprotected sexual intercourse, women can take special pills to prevent pregnancy.	YES . . . . . 1<br>NO 2
10	Rhythm Method.<br>PROBE: To avoid pregnancy, women do not have sexual intercourse on the days of the month they think they can get pregnant.	YES . . . . . 1<br>NO 2
11	Withdrawal.<br>PROBE: Men can be careful and pull out before climax.	YES . . . . . 1<br>NO 2
12	Have you heard of any other ways or methods that women or men can use to avoid pregnancy?	YES, MODERN METHOD<br>__________ A<br>(SPECIFY)<br>YES, TRADITIONAL METHOD<br>__________ B<br>(SPECIFY)<br>NO . . . . . Y

NO.	QUESTIONS AND FILTERS	CODING CATEGORIES	SKIP
302	CHECK 226:<br>NOT PREGNANT OR UNSURE ☐ ↓	PREGNANT ☐	→ 312
303	Are you or your partner currently doing something or using any method to delay or avoid getting pregnant?	YES . . . 1<br>NO . . . 2	 → 312
304	Which method are you using?<br><br>RECORD ALL MENTIONED.<br><br>IF MORE THAN ONE METHOD MENTIONED, FOLLOW SKIP INSTRUCTION FOR HIGHEST METHOD IN LIST.	FEMALE STERILISATION . . . A<br>MALE STERILISATION . . . B<br>IUD . . . C<br>INJECTABLES - 3 MONTH DEPO . . . D<br>INJECTABLES - 2 MONTH NUR-ISTERATE . . E<br>IMPLANTS . . . F<br>PILL . . . G<br>MALE CONDOM . . . H<br>FEMALE CONDOM . . . I<br>EMERGENCY CONTRACEPTION . . . J<br>RHYTHM METHOD . . . K<br>WITHDRAWAL . . . L<br>OTHER MODERN METHOD . . . X<br>OTHER TRADITIONAL METHOD . . . Y	C–Y → 309
307	In what facility did the sterilisation take place?<br><br>PROBE TO IDENTIFY THE TYPE OF SOURCE.<br><br>IF UNABLE TO DETERMINE IF PUBLIC OR PRIVATE SECTOR, RECORD 96 AND WRITE THE NAME OF THE PLACE.	**PUBLIC SECTOR**<br>GOVT. HOSPITAL . . . 11<br>GOVT. HEALTH CLINIC/COMMUNITY HEALTH CENTRE . . . 12<br>OTHER PUBLIC SECTOR<br>________ 16<br>(SPECIFY)<br><br>**PRIVATE MEDICAL SECTOR**<br>PRIVATE HOSPITAL/CLINIC . . . 21<br>PRIVATE DOCTOR'S ROOM . . . 22<br>OTHER PRIVATE MEDICAL SECTOR<br>________ 26<br>(SPECIFY)<br><br>OTHER ________ 96<br>(SPECIFY)<br>DON'T KNOW . . . 98	
308	In what month and year was the sterilisation performed?	MONTH . . . ☐☐<br>YEAR . . . ☐☐☐☐	→ 310
309	Since what month and year have you been using (CURRENT METHOD) without stopping?<br><br>PROBE: For how long have you been using (CURRENT METHOD) now without stopping?	MONTH . . . ☐☐<br>YEAR . . . ☐☐☐☐	
310	CHECK 308 AND 309, 215 AND 231: ANY BIRTH OR PREGNANCY TERMINATION AFTER MONTH AND YEAR OF START OF USE OF CONTRACEPTION IN 308 OR 309<br><br>NO ☐ ↓<br><br>YES ☐ → GO BACK TO 308 OR 309, PROBE AND RECORD MONTH AND YEAR AT START OF CONTINUOUS USE OF CURRENT METHOD (MUST BE AFTER LAST BIRTH OR PREGNANCY TERMINATION).		

311	CHECK 308 AND 309:<br><br>YEAR IS 2011-2016 ☐<br>C ENTER CODE FOR METHOD USED IN MONTH OF INTERVIEW IN THE CALENDAR AND IN EACH MONTH BACK TO THE DATE STARTED USING.<br>THEN CONTINUE<br><br>YEAR IS 2010 OR EARLIER ☐<br>C ENTER CODE FOR METHOD USED IN MONTH OF INTERVIEW IN THE CALENDAR AND EACH MONTH BACK TO JANUARY 2011.<br>THEN (SKIP TO 324)
312	I would like to ask you some questions about the times you or your partner may have used a method to avoid getting pregnant during the last few years.<br>C USE CALENDAR TO PROBE FOR EARLIER PERIODS OF USE AND NONUSE, STARTING WITH MOST RECENT USE, BACK TO JANUARY 2011. USE NAMES OF CHILDREN, DATES OF BIRTH, AND PERIODS OF PREGNANCY AS REFERENCE POINTS.

		COLUMN 1	COLUMN 2	COLUMN 3
312A	MONTH AND YEAR OF START OF INTERVAL OF USE OR NON-USE.	MONTH ☐☐<br>☐☐☐☐<br>YEAR	MONTH ☐☐<br>☐☐☐☐<br>YEAR	MONTH ☐☐<br>☐☐☐☐<br>YEAR
312B	Between (EVENT) in (MONTH/YEAR) and (EVENT) in (MONTH/YEAR), did you or your partner use any method of contraception?	YES . . . . 1<br>NO . . . . 2<br>(SKIP TO 312I)	YES . . . . 1<br>NO . . . . 2<br>(SKIP TO 312I)	YES . . . . 1<br>NO . . . . 2<br>(SKIP TO 312I)
312C	Which method was that?	METHOD CODE . . ☐	METHOD CODE . . ☐	METHOD CODE . . ☐
312D	How many months after (EVENT) in (MONTH/YEAR) did you start to use (METHOD)?<br>CIRCLE '95' IF RESPONDENT GIVES THE DATE OF STARTING TO USE THE METHOD.	IMMEDIATELY . . . . . 00<br>MONTHS . . ☐☐<br>(SKIP TO 312F)<br>DATE GIVEN 95	IMMEDIATELY . . . . . 00<br>MONTHS . . ☐☐<br>(SKIP TO 312F)<br>DATE GIVEN 95	IMMEDIATELY . . . . . 00<br>MONTHS . . ☐☐<br>(SKIP TO 312F)<br>DATE GIVEN 95
312E	RECORD MONTH AND YEAR RESPONDENT STARTED USING METHOD.	MONTH ☐☐<br>☐☐☐☐<br>YEAR	MONTH ☐☐<br>☐☐☐☐<br>YEAR	MONTH ☐☐<br>☐☐☐☐<br>YEAR
312F	For how many months did you use (METHOD)?<br>CIRCLE '95' IF RESPONDENT GIVES THE DATE OF TERMINATION OF USE.	MONTHS . . ☐☐<br>(SKIP TO 312H)<br>DATE GIVEN 95	MONTHS . . ☐☐<br>(SKIP TO 312H)<br>DATE GIVEN 95	MONTHS . . ☐☐<br>(SKIP TO 312H)<br>DATE GIVEN 95
312G	RECORD MONTH AND YEAR RESPONDENT STOPPED USING METHOD.	MONTH ☐☐<br>☐☐☐☐<br>YEAR	MONTH ☐☐<br>☐☐☐☐<br>YEAR	MONTH ☐☐<br>☐☐☐☐<br>YEAR
312H	Why did you stop using (METHOD)?	REASON STOPPED . . . . . ☐	REASON STOPPED . . . . . ☐	REASON STOPPED . . . . . ☐
312I		GO BACK TO 312A IN NEXT COLUMN; OR, IF NO MORE GAPS, GO TO 313.	GO BACK TO 312A IN NEXT COLUMN; OR, IF NO MORE GAPS, GO TO 313.	GO BACK TO 312A IN NEW QUESTIONNAIRE; OR, IF NO MORE GAPS, GO TO 313.

SECTION 3. CONTRACEPTION

NO.	QUESTIONS AND FILTERS	CODING CATEGORIES	SKIP
313	CHECK THE CALENDAR FOR USE OF ANY CONTRACEPTIVE METHOD IN ANY MONTH<br>NO METHOD USED ☐ ↓	ANY METHOD USED ☐	→ 315
314	Have you ever used anything or tried in any way to delay or avoid getting pregnant?	YES . . . . . 1<br>NO 2	→ 326 (1, 2)
315	CHECK 304:<br><br>CIRCLE METHOD CODE:<br><br>IF MORE THAN ONE METHOD CODE CIRCLED IN 304, CIRCLE CODE FOR HIGHEST METHOD IN LIST.	NO CODE CIRCLED . . . . . 00<br>FEMALE STERILISATION . . . . . 01<br>MALE STERILISATION . . . . . 02<br>IUD . . . . . 03<br>INJECTABLES - 3 MONTH DEPO . . . . . 04<br>INJECTABLES - 2 MONTH NUR-ISTERATE . . 05<br>IMPLANTS . . . . . 06<br>PILL . . . . . 07<br>MALE CONDOM . . . . . 08<br>FEMALE CONDOM . . . . . 09<br>EMERGENCY CONTRACEPTION . . . . . 10<br>RHYTHM METHOD . . . . . 11<br>WITHDRAWAL . . . . . 12<br>OTHER MODERN METHOD . . . . . 95<br>OTHER TRADITIONAL METHOD 96	00 → 326<br>01 → 319<br>02 → 329<br><br>11, 12 → 323
316	You first started using (CURRENT METHOD) in (DATE FROM 309). Where did you get it at that time?<br><br>PROBE TO IDENTIFY THE TYPE OF SOURCE.<br><br>IF UNABLE TO DETERMINE IF PUBLIC OR PRIVATE SECTOR, RECORD 96 AND WRITE THE NAME OF THE PLACE.	**PUBLIC SECTOR**<br>GOVT. HOSPITAL . . . . . 11<br>GOVT. CLINIC/COMMUNITY HEALTH CENTRE 12<br>MOBILE CLINIC . . . . . 13<br>COMMUNITY HEALTH WORKER . . . . . 14<br>OTHER PUBLIC SECTOR<br>________ 16<br>(SPECIFY)<br><br>**PRIVATE MEDICAL SECTOR**<br>PRIVATE HOSPITAL/CLINIC . . . . . 21<br>CHEMIST/PHARMACY . . . . . 22<br>PRIVATE DOCTOR . . . . . 23<br>OTHER PRIVATE MEDICAL SECTOR<br>________ 26<br>(SPECIFY)<br><br>**OTHER SOURCE**<br>WORKPLACE/WORKPLACE CLINIC . . . . . 31<br>COMMUNITY CENTER, LIBRARY OR OTHER PUBLIC PLACE . . . . . 32<br>SHOP . . . . . 33<br>CHURCH . . . . . 34<br>FRIEND/RELATIVE . . . . . 35<br><br>OTHER ________ 96<br>(SPECIFY)	
317	CHECK 304:<br><br>CIRCLE METHOD CODE:<br><br>IF MORE THAN ONE METHOD CODE CIRCLED IN 304, CIRCLE CODE FOR HIGHEST METHOD IN LIST.	IUD . . . . . 03<br>INJECTABLES - 3 MONTH DEPO . . . . . 04<br>INJECTABLES - 2 MONTH NUR-ISTERATE . . 05<br>IMPLANTS . . . . . 06<br>PILL . . . . . 07<br>MALE CONDOM . . . . . 08<br>FEMALE CONDOM . . . . . 09<br>EMERGENCY CONTRACEPTION . . . . . 10<br>OTHER MODERN METHOD . . . . . 95<br>OTHER TRADITIONAL METHOD 96	08 → 323<br>09, 10, 95 → 322<br>96 → 323

NO.	QUESTIONS AND FILTERS	CODING CATEGORIES	SKIP
318	At that time, were you told about side effects or problems you might have with the method?	YES . . . 1<br>NO . . . 2	→ 321<br>→ 320
319	When you got sterilized, were you told about side effects or problems you might have with the method?	YES . . . 1<br>NO . . . 2	→ 321
320	Were you ever told by a nurse or health care worker about side effects or problems you might have with the method?	YES . . . 1<br>NO . . . 2	 → 322
321	Were you told what to do if you experienced side effects or problems?	YES . . . 1<br>NO . . . 2	
322	CHECK 318 AND 319:<br>ANY YES' ☐ / OTHER ☐<br>a) At that time, were you told about other methods of family planning that you could use?<br>b) When you obtained (CURRENT METHOD FROM 315) from (SOURCE OF METHOD FROM 307 OR 316), were you told about other methods of family planning that you could use?	YES . . . 1<br>NO . . . 2	→ 324
323	Were you ever told by a nurse or health care worker about other methods of family planning that you could use?	YES . . . 1<br>NO . . . 2	
324	CHECK 304:<br><br>CIRCLE METHOD CODE:<br><br>IF MORE THAN ONE METHOD CODE CIRCLED IN 304, CIRCLE CODE FOR HIGHEST METHOD IN LIST.	FEMALE STERILISATION . . . 01<br>MALE STERILISATION . . . 02<br>IUD . . . 03<br>INJECTABLES - 3 MONTH DEPO . . . 04<br>INJECTABLES - 2 MONTH NUR-ISTERATE . . 05<br>IMPLANTS . . . 06<br>PILL . . . 07<br>MALE CONDOM . . . 08<br>FEMALE CONDOM . . . 09<br>EMERGENCY CONTRACEPTION . . . 10<br>RHYTHM METHOD . . . 11<br>WITHDRAWAL . . . 12<br>OTHER MODERN METHOD . . . 95<br>OTHER TRADITIONAL METHOD . . . 96	(01–02) → 329<br><br>(11–12) → 329<br><br>(96) → 329

NO.	QUESTIONS AND FILTERS	CODING CATEGORIES	SKIP
325	Where did you obtain (CURRENT METHOD) the last time?<br><br>PROBE TO IDENTIFY THE TYPE OF SOURCE.<br><br>IF UNABLE TO DETERMINE IF PUBLIC OR PRIVATE SECTOR, RECORD 96 AND WRITE THE NAME OF THE PLACE.	**PUBLIC SECTOR**<br>GOVT. HOSPITAL . . . . . 11<br>GOVT. HEALTH CLINIC/COMMUNITY HEALTH CENTRE . . . . . 12<br>MOBILE CLINIC . . . . . 13<br>CHW . . . . . 14<br>OTHER PUBLIC SECTOR ____ 16<br>(SPECIFY)<br><br>**PRIVATE MEDICAL SECTOR**<br>PRIVATE HOSPITAL/CLINIC . . . . . 21<br>CHEMIST/PHARMACY . . . . . 22<br>PRIVATE DOCTOR . . . . . 23<br>OTHER PRIVATE MEDICAL SECTOR ____ 26<br>(SPECIFY)<br><br>**OTHER SOURCE**<br>WORKPLACE/WORKPLACE CLINIC . . . . . 31<br>COMMUNITY CENTER, LIBRARY OR OTHER PUBLIC PLACE . . . . . 32<br>SHOP . . . . . 33<br>CHURCH . . . . . 34<br>FRIEND/RELATIVE . . . . . 35<br><br>OTHER ____ 96<br>(SPECIFY)	→ 329
326	Do you know of a place where you can obtain a method of family planning?	YES . . . . . 1<br>NO 2	
329	CHECK 202: LIVING CHILDREN<br>YES ☐ / NO ☐<br>a) In the last 12 months, have you visited a health facility for care for yourself or your children?<br>b) In the last 12 months, have you visited a health facility for care for yourself?	YES . . . . . 1<br>NO 2	 2 → 401
330	Did any staff member at the health facility speak to you about family planning methods?	YES . . . . . 1<br>NO 2	

401	CHECK 224:<br>ONE OR MORE BIRTHS IN 2011-2016 ☐<br>NO BIRTHS IN 2011-2016 ☐ → 648		
402	CHECK 215: RECORD THE BIRTH HISTORY NUMBER IN 403 AND THE NAME AND SURVIVAL STATUS IN 404 FOR EACH BIRTH IN 2011-2016. ASK THE QUESTIONS ABOUT ALL OF THESE BIRTHS. BEGIN WITH THE LAST BIRTH. IF THERE ARE MORE THAN 2 BIRTHS, USE LAST COLUMN OF ADDITIONAL QUESTIONNAIRE(S).<br>Now I would like to ask some questions about your children born in the last five years. (We will talk about each separately.)		
403	BIRTH HISTORY NUMBER FROM 212 IN BIRTH HISTORY.	LAST BIRTH<br>BIRTH HISTORY NUMBER . . . . ☐☐	NEXT-TO-LAST BIRTH<br>BIRTH HISTORY NUMBER . . . . ☐☐
404	FROM 212 AND 216:	NAME ____________<br>LIVING ☐ DEAD ☐	NAME ____________<br>LIVING ☐ DEAD ☐
405	When you got pregnant with (NAME), did you want to get pregnant at that time?	YES . . . . 1 (SKIP TO 408)<br>NO 2	YES . . . . 1 (SKIP TO 426)<br>NO 2
406	CHECK 208:<br>ONLY ONE BIRTH ☐<br>a) Did you want to have a baby later on, or did you not want any children?<br>MORE THAN ONE BIRTH ☐<br>b) Did you want to have a baby later on, or did you not want any more children?	LATER . . . . 1<br>NO MORE/NONE . . . . 2 (SKIP TO 408)	LATER . . . . 1<br>NO MORE/NONE . . . . 2 (SKIP TO 426)
407	How much longer did you want to wait?	MONTHS . . . . 1 ☐☐<br>YEARS . . . . 2 ☐☐<br>DON'T KNOW 998	MONTHS . . . . 1 ☐☐<br>YEARS . . . . 2 ☐☐<br>DON'T KNOW 998
408	Did you see anyone for antenatal care for this pregnancy?	YES . . . . 1<br>NO . . . . 2 (SKIP TO 414)	
409	Whom did you see?<br>Anyone else?<br>PROBE TO IDENTIFY EACH TYPE OF PERSON AND RECORD ALL MENTIONED.	**HEALTH PERSONNEL**<br>DOCTOR/GYNAECOLOGIST A<br>NURSE/MIDWIFE . . . . B<br>**OTHER PERSON**<br>TRADITIONAL BIRTH ATTENDANT . . . . C<br>COMMUNITY HEALTH WORKER . . . . D<br>OTHER ____________ X<br>(SPECIFY)	

SECTION 4. PREGNANCY AND POSTNATAL CARE

NO.	QUESTIONS AND FILTERS	LAST BIRTH<br>NAME ________	NEXT-TO-LAST BIRTH<br>NAME ________
410	Where did you receive antenatal care for this pregnancy?<br><br>Anywhere else?<br><br>PROBE TO IDENTIFY THE TYPE OF SOURCE.<br><br>IF UNABLE TO DETERMINE IF PUBLIC OR PRIVATE SECTOR, RECORD 'X' AND WRITE THE NAME OF THE PLACE(S).	**HOME**<br>HER HOME . . . . A<br>OTHER HOME . . . . B<br><br>**PUBLIC SECTOR**<br>GOVERNMENT HOSPITAL . . C<br>GOVERNMENT CLINIC/ COMM. HEALTH CENTRE D<br>MOBILE CLINIC . . . . E<br>OTHER PUBLIC SECTOR<br>________ F<br>(SPECIFY)<br><br>**PRIVATE MEDICAL SECTOR**<br>PRIVATE HOSPITAL/DOCTOR G<br>OTHER PRIVATE MEDICAL SECTOR<br>________ H<br>(SPECIFY)<br><br>OTHER ________ X<br>(SPECIFY)	
411	How many months pregnant were you when you first received antenatal care for this pregnancy?	MONTHS . . . . [ ][ ]<br><br>DON'T KNOW 98	
412	How many times did you receive antenatal care during this pregnancy?	NUMBER OF TIMES . . . . . [ ][ ]<br><br>DON'T KNOW 98	
413	As part of your antenatal care during this pregnancy, were any of the following done at least once:<br>a) Was your blood pressure measured?<br>b) Did you give a urine sample?<br>c) Did you give a blood sample?<br>d) Were you asked about the use of alcohol?<br>e) Were you asked about smoking tobacco?	YES NO<br>a) BP . . . . 1 2<br>b) URINE . . . . 1 2<br>c) BLOOD . . . . 1 2<br>d) ALCOHOL . . . . . 1 2<br>e) SMOKING 1 2	
414	During this pregnancy, were you given an injection in the arm to prevent the baby from getting tetanus, that is, convulsions after birth or lockjaw?	YES . . . . 1<br>NO . . . . 2<br>(SKIP TO 417)<br>DON'T KNOW 8	
415	During this pregnancy, how many times did you get a tetanus injection?	TIMES . . . . [ ]<br><br>DON'T KNOW 8	
416	CHECK 415: TETANUS INJECTIONS	2 OR MORE TIMES [ ] (SKIP TO 420)<br>OTHER [ ]	

NO.	QUESTIONS AND FILTERS	LAST BIRTH<br>NAME ______	NEXT-TO-LAST BIRTH<br>NAME ______
417	At any time before this pregnancy, did you receive any tetanus injections?	YES . . . . . . 1<br>NO . . . . . . 2 (SKIP TO 420)<br>DON'T KNOW . . . . . . 8 (SKIP TO 420)	
418	Before this pregnancy, how many times did you receive a tetanus injection?<br><br>IF 7 OR MORE TIMES, RECORD '7'.	TIMES . . . . . . [ ]<br>DON'T KNOW 8	
419	CHECK 418:<br>ONLY ONE TIME [ ] ↓ MORE THAN ONE TIME [ ] ↓<br>a) How many years ago did you receive that tetanus injection?<br>b) How many years ago did you receive the last tetanus injection prior to this pregnancy?	YEARS AGO . . . . . [ ][ ]	
420	During this pregnancy, were you given or did you buy any iron tablets?<br><br>SHOW TABLETS.	YES . . . . . . 1<br>NO . . . . . . 2 (SKIP TO 426)<br>DON'T KNOW . . . . . . 8 (SKIP TO 426)	
421	During the whole pregnancy, for how many days did you take the tablets?<br><br>IF ANSWER IS NOT NUMERIC, PROBE FOR APPROXIMATE NUMBER OF DAYS.	DAYS . . . . . [ ][ ][ ]<br>DON'T KNOW 998	
426	When (NAME) was born, was (NAME) very large, larger than average, average, smaller than average, or very small?	VERY LARGE . . . . . . 1<br>LARGER THAN AVERAGE . . . . . . 2<br>AVERAGE . . . . . . 3<br>SMALLER THAN AVERAGE . . . . . . 4<br>VERY SMALL . . . . . . 5<br>DON'T KNOW 8	VERY LARGE . . . . . . 1<br>LARGER THAN AVERAGE . . . . . . 2<br>AVERAGE . . . . . . 3<br>SMALLER THAN AVERAGE . . . . . . 4<br>VERY SMALL . . . . . . 5<br>DON'T KNOW 8
427	Was (NAME) weighed at birth?	YES . . . . . . 1<br>NO . . . . . . 2 (SKIP TO 429)<br>DON'T KNOW . . . . . . 8 (SKIP TO 429)	YES . . . . . . 1<br>NO . . . . . . 2 (SKIP TO 429)<br>DON'T KNOW . . . . . . 8 (SKIP TO 429)

NO.	QUESTIONS AND FILTERS	LAST BIRTH<br>NAME ______	NEXT-TO-LAST BIRTH<br>NAME ______
428	How much did (NAME) weigh?<br><br>RECORD WEIGHT IN KILOGRAMS FROM ROAD TO HEALTH BOOKLET OR OTHER HEALTH CARD, IF AVAILABLE.	KG FROM BOOKLET/CARD<br>1 [ ] . [ ][ ][ ]<br>KG FROM RECALL<br>2 [ ] . [ ][ ][ ]<br>DON'T KNOW 99998	KG FROM BOOKLET/CARD<br>1 [ ] . [ ][ ][ ]<br>KG FROM RECALL<br>2 [ ] . [ ][ ][ ]<br>DON'T KNOW 99998
429	Who assisted with the delivery of (NAME)?<br><br>PROBE FOR THE TYPE(S) OF PERSON(S) AND RECORD ALL MENTIONED.<br><br>IF RESPONDENT SAYS NO ONE ASSISTED, PROBE TO DETERMINE WHETHER ANY ADULTS WERE PRESENT AT THE DELIVERY.	**HEALTH PERSONNEL**<br>DOCTOR/GYNAECOLOGIST A<br>NURSE/MIDWIFE . . . . B<br>**OTHER PERSON**<br>TRADITIONAL BIRTH ATTENDANT . . . . C<br>RELATIVE/FRIEND . . . . D<br>OTHER ______ X<br>(SPECIFY)<br>NO ONE ASSISTED . . . . Y	**HEALTH PERSONNEL**<br>DOCTOR/GYNAECOLOGIST A<br>NURSE/MIDWIFE . . . . B<br>**OTHER PERSON**<br>TRADITIONAL BIRTH ATTENDANT . . . . C<br>RELATIVE/FRIEND . . . . D<br>OTHER ______ X<br>(SPECIFY)<br>NO ONE ASSISTED . . . . Y
430	Where did you give birth to (NAME)?<br><br>PROBE TO IDENTIFY THE TYPE OF SOURCE.<br><br>IF UNABLE TO DETERMINE IF PUBLIC OR PRIVATE SECTOR, RECORD 96 AND WRITE THE NAME OF THE PLACE.	**HOME**<br>HER HOME . . . . 11<br>(SKIP TO 434)<br>OTHER HOME . . . . 12<br>**PUBLIC SECTOR**<br>GOVERNMENT HOSPITAL . . 21<br>GOVERNMENT CLINIC/ COMM. HEALTH CENTRE 22<br>MOBILE CLINIC . . . . 23<br>OTHER PUBLIC SECTOR<br>______ 26<br>(SPECIFY)<br>**PRIVATE MEDICAL SECTOR**<br>PRIVATE HOSPITAL/DOCTOR 31<br>OTHER PRIVATE MEDICAL SECTOR<br>______ 36<br>(SPECIFY)<br>OTHER ______ 96<br>(SPECIFY)<br>(SKIP TO 434)	**HOME**<br>HER HOME . . . . 11<br>(SKIP TO 434)<br>OTHER HOME . . . . 12<br>**PUBLIC SECTOR**<br>GOVERNMENT HOSPITAL . . 21<br>GOVERNMENT CLINIC/ COMM. HEALTH CENTRE 22<br>MOBILE CLINIC . . . . 23<br>OTHER PUBLIC SECTOR<br>______ 26<br>(SPECIFY)<br>**PRIVATE MEDICAL SECTOR**<br>PRIVATE HOSPITAL/DOCTOR 31<br>OTHER PRIVATE MEDICAL SECTOR<br>______ 36<br>(SPECIFY)<br>OTHER ______ 96<br>(SPECIFY)<br>(SKIP TO 434)
431	How long after (NAME) was delivered did you stay there?<br><br>IF LESS THAN ONE DAY, RECORD HOURS;<br>IF LESS THAN ONE WEEK, RECORD DAYS.	HOURS . . . . 1 [ ][ ]<br>DAYS . . . . 2 [ ][ ]<br>WEEKS . . . . 3 [ ][ ]<br>DON'T KNOW 998	
431A	Was (NAME) discharged at the same time as you?	YES . . . . 1<br>(SKIP TO 432)<br>NO 2	

NO.	QUESTIONS AND FILTERS	LAST BIRTH<br>NAME ______	NEXT-TO-LAST BIRTH<br>NAME ______
431B	How long after (NAME) was delivered did (NAME) stay at the facility?<br><br>IF LESS THAN ONE DAY, RECORD HOURS;<br>IF LESS THAN ONE WEEK, RECORD DAYS.	HOURS . . . . 1 [ ][ ]<br>DAYS . . . . 2 [ ][ ]<br>WEEKS . . . . 3 [ ][ ]<br>DON'T KNOW 998	
432	Was (NAME) delivered by caesarean, that is, did they cut your belly open to take the baby out?	YES . . . . 1<br>NO . . . . 2<br>(SKIP TO 434)	YES . . . . 1<br>NO . . . . 2<br>(SKIP TO 434)
433	When was the decision made to have the caesarean section? Was it before or after your labor pains started?	BEFORE . . . . 1<br>AFTER 2	BEFORE . . . . 1<br>AFTER 2
434	Immediately after the birth, was (NAME) put on your chest?	YES . . . . 1<br>NO . . . . 2<br>(SKIP TO 434B)<br>DON'T KNOW 8	YES . . . . 1<br>NO . . . . 2<br>(SKIP TO 434B)<br>DON'T KNOW 8
434A	Was (NAME)'s bare skin touching your bare skin?	YES . . . . 1<br>NO . . . . 2<br>DON'T KNOW 8	YES . . . . 1<br>NO . . . . 2<br>DON'T KNOW 8
434B	CHECK 430: PLACE OF DELIVERY	CODE 11, 12, OR 96 CIRCLED [ ] (SKIP TO 449)<br>OTHER [ ]	
435	I would like to talk to you about checks on your health after delivery, for example, someone asking you questions about your health or examining you. Did anyone check on your health while you were still in the facility?	YES . . . . 1<br>NO . . . . 2<br>(SKIP TO 438)	
436	How long after delivery did the first check take place?<br><br>IF LESS THAN ONE DAY, RECORD HOURS;<br>IF LESS THAN ONE WEEK, RECORD DAYS.	HOURS . . . . 1 [ ][ ]<br>DAYS . . . . 2 [ ][ ]<br>WEEKS . . . . 3 [ ][ ]<br>DON'T KNOW 998	
437	Who checked on your health at that time?<br><br>PROBE FOR MOST QUALIFIED PERSON.	**HEALTH PERSONNEL**<br>DOCTOR/GYNAECOLOGIST 11<br>NURSE/MIDWIFE . . . . 12<br><br>**OTHER PERSON**<br>TRADITIONAL BIRTH ATTENDANT . . . . 21<br>COMMUNITY HEALTH WORKER . . . . 22<br><br>OTHER ______ 96<br>(SPECIFY)	

NO.	QUESTIONS AND FILTERS	LAST BIRTH<br>NAME ______	NEXT-TO-LAST BIRTH<br>NAME ______
438	Now I would like to talk to you about checks on (NAME)'s health after delivery – for example, someone examining (NAME), checking the cord, or seeing if (NAME) is OK. Did anyone check on (NAME)'s health while you were still in the facility?	YES . . . . . 1<br>NO . . . . . 2 (SKIP TO 441)<br>DON'T KNOW . . . . . 8 (SKIP TO 441)	
439	How long after delivery was (NAME)'s health first checked?<br><br>IF LESS THAN ONE DAY, RECORD HOURS;<br>IF LESS THAN ONE WEEK, RECORD DAYS.	HOURS . . . . . 1 [ ][ ]<br>DAYS . . . . . 2 [ ][ ]<br>WEEKS . . . . . 3 [ ][ ]<br>DON'T KNOW 998	
440	Who checked on (NAME)'s health at that time?<br><br>PROBE FOR MOST QUALIFIED PERSON.	**HEALTH PERSONNEL**<br>DOCTOR/PAEDIATRICIAN 11<br>NURSE/MIDWIFE . . . . . 12<br><br>**OTHER PERSON**<br>TRADITIONAL BIRTH ATTENDANT . . . . . 21<br>COMMUNITY HEALTH WORKER . . . . . 22<br><br>OTHER ______ 96<br>(SPECIFY)	
441	Now I want to talk to you about what happened after you left the facility. Did anyone check on your health after you left the facility?	YES . . . . . 1<br>NO . . . . . 2 (SKIP TO 445)	
442	How long after delivery did that check take place?<br><br>IF LESS THAN ONE DAY, RECORD HOURS;<br>IF LESS THAN ONE WEEK, RECORD DAYS.	HOURS . . . . . 1 [ ][ ]<br>DAYS . . . . . 2 [ ][ ]<br>WEEKS . . . . . 3 [ ][ ]<br>DON'T KNOW 998	
443	Who checked on your health at that time?<br><br>PROBE FOR MOST QUALIFIED PERSON.	**HEALTH PERSONNEL**<br>DOCTOR/GYNAECOLOGIST 11<br>NURSE/MIDWIFE . . . . . 12<br><br>**OTHER PERSON**<br>TRADITIONAL BIRTH ATTENDANT . . . . . 21<br>COMMUNITY HEALTH WORKER . . . . . 22<br><br>OTHER ______ 96<br>(SPECIFY)	

NO.	QUESTIONS AND FILTERS	LAST BIRTH<br>NAME ______	NEXT-TO-LAST BIRTH<br>NAME ______
444	Where did the check take place?<br><br>PROBE TO IDENTIFY THE TYPE OF SOURCE.<br><br>IF UNABLE TO DETERMINE IF PUBLIC OR PRIVATE SECTOR, RECORD 96 AND WRITE THE NAME OF THE PLACE.	**HOME**<br>HER HOME . . . . 11<br>OTHER HOME . . . . 12<br><br>**PUBLIC SECTOR**<br>GOVERNMENT HOSPITAL . . 21<br>GOVERNMENT CLINIC/<br>COMM. HEALTH CENTRE 22<br>MOBILE CLINIC . . . . 23<br>OTHER PUBLIC SECTOR<br>______ 26<br>(SPECIFY)<br><br>**PRIVATE MEDICAL SECTOR**<br>PRIVATE HOSPITAL/DOCTOR 31<br>OTHER PRIVATE<br>MEDICAL SECTOR<br>______ 36<br>(SPECIFY)<br><br>OTHER ______ 96<br>(SPECIFY)	
445	I would like to talk to you about checks on (NAME)'s health after you left (FACILITY IN 430). Did any health care provider check on (NAME)'s health in the two months after you left (FACILITY IN 430)?	YES . . . . 1<br>NO . . . . 2<br>(SKIP TO 457)<br>DON'T KNOW 8	
446	How many hours, days or weeks after the birth of (NAME) did that check take place?<br><br>IF LESS THAN ONE DAY, RECORD HOURS;<br>IF LESS THAN ONE WEEK, RECORD DAYS.	HOURS . . . . 1 [ ][ ]<br>DAYS . . . . 2 [ ][ ]<br>WEEKS . . . . 3 [ ][ ]<br><br>DON'T KNOW 998	
447	Who checked on (NAME)'s health at that time?<br><br>PROBE FOR MOST QUALIFIED PERSON.	**HEALTH PERSONNEL**<br>DOCTOR/PAEDIATRICIAN 11<br>NURSE/MIDWIFE . . . . 12<br><br>**OTHER PERSON**<br>TRADITIONAL BIRTH<br>ATTENDANT . . . . 21<br>COMMUNITY HEALTH<br>WORKER . . . . 22<br><br>OTHER ______ 96<br>(SPECIFY)	

NO.	QUESTIONS AND FILTERS	LAST BIRTH<br>NAME ________	NEXT-TO-LAST BIRTH<br>NAME ________
448	Where did this check of (NAME) take place?<br><br>PROBE TO IDENTIFY THE TYPE OF SOURCE.<br><br>IF UNABLE TO DETERMINE IF PUBLIC OR PRIVATE SECTOR, RECORD 96 AND WRITE THE NAME OF THE PLACE.	**HOME**<br>HER HOME . . . . 11<br>OTHER HOME . . . . 12<br><br>**PUBLIC SECTOR**<br>GOVERNMENT HOSPITAL . . 21<br>GOVERNMENT CLINIC/ COMM. HEALTH CENTRE 22<br>MOBILE CLINIC . . . . 23<br>OTHER PUBLIC SECTOR ________ 26<br>(SPECIFY)<br><br>**PRIVATE MEDICAL SECTOR**<br>PRIVATE HOSPITAL/DOCTOR 31<br>OTHER PRIVATE MEDICAL SECTOR ________ 36<br>(SPECIFY)<br><br>OTHER ________ 96<br>(SPECIFY)<br><br>(SKIP TO 457)	
449	I would like to talk to you about checks on your health after delivery, for example, someone asking you questions about your health or examining you. Did anyone check on your health after you gave birth to (NAME)?	YES . . . . 1<br>NO . . . . 2<br>(SKIP TO 453)	
450	How long after delivery did the first check take place?<br><br>IF LESS THAN ONE DAY, RECORD HOURS;<br>IF LESS THAN ONE WEEK, RECORD DAYS.	HOURS . . . . 1 [ ][ ]<br>DAYS . . . . 2 [ ][ ]<br>WEEKS . . . . 3 [ ][ ]<br>DON'T KNOW 998	
451	Who checked on your health at that time?<br><br>PROBE FOR MOST QUALIFIED PERSON.	**HEALTH PERSONNEL**<br>DOCTOR/GYNAECOLOGIST 11<br>NURSE/MIDWIFE . . . . 12<br><br>**OTHER PERSON**<br>TRADITIONAL BIRTH ATTENDANT . . . . 21<br>COMMUNITY HEALTH WORKER . . . . 22<br><br>OTHER ________ 96<br>(SPECIFY)	

NO.	QUESTIONS AND FILTERS	LAST BIRTH<br>NAME ______	NEXT-TO-LAST BIRTH<br>NAME ______
452	Where did this first check take place?<br><br>PROBE TO IDENTIFY THE TYPE OF SOURCE.<br><br>IF UNABLE TO DETERMINE IF PUBLIC OR PRIVATE SECTOR, RECORD 96 AND WRITE THE NAME OF THE PLACE.	**HOME**<br>HER HOME . . . . 11<br>OTHER HOME . . . . 12<br><br>**PUBLIC SECTOR**<br>GOVERNMENT HOSPITAL . . 21<br>GOVERNMENT CLINIC/<br>COMM. HEALTH CENTRE 22<br>MOBILE CLINIC . . . . 23<br>OTHER PUBLIC SECTOR<br>______ 26<br>(SPECIFY)<br><br>**PRIVATE MEDICAL SECTOR**<br>PRIVATE HOSPITAL/DOCTOR 31<br>OTHER PRIVATE<br>MEDICAL SECTOR<br>______ 36<br>(SPECIFY)<br><br>OTHER ______ 96<br>(SPECIFY)	
453	I would like to talk to you about checks on (NAME)'s health after delivery – for example, someone examining (NAME), checking the cord, or seeing if (NAME) is OK. In the two months after (NAME) was born, did any health care provider or a traditional birth attendant check on (NAME)'s health?	YES . . . . 1<br>NO . . . . 2<br>(SKIP TO 457)<br>DON'T KNOW 8	
454	How many hours, days or weeks after the birth of (NAME) did the first check take place?<br><br>IF LESS THAN ONE DAY, RECORD HOURS;<br>IF LESS THAN ONE WEEK, RECORD DAYS.	HOURS AFTER BIRTH . . . . . 1 [ ][ ]<br>DAYS AFTER BIRTH . . . . . 2 [ ][ ]<br>WEEKS AFTER BIRTH . . . . . 3 [ ][ ]<br><br>DON'T KNOW 998	
455	Who checked on (NAME)'s health at that time?<br><br>PROBE FOR MOST QUALIFIED PERSON.	**HEALTH PERSONNEL**<br>DOCTOR/PAEDIATRICIAN 11<br>NURSE/MIDWIFE . . . . 12<br><br>**OTHER PERSON**<br>TRADITIONAL BIRTH<br>ATTENDANT . . . . 21<br>COMMUNITY HEALTH<br>WORKER . . . . 22<br><br>OTHER ______ 96<br>(SPECIFY)	

NO.	QUESTIONS AND FILTERS	LAST BIRTH<br>NAME ______	NEXT-TO-LAST BIRTH<br>NAME ______
456	Where did this first check of (NAME) take place?<br><br>PROBE TO IDENTIFY THE TYPE OF SOURCE.<br><br>IF UNABLE TO DETERMINE IF PUBLIC OR PRIVATE SECTOR, RECORD 96 AND WRITE THE NAME OF THE PLACE.	**HOME**<br>HER HOME . . . . 11<br>OTHER HOME . . . . 12<br><br>**PUBLIC SECTOR**<br>GOVERNMENT HOSPITAL . . 21<br>GOVERNMENT CLINIC/ COMM. HEALTH CENTRE 22<br>MOBILE CLINIC . . . . 23<br>OTHER PUBLIC SECTOR<br>______ 26<br>(SPECIFY)<br><br>**PRIVATE MEDICAL SECTOR**<br>PRIVATE HOSPITAL/DOCTOR 31<br>OTHER PRIVATE MEDICAL SECTOR<br>______ 36<br>(SPECIFY)<br><br>OTHER ______ 96<br>(SPECIFY)	
457	During the first two days after (NAME)'s birth, did any health care provider do the following:<br>a) Examine the cord?<br>b) Measure (NAME)'s temperature?<br>c) Counsel you on danger signs for newborns?<br>d) Counsel you on breastfeeding?<br>e) Observe (NAME) breastfeeding?	YES NO DK<br>a) CORD . . . . 1 2 8<br>b) TEMP. . . . . . 1 2 8<br>c) SIGNS . . . . . 1 2 8<br>d) COUNSEL BREAST-FEED 1 2 8<br>e) OBSERVE BREAST-FEED 1 2 8	
458	Has your menstrual period returned since the birth of (NAME)?	YES . . . . 1 (SKIP TO 460)<br>NO . . . . 2 (SKIP TO 461)	
459	Did your period return between the birth of (NAME) and your next pregnancy?		YES . . . . 1<br>NO . . . . 2 (SKIP TO 463)
460	For how many months after the birth of (NAME) did you not have a period?	MONTHS . . . . [ ][ ]<br><br>DON'T KNOW 98	MONTHS . . . . [ ][ ]<br><br>DON'T KNOW 98
461	CHECK 226: IS RESPONDENT PREGNANT?	NOT PREGNANT [ ]<br>PREGNANT OR UNSURE [ ] (SKIP TO 463)	

SECTION 4. PREGNANCY AND POSTNATAL CARE

NO.	QUESTIONS AND FILTERS	LAST BIRTH<br>NAME ______	NEXT-TO-LAST BIRTH<br>NAME ______
462	Have you had sexual intercourse since the birth of (NAME)?	YES . . . . . 1<br>NO . . . . . 2 (SKIP TO 464)	
463	For how many months after the birth of (NAME) did you not have sexual intercourse?	MONTHS . . . . . [ ][ ]<br>DON'T KNOW 98	MONTHS . . . . . [ ][ ]<br>DON'T KNOW 98
464	Did you ever breastfeed (NAME)?	YES . . . . . 1 (SKIP TO 466)<br>NO 2	YES . . . . . 1<br>NO 2
465	CHECK 404: IS CHILD LIVING?	LIVING [ ] (SKIP TO 470)<br>DEAD [ ] (SKIP TO 471)	
466	How long after birth did you first put (NAME) to the breast for feeding?<br><br>IF LESS THAN 1 HOUR, RECORD '00' HOURS;<br>IF LESS THAN 24 HOURS, RECORD HOURS;<br>OTHERWISE, RECORD DAYS.	IMMEDIATELY . . . . . 000<br>HOURS . . . . . 1 [ ][ ]<br>DAYS . . . . . 2 [ ][ ]	
467	In the first three days after delivery, was (NAME) given anything to drink other than breast milk?	YES . . . . . 1<br>NO 2	
468	CHECK 404: IS CHILD LIVING?	LIVING [ ] ↓<br>DEAD [ ] (SKIP TO 471)	LIVING [ ] ↓<br>DEAD [ ] (SKIP TO 471)
469	Are you still breastfeeding (NAME)?	YES . . . . . 1 (SKIP TO 470)<br>NO 2	
469A	For how many months did you breastfeed (NAME)?	MONTHS . . . . . [ ][ ]<br>DON'T KNOW 98	
470	Did (NAME) drink anything from a bottle with a teat yesterday or last night?	YES . . . . . 1<br>NO . . . . . 2<br>DON'T KNOW 8	YES . . . . . 1<br>NO . . . . . 2<br>DON'T KNOW 8
471		GO BACK TO 405 IN NEXT COLUMN; OR, IF NO MORE BIRTHS, GO TO 501A.	GO BACK TO 405 IN NEXT-TO-LAST COLUMN OF NEW QUESTIONNAIRE; OR, IF NO MORE BIRTHS, GO TO 501A.

SECTION 5A. CHILD IMMUNISATION (LAST BIRTH)

NO.	QUESTIONS AND FILTERS	CODING CATEGORIES	SKIP
501A	CHECK 215 IN THE BIRTH HISTORY: ANY BIRTHS IN 2013-2016?<br>ONE OR MORE BIRTHS IN 2013-2016 ☐	NO BIRTHS IN 2013-2016 ☐	→ 601
502A	RECORD THE NAME AND BIRTH HISTORY NUMBER FROM 212 OF THE LAST CHILD BORN IN 2013-2016.<br>NAME OF LAST BIRTH ______________________	BIRTH HISTORY NUMBER . . . . ☐☐	
503A	CHECK 216 FOR CHILD:<br>LIVING ☐	DEAD ☐	→ 501B
504A	Do you have a Road to Health booklet/card or other document where (NAME)'s vaccinations are written down?	YES, HAS ONLY A BOOKLET . . . . 1<br>YES, HAS ONLY ANOTHER DOCUMENT . . . . . 2<br>YES, HAS BOOKLET AND OTHER DOCUMENT 3<br>NO, NO BOOKLET AND NO OTHER DOCUMENT 4	1 → 507A<br>3 → 507A
505A	Did you ever have a Road to Health booklet for (NAME)?	YES . . . . 1<br>NO 2	2 → 505A2
505A1	What happened to (NAME)'s Road to Health booklet?	BOOKLET WITH RELATIVE . . . . 1<br>BOOKLET MISPLACED OR LOST . . . . 2<br>BOOKLET STOLEN . . . . 3<br>BOOKLET HELD AS COLLATERAL/RANSOM . . 4<br>BOOKLET DESTROYED . . . . 5<br>OTHER ______________________ 6<br>(SPECIFY)	→ 506A
505A2	Why don't you have a Road to Health booklet for (NAME)?	NONE AVAILABLE AT HEALTH FACILITY . . . . . 1<br>FOREIGNERS NOT GIVEN ONE . . . . 2<br>REQUIRED TO PAY FOR IT . . . . 3<br>TOO BUSY TO GET ONE . . . . 4<br>OTHER ______________________ 6<br>(SPECIFY)	
506A	CHECK 504A:<br>CODE '2' CIRCLED ☐	CODE '4' CIRCLED ☐	→ 511A
506A1	May I see the document where (NAME)'s vaccinations are written down?	YES, OTHER DOCUMENT SEEN . . . . 1<br>NO DOCUMENT SEEN 2	1 → 508A<br>2 → 511A
507A	May I see the Road to Health booklet or other document where (NAME)'s vaccinations are written down?	YES, ONLY BOOKLET SEEN . . . . 1<br>YES, ONLY OTHER DOCUMENT SEEN . . . . . 2<br>YES, BOOKLET AND OTHER DOCUMENT SEEN 3<br>NO BOOKLET AND NO OTHER DOCUMENT SEEN 4	1 → 508A<br>3 → 508A
507A1	Where is (NAME)'s Road to Health booklet?	BOOKLET WITH RELATIVE . . . . 1<br>BOOKLET MISPLACED OR LOST . . . . 2<br>BOOKLET STOLEN . . . . 3<br>BOOKLET HELD AS COLLATERAL/RANSOM . . 4<br>BOOKLET AT HEALTH FACILITY . . . . 5<br>OTHER ______________________ 6<br>(SPECIFY)	
507A2	CHECK 507A:<br>CODE '2' CIRCLED ☐	CODE '4' CIRCLED ☐	→ 511A

508A PHOTOGRAPH VACCINATION PAGE OF BOOKLET OR OTHER DOCUMENT WHERE VACCINATIONS ARE WRITTEN.
COPY DATES FROM THE BOOKLET.
WRITE '44' IN 'DAY' COLUMN IF BOOKLET SHOWS THAT A DOSE WAS GIVEN, BUT NO DATE IS RECORDED.

	DAY		MONTH		YEAR			
BCG								
ORAL POLIO VACCINE (OPV) 0 (BIRTH DOSE)								
ORAL POLIO VACCINE (OPV) 1								
ROTAVIRUS (RV) 1								
DTAP-IPV-HIB 1								
HEPATITIS B (HEP B) 1								
PNEUMOCOCCAL CONJUGATE VACCINE (PCV) 1								
DTAP-IPV-HIB 2								
HEPATITIS B (HEP B) 2								
DTAP-IPV-HIB 3								
HEPATITIS B (HEP B) 3								
PNEUMOCOCCAL CONJUGATE VACCINE (PCV) 2								
ROTAVIRUS (RV) 2								
MEASLES 1								
PNEUMOCOCCAL CONJUGATE VACCINE (PCV) 3								
DTAP-IPV-HIB 4								
MEASLES 2								
VITAMIN A (MOST RECENT)								

509A CHECK 508A: 'BCG' TO 'MEASLES 2' ALL RECORDED?

NO ☐ YES ☐ → 525A

510A	In addition to what is recorded on (this document/these documents), did (NAME) receive any other vaccinations, including vaccinations received in immunisation campaigns?<br><br>RECORD 'YES' ONLY IF THE RESPONDENT MENTIONS AT LEAST ONE OF THE VACCINATIONS IN 508A THAT ARE NOT RECORDED AS HAVING BEEN GIVEN.	YES . . . . 1<br>(PROBE FOR VACCINATIONS AND WRITE '66' IN THE CORRESPONDING DAY COLUMN IN 508A THEN WRITE '00' IN THE CORRESPONDING DAY COLUMN FOR ALL VACCINATIONS NOT GIVEN)<br>(THEN SKIP TO 525A)<br><br>NO . . . . 2<br>DON'T KNOW . . . . 8<br>(WRITE '00' IN THE CORRESPONDING DAY COLUMN FOR ALL VACCINATIONS NOT GIVEN)<br>(THEN SKIP TO 525A)	

SECTION 5A. CHILD IMMUNISATION (LAST BIRTH)

NO.	QUESTIONS AND FILTERS	CODING CATEGORIES	SKIP
	NAME OF LAST BIRTH ____________	BIRTH HISTORY NUMBER . . . . [ ][ ]	
511A	Did (NAME) ever receive any vaccinations to prevent (NAME) from getting diseases, including vaccinations received in immunisation campaigns?	YES . . . 1<br>NO . . . 2<br>DON'T KNOW . . . 8	 2 → 526A<br>8 → 501B
512A	Has (NAME) ever received a BCG vaccination against tuberculosis, that is, an injection in the arm or shoulder that usually causes a scar?	YES . . . 1<br>NO . . . 2<br>DON'T KNOW . . . 8	
514A	Has (NAME) ever received oral polio vaccine, that is, about two drops in the mouth to prevent polio?	YES . . . 1<br>NO . . . 2<br>DON'T KNOW . . . 8	2, 8 → 517A
515A	Did (NAME) receive the first oral polio vaccine in the first two weeks after birth or later?	FIRST TWO WEEKS . . . 1<br>LATER . . . 2	
516A	How many times did (NAME) receive the oral polio vaccine?	NUMBER OF TIMES . . . [ ]	
517A	Has (NAME) ever received a DTP-combination vaccination, also known as a pentavalent vaccination? That is, an injection given in the left thigh or left arm to prevent diphtheria, tetanus, and whooping cough?	YES . . . 1<br>NO . . . 2<br>DON'T KNOW . . . 8	2, 8 → 518A1
518A	How many times did (NAME) receive the DTP-combination vaccine?	NUMBER OF TIMES . . . [ ]	
518A1	Has (NAME) ever received a hepatitis B vaccination, that is, an injection given in the right thigh to prevent hepatitis B?	YES . . . 1<br>NO . . . 2<br>DON'T KNOW . . . 8	2, 8 → 519A
518A2	How many times did (NAME) receive the hepatitis B vaccine?	NUMBER OF TIMES . . . [ ]	
519A	Has (NAME) ever received a pneumococcal vaccination, that is, an injection in the right thigh to prevent pneumonia?	YES . . . 1<br>NO . . . 2<br>DON'T KNOW . . . 8	2, 8 → 521A
520A	How many times did (NAME) receive the pneumococcal vaccine?	NUMBER OF TIMES . . . [ ]	
521A	Has (NAME) ever received a rotavirus vaccination, that is, syrup in the mouth to prevent diarrhoea?	YES . . . 1<br>NO . . . 2<br>DON'T KNOW . . . 8	2, 8 → 523A
522A	How many times did (NAME) receive the rotavirus vaccine?	NUMBER OF TIMES . . . [ ]	
523A	Has (NAME) ever received a measles vaccination, that is, an injection in the left thigh or right arm to prevent measles?	YES . . . 1<br>NO . . . 2<br>DON'T KNOW . . . 8	2, 8 → 525A
524A	How many times did (NAME) receive the measles vaccine?	NUMBER OF TIMES . . . [ ]	
525A	Did (NAME) ever miss getting a vaccination or get a vaccination late?	YES . . . 1<br>NO . . . 2<br>DON'T KNOW . . . 8	2, 8 → 501B
526A	CHECK 508A AND 511A:<br>CHILD RECEIVED AT LEAST ONE VACCINATION [ ] ↓ / CHILD RECEIVED RECEIVED NO VACCINATIONS [ ] ↓<br>a) What was the reason for (NAME) missing the vaccination or getting it late? PROBE: Any other reason?<br>b) What is the reason (NAME) has not received any vaccinations? PROBE: Any other reason?	CLINIC OUT OF STOCK . . . A<br>NOT AWARE OF NEED FOR A VACCINATION B<br>FEAR OF SIDE EFFECTS . . . C<br>DID NOT KNOW WHERE TO GO . . . D<br>TOO BUSY TO TAKE CHILD . . . E<br>NO MONEY FOR TRANSPORT . . . F<br>CHILD WAS ILL . . . G<br>RESPONDENT WAS ILL . . . H<br>OTHER ____________ X<br>(SPECIFY)<br>DON'T KNOW . . . Z	

SECTION 5B. CHILD IMMUNISATION (NEXT-TO-LAST BIRTH)

NO.	QUESTIONS AND FILTERS	CODING CATEGORIES	SKIP
501B	CHECK 215 IN THE BIRTH HISTORY: ANY MORE BIRTHS IN 2013-2016?<br>MORE BIRTHS IN 2013-2016 ☐ ↓ NO MORE BIRTHS IN 2013-2016 ☐		→ 601
502B	RECORD THE NAME AND BIRTH HISTORY NUMBER FROM 212 OF THE NEXT-TO-LAST CHILD BORN IN 2013-2016.<br>NAME OF NEXT-TO-LAST BIRTH ______________	BIRTH HISTORY NUMBER . . . . [ ][ ]	
503B	CHECK 216 FOR CHILD:<br>LIVING ☐ ↓ DEAD ☐		→ 527B
504B	Do you have a Road to Health booklet/card or other document where (NAME)'s vaccinations are written down?	YES, HAS ONLY A BOOKLET . . . . 1<br>YES, HAS ONLY ANOTHER DOCUMENT . . . . . 2<br>YES, HAS BOOKLET AND OTHER DOCUMENT 3<br>NO, NO BOOKLET AND NO OTHER DOCUMENT 4	1 → 507B<br>3 → 507B
505B	Did you ever have a Road to Health booklet for (NAME)?	YES . . . . 1<br>NO 2	2 → 505B2
505B1	What happened to (NAME)'s Road to Health booklet?	BOOKLET WITH RELATIVE . . . . 1<br>BOOKLET MISPLACED OR LOST . . . . 2<br>BOOKLET STOLEN . . . . 3<br>BOOKLET HELD AS COLLATERAL/RANSOM . . 4<br>BOOKLET DESTROYED . . . . 5<br>OTHER ______________ 6<br>(SPECIFY)	1-6 → 506B
505B2	Why don't you have a Road to Health booklet for (NAME)?	NONE AVAILABLE AT HEALTH FACILITY . . . . . 1<br>FOREIGNERS NOT GIVEN ONE . . . . 2<br>REQUIRED TO PAY FOR IT . . . . 3<br>TOO BUSY TO GET ONE . . . . 4<br>OTHER ______________ 6<br>(SPECIFY)	
506B	CHECK 504B:<br>CODE '2' CIRCLED ☐ ↓ CODE '4' CIRCLED ☐		→ 511B
506B1	May I see the document where (NAME)'s vaccinations are written down?	YES, OTHER DOCUMENT SEEN . . . . 1<br>NO DOCUMENT SEEN 2	1 → 508B<br>2 → 511B
507B	May I see the Road to Health booklet or other document where (NAME)'s vaccinations are written down?	YES, ONLY BOOKLET SEEN . . . . 1<br>YES, ONLY OTHER DOCUMENT SEEN . . . . . 2<br>YES, BOOKLET AND OTHER DOCUMENT SEEN 3<br>NO BOOKLET AND NO OTHER DOCUMENT SEEN 4	1 → 508B<br>3 → 508B
507B1	Where is (NAME)'s Road to Health booklet?	BOOKLET WITH RELATIVE . . . . 1<br>BOOKLET MISPLACED OR LOST . . . . 2<br>BOOKLET STOLEN . . . . 3<br>BOOKLET HELD AS COLLATERAL/RANSOM . . 4<br>BOOKLET AT HEALTH FACILITY . . . . 5<br>OTHER ______________ 6<br>(SPECIFY)	
507B2	CHECK 507B:<br>CODE '2' CIRCLED ☐ ↓ CODE '4' CIRCLED ☐		→ 511B

508B PHOTOGRAPH VACCINATION PAGE OF BOOKLET OR OTHER DOCUMENT WHERE VACCINATIONS ARE WRITTEN.
COPY DATES FROM THE BOOKLET.
WRITE '44' IN 'DAY' COLUMN IF BOOKLET SHOWS THAT A DOSE WAS GIVEN, BUT NO DATE IS RECORDED.

	DAY		MONTH		YEAR			
BCG								
ORAL POLIO VACCINE (OPV) 0 (BIRTH DOSE)								
ORAL POLIO VACCINE (OPV) 1								
ROTAVIRUS (RV) 1								
DTAP-IPV-HIB 1								
HEPATITIS B (HEP B) 1								
PNEUMOCOCCAL CONJUGATE VACCINE (PCV) 1								
DTAP-IPV-HIB 2								
HEPATITIS B (HEP B) 2								
DTAP-IPV-HIB 3								
HEPATITIS B (HEP B) 3								
PNEUMOCOCCAL CONJUGATE VACCINE (PCV) 2								
ROTAVIRUS (RV) 2								
MEASLES 1								
PNEUMOCOCCAL CONJUGATE VACCINE (PCV) 3								
DTAP-IPV-HIB 4								
MEASLES 2								
VITAMIN A (MOST RECENT)								

509B CHECK 508B: 'BCG' TO 'MEASLES 2' ALL RECORDED?

NO ☐ ↓ YES ☐ → 525B

510B	In addition to what is recorded on (this document/these documents), did (NAME) receive any other vaccinations, including vaccinations received in immunisation campaigns?<br><br>RECORD 'YES' ONLY IF THE RESPONDENT MENTIONS AT LEAST ONE OF THE VACCINATIONS IN 508B THAT ARE NOT RECORDED AS HAVING BEEN GIVEN.	YES . . . . 1<br>(PROBE FOR VACCINATIONS AND WRITE '66' IN THE CORRESPONDING DAY COLUMN IN 508B THEN WRITE '00' IN THE CORRESPONDING DAY COLUMN FOR ALL VACCINATIONS NOT GIVEN)<br>(THEN SKIP TO 525B)<br><br>NO . . . . 2<br>DON'T KNOW . . . . 8<br>(WRITE '00' IN THE CORRESPONDING DAY COLUMN FOR ALL VACCINATIONS NOT GIVEN)<br>(THEN SKIP TO 525B)	

SECTION 5B. CHILD IMMUNISATION (NEXT-TO-LAST BIRTH)

NO.	QUESTIONS AND FILTERS	CODING CATEGORIES	SKIP
	NAME OF NEXT-TO-LAST BIRTH ________________	BIRTH HISTORY NUMBER . . . . [ ][ ]	
511B	Did (NAME) ever receive any vaccinations to prevent (NAME) from getting diseases, including vaccinations received in immunisation campaigns?	YES . . . . . 1<br>NO . . . . . 2<br>DON'T KNOW 8	 2 → 526B<br>8 → 527B
512B	Has (NAME) ever received a BCG vaccination against tuberculosis, that is, an injection in the arm or shoulder that usually causes a scar?	YES . . . . . 1<br>NO . . . . . 2<br>DON'T KNOW 8	
514B	Has (NAME) ever received oral polio vaccine, that is, about two drops in the mouth to prevent polio?	YES . . . . . 1<br>NO . . . . . 2<br>DON'T KNOW 8	2, 8 → 517B
515B	Did (NAME) receive the first oral polio vaccine in the first two weeks after birth or later?	FIRST TWO WEEKS . . . . . 1<br>LATER 2	
516B	How many times did (NAME) receive the oral polio vaccine?	NUMBER OF TIMES . . . . . [ ]	
517B	Has (NAME) ever received a DTP-combination vaccination, also known as a pentavalent vaccination? That is, an injection given in the left thigh or left arm to prevent diphtheria, tetanus, and whooping cough?	YES . . . . . 1<br>NO . . . . . 2<br>DON'T KNOW 8	2, 8 → 518B1
518B	How many times did (NAME) receive the DTP-combination vaccine?	NUMBER OF TIMES . . . . . [ ]	
518B1	Has (NAME) ever received a hepatitis B vaccination, that is, an injection given in the right thigh to prevent hepatitis B?	YES . . . . . 1<br>NO . . . . . 2<br>DON'T KNOW 8	2, 8 → 519B
518B2	How many times did (NAME) receive the hepatitis B vaccine?	NUMBER OF TIMES . . . . . [ ]	
519B	Has (NAME) ever received a pneumococcal vaccination, that is, an injection in the right thigh to prevent pneumonia?	YES . . . . . 1<br>NO . . . . . 2<br>DON'T KNOW 8	2, 8 → 521B
520B	How many times did (NAME) receive the pneumococcal vaccine?	NUMBER OF TIMES . . . . . [ ]	
521B	Has (NAME) ever received a rotavirus vaccination, that is, syrup in the mouth to prevent diarrhoea?	YES . . . . . 1<br>NO . . . . . 2<br>DON'T KNOW 8	2, 8 → 523B
522B	How many times did (NAME) receive the rotavirus vaccine?	NUMBER OF TIMES . . . . . [ ]	
523B	Has (NAME) ever received a measles vaccination, that is, an injection in the left thigh or right arm to prevent measles?	YES . . . . . 1<br>NO . . . . . 2<br>DON'T KNOW 8	2, 8 → 525B
524B	How many times did (NAME) receive the measles vaccine?	NUMBER OF TIMES . . . . . [ ]	

SECTION 5B. CHILD IMMUNISATION (NEXT-TO-LAST BIRTH)

NO.	QUESTIONS AND FILTERS	CODING CATEGORIES	SKIP
	NAME OF NEXT-TO-LAST BIRTH ____________	BIRTH HISTORY NUMBER . . . . [ ][ ]	
525B	Did (NAME) ever miss getting a vaccination or get a vaccination late?	YES . . . . . 1<br>NO . . . . . 2<br>DON'T KNOW 8	2, 8 → 526B
526B	CHECK 508B AND 511B:<br>CHILD RECEIVED AT LEAST ONE VACCINATION [ ] ↓ a) What was the reason for (NAME) missing the vaccination or getting it late? PROBE: Any other reason?<br>CHILD RECEIVED RECEIVED NO VACCINATIONS [ ] ↓ b) What is the reason (NAME) has not received any vaccinations? PROBE: Any other reason?	CLINIC OUT OF STOCK . . . . . A<br>NOT AWARE OF NEED FOR A VACCINATION B<br>FEAR OF SIDE EFFECTS . . . . . C<br>DID NOT KNOW WHERE TO GO . . . . . D<br>TOO BUSY TO TAKE CHILD . . . . . E<br>NO MONEY FOR TRANSPORT . . . . . F<br>CHILD WAS ILL . . . . . G<br>RESPONDENT WAS ILL . . . . . H<br>OTHER ____________ (SPECIFY) X<br>DON'T KNOW . . . . . Z	
527B	CHECK 215 IN BIRTH HISTORY: ANY MORE BIRTHS IN 2013-2016?<br>MORE BIRTHS IN 2013-2016 [ ] (GO TO 502B IN AN ADDITIONAL QUESTIONNAIRE)<br>NO MORE BIRTHS IN 2013-2016 [ ]		NO MORE BIRTHS → 601

SECTION 6. CHILD HEALTH AND NUTRITION

601	CHECK 224:<br>ONE OR MORE BIRTHS IN 2011-2016 ☐ ↓<br>NO BIRTHS IN 2011-2016 ☐ → 648		
602	CHECK 215: RECORD THE BIRTH HISTORY NUMBER IN 603 AND THE NAME AND SURVIVAL STATUS IN 604 FOR EACH BIRTH IN 2011-2016. ASK THE QUESTIONS ABOUT ALL OF THESE BIRTHS. BEGIN WITH THE LAST BIRTH. IF THERE ARE MORE THAN 2 BIRTHS, USE LAST COLUMN OF ADDITIONAL QUESTIONNAIRE(S).<br><br>Now I would like to ask some questions about your children born in the last five years. (We will talk about each separately.)		
603	BIRTH HISTORY NUMBER FROM 212 IN BIRTH HISTORY.	LAST BIRTH<br>BIRTH HISTORY NUMBER . . . . ☐☐	NEXT-TO-LAST BIRTH<br>BIRTH HISTORY NUMBER . . . . ☐☐
604	FROM 212 AND 216:	NAME ______<br>LIVING ☐ ↓ DEAD ☐ (SKIP TO 646)	NAME ______<br>LIVING ☐ ↓ DEAD ☐ (SKIP TO 646)
605	In the last six months, was (NAME) given a vitamin A dose like [this/any of these]?<br><br>SHOW COMMON TYPES OF AMPULES/CAPSULES/SYRUPS.	YES . . . . 1<br>NO . . . . 2<br>DON'T KNOW 8	YES . . . . 1<br>NO . . . . 2<br>DON'T KNOW 8
607	Was (NAME) given any drug for intestinal worms in the last six months?<br><br>IF RESPONDENT SAYS NO, CHECK ROAD TO HEALTH BOOKLET.	YES . . . . 1<br>NO . . . . 2<br>DON'T KNOW 8	YES . . . . 1<br>NO . . . . 2<br>DON'T KNOW 8
608	Has (NAME) had diarrhoea/loose stools in the last 2 weeks?	YES . . . . 1<br>NO . . . . 2<br>(SKIP TO 618)<br>DON'T KNOW 8	YES . . . . 1<br>NO . . . . 2<br>(SKIP TO 618)<br>DON'T KNOW 8
609	CHECK 469: CURRENTLY BREASTFEEDING?<br><br>YES ☐ ↓<br>a) Now I would like to know how much (NAME) was given to drink during the diarrhoea including breastmilk. Was (NAME) given less than usual to drink, about the same amount, or more than usual to drink?<br>IF LESS, PROBE: Was (NAME) given much less than usual to drink or somewhat less?<br><br>NO ☐ ↓<br>b) Now I would like to know how much (NAME) was given to drink during the diarrhoea. Was (NAME) given less than usual to drink, about the same amount, or more than usual to drink?<br>IF LESS, PROBE: Was (NAME) given much less than usual to drink or somewhat less?	MUCH LESS . . . . . . 1<br>SOMEWHAT LESS . . . . 2<br>ABOUT THE SAME . . . . 3<br>MORE . . . . 4<br>NOTHING TO DRINK . . . 5<br>DON'T KNOW 8	MUCH LESS . . . . . . 1<br>SOMEWHAT LESS . . . . 2<br>ABOUT THE SAME . . . . 3<br>MORE . . . . 4<br>NOTHING TO DRINK . . . 5<br>DON'T KNOW 8

SECTION 6. CHILD HEALTH AND NUTRITION

NO.	QUESTIONS AND FILTERS	LAST BIRTH<br>NAME ________	NEXT-TO-LAST BIRTH<br>NAME ________
610	When (NAME) had diarrhoea, was (NAME) given less than usual to eat, about the same amount, more than usual, or nothing to eat?<br><br>IF LESS, PROBE: Was (NAME) given much less than usual to eat or somewhat less?	MUCH LESS . . . . 1<br>SOMEWHAT LESS . . . . 2<br>ABOUT THE SAME . . . . 3<br>MORE . . . . 4<br>STOPPED FOOD . . . . 5<br>NEVER GAVE FOOD . . . . 6<br>DON'T KNOW 8	MUCH LESS . . . . 1<br>SOMEWHAT LESS . . . . 2<br>ABOUT THE SAME . . . . 3<br>MORE . . . . 4<br>STOPPED FOOD . . . . 5<br>NEVER GAVE FOOD . . . . 6<br>DON'T KNOW 8
611	Did you seek advice or treatment for the diarrhoea from any source?	YES . . . . 1<br>NO . . . . 2<br>(SKIP TO 615)	YES . . . . 1<br>NO . . . . 2<br>(SKIP TO 615)
612	Where did you seek advice or treatment?<br><br>Anywhere else?<br><br>PROBE TO IDENTIFY THE TYPE OF SOURCE.<br><br>IF UNABLE TO DETERMINE IF PUBLIC OR PRIVATE SECTOR, RECORD 'X' AND WRITE THE NAME OF THE PLACE(S).	**PUBLIC SECTOR**<br>GOVERNMENT HOSPITAL . . A<br>GOVERNMENT CLINIC/<br>COMM. HEALTH CENTRE B<br>MOBILE CLINIC . . . . C<br>COMM. HEALTH WORKER . . D<br>OTHER PUBLIC SECTOR<br>________ E<br>(SPECIFY)<br><br>**PRIVATE MEDICAL SECTOR**<br>PRIVATE HOSPITAL/CLINIC F<br>CHEMIST/PHARMACY . . . . . G<br>PRIVATE DOCTOR . . . . H<br>OTHER PRIVATE<br>MEDICAL SECTOR<br>________ I<br>(SPECIFY)<br><br>**OTHER SOURCE**<br>SUPERMARKET/SHOP . . . . . J<br>TRADITIONAL<br>HEALER . . . . K<br>MARKET . . . . L<br><br>OTHER ________ X<br>(SPECIFY)	**PUBLIC SECTOR**<br>GOVERNMENT HOSPITAL . . A<br>GOVERNMENT CLINIC/<br>COMM. HEALTH CENTRE B<br>MOBILE CLINIC . . . . C<br>COMM. HEALTH WORKER . . D<br>OTHER PUBLIC SECTOR<br>________ E<br>(SPECIFY)<br><br>**PRIVATE MEDICAL SECTOR**<br>PRIVATE HOSPITAL/CLINIC F<br>CHEMIST/PHARMACY . . . . . G<br>PRIVATE DOCTOR . . . . H<br>OTHER PRIVATE<br>MEDICAL SECTOR<br>________ I<br>(SPECIFY)<br><br>**OTHER SOURCE**<br>SUPERMARKET/SHOP . . . . . J<br>TRADITIONAL<br>HEALER . . . . K<br>MARKET . . . . L<br><br>OTHER ________ X<br>(SPECIFY)
613	CHECK 612:	TWO OR MORE CODES CIRCLED ☐<br>ONLY ONE CODE CIRCLED ☐ (SKIP TO 615)	TWO OR MORE CODES CIRCLED ☐<br>ONLY ONE CODE CIRCLED ☐ (SKIP TO 615)
614	Where did you first seek advice or treatment?<br><br>USE LETTER CODE FROM 612.	FIRST PLACE . . . . ☐	FIRST PLACE . . . . ☐

NO.	QUESTIONS AND FILTERS	LAST BIRTH<br>NAME ______	NEXT-TO-LAST BIRTH<br>NAME ______
615	Was (NAME) given any of the following at any time since (NAME) started having the diarrhoea:<br>a) A fluid made from a special packet called Sorol or Rehidrat?<br>b) A clinic-recommended sugar-salt solution?<br>c) Zinc tablets or syrup?	YES NO DK<br>a) FLUID FROM ORS PACKET . . 1 2 8<br>b) HOMEMADE FLUID . . . . . 1 2 8<br>c) ZINC 1 2 8	YES NO DK<br>a) FLUID FROM ORS PACKET . . 1 2 8<br>b) HOMEMADE FLUID . . . . . 1 2 8<br>c) ZINC 1 2 8
616	CHECK 615:<br>ANY 'YES' ↓ a) Was anything else given to treat the diarrhoea?<br>ALL 'NO' OR 'DK' ↓ b) Was anything given to treat the diarrhoea?	YES . . . . . 1<br>NO . . . . . 2<br>(SKIP TO 618)<br>DON'T KNOW 8	YES . . . . . 1<br>NO . . . . . 2<br>(SKIP TO 618)<br>DON'T KNOW 8
617	CHECK 615:<br>ANY 'YES' ↓ a) What else was given to treat the diarrhoea? Anything else?<br>ALL 'NO' OR 'DK' ↓ b) What was given to treat the diarrhoea? Anything else?<br>RECORD ALL TREATMENTS GIVEN.	**PILL OR SYRUP**<br>ANTIBIOTIC . . . . . A<br>ANTIMOTILITY . . . . . B<br>OTHER (NOT ANTIBIOTIC OR ANTIMOTILITY) . . . . . C<br>UNKNOWN PILL OR SYRUP . . . . . D<br>**INJECTION**<br>ANTIBIOTIC . . . . . E<br>NON-ANTIBIOTIC . . . . . F<br>UNKNOWN INJECTION . . . . . G<br>(IV) INTRAVENOUS . . . . . H<br>HOME REMEDY/ HERBAL MEDICINE . . . . . I<br>OTHER ______ X<br>(SPECIFY)	**PILL OR SYRUP**<br>ANTIBIOTIC . . . . . A<br>ANTIMOTILITY . . . . . B<br>OTHER (NOT ANTIBIOTIC OR ANTIMOTILITY) . . . . . C<br>UNKNOWN PILL OR SYRUP . . . . . D<br>**INJECTION**<br>ANTIBIOTIC . . . . . E<br>NON-ANTIBIOTIC . . . . . F<br>UNKNOWN INJECTION . . . . . G<br>(IV) INTRAVENOUS . . . . . H<br>HOME REMEDY/ HERBAL MEDICINE . . . . . I<br>OTHER ______ X<br>(SPECIFY)
618	Has (NAME) been ill with a fever at any time in the last 2 weeks?	YES . . . . . 1<br>NO . . . . . 2<br>DON'T KNOW 8	YES . . . . . 1<br>NO . . . . . 2<br>DON'T KNOW 8
620	Has (NAME) had an illness with a cough at any time in the last 2 weeks?	YES . . . . . 1<br>NO . . . . . 2<br>DON'T KNOW 8	YES . . . . . 1<br>NO . . . . . 2<br>DON'T KNOW 8
621	Has (NAME) had fast, short, rapid breaths or difficulty breathing at any time in the last 2 weeks?	YES . . . . . 1<br>NO . . . . . 2<br>(SKIP TO 623)<br>DON'T KNOW 8	YES . . . . . 1<br>NO . . . . . 2<br>(SKIP TO 623)<br>DON'T KNOW 8
622	Was the fast or difficult breathing due to a problem in the chest or to a blocked or runny nose?	CHEST ONLY . . . . . 1<br>NOSE ONLY . . . . . 2<br>BOTH . . . . . 3<br>OTHER ______ 6<br>(SPECIFY)<br>DON'T KNOW . . . . . 8<br>(SKIP TO 624)	CHEST ONLY . . . . . 1<br>NOSE ONLY . . . . . 2<br>BOTH . . . . . 3<br>OTHER ______ 6<br>(SPECIFY)<br>DON'T KNOW . . . . . 8<br>(SKIP TO 624)
623	CHECK 618: HAD FEVER?	YES ↓ NO OR DK (SKIP TO 646)	YES ↓ NO OR DK (SKIP TO 646)

NO.	QUESTIONS AND FILTERS	LAST BIRTH<br>NAME ______	NEXT-TO-LAST BIRTH<br>NAME ______
624	Did you seek advice or treatment for the illness from any source?	YES . . . . . 1<br>NO . . . . . 2 (SKIP TO 629)	YES . . . . . 1<br>NO . . . . . 2 (SKIP TO 629)
625	Where did you seek advice or treatment?<br><br>Anywhere else?<br><br>PROBE TO IDENTIFY THE TYPE OF SOURCE.<br><br>IF UNABLE TO DETERMINE IF PUBLIC OR PRIVATE SECTOR, RECORD 'X' AND WRITE THE NAME OF THE PLACE(S).	**PUBLIC SECTOR**<br>GOVERNMENT HOSPITAL . . A<br>GOVERNMENT CLINIC/ COMM. HEALTH CENTRE B<br>MOBILE CLINIC . . . . . C<br>COMM. HEALTH WORKER . . D<br>OTHER PUBLIC SECTOR ______ E (SPECIFY)<br>**PRIVATE MEDICAL SECTOR**<br>PRIVATE HOSPITAL/CLINIC F<br>CHEMIST/PHARMACY . . . . . G<br>PRIVATE DOCTOR . . . . . H<br>OTHER PRIVATE MEDICAL SECTOR ______ I (SPECIFY)<br>**OTHER SOURCE**<br>SUPERMARKET/SHOP . . . . . J<br>TRADITIONAL HEALER . . . . . K<br>MARKET . . . . . L<br>OTHER ______ X (SPECIFY)	**PUBLIC SECTOR**<br>GOVERNMENT HOSPITAL . . A<br>GOVERNMENT CLINIC/ COMM. HEALTH CENTRE B<br>MOBILE CLINIC . . . . . C<br>COMM. HEALTH WORKER . . D<br>OTHER PUBLIC SECTOR ______ E (SPECIFY)<br>**PRIVATE MEDICAL SECTOR**<br>PRIVATE HOSPITAL/CLINIC F<br>CHEMIST/PHARMACY . . . . . G<br>PRIVATE DOCTOR . . . . . H<br>OTHER PRIVATE MEDICAL SECTOR ______ I (SPECIFY)<br>**OTHER SOURCE**<br>SUPERMARKET/SHOP . . . . . J<br>TRADITIONAL HEALER . . . . . K<br>MARKET . . . . . L<br>OTHER ______ X (SPECIFY)
626	CHECK 625:	TWO OR MORE CODES CIRCLED ☐<br>ONLY ONE CODE CIRCLED ☐ (SKIP TO 628)	TWO OR MORE CODES CIRCLED ☐<br>ONLY ONE CODE CIRCLED ☐ (SKIP TO 628)
627	Where did you first seek advice or treatment?<br><br>USE LETTER CODE FROM 625.	FIRST PLACE . . . . . ☐	FIRST PLACE . . . . . ☐
628	How many days after the illness began did you first seek advice or treatment for (NAME)?<br>IF THE SAME DAY RECORD '00'.	DAYS . . . . . ☐☐	DAYS . . . . . ☐☐
629	At any time during the illness, did (NAME) take any drugs for the illness?	YES . . . . . 1<br>NO . . . . . 2<br>DON'T KNOW . . . . . 8<br>(SKIP TO 646)	YES . . . . . 1<br>NO . . . . . 2<br>DON'T KNOW . . . . . 8<br>(SKIP TO 646)

NO.	QUESTIONS AND FILTERS	LAST BIRTH<br>NAME ____________	NEXT-TO-LAST BIRTH<br>NAME ____________
630	What drugs did (NAME) take?<br><br>Any other drugs?<br><br>RECORD ALL MENTIONED.	**ANTIMALARIAL DRUGS**<br>COARTEM/ARTEMISININ COMBINATION THERAPY (ACT) . . . . . A<br>OTHER ANTIMALARIAL<br>____________ B<br>(SPECIFY)<br><br>**ANTIBIOTIC DRUGS**<br>PILL/SYRUP . . . . C<br>INJECTION/IV . . . . D<br><br>**OTHER DRUGS**<br>ASPIRIN . . . . E<br>PARACETAMOL/PANADO . . F<br>BRUFEN . . . . G<br>PONSTAN . . . . H<br><br>OTHER ____________ X<br>(SPECIFY)<br>DON'T KNOW . . . . Z	**ANTIMALARIAL DRUGS**<br>COARTEM/ARTEMISININ COMBINATION THERAPY (ACT) . . . . . A<br>OTHER ANTIMALARIAL<br>____________ B<br>(SPECIFY)<br><br>**ANTIBIOTIC DRUGS**<br>PILL/SYRUP . . . . C<br>INJECTION/IV . . . . D<br><br>**OTHER DRUGS**<br>ASPIRIN . . . . E<br>PARACETAMOL/PANADO . . F<br>BRUFEN . . . . G<br>PONSTAN . . . . H<br><br>OTHER ____________ X<br>(SPECIFY)<br>DON'T KNOW . . . . Z
646		GO BACK TO 604 IN NEXT COLUMN; OR, IF NO MORE BIRTHS, GO TO 647.	GO TO 604 IN NEXT-TO-LAST COLUMN OF NEW QUESTIONNAIRE; OR, IF NO MORE BIRTHS, GO TO 647.

NO.	QUESTIONS AND FILTERS	CODING CATEGORIES	SKIP
647	CHECK 615(a), ALL COLUMNS:<br>NO CHILD RECEIVED FLUID FROM ORS PACKET ☐ ↓<br>ANY CHILD RECEIVED FLUID FROM ORS PACKET ☐ → 648B		648B
648	Have you ever heard of a special product called Sorol or Rehidrat that you can get for the treatment of diarrhoea?	YES . . . . 1<br>NO 2	
648A	CHECK 224:<br>ONE OR MORE BIRTHS IN 2011-2016 ☐ ↓<br>NO BIRTHS IN 2011-2016 ☐ → 648C		648C
648B	CHECK 615(b), ALL COLUMNS:<br>NO CHILD RECEIVED CLINIC RECOMMENDED SUGAR-SALT SOLUTION ☐ ↓<br>ANY CHILD RECEIVED CLINIC RECOMMENDED SUGAR-SALT SOLUTION ☐ → 649		649
648C	Have you ever heard from a health care worker about a sugar-salt solution that can be made at home for the treatment of diarrhoea?	YES . . . . 1<br>NO 2	
649	CHECK 215 AND 218, ALL ROWS: NUMBER OF CHILDREN BORN IN 2014-2016 LIVING WITH THE RESPONDENT<br>ONE OR MORE ☐ ↓<br>NONE ☐ → 701<br>________________<br>(NAME OF YOUNGEST CHILD LIVING WITH HER) ↓		701

NO.	QUESTIONS AND FILTERS	CODING CATEGORIES	SKIP
650	Now I would like to ask you about liquids or foods that (NAME FROM 649) had yesterday during the day or at night. I am interested in whether your child had the item I mention even if it was combined with other foods.<br>Did (NAME FROM 649) drink or eat:	YES NO DK	
	a) Plain water?	a) 1 2 8	
	b) Fruit juice or squashes?	b) 1 2 8	
	d) Milk such as tinned, powdered, or fresh animal milk?<br>IF YES: How many times did (NAME) drink milk?<br>IF 7 OR MORE TIMES, RECORD '7'.	d) . . . . 1 2 8<br>NUMBER OF TIMES DRANK MILK [ ]	
	e) Infant formula?<br>IF YES: How many times did (NAME) drink infant formula?<br>IF 7 OR MORE TIMES, RECORD '7'.	e) . . . . 1 2 8<br>NUMBER OF TIMES DRANK FORMULA [ ]	
	eb) Coke, Stoney, Dixi Cola, Jive or other sugary drinks?	eb) 1 2 8	
	f) Any other liquids?	f) 1 2 8	
	g) Yogurt, amasi, maas or custard?<br>IF YES: How many times did (NAME) eat yogurt, amasi, maas or custard?<br>IF 7 OR MORE TIMES, RECORD '7'.	g) . . . . 1 2 8<br>NUMBER OF TIMES ATE YOGURT [ ]	
	h) Any Purity, Cerelac, Ace or other commercially fortified baby cereal or porridge?	h) 1 2 8	
	i) Porridge, pap, bread, rice, noodles, Morvite or other foods made from grains?	i) 1 2 8	
	j) Pumpkin, carrots, squash, or sweet potatoes that are yellow or orange inside?	j) 1 2 8	
	k) White potatoes, white sweet potatoes, white yams, or any other foods made from roots?	k) 1 2 8	
	l) Any dark green, leafy vegetables?	l) 1 2 8	
	m) Ripe mangoes, ripe papayas, or orange melon?	m) 1 2 8	
	n) Any other fruits or vegetables such as oranges, apples, bananas, guava, green melon, pineapples, avocados, or mushrooms?	n) 1 2 8	
	o) Liver, kidney, heart, or other organ meats?	o) 1 2 8	
	p) Any meat, such as beef, pork, lamb, goat, chicken, or duck?	p) 1 2 8	
	q) Eggs?	q) 1 2 8	
	r) Fresh, dried or tinned fish or shellfish?	r) 1 2 8	
	s) Any foods made from beans, peas, lentils, or nuts?	s) 1 2 8	
	t) Cheese or other food made from milk?	t) 1 2 8	

NO.	QUESTIONS AND FILTERS	CODING CATEGORIES	SKIP
	u) Any oils, fats, butter, or foods made with any of these?	u) 1 2 8	
	v) Any sugary foods such as chocolates, sweets, candies, pastries, cakes or biscuits?	v) 1 2 8	
	va) Any salty snacks such as Nik Naks, Simba, Flings, or Spookies?	va) 1 2 8	
	w) Any other solid, semi-solid, or soft food?	w) 1 2 8	
651	CHECK 650 (CATEGORIES 'g' THROUGH 'w'):<br>NOT A SINGLE 'YES' ☐ AT LEAST ONE 'YES' ☐		653
652	Did (NAME FROM 649) eat any solid, semi-solid, or soft foods yesterday during the day or at night?<br><br>IF 'YES' PROBE: What kind of solid, semi-solid or soft foods did (NAME) eat?	YES . . . . 1<br>(GO BACK TO 650 TO RECORD FOOD EATEN YESTERDAY)<br>(THEN CONTINUE TO 653)<br><br>NO 2	 <br><br><br><br>653A
653	How many times did (NAME FROM 649) eat solid, semi-solid, or soft foods yesterday during the day or at night?<br><br>IF 7 OR MORE TIMES, RECORD '7'.	NUMBER OF TIMES . . . . ☐<br><br>DON'T KNOW 8	
653A	CHECK 215: CHILD AGE 6 MONTHS OR OLDER?<br>YES ☐ NO ☐		654
653B	Has (NAME FROM 649) ever eaten liver?	YES . . . . 1<br>NO 2	 654
653C	In the last four weeks, how many times has (NAME FROM 649) eaten liver?	NUMBER OF TIMES ☐☐<br><br>DON'T KNOW 98	
654	The last time (NAME FROM 649) passed stools, what was done to dispose of the stools?	CHILD USED TOILET OR LATRINE . . . . 01<br>PUT/RINSED<br>INTO TOILET OR LATRINE . . . . 02<br>PUT/RINSED<br>INTO DRAIN, DITCH, RIVER OR STREAM 03<br>THROWN INTO GARBAGE . . . . 04<br>BURIED . . . . 05<br>LEFT IN THE OPEN . . . . 06<br><br>OTHER ______________________ 96<br>(SPECIFY)	

SECTION 7. MARRIAGE AND SEXUAL ACTIVITY

NO.	QUESTIONS AND FILTERS	CODING CATEGORIES	SKIP
701	Are you currently married or living together with someone as if married?	YES, CURRENTLY MARRIED . . . 1<br>YES, LIVING WITH A PARTNER . . . 2<br>NO . . . 3	1, 2 → 701B
701A	Do you have a regular boyfriend/partner or fiancé?	YES . . . 1<br>NO . . . 2	2 → 702
701B	Is this person a man or a woman?	MAN . . . 1<br>WOMAN . . . 2<br>INTERSEX OR TRANSGENDERED . . . 3	
701C	CHECK 701: RESPONDENT'S CURRENT MARITAL STATUS<br>701 = 3 ☐ ↓<br>701 = 1 OR 2 ☐		701 = 1 OR 2 → 703A
702	Have you ever been married or lived together with someone as if married?	YES, FORMERLY MARRIED . . . 1<br>YES, LIVED WITH A PARTNER . . . 2<br>NO . . . 3	3 → 703A
703	What is your marital status now: are you widowed, divorced, or separated?	WIDOWED . . . 1<br>DIVORCED . . . 2<br>SEPARATED . . . 3	
703A	CHECK 106: AGE OF RESPONDENT<br>AGE 15-49 ☐ ↓<br>AGE 50 AND ABOVE ☐		AGE 50 AND ABOVE → 901
703B	CHECK 701 AND 702: EVER MARRIED OR LIVED WITH A PARTNER?<br>702 = 1 OR 2 ☐<br>701 = 1 OR 2 ☐ ↓<br>701 = 3 AND 702 = 3 ☐		702 = 1 OR 2 → 709<br>701 = 3 AND 702 = 3 → 713
704	Is your (spouse/partner) living with you now or is he/she staying elsewhere?	LIVING WITH HER . . . 1<br>STAYING ELSEWHERE . . . 2	
705	RECORD THE SPOUSE'S/PARTNER'S NAME AND LINE NUMBER FROM THE HOUSEHOLD QUESTIONNAIRE. IF HE/SHE IS NOT LISTED IN THE HOUSEHOLD, RECORD '00'.	NAME ________<br>LINE NO. . . . ☐☐	
705A	CHECK 701B: SEX OF SPOUSE/PARTNER<br>SPOUSE/PARTNER IS MALE (701B = 1) ☐ ↓<br>SPOUSE/PARTNER IS FEMALE OR INTERSEX (701B = 2 OR 3) ☐		FEMALE OR INTERSEX → 709
706	Does your (husband/partner) have other wives or does he live with other women as if married?	YES . . . 1<br>NO . . . 2<br>DON'T KNOW . . . 8	2, 8 → 709
707	Including yourself, in total, how many wives or live-in partners does he have?	TOTAL NUMBER OF WIVES AND LIVE-IN PARTNERS . . . ☐☐<br>DON'T KNOW . . . 98	
708	Are you the first, second, … wife?	RANK . . . ☐☐	

SECTION 7. MARRIAGE AND SEXUAL ACTIVITY

NO.	QUESTIONS AND FILTERS	CODING CATEGORIES	SKIP
709	Have you been married or lived with someone only once or more than once?	ONLY ONCE . . . . . 1<br>MORE THAN ONCE 2	
710	CHECK 709:<br>MARRIED/LIVED WITH A PARTNER ONLY ONCE ☐ / MARRIED/ LIVED WITH A PARTNER MORE THAN ONCE ☐<br>a) In what month and year did you start living with your (spouse/partner)?<br>b) Now I would like to ask about your first (spouse/partner). In what month and year did you start living with your first (spouse/partner)?	MONTH . . . . . ☐☐<br>DON'T KNOW MONTH . . . . . 98<br>YEAR . . . . . ☐☐☐☐<br>DON'T KNOW YEAR 9998	→712
711	How old were you when you first started living together?	AGE . . . . . ☐☐	
712	**CHECK FOR PRESENCE OF OTHERS. BEFORE CONTINUING, MAKE EVERY EFFORT TO ENSURE PRIVACY.**		
713	Now I would like to ask some questions about sexual activity in order to gain a better understanding of some important life issues. Let me assure you again that your answers are completely confidential and will not be told to anyone. If we should come to any question that you don't want to answer, just let me know and we will go to the next question. How old were you when you had sexual intercourse for the very first time?	NEVER HAD SEXUAL INTERCOURSE . . . . . 00<br>AGE IN YEARS . . . . . ☐☐	00 →731
714	I would like to ask you about your recent sexual activity. When was the last time you had sexual intercourse?<br>IF LESS THAN 12 MONTHS, ANSWER MUST BE RECORDED IN DAYS, WEEKS OR MONTHS. IF 12 MONTHS (ONE YEAR) OR MORE, ANSWER MUST BE RECORDED IN YEARS.	DAYS AGO . . . . . 1 ☐☐<br>WEEKS AGO . . . . . 2 ☐☐<br>MONTHS AGO . . . . . 3 ☐☐<br>YEARS AGO . . . . . 4 ☐☐	1–3 →716<br>4 →727

		LAST SEXUAL PARTNER	SECOND-TO-LAST SEXUAL PARTNER	THIRD-TO-LAST SEXUAL PARTNER
715	When was the last time you had sexual intercourse with this person?		DAYS AGO . . 1 [ ][ ]<br>WEEKS AGO . . 2 [ ][ ]<br>MONTHS AGO . . 3 [ ][ ]	DAYS AGO . . 1 [ ][ ]<br>WEEKS AGO . . 2 [ ][ ]<br>MONTHS AGO . . 3 [ ][ ]
716	The last time you had sexual intercourse with this person, was a condom used?	YES . . . . 1<br>NO . . . . 2<br>(SKIP TO 718)	YES . . . . 1<br>NO . . . . 2<br>(SKIP TO 718)	YES . . . . 1<br>NO . . . . 2<br>(SKIP TO 718)
717	Was a condom used every time you had sexual intercourse with this person in the last 12 months?	YES . . . . 1<br>NO 2	YES . . . . 1<br>NO 2	YES . . . . 1<br>NO 2
718	What was your relationship to this person with whom you had sexual intercourse?<br><br>IF BOYFRIEND/GIRLFRIEND: Were you living together as if married?<br><br>IF YES, RECORD '2'.<br>IF NO, RECORD '3'.	SPOUSE . . . . 1<br>LIVE-IN PARTNER . . . . . 2<br>BOYFRIEND/GIRLFRIEND NOT LIVING WITH RESPONDENT . . . . . 3<br>CASUAL ACQUAINTANCE . . 4<br>CLIENT/SEX WORKER . . 5<br>OTHER ______ 6<br>(SPECIFY)	SPOUSE . . . . 1<br>LIVE-IN PARTNER . . . . . 2<br>BOYFRIEND/GIRLFRIEND NOT LIVING WITH RESPONDENT . . . . . 3<br>CASUAL ACQUAINTANCE . . 4<br>CLIENT/SEX WORKER . . 5<br>OTHER ______ 6<br>(SPECIFY)	SPOUSE . . . . 1<br>LIVE-IN PARTNER . . . . . 2<br>BOYFRIEND/GIRLFRIEND NOT LIVING WITH RESPONDENT . . . . . 3<br>CASUAL ACQUAINTANCE . . 4<br>CLIENT/SEX WORKER . . 5<br>OTHER ______ 6<br>(SPECIFY)
719	How long ago did you first have sexual intercourse with this person?	DAYS AGO . . 1 [ ][ ]<br>WEEKS AGO . . 2 [ ][ ]<br>MONTHS AGO . . 3 [ ][ ]<br>YEARS AGO . . 4 [ ][ ]	DAYS AGO . . 1 [ ][ ]<br>WEEKS AGO . . 2 [ ][ ]<br>MONTHS AGO . . 3 [ ][ ]<br>YEARS AGO . . 4 [ ][ ]	DAYS AGO . . 1 [ ][ ]<br>WEEKS AGO . . 2 [ ][ ]<br>MONTHS AGO . . 3 [ ][ ]<br>YEARS AGO . . 4 [ ][ ]
720	How many times during the last 12 months did you have sexual intercourse with this person?<br><br>IF NON-NUMERIC ANSWER, PROBE TO GET AN ESTIMATE. IF NUMBER OF TIMES IS 95 OR MORE, RECORD '95'.	NUMBER OF TIMES . . . . . [ ][ ]	NUMBER OF TIMES . . . . . [ ][ ]	NUMBER OF TIMES . . . . . [ ][ ]
721	How old is this person?	AGE OF PARTNER [ ][ ]<br><br>DON'T KNOW 98	AGE OF PARTNER [ ][ ]<br><br>DON'T KNOW 98	AGE OF PARTNER [ ][ ]<br><br>DON'T KNOW 98
722	Apart from this person, have you had sexual intercourse with any other person in the last 12 months?	YES . . . . 1<br>(GO BACK TO 715 IN NEXT COLUMN)<br>NO . . . . 2<br>(SKIP TO 724)	YES . . . . 1<br>(GO BACK TO 715 IN NEXT COLUMN)<br>NO . . . . 2<br>(SKIP TO 724)	
723	In total, with how many different people have you had sexual intercourse in the last 12 months?<br>IF NON-NUMERIC ANSWER, PROBE TO GET AN ESTIMATE. IF NUMBER OF PARTNERS IS 95 OR MORE, RECORD '95'.			NUMBER OF PARTNERS LAST 12 MONTHS . . [ ][ ]<br><br>DON'T KNOW 98

SECTION 7. MARRIAGE AND SEXUAL ACTIVITY

NO.	QUESTIONS AND FILTERS	CODING CATEGORIES	SKIP
724	CHECK 106:<br>AGE 15-24 ☐ (↓) AGE 25-49 ☐		→ 727 (AGE 25-49)
725	CHECK 701:<br>NOT CURRENTLY MARRIED/ LIVING WITH A SPOUSE ☐ (↓) CURRENTLY MARRIED/ LIVING WITH A SPOUSE ☐		→ 727 (CURRENTLY MARRIED/ LIVING WITH A SPOUSE)
726	In the past 12 months have you had sex or been sexually involved with anyone because he gave you or told you he would give you gifts, cash, or anything else?	YES . . . . . 1<br>NO 2	
727	In total, with how many different people have you had sexual intercourse in your lifetime?<br><br>IF NON-NUMERIC ANSWER, PROBE TO GET AN ESTIMATE. IF NUMBER OF PARTNERS IS 95 OR MORE, RECORD '95'.	NUMBER OF PARTNERS IN LIFETIME . . . . . [ ][ ]<br><br>DON'T KNOW 98	
731	PRESENCE OF OTHERS DURING THIS SECTION.	YES NO<br>CHILDREN <10 . . . . . 1 2<br>MALE ADULTS . . . . . 1 2<br>FEMALE ADULTS 1 2	

SECTION 8. FERTILITY PREFERENCES

NO.	QUESTIONS AND FILTERS	CODING CATEGORIES	SKIP
801	CHECK 304:<br>NEITHER STERILISED ☐ ↓	HE OR SHE STERILISED ☐ →	813
802	CHECK 226:<br>PREGNANT ☐ ↓	NOT PREGNANT OR UNSURE ☐ →	804
803	Now I have some questions about the future. After the child you are expecting now, would you like to have another child, or would you prefer not to have any more children?	HAVE ANOTHER CHILD . . . . 1<br>NO MORE . . . . 2<br>UNDECIDED/DON'T KNOW 8	1 → 805<br>2, 8 → 812
804	Now I have some questions about the future. Would you like to have (a/another) child, or would you prefer not to have any (more) children?	HAVE (A/ANOTHER) CHILD . . . . 1<br>NO MORE/NONE . . . . 2<br>SAYS SHE CAN'T GET PREGNANT . . . . 3<br>UNDECIDED/DON'T KNOW 8	 → 807<br>→ 813<br>→ 811
805	CHECK 226:<br>NOT PREGNANT OR UNSURE ☐ ↓ ¦ PREGNANT ☐ ↓<br>a) How long would you like to wait from now before the birth of (a/another) child?<br>b) After the birth of the child you are expecting now, how long would you like to wait before the birth of another child?	MONTHS . . . . 1 [ ][ ]<br>YEARS . . . . 2 [ ][ ]<br>SOON/NOW . . . . 993<br>SAYS SHE CAN'T GET PREGNANT . . . . .994<br>AFTER MARRIAGE . . . . 995<br>OTHER ______________ 996<br>(SPECIFY)<br>DON'T KNOW 998	 <br>993 → 811<br>994 → 813<br>995, 996, 998 → 811
806	CHECK 226:<br>NOT PREGNANT OR UNSURE ☐ ↓	PREGNANT ☐ →	812
807	CHECK 303: USING A CONTRACEPTIVE METHOD?<br>NOT CURRENTLY USING ☐ ↓	CURRENTLY USING ☐ →	813
808	CHECK 805:<br>'24' OR MORE MONTHS OR '02' OR MORE YEARS ☐ ↓ NOT ASKED ☐ ↓	'00-23' MONTHS OR '00-01' YEAR ☐ →	812
809	CHECK 714:<br>DAYS, WEEKS OR MONTHS AGO ☐ ↓	YEARS AGO ☐ →<br>NOT ASKED ☐ →	811<br>811

SECTION 8. FERTILITY PREFERENCES

NO.	QUESTIONS AND FILTERS	CODING CATEGORIES	SKIP
810	CHECK 804:<br>WANTS TO HAVE A/ANOTHER CHILD ☐ ↓ | WANTS NO MORE/ NONE ☐ ↓<br>a) You have said that you do not want (a/another) child soon. Can you tell me why you are not using a method to prevent pregnancy?<br>Any other reason?<br>b) You have said that you do not want any (more) children. Can you tell me why you are not using a method to prevent pregnancy?<br>Any other reason?<br>RECORD ALL REASONS MENTIONED.	NOT MARRIED . . . A<br>**FERTILITY-RELATED REASONS**<br>NOT HAVING SEX . . . B<br>INFREQUENT SEX . . . C<br>MENOPAUSAL/HYSTERECTOMY . . . D<br>CAN'T GET PREGNANT . . . E<br>NOT MENSTRUATED SINCE LAST BIRTH . . . F<br>BREASTFEEDING . . . G<br>UP TO GOD/FATALISTIC . . . H<br>**OPPOSITION TO USE**<br>RESPONDENT OPPOSED . . . I<br>HUSBAND/PARTNER OPPOSED . . . J<br>OTHERS OPPOSED . . . K<br>RELIGIOUS PROHIBITION . . . L<br>**LACK OF KNOWLEDGE**<br>KNOWS NO METHOD . . . M<br>KNOWS NO SOURCE . . . N<br>**METHOD-RELATED REASONS**<br>SIDE EFFECTS/HEALTH CONCERNS . . . O<br>LACK OF ACCESS/TOO FAR . . . P<br>COSTS TOO MUCH . . . Q<br>PREFERRED METHOD NOT AVAILABLE . . . R<br>NO METHOD AVAILABLE . . . S<br>INCONVENIENT TO USE . . . T<br>INTERFERES WITH BODY'S NORMAL PROCESSES . . . U<br>**OTHER**<br>PARTNER IS A WOMAN . . . V<br>OTHER ______ (SPECIFY) X<br>DON'T KNOW . . . Z	
811	CHECK 303: USING A CONTRACEPTIVE METHOD?<br>NOT ASKED ☐ ↓ | NO, NOT CURRENTLY USING ☐ ↓ | YES, CURRENTLY USING ☐ → 813		→ 813
812	Do you think you will use a contraceptive method to delay or avoid pregnancy at any time in the future?	YES . . . 1<br>NO . . . 2<br>DON'T KNOW . . . 8	
813	CHECK 216:<br>HAS LIVING CHILDREN ☐ ↓ | NO LIVING CHILDREN ☐ ↓<br>a) If you could go back to the time you did not have any children and could choose exactly the number of children to have in your whole life, how many would that be?<br>b) If you could choose exactly the number of children to have in your whole life, how many would that be?<br>PROBE FOR A NUMERIC RESPONSE.	NONE . . . 00<br>NUMBER . . . [ ][ ]<br>OTHER ______ (SPECIFY) 96	00 → 815<br>96 → 815

SECTION 8. FERTILITY PREFERENCES

NO.	QUESTIONS AND FILTERS	CODING CATEGORIES	SKIP
814	How many of these children would you like to be boys, how many would you like to be girls and for how many would it not matter if it's a boy or a girl?	BOYS / GIRLS / EITHER<br>NUMBER . . [ ][ ] / [ ][ ] / [ ][ ]<br>OTHER ______ 96<br>(SPECIFY)	
815	In the last six months have you:<br>a) Heard about family planning on the radio?<br>b) Seen anything about family planning on the television?<br>c) Read about family planning in a newspaper or magazine?<br>d) Heard about family planning from a community health worker?	YES NO<br>a) RADIO . . . . 1 2<br>b) TELEVISION . . . . 1 2<br>c) NEWSPAPER OR MAGAZINE . . . . 1 2<br>d) COMMUNITY HEALTH WORKER . . . 1 2	
815A	CHECK Q18 IN HOUSEHOLD QUESTIONNAIRE:<br>YES, CURRENTLY ATTENDING SCHOOL [ ] ↓ NO, NOT CURRENTLY ATTENDING SCHOOL [ ] →		817
815	e) Heard about family planning at school?	YES NO<br>e) SCHOOL 1 2	
817	CHECK 701, 701A AND 701B:<br>YES, CURRENTLY MARRIED TO A MAN [ ] ↓ YES, LIVING WITH A MAN [ ] ↓ YES, HAS REGULAR MALE PARTNER/BOYFRIEND [ ] ↓ NO, NOT IN A UNION OR, IN UNION, BUT NOT WITH A MAN [ ] →		901
818	CHECK 303: USING A CONTRACEPTIVE METHOD?<br>CURRENTLY USING [ ] ↓ NOT CURRENTLY USING [ ] → 820<br>NOT ASKED [ ] → 822		820<br>822
819	Would you say that using contraception is mainly your decision, mainly your (husband's/partner's) decision, or did you both decide together?	MAINLY RESPONDENT . . . . 1<br>MAINLY HUSBAND/PARTNER . . . . . . 2<br>JOINT DECISION . . . . 3<br>OTHER ______ 6<br>(SPECIFY)	→ 821
820	Would you say that not using contraception is mainly your decision, mainly your (husband's/partner's) decision, or did you both decide together?	MAINLY RESPONDENT . . . . 1<br>MAINLY HUSBAND/PARTNER . . . . . . 2<br>JOINT DECISION . . . . 3<br>OTHER ______ 6<br>(SPECIFY)	
821	CHECK 304:<br>NEITHER ARE STERILISED [ ] ↓ HE OR SHE ARE STERILISED [ ] →		901
822	Does your (husband/partner) want the same number of children that you want, or does he want more or fewer than you want?	SAME NUMBER . . . . 1<br>MORE CHILDREN . . . . 2<br>FEWER CHILDREN . . . . 3<br>DON'T KNOW 8	

SECTION 9. SPOUSE'S BACKGROUND AND WOMAN'S WORK

NO.	QUESTIONS AND FILTERS	CODING CATEGORIES	SKIP
901	CHECK 701 AND 701A:<br>CURRENTLY MARRIED/ LIVING WITH SOMEONE OR HAS A REGULAR PARTNER/BOYFRIEND ☐ ↓	NOT IN UNION OR NO REGULAR PARTNER/BOYFRIEND ☐	→ 909
902	How old was your (spouse/partner) on his/her last birthday?	AGE IN COMPLETED YEARS . . . . ☐☐	
903	Did your (spouse/partner) ever attend an educational institution?	YES . . . . 1<br>NO 2	 → 906
904	What was the highest level he/she attended: primary, secondary, or higher than secondary?	PRIMARY . . . . 1<br>SECONDARY . . . . 2<br>HIGHER THAN SECONDARY . . . . 3<br>DON'T KNOW 8	 <br><br>→ 906
905	What was the highest grade or form he/she completed at that level?	**PRIMARY SCHOOL**<br>LESS THAN 1 YEAR COMPLETED . . . . 00<br>GRADE 1/SUB A/CLASS 1 . . . . 11<br>GRADE 2/SUB B/CLASS 2 . . . . 12<br>GRADE 3/STANDARD 1/ AET 1 (KHA RI GUDE, SANLI) . . . . 13<br>GRADE 4/STANDARD 2 . . . . 14<br>GRADE 5/STANDARD 3/AET 2 . . . . 15<br>GRADE 6 /STANDARD 4 . . . . 16<br>GRADE 7/STANDARD 5/AET 3 . . . . 17<br>**SECONDARY SCHOOL**<br>LESS THAN 1 YEAR COMPLETED . . . . 20<br>GRADE 8/STANDARD 6/FORM 1/NTC 1/ N1/NC (V) LEVEL 2 . . . . 21<br>GRADE 9/STANDARD 7/FORM 2/AET 4/NTC 2/ N2/NC (V) LEVEL 3 . . . . 22<br>GRADE 10/STANDARD 8/FORM 3/NTC 3/ N3/NC (V) LEVEL 4 . . . . 23<br>GRADE 11/STANDARD 9/FORM 4 . . . . 24<br>CERTIFICATE OR DIPLOMA WITH LESS THAN GRADE 12/STANDARD 10 COMPLETED . . 25<br>GRADE 12/STANDARD 10/FORM 5/MATRIC . . 26<br>N4/NTC4 . . . . 27<br>N5/NTC5 . . . . 28<br>N6/NTC6 . . . . 29<br>**HIGHER EDUCATION**<br>FURTHER STUDIES INCOMPLETE OR ONGOING . . . . 30<br>CERTIFICATE OR DIPLOMA WITH GRADE 12/ STANDARD 10 COMPLETED . . . . 31<br>HIGHER DIPLOMA (TECHNIKON/ U. OF TECHNOLOGY) . . . . 32<br>POST HIGHER DIPLOMA (TECHNIKON/ U. TECHNOLOGY MASTERS, DOCTORAL) 33<br>BACHELORS DEGREE/BACHELORS DEGREE AND POST GRADUATE DIPLOMA . . 34<br>HONOURS DEGREE . . . . 35<br>HIGHER DEGREE (MASTERS, DOCTORATE) 36<br>DON'T KNOW 98	
906	Has your (spouse/partner) done any work in the last 7 days?	YES . . . . 1<br>NO . . . . 2<br>DON'T KNOW 8	→ 908
907	Has your (spouse/partner) done any work in the last 12 months?	YES . . . . 1<br>NO . . . . 2<br>DON'T KNOW 8	 → 909
908	What is your (spouse's/partner's) occupation? That is, what kind of work does he/she mainly do?	__________<br>__________<br>☐☐☐☐	
909	Aside from your own housework, have you done any work in the last seven days?	YES . . . . 1<br>NO 2	→ 913

SECTION 9. SPOUSE'S BACKGROUND AND WOMAN'S WORK

NO.	QUESTIONS AND FILTERS	CODING CATEGORIES	SKIP
910	As you know, some women take up jobs for which they are paid in cash or kind. Others sell things, have a small business or work on the family farm or in the family business. In the last seven days, have you done any of these things or any other work?	YES . . . . 1<br>NO 2	1 → 913
911	Although you did not work in the last seven days, do you have any job or business from which you were absent for leave, illness, vacation, maternity leave, or any other such reason?	YES . . . . 1<br>NO 2	1 → 913
912	Have you done any work in the last 12 months?	YES . . . . 1<br>NO 2	2 → 913A
913	What is your occupation? That is, what kind of work do you mainly do?	______________<br>______________<br>[ ][ ][ ][ ]	
913A	CHECK 106: AGE OF RESPONDENT<br>AGE 15-49 [ ] ↓ AGE 50 AND ABOVE [ ] → 1202		AGE 50 AND ABOVE → 1202
913B	CHECK 909, 910, 911, AND 912: ANY YES?<br>YES [ ] ↓ NO [ ] → 917		NO → 917
914	Do you do this work for a member of your family, for someone else, or are you self-employed?	FOR FAMILY MEMBER . . . . 1<br>FOR SOMEONE ELSE . . . . 2<br>SELF-EMPLOYED 3	
915	Do you usually work throughout the year, or do you work seasonally, or only once in a while?	THROUGHOUT THE YEAR . . . . 1<br>SEASONALLY/PART OF THE YEAR . . . . 2<br>ONCE IN A WHILE 3	
916	Are you paid in cash or kind for this work or are you not paid at all?	CASH ONLY . . . . 1<br>CASH AND KIND . . . . 2<br>IN KIND ONLY . . . . 3<br>NOT PAID 4	
917	CHECK 701, 701A AND 701B:<br>CURRENTLY MARRIED/ LIVING WITH A MAN, OR HAS REGULAR MALE PARTNER/BOYFRIEND [ ] ↓ NOT IN UNION OR NOT IN UNION WITH A MAN [ ] → 925		NOT IN UNION → 925
918	CHECK 916:<br>CODE '1' OR '2' CIRCLED [ ] ↓ OTHER [ ] → 921		OTHER → 921
919	Who usually decides how the money you earn will be used: you, your (husband/partner), or you and your (husband/partner) jointly?	RESPONDENT . . . . 1<br>HUSBAND/PARTNER . . . . 2<br>RESPONDENT AND HUSBAND/PARTNER JOINTLY . . . . 3<br>OTHER ______ (SPECIFY) 6	
920	Would you say that the money that you earn is more than what your (husband/partner) earns, less than what he earns, or about the same?	MORE THAN HIM . . . . 1<br>LESS THAN HIM . . . . 2<br>ABOUT THE SAME . . . . 3<br>HUSBAND/PARTNER HAS NO EARNINGS . . . . 4<br>DON'T KNOW 8	4 → 922
921	Who usually decides how your (husband's/partner's) earnings will be used: you, your (husband/partner), or you and your (husband/partner) jointly?	RESPONDENT . . . . 1<br>HUSBAND/PARTNER . . . . 2<br>RESPONDENT AND HUSBAND/PARTNER JOINTLY . . . . 3<br>HUSBAND/PARTNER HAS NO EARNINGS . . . . 4<br>OTHER ______ (SPECIFY) 6	

NO.	QUESTIONS AND FILTERS	CODING CATEGORIES	SKIP
922	Who usually makes decisions about health care for yourself: you, your (husband/partner), you and your (husband/partner) jointly, or someone else?	RESPONDENT . . . 1<br>HUSBAND/PARTNER . . . 2<br>RESPONDENT AND HUSBAND/PARTNER JOINTLY . . . 3<br>SOMEONE ELSE . . . 4<br>OTHER . . . 6	
923	Who usually makes decisions about making major household purchases?	RESPONDENT . . . 1<br>HUSBAND/PARTNER . . . 2<br>RESPONDENT AND HUSBAND/PARTNER JOINTLY . . . 3<br>SOMEONE ELSE . . . 4<br>OTHER . . . 6	
924	Who usually makes decisions about visits to your family or relatives?	RESPONDENT . . . 1<br>HUSBAND/PARTNER . . . 2<br>RESPONDENT AND HUSBAND/PARTNER JOINTLY . . . 3<br>SOMEONE ELSE . . . 4<br>OTHER . . . 6	
925	Do you own this or any other house either alone or jointly with someone else?	ALONE ONLY . . . 1<br>JOINTLY ONLY . . . 2<br>BOTH ALONE AND JOINTLY . . . 3<br>DOES NOT OWN . . . 4	 <br><br>4 → 931
926	Do you have a title deed or documents for any house you own?	YES . . . 1<br>NO . . . 2<br>DON'T KNOW . . . 8	 2, 8 → 931
927	Is your name on the title deed or documents?	YES . . . 1<br>NO . . . 2<br>DON'T KNOW . . . 8	

931	PRESENCE OF OTHERS AT THIS POINT (PRESENT AND LISTENING, PRESENT BUT NOT LISTENING, OR NOT PRESENT)		PRES./ LISTEN.	PRES./ NOT LISTEN.	NOT PRES.
		CHILDREN < 10	1	2	3
		HUSBAND	1	2	3
		OTHER MALES	1	2	3
		OTHER FEMALES	1	2	3

932	In your opinion, is a husband justified in hitting or beating his wife in the following situations:		YES	NO	DK
	a) If she goes out without telling him?	a) GOES OUT	1	2	8
	b) If she neglects the children?	b) NEGLECTS CHILDREN	1	2	8
	c) If she argues with him?	c) ARGUES	1	2	8
	d) If she refuses to have sex with him?	d) REFUSES SEX	1	2	8
	e) If she burns the food?	e) BURNS FOOD	1	2	8

933	CHECK 217 AND 218:		
	ONE OR MORE CHILDREN LESS THAN AGE 18 LIVING WITH HER ↓	NO CHILDREN OR NO CHILDREN LESS THAN AGE 18 LIVING WITH HER	→ 1001

934	Now I would like to ask you questions about how you discipline or punish your (child/children). In the past 12 months, have you ever:		YES	NO
	a) Hit or slapped your (child/children) with your hand to punish or discipline the child?	a) HIT WITH HAND	1	2
	b) Hit or beat your (child/children) using a belt, spoon, stick, shoe or any other implement to punish or discipline the child?	b) HIT WITH IMPLEMENT	1	2

SECTION 10. HIV/AIDS

NO.	QUESTIONS AND FILTERS	CODING CATEGORIES	SKIP
1001	Now I would like to talk about something else. Have you ever heard of HIV or AIDS?	YES . . . 1<br>NO . . . 2	 → 1042
1008	Can HIV be transmitted from a mother to her baby:<br>a) During pregnancy?<br>b) During delivery?<br>c) By breastfeeding?	YES / NO / DK<br>a) DURING PREGNANCY . . 1 2 8<br>b) DURING DELIVERY . . . . . 1 2 8<br>c) BREASTFEEDING 1 2 8	
1009	CHECK 1008:<br>AT LEAST ONE 'YES' ☐ ↓	OTHER ☐	→ 1011
1010	Are there any special drugs that a doctor or a nurse can give to a woman infected with HIV to reduce the risk of transmission to the baby?	YES . . . 1<br>NO . . . 2<br>DON'T KNOW . . . 8	
1011	CHECK 208 AND 215:<br>LAST BIRTH IN 2014-2016 ☐ ↓	NO BIRTHS ☐<br>LAST BIRTH IN 2013 OR EARLIER ☐	→ 1027<br>→ 1027
1012	CHECK 408 FOR LAST BIRTH:<br>HAD ANTENATAL CARE ☐ ↓	NO ANTENATAL CARE ☐	→ 1020
1013	**CHECK FOR PRESENCE OF OTHERS. BEFORE CONTINUING, MAKE EVERY EFFORT TO ENSURE PRIVACY.**		
1014	During any of the antenatal visits for your last birth were you given any information about:<br>a) Babies getting HIV from their mother?<br>b) Things that you can do to prevent getting HIV?<br>c) Getting tested for HIV?	YES / NO / DK<br>a) HIV FROM MOTHER . . 1 2 8<br>b) THINGS TO DO . . . . 1 2 8<br>c) TESTED FOR HIV 1 2 8	
1015	Were you offered a test for HIV as part of your antenatal care?	YES . . . 1<br>NO . . . 2	
1016	I don't want to know the results, but were you tested for HIV as part of your antenatal care?	YES . . . 1<br>NO . . . 2	 → 1020

NO.	QUESTIONS AND FILTERS	CODING CATEGORIES	SKIP
1017	Where was the test done?<br><br>PROBE TO IDENTIFY THE TYPE OF SOURCE.<br><br>IF UNABLE TO DETERMINE IF PUBLIC OR PRIVATE SECTOR, RECORD 96 AND WRITE THE NAME OF THE PLACE.	**PUBLIC SECTOR**<br>GOVERNMENT HOSPITAL . . . . 11<br>GOVERNMENT CLINIC/COMMUNITY HEALTH CENTRE . . . . 12<br>MOBILE/TEMPORARY HCT SERVICES . . . . 13<br>OTHER PUBLIC SECTOR<br>______ 16<br>(SPECIFY)<br><br>**PRIVATE MEDICAL SECTOR**<br>PRIVATE HOSPITAL/CLINIC/ PRIVATE DOCTOR . . . . 21<br>NEW START TESTING SITE . . . . 22<br>CHEMIST/PHARMACY . . . . 23<br><br>OTHER PRIVATE MEDICAL SECTOR<br>______ 26<br>(SPECIFY)<br>**OTHER SOURCE**<br>HOME . . . . 31<br>WORKPLACE . . . . 32<br>CORRECTIONAL FACILITY . . . . 33<br><br>OTHER ______ 96<br>(SPECIFY)	
1018	I don't want to know the results, but did you get the results of the test?	YES . . . . 1<br>NO 2	 2 → 1020
1019	All women are supposed to receive counselling after being tested. After you were tested, did you receive counselling?	YES . . . . 1<br>NO . . . . 2<br>DON'T KNOW 8	
1020	CHECK 430 FOR LAST BIRTH:<br>ANY CODE '21-36' CIRCLED ☐ ↓	OTHER ☐ →	1024
1021	Between the time you went for delivery but before the baby was born, were you offered an HIV test?	YES . . . . 1<br>NO 2	
1022	I don't want to know the results, but were you tested for HIV at that time?	YES . . . . 1<br>NO 2	 2 → 1024
1023	I don't want to know the results, but did you get the results of the test?	YES . . . . 1<br>NO 2	1, 2 → 1025
1024	CHECK 1016:<br>YES ☐ ↓	NO OR NOT ASKED ☐ →	1027
1025	Have you been tested for HIV since that time you were tested during your pregnancy?	YES . . . . 1<br>NO 2	1 → 1028
1026	How many months ago was your most recent HIV test?	MONTHS AGO . . . . ☐☐<br><br>TWO OR MORE YEARS 95	→ 1033

NO.	QUESTIONS AND FILTERS	CODING CATEGORIES	SKIP
1027	I don't want to know the results, but have you ever been tested for HIV?	YES . . . 1<br>NO . . . 2	 → 1031
1028	How many months ago was your most recent HIV test?	MONTHS AGO . . . [ ][ ]<br>TWO OR MORE YEARS . . . 95	
1029	I don't want to know the results, but did you get the results of the test?	YES . . . 1<br>NO . . . 2	
1030	Where was the test done?<br><br>PROBE TO IDENTIFY THE TYPE OF SOURCE.<br><br>IF UNABLE TO DETERMINE IF PUBLIC OR PRIVATE SECTOR, RECORD 96 AND WRITE THE NAME OF THE PLACE.	**PUBLIC SECTOR**<br>GOVERNMENT HOSPITAL . . . 11<br>GOVERNMENT CLINIC/COMMUNITY HEALTH CENTRE . . . 12<br>MOBILE/TEMPORARY HCT SERVICES . . . 13<br>OTHER PUBLIC SECTOR<br>________ 16<br>(SPECIFY)<br><br>**PRIVATE MEDICAL SECTOR**<br>PRIVATE HOSPITAL/CLINIC/ PRIVATE DOCTOR . . . 21<br>NEW START TESTING SITE . . . 22<br>CHEMIST/PHARMACY . . . 23<br>OTHER PRIVATE MEDICAL SECTOR<br>________ 26<br>(SPECIFY)<br>**OTHER SOURCE**<br>HOME . . . 31<br>WORKPLACE . . . 32<br>CORRECTIONAL FACILITY . . . 33<br>OTHER ________ 96<br>(SPECIFY)	→ 1033
1031	Do you know of a place where people can go to get an HIV test?	YES . . . 1<br>NO . . . 2	 → 1033
1032	Where is that?<br><br>Any other place?<br><br>PROBE TO IDENTIFY THE TYPE OF SOURCE.<br><br>IF UNABLE TO DETERMINE IF PUBLIC OR PRIVATE SECTOR, RECORD 'X' AND WRITE THE NAME OF THE PLACE(S).	**PUBLIC SECTOR**<br>GOVERNMENT HOSPITAL . . . A<br>GOVERNMENT CLINIC/COMMUNITY HEALTH CENTRE . . . B<br>MOBILE/TEMPORARY HCT SERVICES . . . C<br>OTHER PUBLIC SECTOR<br>________ D<br>(SPECIFY)<br><br>**PRIVATE MEDICAL SECTOR**<br>PRIVATE HOSPITAL/CLINIC/ PRIVATE DOCTOR . . . E<br>NEW START TESTING SITE . . . F<br>CHEMIST/PHARMACY . . . G<br>OTHER PRIVATE MEDICAL SECTOR<br>________ H<br>(SPECIFY)<br><br>OTHER ________ X<br>(SPECIFY)	

NO.	QUESTIONS AND FILTERS	CODING CATEGORIES	SKIP
1033	Have you heard of test kits people can use to test themselves for HIV?	YES . . . . . 1<br>NO 2	 2 → 1042
1034	Have you ever tested yourself for HIV using a self-test kit?	YES . . . . . 1<br>NO 2	
1042	CHECK 1001:<br>HEARD ABOUT HIV OR AIDS ☐ ↓ a) Apart from HIV, have you heard about other infections that can be transmitted through sexual contact?<br>NOT HEARD ABOUT HIV OR AIDS ☐ ↓ b) Have you heard about infections that can be transmitted through sexual contact?	YES . . . . . 1<br>NO 2	
1043	CHECK 713:<br>HAS HAD SEXUAL INTERCOURSE ☐ ↓<br>NEVER HAD SEXUAL INTERCOURSE ☐ → 1101		NEVER HAD → 1101
1044	CHECK 1042: HEARD ABOUT OTHER SEXUALLY TRANSMITTED INFECTIONS?<br>YES ☐ ↓<br>NO ☐ → 1046		NO → 1046
1045	Now I would like to ask you some questions about your health in the last 12 months. During the last 12 months, have you had a disease which you got through sexual contact?	YES . . . . . 1<br>NO . . . . . 2<br>DON'T KNOW 8	
1046	Sometimes women experience a bad-smelling abnormal genital discharge. During the last 12 months, have you had a bad-smelling abnormal genital discharge?	YES . . . . . 1<br>NO . . . . . 2<br>DON'T KNOW 8	
1047	Sometimes women have a genital sore or ulcer. During the last 12 months, have you had a genital sore or ulcer?	YES . . . . . 1<br>NO . . . . . 2<br>DON'T KNOW 8	
1048	CHECK 1045, 1046, AND 1047:<br>HAS HAD AN INFECTION (ANY 'YES') ☐ ↓<br>HAS NOT HAD AN INFECTION OR DOES NOT KNOW ☐ → 1053		HAS NOT HAD → 1053
1049	The last time you had (PROBLEM FROM 1045/1046/1047), did you seek any kind of advice or treatment?	YES . . . . . 1<br>NO 2	 2 → 1053

NO.	QUESTIONS AND FILTERS	CODING CATEGORIES	SKIP
1050	Where did you go?<br><br>Any other place?<br><br>PROBE TO IDENTIFY THE TYPE OF SOURCE.<br><br>IF UNABLE TO DETERMINE IF PUBLIC OR PRIVATE SECTOR, RECORD 'X' AND WRITE THE NAME OF THE PLACE(S).	**PUBLIC SECTOR**<br>GOVERNMENT HOSPITAL . . . . A<br>GOVERNMENT CLINIC/COMMUNITY HEALTH CENTRE . . . . B<br>MOBILE/TEMPORARY HCT SERVICES . . . . . C<br>OTHER PUBLIC SECTOR<br>_____________ D<br>(SPECIFY)<br><br>**PRIVATE MEDICAL SECTOR**<br>PRIVATE HOSPITAL/CLINIC/ PRIVATE DOCTOR . . . . E<br>NEW START TESTING SITE . . . . F<br>CHEMIST/PHARMACY . . . . G<br><br>OTHER PRIVATE MEDICAL SECTOR<br>_____________ H<br>(SPECIFY)<br>**OTHER SOURCE**<br>SHOP . . . . I<br>TRADITIONAL HERBALIST . . . . J<br>TRADITIONAL HEALER . . . . K<br><br>OTHER _____________ X<br>(SPECIFY)	
1053	CHECK 701, 701A AND 701B:<br>CURRENTLY MARRIED/ LIVING WITH A MAN OR HAS REGULAR PARTNER/BOYFRIEND ☐ ↓	NOT IN UNION OR NO REGULAR PARTNER/BOYFRIEND OR NOT IN UNION/PARTNERED WITH A MAN ☐	→ 1101
1054	Can you say no to your (husband/partner) if you do not want to have sexual intercourse?	YES . . . . 1<br>NO . . . . 2<br>DEPENDS/NOT SURE 8	
1055	Could you ask your (husband/partner) to use a condom if you wanted him to?	YES . . . . 1<br>NO . . . . 2<br>DEPENDS/NOT SURE 8	

NO.		CODING CATEGORIES	SKIP
1101	Now I would like to ask you some questions about your brothers and sisters, that is, all of the children born to your biological mother, including those who are living with you, those living elsewhere and those who have died. How many children did your mother give birth to, including you?	NUMBER OF BIRTHS TO BIOLOGICAL MOTHER	
1102	CHECK 1101:<br>TWO OR MORE BIRTHS ☐ ↓ ONLY ONE BIRTH (RESPONDENT ONLY) ☐		→ 1201
1103	How many births did your mother have before you were born?	NUMBER OF PRECEDING BIRTHS	

1104	What was the name given to your oldest (next oldest) brother or sister?	(1)<br>________	(2)<br>________	(3)<br>________	(4)<br>________	(5)<br>________	(6)<br>________
1105	Is (NAME) male or female?	MALE 1<br>FEMALE 2	MALE 1<br>FEMALE 2	MALE 1<br>FEMALE 2	MALE 1<br>FEMALE 2	MALE 1<br>FEMALE 2	MALE 1<br>FEMALE 2
1106	Is (NAME) still alive?	YES . . . 1<br>NO . . . 2<br>GO TO 1108<br>DK . . . 8<br>GO TO (2)	YES . . . 1<br>NO . . . 2<br>GO TO 1108<br>DK . . . 8<br>GO TO (3)	YES . . . 1<br>NO . . . 2<br>GO TO 1108<br>DK . . . 8<br>GO TO (4)	YES . . . 1<br>NO . . . 2<br>GO TO 1108<br>DK . . . 8<br>GO TO (5)	YES . . . 1<br>NO . . . 2<br>GO TO 1108<br>DK . . . 8<br>GO TO (6)	YES . . . 1<br>NO . . . 2<br>GO TO 1108<br>DK . . . 8<br>GO TO (7)
1107	How old is (NAME)?	GO TO (2)	GO TO (3)	GO TO (4)	GO TO (5)	GO TO (6)	GO TO (7)
1108	How many years ago did (NAME) die?						
1109	How old was (NAME) when he/she died?<br><br>IF DON'T KNOW, PROBE TO GET AN ESTIMATE.	IF MALE OR DIED BEFORE 12 YEARS OF AGE<br>GO TO 1114	IF MALE OR DIED BEFORE 12 YEARS OF AGE<br>GO TO 1114	IF MALE OR DIED BEFORE 12 YEARS OF AGE<br>GO TO 1114	IF MALE OR DIED BEFORE 12 YEARS OF AGE<br>GO TO 1114	IF MALE OR DIED BEFORE 12 YEARS OF AGE<br>GO TO 1114	IF MALE OR DIED BEFORE 12 YEARS OF AGE<br>GO TO 1114
1110	Was (NAME) pregnant when she died?	YES . . . 1<br>GO TO 1113<br>NO . . . 2	YES . . . 1<br>GO TO 1113<br>NO . . . 2	YES . . . 1<br>GO TO 1113<br>NO . . . 2	YES . . . 1<br>GO TO 1113<br>NO . . . 2	YES . . . 1<br>GO TO 1113<br>NO . . . 2	YES . . . 1<br>GO TO 1113<br>NO . . . 2
1111	Did (NAME) die during childbirth?	YES . . . 1<br>GO TO 1113<br>NO . . . 2	YES . . . 1<br>GO TO 1113<br>NO . . . 2	YES . . . 1<br>GO TO 1113<br>NO . . . 2	YES . . . 1<br>GO TO 1113<br>NO . . . 2	YES . . . 1<br>GO TO 1113<br>NO . . . 2	YES . . . 1<br>GO TO 1113<br>NO . . . 2
1112	Did (NAME) die within two months after the end of a pregnancy or childbirth?	YES . . . 1<br>NO . . . 2	YES . . . 1<br>NO . . . 2	YES . . . 1<br>NO . . . 2	YES . . . 1<br>NO . . . 2	YES . . . 1<br>NO . . . 2	YES . . . 1<br>NO . . . 2
1113	How many live born children did (NAME) give birth to during her lifetime?						
1114	Was (NAME)'s death due to an accident or violence?	YES . . . 1<br>NO . . . 2	YES . . . 1<br>NO . . . 2	YES . . . 1<br>NO . . . 2	YES . . . 1<br>NO . . . 2	YES . . . 1<br>NO . . . 2	YES . . . 1<br>NO . . . 2

IF NO MORE BROTHERS OR SISTERS, GO TO NEXT SECTION.

SECTION 12. TOBACCO AND ALCOHOL

NO.	QUESTIONS AND FILTERS	CODING CATEGORIES	SKIP
1201	CHECK COVER SHEET: IS HOUSEHOLD SELECTED FOR MALE SURVEY AND BIOMARKERS OR IS RESPONDENT AGE 50 OR OLDER AND SELECTED FOR HOUSEHOLD RELATIONS MODULE?<br>YES ☐	NO ☐	→ 1501
1202	Would you say your health is poor, average, good, or excellent?	POOR . . . 1<br>AVERAGE . . . 2<br>GOOD . . . 3<br>EXCELLENT . . . 4	
1203	Do you personally think you are underweight, normal weight, overweight, or obese?	UNDERWEIGHT . . . 1<br>NORMAL WEIGHT . . . 2<br>OVERWEIGHT . . . 3<br>OBESE . . . 4<br>DON'T KNOW . . . 8	
1204	Do you currently smoke tobacco every day, some days, or not at all?	EVERY DAY . . . 1<br>SOME DAYS . . . 2<br>NOT AT ALL . . . 3	1 → 1207<br><br>3 → 1206
1205	In the past, have you smoked tobacco every day?	YES . . . 1<br>NO . . . 2	1, 2 → 1208
1206	In the past, have you ever smoked tobacco every day, some days, or not at all?	EVERY DAY . . . 1<br>SOME DAYS . . . 2<br>NOT AT ALL . . . 3	1, 2, 3 → 1209
1207	On average, how many of the following products do you currently smoke each day? Also, let me know if you use the product, but not every day.<br><br>IF RESPONDENT REPORTS USING THE PRODUCT BUT NOT EVERY DAY, RECORD '888'. IF THE PRODUCT IS NOT USED AT ALL, RECORD '000'.	NUMBER DAILY	
	a) Manufactured cigarettes?	a) MANUFACT. CIGARETTES ☐☐☐	→ 1209
	b) Hand-rolled cigarettes?	b) HAND-ROLLED CIGARETTES ☐☐☐	
	c) Pipes full of tobacco?	c) PIPES FULL OF TOBACCO ☐☐☐	
	d) Cigars or cigarillos?	d) CIGARS OR CIGARILLOS ☐☐☐	
	e) Number of hookah, hubbly-bubbly or water pipe sessions?	e) WATER PIPE SESSIONS ☐☐☐	
	f) Any others?<br>__________ (SPECIFY)	f) OTHERS . . . ☐☐☐	

NO.	QUESTIONS AND FILTERS	CODING CATEGORIES	SKIP
1208	On average, how many of the following products do you currently smoke each week? Also, let me know if you use the product, but not every week.<br><br>IF RESPONDENT REPORTS USING THE PRODUCT BUT NOT EVERY WEEK, RECORD '888'. IF THE PRODUCT IS NOT USED AT ALL, RECORD '000'.<br><br>a) Manufactured cigarettes?<br>b) Hand-rolled cigarettes?<br>c) Pipes full of tobacco?<br>d) Cigars or cigarillos?<br>e) Number of hookah, hubbly-bubbly or water pipe sessions?<br>f) Any others?<br>__________ (SPECIFY)	NUMBER WEEKLY<br><br>a) MANUFACT. CIGARETTES [ ][ ][ ]<br>b) HAND-ROLLED CIGARETTES [ ][ ][ ]<br>c) PIPES FULL OF TOBACCO [ ][ ][ ]<br>d) CIGARS OR CIGARILLOS [ ][ ][ ]<br>e) WATER PIPE SESSIONS [ ][ ][ ]<br>f) OTHERS . . . . [ ][ ][ ]	
1209	Do you currently use snuff, chewing tobacco or other smokeless tobacco products every day, some days, or not at all?	EVERY DAY . . . . 1<br>SOME DAYS . . . . 2<br>NOT AT ALL 3	1 → 1211<br>2 → 1212
1210	In the past, have you used snuff, chewing tobacco or other smokeless tobacco products every day, some days, or not at all?	EVERY DAY . . . . 1<br>SOME DAYS . . . . 2<br>NOT AT ALL 3	1, 2, 3 → 1213
1211	On average, how many times a day do you use the following products? Also, let me know if you use the product, but not every day.<br><br>IF RESPONDENT REPORTS USING THE PRODUCT BUT NOT EVERY DAY, RECORD '888'. IF THE PRODUCT IS NOT USED AT ALL, RECORD '000'.<br><br>a) Snuff, by mouth?<br>b) Snuff, by nose?<br>c) Chewing tobacco?<br>d) Any others?<br>__________ (SPECIFY)	TIMES DAILY<br><br>a) SNUFF, BY MOUTH . . . . . [ ][ ][ ]<br>b) SNUFF, BY NOSE . . . . . [ ][ ][ ]<br>c) CHEWING TOBACCO . . . . . [ ][ ][ ]<br>d) ANY OTHERS . . . . [ ][ ][ ]	a–d → 1213
1212	On average, how many times a week do you use the following products? Also, let me know if you use the product, but not every week.<br><br>IF RESPONDENT REPORTS USING THE PRODUCT BUT NOT EVERY WEEK, RECORD '888'. IF THE PRODUCT IS NOT USED AT ALL, RECORD '000'.<br><br>a) Snuff, by mouth?<br>b) Snuff, by nose?<br>c) Chewing tobacco?<br>d) Any others?<br>__________ (SPECIFY)	TIMES WEEKLY<br><br>a) SNUFF, BY MOUTH . . . . . [ ][ ][ ]<br>b) SNUFF, BY NOSE . . . . . [ ][ ][ ]<br>c) CHEWING TOBACCO . . . . . [ ][ ][ ]<br>d) ANY OTHERS . . . . [ ][ ][ ]	

NO.	QUESTIONS AND FILTERS	CODING CATEGORIES	SKIP
1213	CHECK 106: AGE OF RESPONDENT<br>AGE 15-49 ☐ ↓	AGE 50 AND ABOVE ☐	→ 1220
1214	CHECK 224:<br>LIVE BIRTH SINCE JANUARY 2011? YES ☐ ↓	NO ☐	→ 1220
1215	CHECK 212 AND 215:<br>____________________<br>(NAME OF YOUNGEST CHILD) ↓		
1216	CHECK 1204 AND 1206:<br>CURRENTLY SMOKES TOBACCO OR SMOKED IN THE PAST? YES ☐ ↓	NO ☐	→ 1218
1217	During your pregnancy with (NAME) how often did you smoke tobacco: every day, some days, or not at all?	EVERY DAY . . . 1<br>SOME DAYS . . . 2<br>NOT AT ALL . . . 3	
1218	CHECK 1209 AND 1210:<br>CURRENTLY USES SMOKELESS TOBACCO OR USED IN THE PAST? YES ☐ ↓	NO ☐	→ 1220
1219	During your pregnancy with (NAME) how often did you use smokeless tobacco: every day, some days, or not at all?	EVERY DAY . . . 1<br>SOME DAYS . . . 2<br>NOT AT ALL . . . 3	
1220	Do you currently work in a job where other people smoke tobacco around you?	YES . . . 1<br>NO . . . 2<br>NOT CURRENTLY WORKING . . . 3	
1221	Have you ever worked in a job where you were regularly exposed to smoke, dust, fumes or strong smells?	YES . . . 1<br>NO . . . 2	 2 → 1223
1222	How many years did you work at a job where you were regularly exposed to smoke, dust, fumes or strong smells?<br>IF LESS THAN 1 YEAR, RECORD '00'.	YEARS . . . ☐☐	
1223	Do you currently use e-cigarettes every day, some days, or not at all?	EVERY DAY . . . 1<br>SOME DAYS . . . 2<br>NOT AT ALL . . . 3	
1224	Have you ever consumed a drink that contains alcohol such as beer, wine, ciders, spirits, or sorghum beer?<br>PROBE: Even one drink?	YES . . . 1<br>NO . . . 2	 2 → 1301
1225	Was this within the last 12 months?	YES . . . 1<br>NO . . . 2	 2 → 1233
1226	In the last 12 months, how frequently have you had at least one drink?<br>PROBE: Five or more days a week, 1-4 days a week, 1-3 days a month, or less often than once a month?	5 OR MORE DAYS A WEEK . . . 1<br>1-4 DAYS PER WEEK . . . 2<br>1-3 DAYS A MONTH . . . 3<br>LESS OFTEN THAN ONCE A MONTH . . . 4	

NO.	QUESTIONS AND FILTERS	CODING CATEGORIES	SKIP
1227	During each of the last 7 days, how many standard drinks did you have?<br><br>USE SHOWCARD. RECORD TOTAL NUMBER OF DRINKS CONSUMED EACH DAY STARTING WITH THE DAY BEFORE THE DAY OF THE INTERVIEW AND PROCEEDING BACKWARDS.<br><br>IF NONE, RECORD '00'.	MONDAY . . . . [ ][ ]<br>TUESDAY . . . . [ ][ ]<br>WEDNESDAY . . . . [ ][ ]<br>THURSDAY . . . . [ ][ ]<br>FRIDAY . . . . [ ][ ]<br>SATURDAY . . . . [ ][ ]<br>SUNDAY . . . . [ ][ ]	
1227H	During the last 7 days, how many standard home-made beers or other homemade alcohol did you have?<br><br>USE SHOWCARD.	NUMBER OF HOME-MADE BEERS . . [ ][ ]	
1227I	CHECK 1226 AND 1227: CODE 3 OR 4 RECORDED IN 1226 AND CONSUMED 0-1 DRINKS IN THE LAST 7 DAYS IN 1227?<br>NO [ ] ↓	YES [ ]	→ 1233
1228	Have you ever felt that you should cut down on your drinking?	YES . . . . 1<br>NO 2	
1229	Have people annoyed you by criticizing your drinking?	YES . . . . 1<br>NO 2	
1230	Have you ever felt bad or guilty about your drinking?	YES . . . . 1<br>NO 2	
1231	Have you ever had a drink first thing in the morning to steady your nerves or get rid of a hangover?	YES . . . . 1<br>NO 2	
1231A	CHECK 1227: FIVE OR MORE DRINKS IN ONE DAY DURING LAST 7 DAYS?<br>NO [ ] ↓	YES [ ]	→ 1233
1232	In the past 30 days, have you consumed five or more standard drinks on at least one occasion?	YES . . . . 1<br>NO 2	
1233	CHECK 106: AGE OF RESPONDENT<br>AGE 15-49 [ ] ↓	AGE 50 AND ABOVE [ ]	→ 1301
1234	CHECK 224:<br>LIVE BIRTH SINCE JANUARY 2011? YES [ ] ↓	NO [ ]	→ 1301
1235	CHECK 212 AND 215:<br>______________________<br>(NAME OF YOUNGEST CHILD) ↓		
1236	During your pregnancy with (NAME) how often did you drink alcohol: every day, some days, or not at all?	EVERY DAY . . . . 1<br>SOME DAYS . . . . 2<br>NOT AT ALL 3	

SECTION 13. FAT, SALT, SUGAR, FRUIT AND VEGETABLE CONSUMPTION

NO.	QUESTIONS AND FILTERS	CODING CATEGORIES	SKIP
1301	Now I would like to ask you some questions about the foods that you eat. There are no right or wrong answers.<br><br>USE SHOWCARD.		
1304	How often do you usually eat fried foods such as hot chips, fried fish, fried chicken, fried meat, vetkoek or doughnuts?	EVERY DAY . . . . . 1<br>AT LEAST ONCE A WEEK . . . . . 2<br>OCCASIONALLY . . . . . 3<br>NEVER 4	
1305	How often do you eat fast-foods or take-away foods from places like Chicken Licken, KFC, Captain DoRego's, Steers, Nando's, McDonalds, pizza delivery, etc?	EVERY DAY . . . . . 1<br>AT LEAST ONCE A WEEK . . . . . 2<br>OCCASIONALLY . . . . . 3<br>NEVER 4	
1306	How often do you eat chips such as a packet of crispy chips or similar salty snacks such as Doritos, cheese curls, salted nuts, salty biscuits, etc?	EVERY DAY . . . . . 1<br>AT LEAST ONCE A WEEK . . . . . 2<br>OCCASIONALLY . . . . . 3<br>NEVER 4	
1307	How often do you eat processed meat such as polony, viennas, meat pies, or sausage rolls?	EVERY DAY . . . . . 1<br>AT LEAST ONCE A WEEK . . . . . 2<br>OCCASIONALLY . . . . . 3<br>NEVER 4	
1308	Which of the following statements best describes your approach towards salt consumption:<br><br>1) I am not interested in lowering salt in my food.<br><br>2) I am interested in lowering salt in my food within the next six months.<br><br>3) I am interested in lowering salt in my food within the next month.<br><br>4) I have started lowering salt within the last six months.<br><br>5) I have already lowered my salt intake for longer than six months.	NO INTENTION TO LOWER SALT . . . . . 1<br>INTERESTED WITHIN NEXT SIX MONTHS . . . . . 2<br>INTERESTED WITHIN NEXT MONTH . . . . . 3<br>STARTED IN LAST SIX MONTHS . . . . . 4<br>ALREADY LOWERED LONGER THAN SIX MONTHS 5<br><br>DON'T KNOW 8	
1309	Yesterday, how many types of fruit did you eat?<br><br>USE SHOWCARD. IF NONE, RECORD '00'.	TYPES OF FRUIT . . . . . [ ][ ]	
1310	Yesterday, how many types of vegetables, excluding potatoes, did you eat?<br>USE SHOWCARD. IF NONE, RECORD '00'.	TYPES OF VEGETABLES . . . . . [ ][ ]	
1311	Yesterday, did you drink any sugar-sweetened drinks? Sugar-sweetened drinks include fizzy drinks like Coke or drinks like Squash where water is added, but not diet or unsweetened cold drinks.	YES . . . . . 1<br>NO 2	 → 1312
1311A	How many and what size sugar-sweetened drinks did you drink?<br><br>PROBE FOR BEVERAGE NUMBER AND SIZE.	200 ML GLASS . . . . . A [ ][ ]<br>330 ML CAN OR BOTTLE . . . . . B [ ][ ]<br>500 ML BOTTLE . . . . . C [ ][ ]<br>1 L BOTTLE . . . . . D [ ][ ]<br>2 L BOTTLE . . . . . E [ ][ ]	
1312	Yesterday, did you drink any fruit juice?	YES . . . . . 1<br>NO 2	 → 1401
1312A	How many and what size fruit juices did you drink?<br><br>PROBE FOR BEVERAGE NUMBER AND SIZE.	200 ML JUICE CARTON . . . . . A [ ][ ]<br>200 ML GLASS . . . . . B [ ][ ]	

SECTION14. HEALTH CARE

NO.	QUESTIONS AND FILTERS	CODING CATEGORIES	SKIP
1401	Many different factors can prevent women from getting medical advice or treatment for themselves. When you are sick and want to get medical advice or treatment, is each of the following a big problem or not a big problem:<br><br>a) Getting permission to go to the doctor?<br>b) Getting money needed for advice or treatment?<br>c) The distance to the health facility?<br>d) Not wanting to go alone?	BIG PROBLEM / NOT A BIG PROBLEM<br>a) PERMISSION TO GO ..... 1 2<br>b) GETTING MONEY .... 1 2<br>c) DISTANCE .... 1 2<br>d) GO ALONE 1 2	
1402	Are you covered by Medical Aid, Medical Benefit Scheme, Provident Scheme, or Hospital Plan that helps you pay for health care or drug services?	YES ..... 1<br>NO 2	
1404	During the last month, have you received health, medical, or dental care without staying overnight?	YES ..... 1<br>NO 2	NO → 1406
1405	Where have you received health, medical, or dental care?<br><br>PROBE: Anywhere else?<br><br>PROBE TO IDENTIFY THE TYPE OF SOURCE.<br><br>IF UNABLE TO DETERMINE IF PUBLIC OR PRIVATE SECTOR, RECORD 'X' AND WRITE THE NAME OF THE PLACE(S).	**PUBLIC SECTOR**<br>GOVERNMENT HOSPITAL ..... A<br>GOVERNMENT CLINIC/COMMUNITY HEALTH CENTRE ..... B<br>OTHER PUBLIC SECTOR<br>______ C<br>(SPECIFY)<br><br>**PRIVATE MEDICAL SECTOR**<br>PRIVATE HOSPITAL/CLINIC/ PRIVATE DOCTOR ..... D<br>CHEMIST/PHARMACY ..... E<br>DENTIST/ORAL HYGIENIST/ DENTAL THERAPIST ..... F<br>OTHER PRIVATE MEDICAL SECTOR<br>______ G<br>(SPECIFY)<br>**OTHER SOURCE**<br>WORKPLACE HEALTH SERVICE ..... H<br>TRADITIONAL HEALER ..... I<br>TRADITIONAL HERBALIST ..... J<br>FAITH HEALER ..... K<br><br>OTHER ______ X<br>(SPECIFY)	
1406	During the last month, have you had any visits by a home-based care giver or a community-based care giver?	YES ..... 1<br>NO ..... 2<br>DON'T KNOW 8	
1407	Have you ever had a Pap smear?<br>PROBE: When visiting a doctor or nurse, have you ever been asked to lie on your back with your legs apart so they could use a stick to take a sample from your vagina? The sample would have been sent to a laboratory for testing.	YES ..... 1<br>NO ..... 2<br>DON'T KNOW 8	NO, DON'T KNOW → 1410

NO.	QUESTIONS AND FILTERS	CODING CATEGORIES	SKIP
1408	How many years ago was your last Pap smear?	WITHIN THE LAST 3 YEARS . . . 1<br>4-5 YEARS AGO . . . 2<br>6-10 YEARS AGO . . . 3<br>MORE THAN 10 YEARS AGO . . . 4<br>DON'T KNOW/DON'T REMEMBER . . . 8	
1409	The last time you had a Pap smear, did you get the result of the test?	YES . . . 1<br>NO . . . 2<br>DON'T KNOW/DON'T REMEMBER . . . 8	
1410	Has a doctor, nurse or health worker ever told you that you have TB?	YES . . . 1<br>NO . . . 2<br>DON'T KNOW . . . 8	(NO, DON'T KNOW) → 1413
1411	When was the last time you were told you had TB?	IN THE LAST 12 MONTHS . . . 1<br>MORE THAN 12 MONTHS AGO . . . 2	
1412	Did you get medical treatment the last time you had TB?	YES . . . 1<br>NO . . . 2<br>DON'T KNOW/DON'T REMEMBER . . . 8	
1413	Has a doctor, nurse or health worker told you that you have or have had any of the following conditions:<br><br>a) High blood pressure?<br>b) Heart attack or angina/chest pains?<br>c) Cancer?<br>d) Stroke?<br>e) High blood cholesterol or fats in the blood?<br>f) Diabetes or blood sugar?<br>g) Chronic bronchitis, emphysema, or COPD?<br>h) Asthma?	YES NO DK<br><br>a) HIGH BLOOD PRESS. 1 2 8<br>b) HEART ATTACK 1 2 8<br>c) CANCER 1 2 8<br>d) STROKE 1 2 8<br>e) HIGH BLOOD CHOLEST 1 2 8<br>f) DIABETES 1 2 8<br>g) CHRONIC BRONCHITIS 1 2 8<br>h) ASTHMA 1 2 8	
1414	CHECK 1413:<br>ANY QUESTION a-h = YES?	YES ☐ ↓ NO ☐ →	1432
1415	CHECK 1413a:<br>RESPONDENT HAS HAD HIGH BLOOD PRESSURE.	1413a = YES ☐ ↓ 1413a = NO OR DK ☐ →	1417
1416	Did you receive medical treatment for high blood pressure at the time of the diagnosis?	YES . . . 1<br>NO . . . 2<br>DON'T KNOW/DON'T REMEMBER . . . 8	
1417	CHECK 1413b:<br>RESPONDENT HAS HAD HEART ATTACK OR ANGINA.	1413b = YES ☐ ↓ 1413b = NO OR DK ☐ →	1419
1418	Did you receive medical treatment for the heart attack, angina/chest pains at the time of the diagnosis?	YES . . . 1<br>NO . . . 2<br>DON'T KNOW/DON'T REMEMBER . . . 8	
1419	CHECK 1413c:<br>RESPONDENT HAS HAD CANCER.	1413c = YES ☐ ↓ 1413c = NO OR DK ☐ →	1421
1420	Did you receive medical treatment for the cancer at the time of the diagnosis?	YES . . . 1<br>NO . . . 2<br>DON'T KNOW/DON'T REMEMBER . . . 8	
1421	CHECK 1413d:<br>RESPONDENT HAS HAD STROKE.	1413d = YES ☐ ↓ 1413d = NO OR DK ☐ →	1423
1422	Did you receive medical treatment for the stroke at the time of the diagnosis?	YES . . . 1<br>NO . . . 2<br>DON'T KNOW/DON'T REMEMBER . . . 8	

NO.	QUESTIONS AND FILTERS	CODING CATEGORIES	SKIP
1423	CHECK 1413e:<br>RESPONDENT HAS HAD HIGH BLOOD CHOLESTEROL.	1413e = YES ☐ ↓ 1413e = NO OR DK ☐	→ 1425
1424	Did you receive medical treatment for high blood cholesterol or fats in the blood at the time of the diagnosis?	YES . . . 1<br>NO . . . 2<br>DON'T KNOW/DON'T REMEMBER . . . 8	
1425	CHECK 1413f:<br>RESPONDENT HAS HAD DIABETES.	1413f = YES ☐ ↓ 1413f = NO OR DK ☐	→ 1427
1426	Did you receive medical treatment for the diabetes or blood sugar at the time of the diagnosis?	YES . . . 1<br>NO . . . 2<br>DON'T KNOW/DON'T REMEMBER . . . 8	
1427	CHECK 1413g:<br>RESPONDENT HAS HAD CHRONIC BRONCHITIS.	1413g = YES ☐ ↓ 1413g = NO OR DK ☐	→ 1429
1428	Did you receive medical treatment for chronic bronchitis, emphysema, or COPD at the time of the diagnosis?	YES . . . 1<br>NO . . . 2<br>DON'T KNOW/DON'T REMEMBER . . . 8	
1429	CHECK 1413h:<br>RESPONDENT HAS HAD ASTHMA.	1413h = YES ☐ ↓ 1413h = NO OR DK ☐	→ 1432
1430	Did you receive medical treatment for asthma at the time of the diagnosis?	YES . . . 1<br>NO . . . 2<br>DON'T KNOW/DON'T REMEMBER . . . 8	
1432	Compared with other people your age, do you feel you have less breath when exerting yourself?<br><br>PROBE: By exercising or moving a lot?	YES . . . 1<br>NO . . . 2<br>DON'T KNOW . . . 8	
1433	During the last 12 months, have you had wheezing when you breathe?	YES . . . 1<br>NO . . . 2<br>DON'T KNOW . . . 8	NO, DON'T KNOW → 1436
1434	Were you also short of breath when the wheezing noise was present?	YES . . . 1<br>NO . . . 2<br>DON'T KNOW . . . 8	
1435	Have you had the wheezing when you did not have a cold?	YES . . . 1<br>NO . . . 2<br>DON'T KNOW . . . 8	
1436	Have you woken up with a feeling of tightness in your chest at any time in the last 12 months?	YES . . . 1<br>NO . . . 2<br>DON'T KNOW . . . 8	
1437	Have you been woken by an attack of shortness of breath at any time in the last 12 months?	YES . . . 1<br>NO . . . 2<br>DON'T KNOW . . . 8	
1438	Have you been woken by an attack of coughing at any time in the last 12 months?	YES . . . 1<br>NO . . . 2<br>DON'T KNOW . . . 8	
1439	Do you usually cough on most days?	YES . . . 1<br>NO . . . 2<br>DON'T KNOW . . . 8	NO, DON'T KNOW → 1443

SECTION14. HEALTH CARE

NO.	QUESTIONS AND FILTERS	CODING CATEGORIES	SKIP
1440	When you cough, do you usually bring up phlegm from your chest?	YES . . . . 1<br>NO . . . . 2<br>DON'T KNOW 8	 NO, DON'T KNOW → 1443
1441	Have you brought up phlegm every day for at least three months during the last year?	YES . . . . 1<br>NO . . . . 2<br>DON'T KNOW 8	 NO, DON'T KNOW → 1443
1442	For how many years have you brought up phlegm in this way?<br>IF LESS THAN 1 YEAR, RECORD '00'.	YEARS . . . . [ ][ ]	
1443	Are you currently troubled by pain or discomfort, either all the time or on and off?	YES . . . . 1<br>NO 2	 → 1446
1444	Have you had this pain or discomfort for more than 3 months?	YES . . . . 1<br>NO 2	 → 1446
1445	Where do you feel this pain or discomfort?<br><br>RECORD ALL MENTIONED.	BACK PAIN . . . . A<br>NECK OR SHOULDER PAIN . . . . B<br>HEADACHE, FACIAL OR DENTAL PAIN . . . . C<br>STOMACH ACHE OR ABDOMINAL PAIN . . . . D<br>PAIN IN ARMS, HANDS, HIPS, LEGS OR FEET E<br>CHEST PAIN . . . . F<br><br>OTHER ______ X<br>(SPECIFY)	
1446	In the last 12 months, did your teeth or your mouth cause you any pain or discomfort?	YES . . . . 1<br>NO 2	 → 1450
1447	Did you get treatment the last time that you had the problem?	YES . . . . 1<br>NO 2	 → 1449
1448	Who did you see for treatment?<br><br>RECORD ALL MENTIONED.	**PUBLIC SECTOR**<br>DENTIST/ORAL HYGIENIST/DENTAL THERAPIST . . . . A<br>MEDICAL DOCTOR/NURSE . . . . B<br><br>**PRIVATE MEDICAL SECTOR**<br>DENTIST/ORAL HYGIENIST/DENTAL THERAPIST . . . . C<br>MEDICAL DOCTOR/NURSE . . . . D<br><br>**OTHER SOURCE**<br>TRADITIONAL HEALER . . . . E<br><br>OTHER X	A–X → 1450
1449	What was the main reason that you did not get treatment?	NO ORAL HEALTH SERVICE AVAILABLE . . . . 1<br>ORAL HEALTH SERVICES TOO FAR . . . . 2<br>ORAL HEALTH SERVICES TOO EXPENSIVE/ COULD NOT AFFORD . . . . 3<br>PROBLEM WENT AWAY . . . . 4<br>OTHER 6	
1450	Now I would like to ask you about any medication you take. Do you use any medication daily or regularly that has been prescribed by a doctor or nurse?	YES . . . . 1<br>NO 2	 → 1455
1451	How many different prescribed medications do you use daily or regularly?	NUMBER OF MEDICINES . . . . [ ][ ]	

NO.	QUESTIONS AND FILTERS	CODING CATEGORIES	SKIP
1452	Who pays for most of these medications?	RESPONDENT . . . . . 1<br>FAMILY/FRIENDS . . . . . 2<br>MEDICAL AID . . . . . 3<br>EMPLOYER . . . . . 4<br>PROVIDED BY PUBLIC CLINIC OR HOSPITAL . . 5<br><br>OTHER 6	1–4 → 1455<br><br><br><br><br><br>6 → 1455
1453	In the last 12 months, have you ever been sent away from the clinic without a medication because they did not have stock?	YES . . . . . 1<br>NO 2	 2 → 1455
1454	How many times has this happened to you in the last 12 months?<br>PROBE FOR ESTIMATE OF NUMBER OF TIMES.	NUMBER OF TIMES . . . . . [ ][ ]	
1455	In the last 12 months, have you used any medications containing codeine to treat a medical condition?<br><br>USE THE SHOWCARD.	YES . . . . . 1<br>NO . . . . . 2<br>DON'T KNOW 8	 2, 8 → 1500
1457	In the last 12 months, have you used any of these medications for the experience or feeling it gave you rather than for their medicinal effect?	YES . . . . . 1<br>NO 2	 2 → 1500
1458	In the last 12 months, which codeine-containing medications have you used for the experience or feeling rather than for their medical effect?<br><br>RECORD ALL MENTIONED.	BRONCLEER/LENAZINE FORTE . . . . . A<br>ACTIFED DRY COUGH . . . . . B<br>BENYLIN SYRUP WITH CODEINE . . . . . C<br>LENADOL/ADCO-DOL PAIN TABLETS . . . . . D<br>NUROFEN PLUS . . . . . E<br>MYPRODOL . . . . . F<br>STILPANE . . . . . G<br>SYNDOL . . . . . H<br><br>OTHER ______________ X<br>(SPECIFY)	
1459	In the last 12 months, have you received treatment for your problems related to the use of codeine-containing medications for non-medical purposes?	YES . . . . . 1<br>NO 2	

SECTION 15: HOUSEHOLD RELATIONS

NO.	QUESTIONS AND FILTERS	CODING CATEGORIES	SKIP
1500	CHECK COVER PAGE AND 106:<br>WOMAN SELECTED FOR THIS SECTION AND AT LEAST 18 YEARS OLD ☐ ↓	WOMAN NOT SELECTED OR SELECTED BUT AGE 15-17 ☐	→ 1533
1501	CHECK FOR PRESENCE OF OTHERS:<br>DO NOT CONTINUE UNTIL PRIVACY IS ENSURED.<br>PRIVACY OBTAINED . . . . 1 ↓	PRIVACY NOT POSSIBLE 2	→ 1532
1501A	READ TO THE RESPONDENT:<br>Now I would like to ask you questions about some other important aspects of a woman's life. You may find some of these questions very personal. However, your answers are crucial for helping to understand the condition of women in South Africa. Let me assure you that your answers are completely confidential and will not be told to anyone and no one else in your household will know that you were asked these questions. If I ask you any question you don't want to answer, just let me know and I will go on to the next question.		
1502	CHECK 701, 701A, 701B AND 702:<br>NEVER IN UNION WITH A MAN ☐ ↓	CURRENTLY MARRIED/LIVING WITH A MAN OR HAS REGULAR MALE PARTNER/BOYFRIEND ☐<br>FORMERLY MARRIED/LIVED WITH A MAN (READ IN PAST TENSE AND USE 'LAST' WITH 'HUSBAND/PARTNER') ☐	→ 1503<br>→ 1503
1502A	Do you have a boyfriend or have you had one in the past?	YES, CURRENTLY HAS BOYFRIEND 1<br>YES, HAD BOYFRIEND IN PAST . . . 2<br>NO 3	 <br>→ 1516

1503	First, I am going to ask you about some situations which happen to some women. Please tell me if these apply to your relationship with your (last) (husband/partner/boyfriend)?		YES	NO	DK
	a) He (is/was) jealous or angry if you (talk/talked) to other men?	JEALOUS	1	2	8
	b) He frequently (accuses/accused) you of being unfaithful?	ACCUSES	1	2	8
	c) He (does/did) not permit you to meet your female friends?	NOT MEET FRIENDS	1	2	8
	d) He (tries/tried) to limit your contact with your family?	NO FAMILY	1	2	8
	e) He (insists/insisted) on knowing where you (are/were) at all times?	WHERE YOU ARE .	1	2	8

1504 Now I need to ask some more questions about your relationship with your most recent partner.

A Did your (last) partner ever:

B How often did this happen during the last 12 months: often, only sometimes, or not at all?

	EVER	OFTEN	SOME-TIMES	NOT IN LAST 12 MONTHS
a) say or do something to humiliate you in front of others?	YES 1 →<br>NO 2 ↓	1	2	3
b) threaten to hurt or harm you or someone you care about?	YES 1 →<br>NO 2 ↓	1	2	3
c) insult you or make you feel bad about yourself?	YES 1 →<br>NO 2 ↓	1	2	3
d) refuse to give you enough money for household expenses or contribute towards household expenses when he has the money to do so?	YES 1 →<br>NO OF 2<br>N/A ↓	1	2	3

NO.	QUESTIONS AND FILTERS	EVER	CODING CATEGORIES			SKIP
1505	A Did your (last) partner ever do any of the following things to you:		B How often did this happen during the last 12 months: often, only sometimes, or not at all?			
		EVER	OFTEN	SOME-TIMES	NOT IN LAST 12 MONTHS	
	a) slap you, push you, shake you, or throw something at you?	YES 1 → NO 2 ↓	1	2	3	
	e) kick you, drag you, or beat you up?	YES 1 → NO 2 ↓	1	2	3	
	f) try to choke you or burn you on purpose?	YES 1 → NO 2 ↓	1	2	3	
	g) threaten or attack you with a knife, gun, or other weapon?	YES 1 → NO 2 ↓	1	2	3	
	h) physically force you to have sexual intercourse with him when you did not want to?	YES 1 → NO 2 ↓	1	2	3	
	i) physically force you to perform any other sexual acts you did not want to?	YES 1 → NO 2 ↓	1	2	3	
	j) force you with threats or in any other way to perform sexual acts you did not want to?	YES 1 → NO 2 ↓	1	2	3	
1506	CHECK 1505A (a-j):<br>AT LEAST ONE 'YES' ☐ ↓<br>NOT A SINGLE 'YES' ☐					→ 1511
1508	Did the following ever happen as a result of what your (last) partner did to you:					
	a) You had cuts, bruises, or aches?		YES . . . 1<br>NO . . . 2			
	b) You had eye injuries, sprains, dislocations, or burns?		YES . . . 1<br>NO . . . 2			
	c) You had deep wounds, broken bones, broken teeth, or any other serious injury?		YES . . . 1<br>NO . . . 2			
1511	Does (did) your (last) partner drink alcohol?		YES . . . 1<br>NO . . . 2<br>DON'T KNOW . . . 8			NO, DON'T KNOW → 1512A
1512	How often does (did) he get drunk: often, only sometimes, or never?		OFTEN . . . 1<br>SOMETIMES . . . 2<br>NEVER . . . 3			
1512A	Does (did) your (last) partner take drugs?		YES . . . 1<br>NO . . . 2<br>DON'T KNOW . . . 8			NO, DON'T KNOW → 1512C
1512B	How often does (did) he take drugs: often, only sometimes, or never?		OFTEN . . . 1<br>SOMETIMES . . . 2<br>NEVER . . . 3			

NO.	QUESTIONS AND FILTERS	CODING CATEGORIES	SKIP
1512C	Have you ever hit, slapped, kicked, or done anything else to physically hurt your (last) partner at times when he was not already beating or physically hurting you?	YES . . . . 1<br>NO 2	 → 1513
1512D	In the last 12 months, how often have you done this to your (last) partner: often, only sometimes, or not at all?	OFTEN . . . . 1<br>SOMETIMES . . . . 2<br>NOT AT ALL 3	
1513	Are (Were) you afraid of your (last) partner: most of the time, sometimes, or never?	MOST OF THE TIME AFRAID . . . . . 1<br>SOMETIMES AFRAID . . . . 2<br>NEVER AFRAID 3	
1514	CHECK 709:<br>OTHER OR NOT ASKED ☐ ↓<br>MARRIED OR LIVED WITH A MAN MORE THAN ONCE ☐ →		→ 1515
1514A	So far we have been talking about the behaviour of your (current/last) partner. Now I want to ask you about the behaviour of any previous partner. Have you had a previous partner?	YES . . . . 1<br>NO 2	→ 1515Aa<br>→ 1516A

1515

A So far we have been talking about the behaviour of your (current/last) partner. Now I want to ask you about the behaviour of any previous partner.

B How long ago did this last happen?

	EVER	0 - 11 MONTHS AGO	12+ MONTHS AGO	DON'T REMEMBER
a) Did any previous partner ever hit, slap, kick, or do anything else to hurt you physically?	YES 1 →<br>NO 2 ↓	1	2	3
b) Did any previous partner physically force you to have sexual intercourse against your will?	YES 1 →<br>NO 2 ↓	1	2	3
c) Did any previous partner physically force you to perform any other sexual acts against your will?	YES 1 →<br>NO 2 ↓	1	2	3
d) Did any previous partner humiliate, threaten, belittle, insult or try to exert excessive control over you in any way?	YES 1 →<br>NO 2 ↓	1	2	3
e) Did any previous partner refuse to give you enough money for household expenses or contribute towards household expenses?	YES 1 →<br>NO OR N/A 2 ↓	1	2	3

NO.	QUESTIONS AND FILTERS	CODING CATEGORIES	SKIP
1516A	CHECK 1505A (h-j) and 1515A(b, c)<br>AT LEAST ONE 'YES' ☐ ↓<br>NOT A SINGLE 'YES' ☐ →		→ 1516
1516B	How old were you the first time you were forced to have sexual intercourse or perform any other sexual acts by (your/any) partner?	AGE IN COMPLETED YEARS ☐☐<br>DON'T KNOW 98	
1516	CHECK 701, 701A, 701B, 702 AND 1502A:<br>EVER IN UNION OR HAD A BOYFRIEND ☐ ↓<br>a) From the time you were 15 years old has anyone other than (your/any) partner hit you, slapped you, kicked you, or done anything else to hurt you physically?<br>NEVER IN UNION OR HAD A BOYFRIEND ☐ ↓<br>b) From the time you were 15 years old has anyone hit you, slapped you, kicked you, or done anything else to hurt you physically?	YES . . . . 1<br>NO . . . . 2<br>REFUSED TO ANSWER/ NO ANSWER 3	 → 1518A (NO, REFUSED/NO ANSWER)

NO.	QUESTIONS AND FILTERS	CODING CATEGORIES	SKIP
1517	Who has hurt you in this way?<br><br>Anyone else?<br><br>RECORD ALL MENTIONED.	MOTHER/STEP-MOTHER . . . . A<br>FATHER/STEP-FATHER . . . . B<br>SISTER/BROTHER . . . . C<br>DAUGHTER/SON . . . . D<br>OTHER RELATIVE . . . . E<br>MOTHER-IN-LAW . . . . F<br>FATHER-IN-LAW . . . . G<br>OTHER IN-LAW . . . . H<br>TEACHER . . . . I<br>EMPLOYER/SOMEONE AT WORK . J<br>POLICE/SOLDIER . . . . K<br>NEIGHBOUR . . . . L<br><br>OTHER ______________________ X<br>(SPECIFY)	
1518	In the last 12 months, how often has (this person/have these persons) physically hurt you: often, only sometimes, or not at all?	OFTEN . . . . 1<br>SOMETIMES . . . . 2<br>NOT AT ALL 3	
1518A	CHECK 106: AGE OF RESPONDENT<br><br>AGE 18-49 ☐ ↓ AGE 50 AND ABOVE ☐ →		1522
1519	CHECK 201, 226, AND 230:<br><br>EVER BEEN PREGNANT (YES ON 201 OR 226 OR 230) ☐ ↓ NEVER BEEN PREGNANT ☐ →		1522
1519A	CHECK 701, 701A, 701B, 702 AND 15O2A:<br><br>EVER IN UNION OR HAD A BOYFRIEND ☐ ↓ NEVER IN UNION OR HAD A BOYFRIEND ☐ →		1520A
1520	Has a partner ever hit, slapped, kicked, or done anything else to hurt you physically while you were pregnant?	YES . . . . 1<br>NO 2	
1520A	Has any one else ever hit, slapped, kicked, or done anything else to hurt you physically while you were pregnant?	YES . . . . 1<br>NO 2	
1521A	CHECK 1520 AND 1520A:<br><br>EITHER 1520 OR 1520A = YES ☐ ↓ NEITHER 1520 NOR 1520A = YES ☐ →		1522
1521	Who has done any of these things to physically hurt you while you were pregnant?<br><br>Anyone else?<br><br>RECORD ALL MENTIONED.	CURRENT HUSBAND/PARTNER . . . A<br>MOTHER/STEP-MOTHER . . . . B<br>FATHER/STEP-FATHER . . . . C<br>SISTER/BROTHER . . . . D<br>DAUGHTER/SON . . . . E<br>OTHER RELATIVE . . . . F<br>FORMER HUSBAND/PARTNER . . . G<br>CURRENT BOYFRIEND . . . . H<br>FORMER BOYFRIEND . . . . I<br>MOTHER-IN-LAW . . . . J<br>FATHER-IN-LAW . . . . K<br>OTHER IN-LAW . . . . L<br>TEACHER . . . . M<br>EMPLOYER/SOMEONE AT WORK . N<br>POLICE/SOLDIER . . . . O<br>NEIGHBOUR . . . . P<br><br>OTHER ______________________ X<br>(SPECIFY)	

NO.	QUESTIONS AND FILTERS	CODING CATEGORIES	SKIP
1522	CHECK 701, 701A, 701B, 702 AND 1502A:<br><br>EVER IN UNION OR HAD A BOYFRIEND ☐ ↓ | NEVER IN UNION OR HAD A BOYFRIEND ☐ ↓<br><br>a) Now I want to ask you about things that may have been done to you by someone other than (your/any) partner.<br><br>At any time in your life, as a child or as an adult, has anyone ever forced you in any way to have sexual intercourse when you did not want to?<br><br>b) At any time in your life, as a child or as an adult, has anyone ever forced you in any way to have sexual intercourse when you did not want to?	YES . . . . . 1<br>NO . . . . . 2<br>REFUSED TO ANSWER/<br>NO ANSWER 3	 2, 3 → 1522C
1522A	How old were you the first time this happened?	AGE IN COMPLETED YEARS [ ][ ]<br><br>DON'T KNOW 98	
1522B	Who was the person who was forcing you the very first time this happened?	FATHER/STEP-FATHER . . . . . 04<br>BROTHER/STEP-BROTHER . . . . . 05<br>OTHER RELATIVE . . . . . 06<br>IN-LAW . . . . . 07<br>OWN FRIEND/ACQUAINTANCE . . . 08<br>FAMILY FRIEND . . . . . 09<br>TEACHER . . . . . 10<br>EMPLOYER/SOMEONE AT WORK . I1<br>POLICE/SOLDIER . . . . . 12<br>PRIEST/RELIGIOUS LEADER . . . . . 13<br>STRANGER . . . . . 14<br>NEIGHBOUR . . . . . 15<br><br>OTHER ________ 96<br>(SPECIFY)	
1522C	At any time in your life, as a child or as an adult, has anyone (other than any partner) ever forced you in any way to perform any other sexual acts when you did not want to?	YES . . . . . 1<br>NO . . . . . 2<br>REFUSED TO ANSWER/<br>NO ANSWER 3	 2, 3 → 1526
1522D	How old were you the first time this happened?	AGE IN COMPLETED YEARS [ ][ ]<br><br>DON'T KNOW 98	
1523	Who was the person who was forcing you the very first time this happened?	FATHER/STEP-FATHER . . . . . 04<br>BROTHER/STEP-BROTHER . . . . . 05<br>OTHER RELATIVE . . . . . 06<br>IN-LAW . . . . . 07<br>OWN FRIEND/ACQUAINTANCE . . . 08<br>FAMILY FRIEND . . . . . 09<br>TEACHER . . . . . 10<br>EMPLOYER/SOMEONE AT WORK . I1<br>POLICE/SOLDIER . . . . . 12<br>PRIEST/RELIGIOUS LEADER . . . . . 13<br>STRANGER . . . . . 14<br>NEIGHBOUR . . . . . 15<br><br>OTHER ________ 96<br>(SPECIFY)	
1523A	CHECK 1522: EVER FORCED TO HAVE SEXUAL INTERCOURSE?<br><br>YES ☐ ↓ NO ☐ →		1526

NO.	QUESTIONS AND FILTERS	CODING CATEGORIES	SKIP
1524	CHECK 701, 701A, 701B, 702 AND 1502A:<br><br>EVER IN UNION OR HAD A BOYFRIEND ☐ ↓ | NEVER IN UNION OR HAD A BOYFRIEND ☐ ↓<br><br>a) In the last 12 months, has anyone other than (your/any) partner physically forced you to have sexual intercourse when you did not want to?<br><br>b) In the last 12 months has anyone physically forced you to have sexual intercourse when you did not want to?	YES . . . . . 1<br>NO 2	
1526	CHECK 1505A (a-j), 1515A (a, b, c), 1516, 1520, 1520A, 1522, 1522C AND 1524:<br><br>AT LEAST ONE 'YES' ☐ ↓ NOT A SINGLE 'YES' ☐		→ 1530
1527	Thinking about what you yourself have experienced among the different things we have been talking about, have you ever tried to seek help?	YES . . . . . 1<br>NO 2	 → 1529
1528	From whom have you sought help?<br><br>Anyone else?<br><br>RECORD ALL MENTIONED.	OWN FAMILY . . . . . A<br>HUSBAND'S/PARTNER'S FAMILY . B<br>CURRENT/FORMER<br>HUSBAND/PARTNER . . . . . C<br>CURRENT/FORMER BOYFRIEND . D<br>FRIEND . . . . . E<br>NEIGHBOUR . . . . . F<br>RELIGIOUS LEADER . . . . . G<br>DOCTOR/MEDICAL PERSONNEL . H<br>POLICE . . . . . I<br>LAWYER . . . . . J<br>SOCIAL SERVICE ORGANISATION . K<br>COLLEAGUE . . . . . L<br>HELPLINE . . . . . M<br><br>OTHER ______ X<br>(SPECIFY)	→ 1530
1529	Have you ever told any one about this?	YES . . . . . 1<br>NO 2	
1530	As far as you know, did your father or any other husband or boyfriend your mother had ever hit or beat her?	YES . . . . . 1<br>NO . . . . . 2<br>DON'T KNOW 8	

THANK THE RESPONDENT FOR HER COOPERATION AND REASSURE HER ABOUT THE CONFIDENTIALITY OF HER ANSWERS. FILL OUT THE QUESTIONS BELOW WITH REFERENCE TO THE HOUSEHOLD RELATIONS MODULE ONLY.

NO.	QUESTIONS AND FILTERS		YES ONCE	YES, MORE THAN ONCE	NO	SKIP
1531	DID YOU HAVE TO INTERRUPT THE INTERVIEW BECAUSE SOME ADULT WAS TRYING TO LISTEN, OR CAME INTO THE ROOM, OR INTERFERED IN ANY OTHER WAY?	HUSBAND/PARTNER .	1	2	3	
		OTHER MALE ADULT .	1	2	3	
		FEMALE ADULT	1	2	3	

1532 INTERVIEWER'S COMMENTS / EXPLANATION FOR NOT COMPLETING THE HOUSEHOLD RELATIONS MODULE

____

____

____

NO.	QUESTIONS AND FILTERS	CODING CATEGORIES	SKIP
1533	RECORD THE TIME.	HOURS . . . . ☐☐<br>MINUTES . . . . ☐☐	

INTERVIEWER'S OBSERVATIONS

TO BE FILLED IN AFTER COMPLETING INTERVIEW

COMMENTS ABOUT INTERVIEW:

COMMENTS ON SPECIFIC QUESTIONS:

ANY OTHER COMMENTS:

SUPERVISOR'S OBSERVATIONS

INSTRUCTIONS:

ONLY ONE CODE SHOULD APPEAR IN ANY BOX.
COLUMN 1 REQUIRES A CODE IN EVERY MONTH.

INFORMATION TO BE CODED FOR EACH COLUMN

COLUMN 1: <u>BIRTHS, PREGNANCIES, CONTRACEPTIVE USE</u>

B BIRTHS
P PREGNANCIES
C MISCARRIAGES
A INDUCED ABORTIONS
S STILLBIRTHS

0 NO METHOD

1 FEMALE STERILISATION
2 MALE STERILISATION
3 IUD
4 INJECTABLES - 3 MONTH DEPO
5 INJECTABLES - 2 MONTH NUR-ISTERATE
6 IMPLANTS
7 PILL
8 MALE CONDOM
9 FEMALE CONDOM
E EMERGENCY CONTRACEPTION

L RHYTHM METHOD
M WITHDRAWAL
X OTHER MODERN METHOD
Y OTHER TRADITIONAL METHOD

COLUMN 2: <u>DISCONTINUATION OF CONTRACEPTIVE USE</u>

0 INFREQUENT SEX/HUSBAND AWAY
1 BECAME PREGNANT WHILE USING
2 WANTED TO BECOME PREGNANT
3 HUSBAND/PARTNER DISAPPROVED

4 WANTED MORE EFFECTIVE METHOD
5 SIDE EFFECTS/HEALTH CONCERNS
6 LACK OF ACCESS/TOO FAR
7 COSTS TOO MUCH
8 INCONVENIENT TO USE
F UP TO GOD/FATALISTIC
A DIFFICULT TO GET PREGNANT/MENOPAUSAL
D MARITAL DISSOLUTION/SEPARATION
X OTHER

____
(SPECIFY)

Z DON'T KNOW

				COL. 1	COL. 2	
2016	12	DEC	01			2016
	11	NOV	02			
	10	OCT	03			
	09	SEP	04			
	08	AUG	05			
	07	JUL	06			
	06	JUN	07			
	05	MAY	08			
	04	APR	09			
	03	MAR	10			
	02	FEB	11			
	01	JAN	12			
2015	12	DEC	13			2015
	11	NOV	14			
	10	OCT	15			
	09	SEP	16			
	08	AUG	17			
	07	JUL	18			
	06	JUN	19			
	05	MAY	20			
	04	APR	21			
	03	MAR	22			
	02	FEB	23			
	01	JAN	24			
2014	12	DEC	25			2014
	11	NOV	26			
	10	OCT	27			
	09	SEP	28			
	08	AUG	29			
	07	JUL	30			
	06	JUN	31			
	05	MAY	32			
	04	APR	33			
	03	MAR	34			
	02	FEB	35			
	01	JAN	36			
2013	12	DEC	37			2013
	11	NOV	38			
	10	OCT	39			
	09	SEP	40			
	08	AUG	41			
	07	JUL	42			
	06	JUN	43			
	05	MAY	44			
	04	APR	45			
	03	MAR	46			
	02	FEB	47			
	01	JAN	48			
2012	12	DEC	49			2012
	11	NOV	50			
	10	OCT	51			
	09	SEP	52			
	08	AUG	53			
	07	JUL	54			
	06	JUN	55			
	05	MAY	56			
	04	APR	57			
	03	MAR	58			
	02	FEB	59			
	01	JAN	60			
2011	12	DEC	61			2011
	11	NOV	62			
	10	OCT	63			
	09	SEP	64			
	08	AUG	65			
	07	JUL	66			
	06	JUN	67			
	05	MAY	68			
	04	APR	69			
	03	MAR	70			
	02	FEB	71			
	01	JAN	72			

2016 SOUTH AFRICA DEMOGRAPHIC AND HEALTH SURVEY
MAN'S QUESTIONNAIRE

IDENTIFICATION

PLACE NAME ____________

NAME OF HOUSEHOLD HEAD ____________

CLUSTER NUMBER . . . . [ ][ ][ ][ ]

HOUSEHOLD NUMBER . . . . [ ][ ][ ][ ]

NAME AND LINE NUMBER OF MAN ____________ [ ][ ]

INTERVIEWER VISITS

	1	2	3	FINAL VISIT
DATE	______	______	______	DAY [ ][ ]<br>MONTH [ ][ ]<br>YEAR [2][0][1][ ]
INTERVIEWER'S NAME	______	______	______	INT. NO. [ ][ ][ ][ ]
RESULT*	______	______	______	RESULT* [ ]
NEXT VISIT: DATE	______	______		TOTAL NUMBER OF VISITS [ ]
TIME	______	______		

*RESULT CODES:
1 COMPLETED
2 NOT AT HOME
3 POSTPONED
4 REFUSED
5 PARTLY COMPLETED
6 INCAPACITATED
7 OTHER ____________ (SPECIFY)

LANGUAGE OF QUESTIONNAIRE** [0][1]

LANGUAGE OF INTERVIEW** [ ][ ]

HOME LANGUAGE OF RESPONDENT** [ ][ ]

TRANSLATOR USED (YES = 1, NO = 2) [ ]

LANGUAGE OF QUESTIONNAIRE** **ENGLISH**

**LANGUAGE CODES:

01 ENGLISH	05 seSOTHO	09 tshiVENDA
02 AFRIKAANS	06 seTSWANA	10 xiTSONGA
03 isiXHOSA	07 sePEDI	11 isiNDEBELE
04 isiZULU	08 siSWATI	12 OTHER

SUPERVISOR

______ NAME

[ ][ ][ ][ ] NUMBER

100A	CHECK RESPONDENT'S AGE AND MARITAL STATUS IN HOUSEHOLD QUESTIONNAIRE.	
	AGE 15-17 AND NEVER IN UNION ☐ ↓ AGE 18 AND ABOVE OR AGE 15-17 AND EVER IN UNION ☐	→ 100C

INTRODUCTION AND CONSENT (PARENT/GUARDIAN)

100B
Hello. My name is ______________________________________. I am working with Statistics South Africa, We are conducting a survey about health and other topics all over South Africa. The information we collect will help the government to plan health services. Your household was selected for the survey. I would like to talk to (NAME OF MINOR) about his health and well-being. The questions usually take about 30 to 40 minutes. All of the answers (NAME OF MINOR) gives will be confidential and will not be shared with anyone other than members of our survey team. (NAME OF MINOR) doesn't have to be in the survey, but we hope you will agree to allow (NAME OF MINOR) to answer the questions since (NAME OF MINOR)'s views are important.

In case you need more information about the survey, you may contact the person listed on the card that has already been given to your household.

Do you have any questions?
May I begin the interview with (NAME OF MINOR) now?

SIGNATURE OF INTERVIEWER ______________________________ DATE ________________

PARENT/GUARDIAN AGREES MINOR MAY BE INTERVIEWED . . 1 ↓

PARENT/GUARDIAN DOES NOT AGREE TO ALLOW MINOR TO BE INTERVIEWED . . 2 → END

INTRODUCTION AND CONSENT (RESPONDENT)

100C
Hello. My name is ______________________________________. I am working with Statistics South Africa. We are conducting a survey about health and other topics all over South Africa. The information we collect will help the government to plan health services. Your household was selected for the survey. The questions usually take about 30 to 40 minutes. All of the answers you give will be confidential and will not be shared with anyone other than members of our survey team. You don't have to be in the survey, but we hope you will agree to answer the questions since your views are important. If I ask you any question you don't want to answer, just let me know and I will go on to the next question or you can stop the interview at any time.

In case you need more information about the survey, you may contact the person listed on this information sheet.

GIVE INFORMATION SHEET.

Do you have any questions?
May I begin the interview now?

SIGNATURE OF INTERVIEWER ______________________________ DATE ________________

RESPONDENT AGREES TO BE INTERVIEWED . . 1 ↓

RESPONDENT DOES NOT AGREE TO BE INTERVIEWED . . 2 → END

SECTION 1. RESPONDENT'S BACKGROUND

NO.	QUESTIONS AND FILTERS	CODING CATEGORIES	SKIP
101	RECORD THE TIME.	HOURS . . . . [ ][ ]<br>MINUTES . . . . [ ][ ]	
102	How long have you been living continuously in (NAME OF CURRENT CITY, TOWN OR VILLAGE OF RESIDENCE)?<br><br>IF LESS THAN ONE YEAR, RECORD ‘00’ YEARS.	YEARS . . . . [ ][ ]<br><br>ALWAYS . . . . 95<br>VISITOR 96	95, 96 → 105
103	Just before you moved here, where did you live?<br><br>PROBE: Is that a city, a town, a rural area, a farm, a tribal area, or an informal settlement?	CITY . . . . 1<br>TOWN . . . . 2<br>RURAL AREA . . . . 3<br>FARM . . . . 4<br>TRIBAL AREA . . . . 5<br>INFORMAL SETTLEMENT 6	
104	Before you moved here, which province did you live in?	WESTERN CAPE . . . . 01<br>EASTERN CAPE . . . . 02<br>NORTHERN CAPE . . . . 03<br>FREE STATE . . . . 04<br>KWAZULU-NATAL . . . . 05<br>NORTH WEST . . . . 06<br>GAUTENG . . . . 07<br>MPUMALANGA . . . . 08<br>LIMPOPO . . . . 09<br><br>SADC COUNTRY . . . . 16<br><br>OTHER COUNTRY 26	

NO.	QUESTIONS AND FILTERS	CODING CATEGORIES	SKIP
105	On what day, month, and year were you born?	DAY . . . . [ ][ ]<br>DON'T KNOW DAY . . . . 98<br>MONTH . . . . [ ][ ]<br>DON'T KNOW MONTH . . . . 98<br>YEAR . . . . [ ][ ][ ][ ]<br>DON'T KNOW YEAR 9998	
106	How old were you at your last birthday?<br><br>COMPARE AND CORRECT 105 AND/OR 106 IF INCONSISTENT. IF AGE 95 OR OLDER, RECORD 95.	AGE IN COMPLETED YEARS . . . . [ ][ ]	
106A	Which population group do you consider yourself: black, white, coloured, Indian or something else?	BLACK/AFRICAN . . . . 1<br>WHITE . . . . 2<br>COLOURED . . . . 3<br>INDIAN/ASIAN . . . . 4<br>OTHER ______________________ 6<br>(SPECIFY)	
107	Have you ever attended an educational institution?	YES . . . . 1<br>NO 2	 → 111
108	What is the highest level you attended: primary, secondary, or higher than secondary?	PRIMARY . . . . 1<br>SECONDARY . . . . 2<br>HIGHER THAN SECONDARY 3	
109	What is the highest grade or form you completed at that level?	**PRIMARY SCHOOL**<br>LESS THAN 1 YEAR COMPLETED . . . . 00<br>GRADE 1/SUB A/CLASS 1 . . . . 11<br>GRADE 2/SUB B/CLASS 2 . . . . 12<br>GRADE 3/STANDARD 1/<br>AET 1 (KHA RI GUDE, SANLI) . . . . 13<br>GRADE 4/STANDARD 2 . . . . 14<br>GRADE 5/STANDARD 3/AET 2 . . . . 15<br>GRADE 6 /STANDARD 4 . . . . 16<br>GRADE 7/STANDARD 5/AET 3 . . . . 17<br><br>**SECONDARY SCHOOL**<br>LESS THAN 1 YEAR COMPLETED . . . . 20<br>GRADE 8/STANDARD 6/FORM 1/NTC 1/<br>N1/NC (V) LEVEL 2 . . . . 21<br>GRADE 9/STANDARD 7/FORM 2/AET 4/NTC 2/<br>N2/NC (V) LEVEL 3 . . . . 22<br>GRADE 10/STANDARD 8/FORM 3/NTC 3/<br>N3/NC (V) LEVEL 4 . . . . 23<br>GRADE 11/STANDARD 9/FORM 4 . . . . 24<br>CERTIFICATE OR DIPLOMA WITH LESS THAN<br>GRADE 12/STANDARD 10 COMPLETED . . 25<br>GRADE 12/STANDARD 10/FORM 5/MATRIC . . 26<br>N4/NTC4 . . . . 27<br>N5/NTC5 . . . . 28<br>N6/NTC6 . . . . 29<br><br>**HIGHER EDUCATION**<br>FURTHER STUDIES INCOMPLETE OR ONGOIN 30<br>CERTIFICATE OR DIPLOMA WITH GRADE 12/<br>STANDARD 10 COMPLETED . . . . 31<br>HIGHER DIPLOMA (TECHNIKON/<br>U. OF TECHNOLOGY) . . . . 32<br>POST HIGHER DIPLOMA (TECHNIKON/<br>U. TECHNOLOGY MASTERS, DOCTORAL) 33<br>BACHELORS DEGREE/BACHELORS DEGREE<br>AND POST GRADUATE DIPLOMA . . . . . 34<br>HONOURS DEGREE . . . . 35<br>HIGHER DEGREE (MASTERS, DOCTORATE . . 36	
110	CHECK 108:<br>PRIMARY OR SECONDARY [ ] ↓	HIGHER [ ]	→ 113

SECTION 1. RESPONDENT'S BACKGROUND

NO.	QUESTIONS AND FILTERS	CODING CATEGORIES	SKIP
111	Now I would like you to read this sentence to me.<br><br>SHOW CARD TO RESPONDENT.<br><br>IF RESPONDENT CANNOT READ WHOLE SENTENCE, PROBE: Can you read any part of the sentence to me?	CANNOT READ AT ALL . . . . 1<br>ABLE TO READ ONLY PART OF THE SENTENCE . . . . 2<br>ABLE TO READ WHOLE SENTENCE . . . . . . 3<br>NO CARD WITH REQUIRED LANGUAGE ______ 4<br>(SPECIFY LANGUAGE)<br>BLIND/VISUALLY IMPAIRED 5	
112	CHECK 111:<br><br>CODE '2', '3' OR '4' CIRCLED ↓ CODE '1' OR '5' CIRCLED →		→ 114
113	Do you read a newspaper or magazine at least once a week, less than once a week or not at all?	AT LEAST ONCE A WEEK . . . . 1<br>LESS THAN ONCE A WEEK . . . . 2<br>NOT AT ALL 3	
114	Do you listen to the radio at least once a week, less than once a week or not at all?	AT LEAST ONCE A WEEK . . . . 1<br>LESS THAN ONCE A WEEK . . . . 2<br>NOT AT ALL 3	
115	Do you watch television at least once a week, less than once a week or not at all?	AT LEAST ONCE A WEEK . . . . 1<br>LESS THAN ONCE A WEEK . . . . 2<br>NOT AT ALL 3	
116	Do you own a cell phone?	YES . . . . 1<br>NO 2	 → 118
117	Do you use your cell phone for any financial transactions?	YES . . . . 1<br>NO 2	
118	Do you have an account in a bank or other financial institution that you yourself use?	YES . . . . 1<br>NO 2	
119	Have you ever used the internet?	YES . . . . 1<br>NO 2	 → 124
120	In the last 12 months, have you used the internet?<br><br>IF NECESSARY, PROBE FOR USE FROM ANY LOCATION, WITH ANY DEVICE.	YES . . . . 1<br>NO 2	 → 124
121	During the last one month, how often did you use the internet: almost every day, at least once a week, less than once a week, or not at all?	ALMOST EVERY DAY . . . . 1<br>AT LEAST ONCE A WEEK . . . . 2<br>LESS THAN ONCE A WEEK . . . . 3<br>NOT AT ALL 4	
124	In the last 12 months, how many times have you been away from home for one or more nights?	NUMBER OF TIMES . . . . . . [ ][ ]<br><br>NONE 00	 <br>→ 126
125	In the last 12 months, have you been away from home for more than one month at a time?	YES . . . . 1<br>NO 2	
126	CHECK 106: AGE OF RESPONDENT<br><br>AGE 15-59 ↓ AGE 60 AND ABOVE →		→ 401

SECTION 2. REPRODUCTION

NO.	QUESTIONS AND FILTERS	CODING CATEGORIES	SKIP
201	Now I would like to ask about any children you have had during your life. I am interested in all of the children that are biologically yours, even if they are not legally yours or do not have your last name. Have you ever fathered any children with any woman?	YES . . . 1<br>NO . . . 2<br>DON'T KNOW . . . 8	 2, 8 → 206
202	Do you have any sons or daughters that you have fathered who are now living with you?	YES . . . 1<br>NO . . . 2	 → 204
203	a) How many sons live with you?<br>b) And how many daughters live with you?<br>IF NONE, RECORD '00'.	a) SONS AT HOME . . . [ ][ ]<br>b) DAUGHTERS AT HOME . . . [ ][ ]	
204	Do you have any sons or daughters that you have fathered who are alive but do not live with you?	YES . . . 1<br>NO . . . 2	 → 206
205	a) How many sons are alive but do not live with you?<br>b) And how many daughters are alive but do not live with you?<br>IF NONE, RECORD '00'.	a) SONS ELSEWHERE . . . [ ][ ]<br>b) DAUGHTERS ELSEWHERE . . . [ ][ ]	
206	Have you ever fathered a son or a daughter who was born alive but later died?<br>IF NO, PROBE: Any baby who cried, who made any movement, sound, or effort to breathe, or who showed any other signs of life even if for a very short time?	YES . . . 1<br>NO . . . 2<br>DON'T KNOW . . . 8	 2, 8 → 208
207	a) How many boys have died?<br>b) And how many girls have died?<br>IF NONE, RECORD '00'.	a) BOYS DEAD . . . [ ][ ]<br>b) GIRLS DEAD . . . [ ][ ]	
208	SUM ANSWERS TO 203, 205, AND 207, AND ENTER TOTAL. IF NONE, RECORD '00'.	TOTAL CHILDREN . . . [ ][ ]	
209	CHECK 208:<br>HAS HAD MORE THAN ONE CHILD [ ] ↓<br>HAS HAD ONLY ONE CHILD [ ] → 211<br>HAS NOT HAD ANY CHILDREN [ ] → 301		211<br>301
210	Did all of the children you have fathered have the same biological mother?	YES . . . 1<br>NO . . . 2	
211	CHECK 208:<br>HAS HAD MORE THAN ONE CHILD [ ] ↓ — a) How old were you when your first child was born?<br>HAS HAD ONLY ONE CHILD [ ] ↓ — b) How old were you when your child was born?	AGE IN YEARS . . . [ ][ ]	
212	CHECK 203 AND 205:<br>AT LEAST ONE LIVING CHILD [ ] ↓<br>NO LIVING CHILDREN [ ] → 301		301

NO.	QUESTIONS AND FILTERS	CODING CATEGORIES	SKIP
213	CHECK 203 AND 205:<br>MORE THAN ONE LIVING CHILD ☐ ↓ / ONLY ONE LIVING CHILD ☐ ↓<br>a) How old is your youngest child?<br>b) How old is your child?	AGE IN YEARS . . . . [ ][ ]	
214	CHECK 213:<br>(YOUNGEST) CHILD IS AGE 0-2 YEARS ☐ ↓<br>(YOUNGEST) CHILD IS AGE 3 YEARS OR OLDER ☐		→ 220
215	CHECK 203 AND 205:<br>MORE THAN ONE LIVING CHILD ☐ ↓ / ONLY ONE LIVING CHILD ☐ ↓<br>a) What is the name of your youngest child?<br>b) What is the name of your child?	______________________<br>(NAME OF (YOUNGEST) CHILD)	
216	When (NAME)'s mother was pregnant with (NAME), did she have any antenatal check-ups?	YES . . . . 1<br>NO . . . . 2<br>DON'T KNOW 8	 2, 8 → 218
217	Were you ever present during any of those antenatal check-ups?	PRESENT . . . . 1<br>NOT PRESENT 2	
218	Was (NAME) born in a hospital or health facility?	HOSPITAL/HEALTH FACILITY . . . . 1<br>OTHER 2	
219	When a child has diarrhea, how much should he or she be given to drink: more than usual, about the same as usual, less than usual, or nothing to drink at all?	MORE THAN USUAL . . . . 1<br>ABOUT THE SAME . . . . 2<br>LESS THAN USUAL . . . . 3<br>NOTHING TO DRINK . . . . 4<br>DON'T KNOW 8	
220	CHECK 203:<br>AT LEAST ONE CHILD LIVING WITH HIM ☐ ↓<br>NO CHILDREN LIVING WITH HIM ☐		→ 301
221	Do you have at least one child who is biologically yours and is less than age 18 who lives with you?	YES . . . . 1<br>NO 2	

SECTION 3. CONTRACEPTION

301	Now I would like to talk about family planning - the various ways or methods that a couple can use to delay or avoid a pregnancy. Which ways or methods have you heard about? MARK ALL METHODS DECLARED BY THE RESPONDENT.<br><br>FOR METHODS NOT MENTIONED SPONTANEOUSLY, ASK: Have you ever heard of (METHOD)?	
01	Female Sterilisation/Tubal Ligation/Tubes Cut/Tubes Binded.<br>PROBE: Women can have an operation to avoid having any more children.	YES . . . . . 1<br>NO 2
02	Male Sterilisation/Vasectomy/Tubes Cut/Tubes Binded.<br>PROBE: Men can have an operation to avoid having any more children.	YES . . . . . 1<br>NO 2
03	IUD.<br>PROBE: Women can have a loop or coil placed inside them by a doctor or a nurse which can prevent pregnancy for one or more years.	YES . . . . . 1<br>NO 2
04	Injectables/Depo.<br>PROBE: Women can have an injection by a health provider that stops them from becoming pregnant for one or more months.	YES . . . . . 1<br>NO 2
05	Implants/Norplant/Jadelle.<br>PROBE: Women can have one or more small rods placed in their upper arm by a doctor or nurse which can prevent pregnancy for one or more years.	YES . . . . . 1<br>NO 2
06	Pill.<br>PROBE: Women can take a pill every day to avoid becoming pregnant.	YES . . . . . 1<br>NO 2
07	Male Condom.<br>PROBE: Men can put a rubber sheath on their penis before sexual intercourse.	YES . . . . . 1<br>NO 2
08	Female Condom.<br>PROBE: Women can place a sheath in their vagina before sexual intercourse.	YES . . . . . 1<br>NO 2
09	Emergency Contraception.<br>PROBE: As an emergency measure, within three days after they have unprotected sexual intercourse, women can take special pills to prevent pregnancy.	YES . . . . . 1<br>NO 2
10	Rhythm Method.<br>PROBE: To avoid pregnancy, women do not have sexual intercourse on the days of the month they think they can get pregnant.	YES . . . . . 1<br>NO 2
11	Withdrawal.<br>PROBE: Men can be careful and pull out before climax.	YES . . . . . 1<br>NO 2
12	Have you heard of any other ways or methods that women or men can use to avoid pregnancy?	YES, MODERN METHOD<br>______________ A<br>(SPECIFY)<br>YES, TRADITIONAL METHOD<br>______________ B<br>(SPECIFY)<br>NO . . . . . Y

SECTION 3. CONTRACEPTION

NO.	QUESTIONS AND FILTERS	CODING CATEGORIES	SKIP
302	In the last six months have you:<br>a) Heard about family planning on the radio?<br>b) Seen anything about family planning on the television?<br>c) Read about family planning in a newspaper or magazine?<br>d) Heard about family planning from a community health worker?	YES NO<br>a) RADIO . . . . 1 2<br>b) TELEVISION . . . . 1 2<br>c) NEWSPAPER OR MAGAZINE . . . . . 1 2<br>d) COMMUNITY HEALTH WORKER . . 1 2	
302A	CHECK Q18 IN HOUSEHOLD QUESTIONNAIRE:<br>YES, CURRENTLY ATTENDING SCHOOL ☐ ↓ NO, NOT CURRENTLY ATTENDING SCHOOL ☐		→ 303
302	e) Heard about family planning at school?	YES NO<br>e) SCHOOL 1 2	
303	In the last few months, have you discussed family planning with a health worker or health professional?	YES . . . . 1<br>NO 2	
304	Now I would like to ask you about a woman's risk of pregnancy. From one menstrual period to the next, are there certain days when a woman is more likely to become pregnant when she has sexual relations?	YES . . . . 1<br>NO . . . . 2<br>DON'T KNOW 8	(2, 8) → 306
305	Is this time just before her period begins, during her period, right after her period has ended, or halfway between two periods?	JUST BEFORE HER PERIOD BEGINS . . . . 1<br>DURING HER PERIOD . . . . 2<br>RIGHT AFTER HER PERIOD HAS ENDED . . . . . 3<br>HALFWAY BETWEEN TWO PERIODS . . . . 4<br>OTHER ______________________ 6<br>(SPECIFY)<br>DON'T KNOW 8	
306	After the birth of a child, can a woman become pregnant before her menstrual period has returned?	YES . . . . 1<br>NO . . . . 2<br>DON'T KNOW 8	
307	I will now read you some statements about contraception. Please tell me if you agree or disagree with each one.<br>a) Contraception is a woman's concern and a man should not have to worry about it.<br>b) Women who use contraception may become promiscuous.	AGREE / DIS-AGREE / DK<br>a) CONTRACEPTION WOMAN'S CONCERN 1 2 8<br>b) WOMEN MAY BECOME PROMISCUOUS 1 2 8	

SECTION 4. MARRIAGE AND SEXUAL ACTIVITY

NO.	QUESTIONS AND FILTERS	CODING CATEGORIES	SKIP
401	Are you currently married or living together with someone as if married?	YES, CURRENTLY MARRIED . . . . 1<br>YES, LIVING WITH A PARTNER . . . . 2<br>NO 3	1, 2 → 401B
401A	Do you have a regular girlfriend/partner or fiancée?	YES . . . . 1<br>NO 2	2 → 402
401B	Is this person a woman or a man?	WOMAN . . . . 1<br>MAN . . . . 2<br>INTERSEX OR TRANSGENDERED 3	
401C	CHECK 401: RESPONDENT'S CURRENT MARITAL STATUS<br>401 = 3 ☐ (continue) 401 = 1 OR 2 ☐ → 403A		403A
402	Have you ever been married or lived together with a someone as if married?	YES, FORMERLY MARRIED . . . . 1<br>YES, LIVED WITH A PARTNER . . . . 2<br>NO 3	3 → 403A
403	What is your marital status now: are you widowed, divorced, or separated?	WIDOWED . . . . 1<br>DIVORCED . . . . 2<br>SEPARATED 3	
403A	CHECK 106: AGE OF RESPONDENT<br>AGE 15-59 ☐ (continue) AGE 60 AND ABOVE ☐ → 601		601
403B	CHECK 401 AND 402:<br>401 = 1 OR 2 ☐ (continue)<br>402 = 1 OR 2 ☐ → 410<br>401 = 3 AND 402 = 3 ☐ → 413		410<br>413
404	Is your (spouse/partner) living with you now or is she/he staying elsewhere?	LIVING WITH HIM . . . . 1<br>STAYING ELSEWHERE 2	
404A	CHECK 401B: SEX OF SPOUSE/PARTNER<br>SPOUSE/PARTNER IS MALE OR INTERSEX (401b = 2 OR 3) ☐ (continue) SPOUSE/PARTNER IS FEMALE (401B = 1) ☐ → 405		405
404B	RECORD THE SPOUSE'S/PARTNER'S NAME AND LINE NUMBER FROM THE HOUSEHOLD QUESTIONNAIRE. IF HE IS NOT LISTED IN THE HOUSEHOLD, RECORD '00'.	NAME ______________<br>LINE NO. . . . . [ ][ ]	→ 410
405	Do you have other wives or do you live with other women as if married?	YES (MORE THAN ONE WIFE) . . . . 1<br>NO (ONLY ONE WIFE) 2	2 → 407
406	Altogether, how many wives or live-in partners do you have?	TOTAL NUMBER OF WIVES AND LIVE-IN PARTNERS . . . . [ ][ ]	

NO.	QUESTIONS AND FILTERS	NAME	LINE NUMBER	408 How old was (NAME) on her last birthday? AGE
407	CHECK 405:<br>ONE WIFE/PARTNER ☐ MORE THAN ONE WIFE/PARTNER ☐<br>a) Please tell me the name of (your wife/the woman you are living with as if married).<br>b) Please tell me the name of each of your wives or each woman you are living with as if married.			
	RECORD THE NAME AND THE LINE NUMBER FROM THE HOUSEHOLD QUESTIONNAIRE FOR EACH WIFE AND LIVE-IN PARTNER.	______	[ ][ ]	[ ][ ]
	IF A WOMAN IS NOT LISTED IN THE HOUSEHOLD, RECORD '00'.	______	[ ][ ]	[ ][ ]
408	ASK 408 FOR EACH PERSON.	______	[ ][ ]	[ ][ ]

NO.	QUESTIONS AND FILTERS	CODING CATEGORIES	SKIP
409	CHECK 405:<br>ONE WIFE/ PARTNER (405 = 2) ☐ ↓<br>MORE THAN ONE WIFE/ PARTNER (405 = 1) ☐ → 411b		411b
410	Have you been married or lived with someone only once or more than once?	MORE THAN ONCE . . . . 1<br>ONLY ONCE 2	
411	CHECK 405 AND 410:<br>405 ≠ 1 and 410 = 2 ☐ ↓<br>a) In what month and year did you start living with your (spouse/partner)?<br>OTHER ☐ ↓<br>b) Now I would like to ask about your first (spouse/partner). In what month and year did you start living with your first (spouse/partner)?	MONTH . . . . ☐☐<br>DON'T KNOW MONTH . . . . 98<br>YEAR . . . . ☐☐☐☐<br>DON'T KNOW YEAR 9998	→ 413
412	How old were you when you first started living together?	AGE . . . . ☐☐	
413	**CHECK FOR PRESENCE OF OTHERS. BEFORE CONTINUING, MAKE EVERY EFFORT TO ENSURE PRIVACY.**		
414	Now I would like to ask some questions about sexual activity in order to gain a better understanding of some important life issues. Let me assure you again that your answers are completely confidential and will not be told to anyone. If we should come to any question that you don't want to answer, just let me know and we will go to the next question. How old were you when you had sexual intercourse for the very first time?	NEVER HAD SEXUAL INTERCOURSE . . . . 00<br>AGE IN YEARS . . . . ☐☐	→ 501
415	I would like to ask you about your recent sexual activity. When was the last time you had sexual intercourse?<br>IF LESS THAN 12 MONTHS, ANSWER MUST BE RECORDED IN DAYS, WEEKS OR MONTHS. IF 12 MONTHS (ONE YEAR) OR MORE, ANSWER MUST BE RECORDED IN YEARS.	DAYS AGO . . . . 1 ☐☐<br>WEEKS AGO . . . . 2 ☐☐<br>MONTHS AGO . . . . 3 ☐☐<br>YEARS AGO . . . . 4 ☐☐	DAYS/WEEKS/MONTHS → 417<br>YEARS → 427

		LAST SEXUAL PARTNER	SECOND-TO-LAST SEXUAL PARTNER	THIRD-TO-LAST SEXUAL PARTNER
416	When was the last time you had sexual intercourse with this person?		DAYS AGO . . 1 [ ][ ]<br>WEEKS AGO . . 2 [ ][ ]<br>MONTHS AGO . . 3 [ ][ ]	DAYS AGO . . 1 [ ][ ]<br>WEEKS AGO . . 2 [ ][ ]<br>MONTHS AGO . . 3 [ ][ ]
417	The last time you had sexual intercourse with this person, was a condom used?	YES . . . . 1<br>NO . . . . 2<br>(SKIP TO 419)	YES . . . . 1<br>NO . . . . 2<br>(SKIP TO 419)	YES . . . . 1<br>NO . . . . 2<br>(SKIP TO 419)
418	Was a condom used every time you had sexual intercourse with this person in the last 12 months?	YES . . . . 1<br>NO 2	YES . . . . 1<br>NO 2	YES . . . . 1<br>NO 2
419	What was your relationship to this person with whom you had sexual intercourse?<br><br>IF GIRLFRIEND/BOYFRIEND: Were you living together as if married?<br><br>IF YES, RECORD '2'.<br>IF NO, RECORD '3'.	SPOUSE . . . . 1<br>LIVE-IN PARTNER . . . . . 2<br>GIRLFRIEND/BOYFRIEND NOT LIVING WITH RESPONDENT . . . . . 3<br>CASUAL ACQUAINTANCE . . 4<br>CLIENT/SEX WORKER . . 5<br>OTHER ________ 6<br>(SPECIFY)	SPOUSE . . . . 1<br>LIVE-IN PARTNER . . . . . 2<br>GIRLFRIEND/BOYFRIEND NOT LIVING WITH RESPONDENT . . . . . 3<br>CASUAL ACQUAINTANCE . . 4<br>CLIENT/SEX WORKER . . 5<br>OTHER ________ 6<br>(SPECIFY)	SPOUSE . . . . 1<br>LIVE-IN PARTNER . . . . . 2<br>GIRLFRIEND/BOYFRIEND NOT LIVING WITH RESPONDENT . . . . . 3<br>CASUAL ACQUAINTANCE . . 4<br>CLIENT/SEX WORKER . . 5<br>OTHER ________ 6<br>(SPECIFY)
420	How long ago did you first have sexual intercourse with this person?	DAYS AGO . . 1 [ ][ ]<br>WEEKS AGO . . 2 [ ][ ]<br>MONTHS AGO . . 3 [ ][ ]<br>YEARS AGO . . 4 [ ][ ]	DAYS AGO . . 1 [ ][ ]<br>WEEKS AGO . . 2 [ ][ ]<br>MONTHS AGO . . 3 [ ][ ]<br>YEARS AGO . . 4 [ ][ ]	DAYS AGO . . 1 [ ][ ]<br>WEEKS AGO . . 2 [ ][ ]<br>MONTHS AGO . . 3 [ ][ ]<br>YEARS AGO . . 4 [ ][ ]
421	How many times during the last 12 months did you have sexual intercourse with this person?<br><br>IF NON-NUMERIC ANSWER, PROBE TO GET AN ESTIMATE. IF NUMBER OF TIMES IS 95 OR MORE, RECORD '95'.	NUMBER OF TIMES . . . . . [ ][ ]	NUMBER OF TIMES . . . . . [ ][ ]	NUMBER OF TIMES . . . . . [ ][ ]
422	How old is this person?	AGE OF PARTNER [ ][ ]<br>DON'T KNOW 98	AGE OF PARTNER [ ][ ]<br>DON'T KNOW 98	AGE OF PARTNER [ ][ ]<br>DON'T KNOW 98
423	Apart from this person, have you had sexual intercourse with any other person in the last 12 months?	YES . . . . 1<br>(GO BACK TO 416 IN NEXT COLUMN)<br>NO . . . . 2<br>(SKIP TO 425)	YES . . . . 1<br>(GO BACK TO 416 IN NEXT COLUMN)<br>NO . . . . 2<br>(SKIP TO 425)	
424	In total, with how many different people have you had sexual intercourse in the last 12 months?<br><br>IF NON-NUMERIC ANSWER, PROBE TO GET AN ESTIMATE. IF NUMBER OF PARTNERS IS 95 OR MORE, RECORD '95'.			NUMBER OF PARTNERS LAST 12 MONTHS . . [ ][ ]<br>DON'T KNOW 98

SECTION 4. MARRIAGE AND SEXUAL ACTIVITY

NO.	QUESTIONS AND FILTERS	CODING CATEGORIES	SKIP
425	CHECK 419 (ALL COLUMNS):<br>AT LEAST ONE PARTNER IS A SEX WORKER ☐ ↓<br>NO PARTNERS ARE SEX WORKERS ☐ → 427		→ 427
426	CHECK 419 AND 417 (ALL COLUMNS):<br>CONDOM USED WITH EVERY SEX WORKER ☐ → 430<br>OTHER ☐ → 431		430<br>431
427	In the last 12 months, did you pay anyone in exchange for having sexual intercourse?	YES . . . . . 1<br>NO 2	1 → 429
428	Have you ever paid anyone in exchange for having sexual intercourse?	YES . . . . . 1<br>NO 2	1, 2 → 431
429	The last time you paid someone in exchange for having sexual intercourse, was a condom used?	YES . . . . . 1<br>NO 2	2 → 431
430	Was a condom used during sexual intercourse every time you paid someone in exchange for having sexual intercourse in the last 12 months?	YES . . . . . 1<br>NO . . . . . 2<br>DON'T KNOW 8	
431	In the past 12 months have you given any gifts or other goods in order to have sex or to become sexually involved with anyone?	YES . . . . . 1<br>NO 2	1 → 433
432	Have you ever given any gifts or other goods in order to have sex or to become sexually involved with anyone?	YES . . . . . 1<br>NO 2	
433	In total, with how many different people have you had sexual intercourse in your lifetime?<br><br>IF NON-NUMERIC ANSWER, PROBE TO GET AN ESTIMATE. IF NUMBER OF PARTNERS IS 95 OR MORE, RECORD '95'.	NUMBER OF PARTNERS IN LIFETIME . . . . . [ ][ ]<br><br>DON'T KNOW 98	
434	CHECK 417: MOST RECENT PARTNER (FIRST COLUMN)<br>NOT ASKED ☐ → 438<br>CONDOM USED ☐ ↓<br>NO CONDOM USED ☐ → 438		438<br>438
437	The last time you had sex did you or your partner use any method other than a condom to avoid or prevent a pregnancy?	YES . . . . . 1<br>NO . . . . . 2<br>DON'T KNOW 8	1 → 439<br>2, 8 → 440
438	The last time you had sex did you or your partner use any method to avoid or prevent a pregnancy?	YES . . . . . 1<br>NO . . . . . 2<br>DON'T KNOW 8	2, 8 → 440
439	What method did you or your partner use?<br><br>PROBE: Did you or your partner use any other method to prevent pregnancy?<br><br>RECORD ALL MENTIONED.	FEMALE STERILISATION . . . . . A<br>MALE STERILISATION . . . . . B<br>IUD . . . . . C<br>INJECTABLES . . . . . D<br>IMPLANTS . . . . . E<br>PILL . . . . . F<br>CONDOM . . . . . G<br>FEMALE CONDOM . . . . . H<br>EMERGENCY CONTRACEPTION . . . . . I<br>RHYTHM METHOD . . . . . J<br>WITHDRAWAL . . . . . K<br>OTHER MODERN METHOD . . . . . X<br>OTHER TRADITIONAL METHOD . . . . . Y	→ 501
440	Do you know of a place where you can obtain a method of family planning?	YES . . . . . 1<br>NO 2	

SECTION 5. FERTILITY PREFERENCES

NO.	QUESTIONS AND FILTERS	CODING CATEGORIES	SKIP
501	CHECK 401, 401A AND 401B:<br>CURRENTLY MARRIED OR LIVING WITH A WOMAN OR HAS REGULAR FEMALE PARTNER/GIRLFRIEND ☐ ↓<br>NOT IN UNION WITH A WOMAN ☐ → 514		514
502	CHECK 439:<br>MAN NOT STERILISED ☐ ↓<br>MAN STERILISED ☐ → 514		514
503	CHECK 407:<br>ONE WIFE/ PARTNER ☐ ↓<br>MORE THAN ONE WIFE/ PARTNER ☐ → 509		509
504	Is your (wife/partner) currently pregnant?	YES . . . . . 1<br>NO . . . . . 2<br>DON'T KNOW 8	 2, 8 → 507
505	Now I have some questions about the future. After the child you and your (wife/partner) are expecting now, would you like to have another child, or would you prefer not to have any more children?	HAVE ANOTHER CHILD . . . . . 1<br>NO MORE . . . . . 2<br>UNDECIDED/DON'T KNOW 8	 2, 8 → 514
506	After the birth of the child you are expecting now, how long would you like to wait before the birth of another child?	MONTHS . . . . . 1 ☐☐<br>YEARS . . . . . 2 ☐☐<br>SOON/NOW . . . . . 993<br>OTHER ______ 996<br>(SPECIFY)<br>DON'T KNOW 998	→ 514
507	CHECK 208:<br>HAS FATHERED CHILDREN ☐ ↓<br>a) Now I have some questions about the future. Would you like to have another child, or would you prefer not to have any more children?<br>HAS NOT FATHERED CHILDREN ☐ ↓<br>b) Now I have some questions about the future. Would you like to have a child, or would you prefer not to have any children?	HAVE (A/ANOTHER) CHILD . . . . . 1<br>NO MORE/NONE . . . . . 2<br>SAYS COUPLE CAN'T GET PREGNANT 3<br>WIFE/PARTNER STERILIZED . . . . . 4<br>UNDECIDED/DON'T KNOW 8	 2, 3, 4, 8 → 514
508	CHECK 208:<br>HAS FATHERED CHILDREN ☐ ↓<br>a) How long would you like to wait from now before the birth of another child?<br>HAS NOT FATHERED CHILDREN ☐ ↓<br>b) How long would you like to wait from now before the birth of a child?	MONTHS . . . . . 1 ☐☐<br>YEARS . . . . . 2 ☐☐<br>SOON/NOW . . . . . 993<br>SAYS COUPLE CAN'T GET PREGNANT . . . . . 994<br>OTHER ______ 996<br>(SPECIFY)<br>DON'T KNOW 998	→ 514
509	Are any of your (wives/partners) currently pregnant?	YES . . . . . 1<br>NO . . . . . 2<br>DON'T KNOW 8	 2, 8 → 512

SECTION 5. FERTILITY PREFERENCES

<table>
<tr><th>NO.</th><th>QUESTIONS AND FILTERS</th><th>CODING CATEGORIES</th><th>SKIP</th></tr>
<tr><td>510</td><td>Now I have some questions about the future. After the (child/children) you and your (wives/partners) are expecting now, would you like to have another child, or would you prefer not to have any more children?</td><td>HAVE ANOTHER CHILD 1
NO MORE 2
UNDECIDED/DON'T KNOW . . . . 8</td><td>2, 8 → 514</td></tr>
<tr><td>511</td><td>After the birth of the child you are expecting now, how long would you like to wait before the birth of another child?</td><td>MONTHS . . . . 1 [ ][ ]
YEARS . . . . 2 [ ][ ]
SOON/NOW 993
OTHER ______________ 996
(SPECIFY)
DON'T KNOW . . . . 998</td><td>→ 514</td></tr>
<tr><td>512</td><td>CHECK 208:
HAS FATHERED CHILDREN [ ] ↓ | HAS NOT FATHERED CHILDREN [ ] ↓
a) Now I have some questions about the future. Would you like to have another child, or would you prefer not to have any more children?
b) Now I have some questions about the future. Would you like to have a child, or would you prefer not to have any children?</td><td>HAVE (A/ANOTHER) CHILD 1
NO MORE/NONE 2
SAYS COUPLE CAN'T GET PREGNANT 3
WIFE (WIVES)/PARTNER(S) STERILIZED 4
UNDECIDED/DON'T KNOW . . . . 8</td><td>2, 3, 4, 8 → 514</td></tr>
<tr><td>513</td><td>CHECK 208:
HAS FATHERED CHILDREN [ ] ↓ | HAS NOT FATHERED CHILDREN [ ] ↓
a) How long would you like to wait from now before the birth of another child?
b) How long would you like to wait from now before the birth of a child?</td><td>MONTHS . . . . 1 [ ][ ]
YEARS . . . . 2 [ ][ ]
SOON/NOW 993
SAYS COUPLE
CAN'T GET PREGNANT 994
OTHER ______________ 996
(SPECIFY)
DON'T KNOW . . . . 998</td><td></td></tr>
<tr><td>514</td><td>CHECK 203 AND 205:
HAS LIVING CHILDREN [ ] ↓ | NO LIVING CHILDREN [ ] ↓
a) If you could go back to the time you did not have any children and could choose exactly the number of children to have in your whole life, how many would that be?
b) If you could choose exactly the number of children to have in your whole life, how many would that be?
PROBE FOR A NUMERIC RESPONSE.</td><td>NONE 00
NUMBER . . . . [ ][ ]
OTHER ______________ 96
(SPECIFY)</td><td>00 → 601
96 → 601</td></tr>
<tr><td>515</td><td>How many of these children would you like to be boys, how many would you like to be girls and for how many would it not matter if it's a boy or a girl?</td><td>BOYS [ ][ ] GIRLS [ ][ ] EITHER [ ][ ]
NUMBER . .
OTHER ______________ 96
(SPECIFY)</td><td></td></tr>
</table>

SECTION 6. EMPLOYMENT AND GENDER ROLES

NO.	QUESTIONS AND FILTERS	CODING CATEGORIES	SKIP
601	Have you done any work in the last seven days?	YES . . . . . 1<br>NO 2	1 → 604
602	Although you did not work in the last seven days, do you have any job or business from which you were absent for leave, illness, vacation, or any other such reason?	YES . . . . . 1<br>NO 2	1 → 604
603	Have you done any work in the last 12 months?	YES . . . . . 1<br>NO 2	2 → 604A
604	What is your occupation? That is, what kind of work do you mainly do?	________________<br>________________<br>[ ][ ][ ][ ]	
604A	CHECK 106: AGE OF RESPONDENT<br>AGE 15-59 ☐ ↓<br>AGE 60 AND ABOVE ☐ → 901		901
604B	CHECK 601, 602, 603: ANY YES?<br>YES ☐ ↓<br>NO ☐ → 607		607
605	Do you usually work throughout the year, or do you work seasonally, or only once in a while?	THROUGHOUT THE YEAR . . . . . 1<br>SEASONALLY/PART OF THE YEAR . . . . . 2<br>ONCE IN A WHILE 3	
606	Are you paid in cash or kind for this work or are you not paid at all?	CASH ONLY . . . . . 1<br>CASH AND KIND . . . . . 2<br>IN KIND ONLY . . . . . 3<br>NOT PAID 4	
607	CHECK 401, 401A AND 401B:<br>CURRENTLY MARRIED OR LIVING WITH A WOMAN OR HAS REGULAR FEMALE PARTNER/GIRLFRIEND ☐ ↓<br>NOT IN UNION OR IN UNION, BUT NOT WITH A WOMAN ☐ → 612		612
608	CHECK 606:<br>CODE '1' OR '2' CIRCLED ☐ ↓<br>OTHER ☐ → 610		610
609	Who usually decides how the money you earn will be used: you, your (wife/partner), or you and your (wife/partner) jointly?	RESPONDENT . . . . . 1<br>WIFE/PARTNER . . . . . 2<br>RESPONDENT AND WIFE/PARTNER JOINTLY . . 3<br>OTHER ________________ 6<br>(SPECIFY)	
610	Who usually makes decisions about health care for yourself: you, your (wife/partner), you and your (wife/partner) jointly, or someone else?	RESPONDENT . . . . . 1<br>WIFE/PARTNER . . . . . 2<br>RESPONDENT AND WIFE/PARTNER JOINTLY . . 3<br>SOMEONE ELSE . . . . . 4<br>OTHER 6	
611	Who usually makes decisions about making major household purchases?	RESPONDENT . . . . . 1<br>WIFE/PARTNER . . . . . 2<br>RESPONDENT AND WIFE/PARTNER JOINTLY . . 3<br>SOMEONE ELSE . . . . . 4<br>OTHER 6	

SECTION 6. EMPLOYMENT AND GENDER ROLES

NO.	QUESTIONS AND FILTERS	CODING CATEGORIES	SKIP
612	Do you own this or any other house either alone or jointly with someone else?	ALONE ONLY . . . 1<br>JOINTLY ONLY . . . 2<br>BOTH ALONE AND JOINTLY . . . 3<br>DOES NOT OWN . . . 4	 <br><br>4 → 618
613	Do you have a title deed or documents for any house you own?	YES . . . 1<br>NO . . . 2<br>DON'T KNOW . . . 8	 2, 8 → 618
614	Is your name on the title deed or documents?	YES . . . 1<br>NO . . . 2<br>DON'T KNOW . . . 8	
618	In your opinion, is a husband justified in hitting or beating his wife in the following situations:<br>a) If she goes out without telling him?<br>b) If she neglects the children?<br>c) If she argues with him?<br>d) If she refuses to have sex with him?<br>e) If she burns the food?	YES / NO / DK<br>a) GOES OUT . . . 1 / 2 / 8<br>b) NEGLECTS CHILDREN . . 1 / 2 / 8<br>c) ARGUES . . . 1 / 2 / 8<br>d) REFUSES SEX . . . 1 / 2 / 8<br>e) BURNS FOOD . . . 1 / 2 / 8	
619	CHECK 203 AND 221:<br>ONE OR MORE CHILDREN LESS THAN AGE 18 LIVING WITH HIM ☐ ↓<br>NO CHILDREN OR NO CHILDREN LESS THAN AGE 18 LIVING WITH HIM ☐		→ 701
620	Now I would like to ask you questions about how you discipline or punish your (child/children). In the past 12 months, have you ever:<br>a) Hit or slapped your (child/children) with your hand to punish or discipline the child?<br>b) Hit or beat your (child/children) using a belt, spoon, stick, shoe or any other implement to punish or discipline the child?	YES / NO<br>a) HIT WITH HAND . . . 1 / 2<br>b) HIT WITH IMPLEMENT . . . 1 / 2	

SECTION 7. HIV/AIDS

NO.	QUESTIONS AND FILTERS	CODING CATEGORIES	SKIP
701	Now I would like to talk about something else. Have you ever heard of HIV or AIDS?	YES . . . . 1<br>NO 2	 2 → 727
708	Can HIV be transmitted from a mother to her baby:<br><br>a) During pregnancy?<br>b) During delivery?<br>c) By breastfeeding?	YES / NO / DK<br>a) DURING PREGNANCY . . 1 / 2 / 8<br>b) DURING DELIVERY . . . . . 1 / 2 / 8<br>c) BREASTFEEDING . . . . . 1 / 2 / 8	
709	CHECK 708:<br>AT LEAST ONE 'YES' ☐ ↓ OTHER ☐ → 711		→ 711
710	Are there any special drugs that a doctor or a nurse can give to a woman infected with HIV to reduce the risk of transmission to the baby?	YES . . . . 1<br>NO . . . . 2<br>DON'T KNOW 8	
711	**CHECK FOR PRESENCE OF OTHERS. BEFORE CONTINUING, MAKE EVERY EFFORT TO ENSURE PRIVACY.**		
712	I don't want to know the results, but have you ever been tested for HIV?	YES . . . . 1<br>NO 2	 2 → 716
713	How many months ago was your most recent HIV test?	MONTHS AGO . . . . ☐☐<br>TWO OR MORE YEARS 95	
714	I don't want to know the results, but did you get the results of the test?	YES . . . . 1<br>NO 2	
715	Where was the test done?<br><br>PROBE TO IDENTIFY THE TYPE OF SOURCE.<br><br>IF UNABLE TO DETERMINE IF PUBLIC OR PRIVATE SECTOR, RECORD 96 AND WRITE THE NAME OF THE PLACE.	**PUBLIC SECTOR**<br>GOVERNMENT HOSPITAL . . . . 11<br>GOVERNMENT CLINIC/COMMUNITY HEALTH CENTRE . . . . 12<br>MOBILE/TEMPORARY HCT SERVICES . . . . 13<br>OTHER PUBLIC SECTOR<br>__________ 16<br>(SPECIFY)<br>**PRIVATE MEDICAL SECTOR**<br>PRIVATE HOSPITAL/CLINIC/ PRIVATE DOCTOR . . . . 21<br>NEW START TESTING SITE . . . . 22<br>CHEMIST/PHARMACY . . . . 23<br>OTHER PRIVATE MEDICAL SECTOR<br>__________ 26<br>(SPECIFY)<br>**OTHER SOURCE**<br>HOME . . . . 31<br>WORKPLACE . . . . 32<br>CORRECTIONAL FACILITY . . . . 33<br>OTHER __________ 96<br>(SPECIFY)	→ 718

NO.	QUESTIONS AND FILTERS	CODING CATEGORIES	SKIP
716	Do you know of a place where people can go to get an HIV test?	YES . . . 1<br>NO . . . 2	 →718
717	Where is that?<br><br>Any other place?<br><br>PROBE TO IDENTIFY THE TYPE OF SOURCE.<br><br>IF UNABLE TO DETERMINE IF PUBLIC OR PRIVATE SECTOR, RECORD 'X' AND WRITE THE NAME OF THE PLACE(S).	**PUBLIC SECTOR**<br>GOVERNMENT HOSPITAL . . . A<br>GOVERNMENT CLINIC/COMMUNITY HEALTH CENTRE . . . B<br>MOBILE/TEMPORARY HCT SERVICES . . . C<br>OTHER PUBLIC SECTOR<br>__________ D<br>(SPECIFY)<br><br>**PRIVATE MEDICAL SECTOR**<br>PRIVATE HOSPITAL/CLINIC/ PRIVATE DOCTOR . . . E<br>NEW START TESTING SITE . . . F<br>CHEMIST/PHARMACY . . . G<br><br>OTHER PRIVATE MEDICAL SECTOR<br>__________ H<br>(SPECIFY)<br><br>OTHER __________ X<br>(SPECIFY)	
718	Have you heard of test kits people can use to test themselves for HIV?	YES . . . 1<br>NO . . . 2	 →727
719	Have you ever tested yourself for HIV using a self-test kit?	YES . . . 1<br>NO . . . 2	
727	CHECK 701:<br><br>HEARD ABOUT HIV OR AIDS ☐ ↓<br>a) Apart from HIV, have you heard about other infections that can be transmitted through sexual contact?<br><br>NOT HEARD ABOUT HIV OR AIDS ☐ ↓<br>b) Have you heard about infections that can be transmitted through sexual contact?	YES . . . 1<br>NO . . . 2	
728	CHECK 414:<br><br>HAS HAD SEXUAL INTERCOURSE ☐ ↓<br>NEVER HAD SEXUAL INTERCOURSE ☐ →		738
729	CHECK 727: HEARD ABOUT OTHER SEXUALLY TRANSMITTED INFECTIONS?<br><br>YES ☐ ↓<br>NO ☐ →		731
730	Now I would like to ask you some questions about your health in the last 12 months. During the last 12 months, have you had a disease which you got through sexual contact?	YES . . . 1<br>NO . . . 2<br>DON'T KNOW . . . 8	
731	Sometimes men experience an abnormal discharge from their penis. During the last 12 months, have you had an abnormal discharge from your penis?	YES . . . 1<br>NO . . . 2<br>DON'T KNOW . . . 8	
732	Sometimes men have a sore or ulcer on or near their penis. During the last 12 months, have you had a sore or ulcer on or near your penis?	YES . . . 1<br>NO . . . 2<br>DON'T KNOW . . . 8	

NO.	QUESTIONS AND FILTERS	CODING CATEGORIES	SKIP
733	CHECK 730, 731 AND 732:<br>HAS HAD AN INFECTION (ANY 'YES') ☐	HAS NOT HAD AN INFECTION OR DOES NOT KNOW ☐	→ 738
734	The last time you had (PROBLEM FROM 730/731/732), did you seek any kind of advice or treatment?	YES . . . 1<br>NO . . . 2	 2 → 738
735	Where did you go?<br><br>Any other place?<br><br>PROBE TO IDENTIFY THE TYPE OF SOURCE.<br><br>IF UNABLE TO DETERMINE IF PUBLIC OR PRIVATE SECTOR, RECORD 'X' AND WRITE THE NAME OF THE PLACE(S).	**PUBLIC SECTOR**<br>GOVERNMENT HOSPITAL . . . A<br>GOVERNMENT CLINIC/COMMUNITY HEALTH CENTRE . . . B<br>MOBILE/TEMPORARY HCT SERVICES . . . C<br>OTHER PUBLIC SECTOR<br>________ D<br>(SPECIFY)<br><br>**PRIVATE MEDICAL SECTOR**<br>PRIVATE HOSPITAL/CLINIC/ PRIVATE DOCTOR . . . E<br>NEW START TESTING SITE . . . F<br>CHEMIST/PHARMACY . . . G<br><br>OTHER PRIVATE MEDICAL SECTOR<br>________ H<br>(SPECIFY)<br>**OTHER SOURCE**<br>SHOP . . . I<br>TRADITIONAL HERBALIST . . . J<br>TRADITIONAL HEALER . . . K<br><br>OTHER ________ X<br>(SPECIFY)	
738	Some men are circumcised, that is, the foreskin is completely removed from the penis. Are you circumcised?	YES . . . 1<br>NO . . . 2<br>DON'T KNOW . . . 8	 2, 8 → 901
739	How old were you when you got circumcised?	AGE IN COMPLETED YEARS . . . [ ][ ]<br><br>DURING CHILDHOOD (<5 YEARS) . . . 95<br>DON'T KNOW . . . 98	
740	Who did the circumcision?	TRADITIONAL PRACTITIONER/FAMILY/FRIEND 1<br>HEALTH WORKER/PROFESSIONAL . . . 2<br>OTHER . . . 6<br>DON'T KNOW . . . 8	

SECTION 9. TOBACCO AND ALCOHOL

NO.	QUESTIONS AND FILTERS	CODING CATEGORIES	SKIP
901	Would you say your health is poor, average, good, or excellent?	POOR . . . 1<br>AVERAGE . . . 2<br>GOOD . . . 3<br>EXCELLENT . . . 4	
902	Do you personally think you are underweight, normal weight, overweight, or obese?	UNDERWEIGHT . . . 1<br>NORMAL WEIGHT . . . 2<br>OVERWEIGHT . . . 3<br>OBESE . . . 4<br>DON'T KNOW . . . 8	
903	Do you currently smoke tobacco every day, some days, or not at all?	EVERY DAY . . . 1<br>SOME DAYS . . . 2<br>NOT AT ALL . . . 3	1 → 906<br>3 → 905
904	In the past, have you smoked tobacco every day?	YES . . . 1<br>NO . . . 2	→ 907
905	In the past, have you ever smoked tobacco every day, some days, or not at all?	EVERY DAY . . . 1<br>SOME DAYS . . . 2<br>NOT AT ALL . . . 3	→ 908
906	On average, how many of the following products do you currently smoke each day? Also, let me know if you use the product, but not every day.<br><br>IF RESPONDENT REPORTS USING THE PRODUCT BUT NOT EVERY DAY, RECORD '888'. IF THE PRODUCT IS NOT USED AT ALL, RECORD '000'.<br><br>a) Manufactured cigarettes?<br>b) Hand-rolled cigarettes?<br>c) Pipes full of tobacco?<br>d) Cigars or cigarillos?<br>e) Number of hookah, hubbly-bubbly or water pipe sessions?<br>f) Any others?<br>____________ (SPECIFY)	NUMBER DAILY<br><br>a) MANUFACT. CIGARETTES [ ][ ][ ]<br>b) HAND-ROLLED CIGARETTES [ ][ ][ ]<br>c) PIPES FULL OF TOBACCO [ ][ ][ ]<br>d) CIGARS OR CIGARILLOS [ ][ ][ ]<br>e) WATER PIPE SESSIONS [ ][ ][ ]<br>f) OTHERS . . . [ ][ ][ ]	→ 908
907	On average, how many of the following products do you currently smoke each week? Also, let me know if you use the product, but not every week.<br><br>IF RESPONDENT REPORTS USING THE PRODUCT BUT NOT EVERY WEEK, RECORD '888'. IF THE PRODUCT IS NOT USED AT ALL, RECORD '000'.<br><br>a) Manufactured cigarettes?<br>b) Hand-rolled cigarettes?<br>c) Pipes full of tobacco?<br>d) Cigars or cigarillos?<br>e) Number of hookah, hubbly-bubbly or water pipe sessions?<br>f) Any others?<br>____________ (SPECIFY)	NUMBER WEEKLY<br><br>a) MANUFACT. CIGARETTES [ ][ ][ ]<br>b) HAND-ROLLED CIGARETTES [ ][ ][ ]<br>c) PIPES FULL OF TOBACCO [ ][ ][ ]<br>d) CIGARS OR CIGARILLOS [ ][ ][ ]<br>e) WATER PIPE SESSIONS [ ][ ][ ]<br>f) OTHERS . . . [ ][ ][ ]	

SECTION 9. TOBACCO AND ALCOHOL

NO.	QUESTIONS AND FILTERS	CODING CATEGORIES	SKIP
908	Do you currently use snuff, chewing tobacco, or other smokeless tobacco product every day, some days, or not at all?	EVERY DAY . . . . . 1<br>SOME DAYS . . . . . 2<br>NOT AT ALL 3	1 → 910<br>2 → 911
909	In the past, have you used snuff, chewing tobacco or other smokeless tobacco products every day, some days, or not at all?	EVERY DAY . . . . . 1<br>SOME DAYS . . . . . 2<br>NOT AT ALL 3	1, 2, 3 → 912
910	On average, how many times a day do you use the following products? Also, let me know if you use the product, but not every day.<br><br>IF RESPONDENT REPORTS USING THE PRODUCT BUT NOT EVERY DAY, RECORD '888'. IF THE PRODUCT IS NOT USED AT ALL, RECORD '000'.<br><br>a) Snuff, by mouth?<br>b) Snuff, by nose?<br>c) Chewing tobacco?<br>d) Any others?<br>__________ (SPECIFY)	TIMES DAILY<br>a) SNUFF, BY MOUTH . . . . . ☐☐☐<br>b) SNUFF, BY NOSE . . . . . ☐☐☐<br>c) CHEWING TOBACCO . . . . . ☐☐☐<br>d) ANY OTHERS . . . . . ☐☐☐	a–d → 912
911	On average, how many times a week do you use the following products? Also, let me know if you use the product, but not every week.<br><br>IF RESPONDENT REPORTS USING THE PRODUCT BUT NOT EVERY WEEK, RECORD '888'. IF THE PRODUCT IS NOT USED AT ALL, RECORD '000'.<br><br>a) Snuff, by mouth?<br>b) Snuff, by nose?<br>c) Chewing tobacco?<br>d) Any others?<br>__________ (SPECIFY)	TIMES WEEKLY<br>a) SNUFF, BY MOUTH . . . . . ☐☐☐<br>b) SNUFF, BY NOSE . . . . . ☐☐☐<br>c) CHEWING TOBACCO . . . . . ☐☐☐<br>d) ANY OTHERS . . . . . ☐☐☐	
912	Do you currently work in a job where other people smoke around you?	YES . . . . . 1<br>NO . . . . . 2<br><br>NOT CURRENTLY WORKING 3	
913	Have you ever worked in a job where you were regularly exposed to smoke, dust, fumes or strong smells?	YES . . . . . 1<br>NO 2	2 → 915
914	How many years did you work at a job where you were regularly exposed to smoke, dust, fumes or strong smells?<br>IF LESS THAN 1 YEAR, RECORD '00'.	YEARS . . . . . ☐☐	
915	Do you currently use e-cigarettes every day, some days, or not at all?	EVERY DAY . . . . . 1<br>SOME DAYS . . . . . 2<br>NOT AT ALL 3	
916	Have you ever consumed a drink that contains alcohol such as beer, wine, ciders, spirits, or sorghum beer?<br>PROBE: Even one drink?	YES . . . . . 1<br>NO 2	2 → 1001

NO.	QUESTIONS AND FILTERS	CODING CATEGORIES	SKIP
917	Was this within the last 12 months?	YES . . . . 1<br>NO 2	 → 1001
918	In the last 12 months, how frequently have you had at least one drink?<br><br>PROBE: Five or more days a week, 1-4 days a week, 1-3 days a month, or less often than once a month?	5 OR MORE DAYS A WEEK . . . . 1<br>1-4 DAYS PER WEEK . . . . 2<br>1-3 DAYS A MONTH . . . . 3<br>LESS OFTEN THAN ONCE A MONTH 4	
919	During each of the last 7 days, how many standard drinks did you have?<br><br>USE SHOWCARD. RECORD TOTAL NUMBER OF DRINKS CONSUMED EACH DAY STARTING WITH THE DAY BEFORE THE DAY OF THE INTERVIEW AND PROCEEDING BACKWARDS.<br><br>IF NONE, RECORD '00'.	MONDAY . . . . [ ][ ]<br>TUESDAY . . . . [ ][ ]<br>WEDNESDAY . . . . [ ][ ]<br>THURSDAY . . . . [ ][ ]<br>FRIDAY . . . . [ ][ ]<br>SATURDAY . . . . [ ][ ]<br>SUNDAY . . . . [ ][ ]	
919H	During the last 7 days, how many standard home-made beers or other homemade alcohol did you have?<br><br>USE SHOWCARD.	NUMBER OF HOME-MADE BEERS . . [ ][ ]	
919I	CHECK 918 AND 919: CODE 3 OR 4 RECORDED IN 918 AND CONSUMED 0-1 DRINKS IN THE LAST 7 DAYS IN 919?<br>NO [ ] ↓ YES [ ] →		1001
920	Have you ever felt that you should cut down on your drinking?	YES . . . . 1<br>NO 2	
921	Have people annoyed you by criticizing your drinking?	YES . . . . 1<br>NO 2	
922	Have you ever felt bad or guilty about your drinking?	YES . . . . 1<br>NO 2	
923	Have you ever had a drink first thing in the morning to steady your nerves or get rid of a hangover?	YES . . . . 1<br>NO 2	
923A	CHECK 919: FIVE OR MORE DRINKS IN ONE DAY DURING LAST 7 DAYS?<br>NO [ ] ↓ YES [ ] →		1001
924	In the past 30 days, have you consumed five or more standard drinks on at least one occasion?	YES . . . . 1<br>NO 2	

SECTION 10. FAT, SALT, SUGAR, FRUIT AND VEGETABLE CONSUMPTION

NO.	QUESTIONS AND FILTERS	CODING CATEGORIES	SKIP
1001	Now I would like to ask you some questions about the foods that you eat. There are no right or wrong answers.<br><br>USE SHOWCARD.		
1004	How often do you usually eat fried foods such as hot chips, fried fish, fried chicken, fried meat, vetkoek or doughnuts?	EVERY DAY . . . . . 1<br>AT LEAST ONCE A WEEK . . . . . 2<br>OCCASIONALLY . . . . . 3<br>NEVER 4	
1005	How often do you eat fast-foods or take-away foods from places like Chicken Licken, KFC, Captain DoRego's, Steers, Nando's, McDonalds, pizza delivery, etc?	EVERY DAY . . . . . 1<br>AT LEAST ONCE A WEEK . . . . . 2<br>OCCASIONALLY . . . . . 3<br>NEVER 4	
1006	How often do you eat chips such as a packet of crispy chips or similar salty snacks such as Doritos, cheese curls, salted nuts, salty biscuits, etc?	EVERY DAY . . . . . 1<br>AT LEAST ONCE A WEEK . . . . . 2<br>OCCASIONALLY . . . . . 3<br>NEVER 4	
1007	How often do you eat processed meat such as polony, viennas, meat pies, or sausage rolls?	EVERY DAY . . . . . 1<br>AT LEAST ONCE A WEEK . . . . . 2<br>OCCASIONALLY . . . . . 3<br>NEVER 4	
1008	Which of the following statements best describes your approach towards salt consumption:<br><br>1) I am not interested in lowering salt in my food.<br><br>2) I am interested in lowering salt in my food within the next six months.<br><br>3) I am interested in lowering salt in my food within the next month.<br><br>4) I have started lowering salt within the last six months.<br><br>5) I have already lowered my salt intake for longer than six months.	NO INTENTION TO LOWER SALT . . . . . 1<br>INTERESTED WITHIN NEXT SIX MONTHS . . . . . 2<br>INTERESTED WITHIN NEXT MONTH . . . . . 3<br>STARTED IN LAST SIX MONTHS . . . . . 4<br>ALREADY LOWERED LONGER THAN SIX MONTH: 5<br><br>DON'T KNOW 8	
1009	Yesterday, how many types of fruit did you eat?<br><br>USE SHOWCARD. IF NONE, RECORD '00'.	TYPES OF FRUIT . . . . . [ ][ ]	
1010	Yesterday, how many types of vegetables, excluding potatoes, did you eat?<br>USE SHOWCARD. IF NONE, RECORD '00'.	TYPES OF VEGETABLES . . . . . [ ][ ]	
1011	Yesterday, did you drink any sugar-sweetened drinks? Sugar-sweetened drinks include fizzy drinks like Coke or drinks like Squash where water is added, but not diet or unsweetened cold drinks.	YES . . . . . 1<br>NO 2	 → 1012
1011A	How many and what size sugar-sweetened drinks did you drink?<br><br>PROBE FOR BEVERAGE NUMBER AND SIZE.	200 ML GLASS . . . . . A [ ][ ]<br>330 ML CAN OR BOTTLE . . . . . B [ ][ ]<br>500 ML BOTTLE . . . . . C [ ][ ]<br>1 L BOTTLE . . . . . D [ ][ ]<br>2 L BOTTLE . . . . . E [ ][ ]	
1012	Yesterday, did you drink any fruit juice?	YES . . . . . 1<br>NO 2	 → 1101
1012A	How many and what size fruit juices did you drink?<br><br>PROBE FOR BEVERAGE NUMBER AND SIZE.	200 ML JUICE CARTON . . . . . A [ ][ ]<br>200 ML GLASS . . . . . B [ ][ ]	

SECTION 11. HEALTH CARE

NO.	QUESTIONS AND FILTERS	CODING CATEGORIES	SKIP
1101	Are you covered by Medical Aid, Medical Benefit Scheme, Provident Scheme, or Hospital Plan that helps you pay for health care or drug services?	YES . . . . . 1<br>NO 2	
1102	During the last month, have you received health, medical, or dental care without staying overnight?	YES . . . . . 1<br>NO 2	NO → 1104
1103	Where have you received health, medical, or dental care?<br><br>PROBE: Anywhere else?<br><br>PROBE TO IDENTIFY THE TYPE OF SOURCE.<br><br>IF UNABLE TO DETERMINE IF PUBLIC OR PRIVATE SECTOR, RECORD 'X' AND WRITE THE NAME OF THE PLACE(S).	**PUBLIC SECTOR**<br>GOVERNMENT HOSPITAL . . . . . A<br>GOVERNMENT CLINIC/COMMUNITY HEALTH CENTRE . . . . . B<br>OTHER PUBLIC SECTOR<br>________ C<br>(SPECIFY)<br><br>**PRIVATE MEDICAL SECTOR**<br>PRIVATE HOSPITAL/CLINIC/ PRIVATE DOCTOR . . . . . D<br>CHEMIST/PHARMACY . . . . . E<br>DENTIST/ORAL HYGIENIST/ DENTAL THERAPIST . . . . . F<br>OTHER PRIVATE MEDICAL SECTOR<br>________ G<br>(SPECIFY)<br>**OTHER SOURCE**<br>WORKPLACE HEALTH SERVICE . . . . . H<br>TRADITIONAL HEALER . . . . . I<br>TRADITIONAL HERBALIST . . . . . J<br>FAITH HEALER . . . . . K<br><br>OTHER ________ X<br>(SPECIFY)	
1104	During the last month, have you had any visits by a home-based care giver or a community-based care giver?	YES . . . . . 1<br>NO . . . . . 2<br>DON'T KNOW 8	
1105	Has a doctor, nurse or other health worker ever told you that you have TB?	YES . . . . . 1<br>NO . . . . . 2<br>DON'T KNOW 8	NO, DON'T KNOW → 1108
1106	When was the last time you were told you had TB?	IN THE LAST 12 MONTHS . . . . . 1<br>MORE THAN 12 MONTHS AGO 2	
1107	Did you get medical treatment the last time you had TB?	YES . . . . . 1<br>NO . . . . . 2<br>DON'T KNOW/DON'T REMEMBER 8	
1108	Has a doctor, nurse or other health worker told you that you have or have had any of the following conditions:<br><br>a) High blood pressure?<br>b) Heart attack or angina/chest pains?<br>c) Cancer?<br>d) Stroke?<br>e) High blood cholesterol or fats in the blood?<br>f) Diabetes or blood sugar?<br>g) Chronic bronchitis, emphysema, or COPD?<br>h) Asthma?	YES NO DK<br><br>a) HIGH BLOOD PRESS. 1 2 8<br>b) HEART ATTACK 1 2 8<br>c) CANCER 1 2 8<br>d) STROKE 1 2 8<br>e) HIGH BLOOD CHOLEST 1 2 8<br>f) DIABETES 1 2 8<br>g) CHRONIC BRONCHITIS 1 2 8<br>h) ASTHMA 1 2 8	
1109	CHECK 1108:<br>ANY QUESTION a-h = YES?	YES ☐ ↓ NO ☐	NO → 1127

NO.	QUESTIONS AND FILTERS	CODING CATEGORIES	SKIP
1110	CHECK 1108a:<br>RESPONDENT HAS HAD HIGH BLOOD PRESSURE.	1108a = YES ☐ ↓ 1108a = NO OR DK ☐	→ 1112
1111	Did you receive medical treatment for high blood pressure at the time of the diagnosis?	YES . . . . . 1<br>NO . . . . . 2<br>DON'T KNOW/DON'T REMEMBER 8	
1112	CHECK 1108b:<br>RESPONDENT HAS HAD HEART ATTACK OR ANGINA.	1108b = YES ☐ ↓ 1108b = NO OR DK ☐	→ 1114
1113	Did you receive medical treatment for the heart attack, angina/chest pains at the time of the diagnosis?	YES . . . . . 1<br>NO . . . . . 2<br>DON'T KNOW/DON'T REMEMBER 8	
1114	CHECK 1108c:<br>RESPONDENT HAS HAD CANCER.	1108c = YES ☐ ↓ 1108c = NO OR DK ☐	→ 1116
1115	Did you receive medical treatment for the cancer at the time of the diagnosis?	YES . . . . . 1<br>NO . . . . . 2<br>DON'T KNOW/DON'T REMEMBER 8	
1116	CHECK 1108d:<br>RESPONDENT HAS HAD STROKE.	1108d = YES ☐ ↓ 1108d = NO OR DK ☐	→ 1118
1117	Did you receive medical treatment for the stroke at the time of the diagnosis?	YES . . . . . 1<br>NO . . . . . 2<br>DON'T KNOW/DON'T REMEMBER 8	
1118	CHECK 1108e:<br>RESPONDENT HAS HAD HIGH BLOOD CHOLESTEROL.	1108e = YES ☐ ↓ 1108e = NO OR DK ☐	→ 1120
1119	Did you receive medical treatment for high blood cholesterol or fats in the blood at the time of the diagnosis?	YES . . . . . 1<br>NO . . . . . 2<br>DON'T KNOW/DON'T REMEMBER 8	
1120	CHECK 1108f:<br>RESPONDENT HAS HAD DIABETES.	1108f = YES ☐ ↓ 1108f = NO OR DK ☐	→ 1122
1121	Did you receive medical treatment for the diabetes or blood sugar at the time of the diagnosis?	YES . . . . . 1<br>NO . . . . . 2<br>DON'T KNOW/DON'T REMEMBER 8	
1122	CHECK 1108g:<br>RESPONDENT HAS HAD CHRONIC BRONCHITIS.	1108g = YES ☐ ↓ 1108g = NO OR DK ☐	→ 1124
1123	Did you receive medical treatment for chronic bronchitis, emphysema, or COPD at the time of the diagnosis?	YES . . . . . 1<br>NO . . . . . 2<br>DON'T KNOW/DON'T REMEMBER 8	
1124	CHECK 1108h:<br>RESPONDENT HAS HAD ASTHMA.	1108h = YES ☐ ↓ 1108h = NO OR DK ☐	→ 1127
1125	Did you receive medical treatment for asthma at the time of the diagnosis?	YES . . . . . 1<br>NO . . . . . 2<br>DON'T KNOW/DON'T REMEMBER 8	
1127	Compared with other people your age, do you feel you have less breath when exerting yourself?<br><br>PROBE: By exercising or moving a lot?	YES . . . . . 1<br>NO . . . . . 2<br>DON'T KNOW 8	
1128	During the last 12 months, have you had wheezing when you breathe?	YES . . . . . 1<br>NO . . . . . 2<br>DON'T KNOW 8	2, 8 → 1131

SECTION 11. HEALTH CARE

NO.	QUESTIONS AND FILTERS	CODING CATEGORIES	SKIP
1129	Were you also short of breath when the wheezing noise was present?	YES . . . 1<br>NO . . . 2<br>DON'T KNOW . . . 8	
1130	Have you had the wheezing when you did not have a cold?	YES . . . 1<br>NO . . . 2<br>DON'T KNOW . . . 8	
1131	Have you woken up with a feeling of tightness in your chest at any time in the last 12 months?	YES . . . 1<br>NO . . . 2<br>DON'T KNOW . . . 8	
1132	Have you been woken by an attack of shortness of breath at any time in the last 12 months?	YES . . . 1<br>NO . . . 2<br>DON'T KNOW . . . 8	
1133	Have you been woken by an attack of coughing at any time in the last 12 months?	YES . . . 1<br>NO . . . 2<br>DON'T KNOW . . . 8	
1134	Do you usually cough on most days?	YES . . . 1<br>NO . . . 2<br>DON'T KNOW . . . 8	2, 8 → 1138
1135	When you cough, do you usually bring up phlegm from your chest?	YES . . . 1<br>NO . . . 2<br>DON'T KNOW . . . 8	2, 8 → 1138
1136	Have you brought up phlegm every day for at least three months during the last year?	YES . . . 1<br>NO . . . 2<br>DON'T KNOW . . . 8	2, 8 → 1138
1137	For how many years have you brought up phlegm in this way?<br>IF LESS THAN 1 YEAR, RECORD '00'.	YEARS . . . [ ][ ]	
1138	Are you currently troubled by pain or discomfort, either all the time or on and off?	YES . . . 1<br>NO . . . 2	2 → 1141
1139	Have you had this pain or discomfort for more than 3 months?	YES . . . 1<br>NO . . . 2	2 → 1141
1140	Where do you feel this pain or discomfort?<br><br>RECORD ALL MENTIONED.	BACK PAIN . . . A<br>NECK OR SHOULDER PAIN . . . B<br>HEADACHE, FACIAL OR DENTAL PAIN . . . C<br>STOMACH ACHE OR ABDOMINAL PAIN . . . D<br>PAIN IN ARMS, HANDS, HIPS, LEGS OR FEET . . E<br>CHEST PAIN . . . F<br><br>OTHER ______ X<br>(SPECIFY)	
1141	In the last 12 months, did your teeth or your mouth cause you any pain or discomfort?	YES . . . 1<br>NO . . . 2	2 → 1145
1142	Did you get treatment the last time that you had the problem?	YES . . . 1<br>NO . . . 2	2 → 1144
1143	Who did you see for treatment?<br><br>RECORD ALL MENTIONED.	**PUBLIC SECTOR**<br>DENTIST/ORAL HYGIENIST/DENTAL THERAPIST . . . A<br>MEDICAL DOCTOR/NURSE . . . B<br><br>**PRIVATE MEDICAL SECTOR**<br>DENTIST/ORAL HYGIENIST/DENTAL THERAPIST . . . C<br>MEDICAL DOCTOR/NURSE . . . D<br><br>**OTHER SOURCE**<br>TRADITIONAL HEALER . . . E<br><br>OTHER . . . X	A–X → 1145

NO.	QUESTIONS AND FILTERS	CODING CATEGORIES	SKIP
1144	What was the main reason that you did not get treatment?	NO ORAL HEALTH SERVICE AVAILABLE . . . . . 1<br>ORAL HEALTH SERVICES TOO FAR . . . . 2<br>ORAL HEALTH SERVICES TOO EXPENSIVE/ COULD NOT AFFORD . . . . 3<br>PROBLEM WENT AWAY . . . . 4<br>OTHER 6	
1145	Now I would like to ask you about any medication you take. Do you use any medicine daily or regularly that has been prescribed by a doctor or nurse?	YES . . . . 1<br>NO 2	 2 → 1150
1146	How many different prescribed medications do you use daily or regularly?	NUMBER OF MEDICINES . . . . [ ][ ]	
1147	Who pays for most of these medications?	RESPONDENT . . . . 1<br>FAMILY/FRIENDS . . . . 2<br>MEDICAL AID . . . . 3<br>EMPLOYER . . . . 4<br>PROVIDED BY PUBLIC CLINIC OR HOSPITAL . . 5<br>OTHER 6	1–4 → 1150<br>6 → 1150
1148	In the last 12 months, have you ever been sent away from the clinic without a medication because they did not have stock?	YES . . . . 1<br>NO 2	 2 → 1150
1149	How many times has this happened to you in the last 12 months?<br>PROBE FOR ESTIMATE OF NUMBER OF TIMES.	NUMBER OF TIMES . . . . [ ][ ]	
1150	In the last 12 months, have you used any medications containing codeine to treat a medical condition?<br>USE THE SHOWCARD.	YES . . . . 1<br>NO . . . . 2<br>DON'T KNOW 8	2, 8 → 1155
1152	In the last 12 months, have you used any of these medications for the experience or feeling it gave you rather than for their medicinal effect?	YES . . . . 1<br>NO 2	 2 → 1155
1153	In the last 12 months, which codeine-containing medications have you used for the experience or feeling rather than for their medical effect?<br>RECORD ALL MENTIONED.	BRONCLEER/LENAZINE FORTE . . . . A<br>ACTIFED DRY COUGH . . . . B<br>BENYLIN SYRUP WITH CODEINE . . . . C<br>LENADOL/ADCO-DOL PAIN TABLETS . . . . D<br>NUROFEN PLUS . . . . E<br>MYPRODOL . . . . F<br>STILPANE . . . . G<br>SYNDOL . . . . H<br>OTHER ______________________ X<br>(SPECIFY)	
1154	In the last 12 months, have you received treatment for your problems related to the use of codeine-containing medications for non-medical purposes?	YES . . . . 1<br>NO 2	
1155	RECORD THE TIME.	HOURS . . . . [ ][ ]<br>MINUTES . . . . [ ][ ]	

INTERVIEWER'S OBSERVATIONS

TO BE FILLED IN AFTER COMPLETING INTERVIEW

COMMENTS ABOUT INTERVIEW:

COMMENTS ON SPECIFIC QUESTIONS:

ANY OTHER COMMENTS:

SUPERVISOR'S OBSERVATIONS

2016 SOUTH AFRICA DEMOGRAPHIC AND HEALTH SURVEY
CAREGIVER'S QUESTIONNAIRE FOR CHILD WHOSE BIOLOGICAL MOTHER DOES NOT LIVE IN THE HOUSEHOLD

IDENTIFICATION

PLACE NAME ______

NAME OF HOUSEHOLD HEAD ______

CLUSTER NUMBER . . . . [ ][ ][ ][ ]

HOUSEHOLD NUMBER . . . . [ ][ ][ ][ ]

NAME AND LINE NUMBER OF CHILD ______ [ ][ ]

NAME AND LINE NUMBER OF GUARDIAN/FOSTER PARENT/OTHER ______ [ ][ ]

INTERVIEWER VISITS

	1	2	3	FINAL VISIT
DATE	______	______	______	DAY [ ][ ]<br>MONTH [ ][ ]<br>YEAR [2][0][1][ ]
INTERVIEWER'S NAME	______	______	______	INT. NO. [ ][ ][ ][ ]
RESULT*	______	______	______	RESULT* [ ]
NEXT VISIT: DATE	______	______		TOTAL NUMBER OF VISITS [ ]
TIME	______	______		

*RESULT CODES:
1 COMPLETED
2 NOT AT HOME
3 POSTPONED
4 REFUSED
5 PARTLY COMPLETED
6 INCAPACITATED
7 OTHER ______ (SPECIFY)

LANGUAGE OF QUESTIONNAIRE** [0][1]
LANGUAGE OF INTERVIEW** [ ][ ]
HOME LANGUAGE OF RESPONDENT** [ ][ ]
TRANSLATOR USED (YES = 1, NO = 2) [ ]

LANGUAGE OF QUESTIONNAIRE** **ENGLISH**

**LANGUAGE CODES:

01 ENGLISH	05 seSOTHO	09 tshiVENDA
02 AFRIKAANS	06 seTSWANA	10 xiTSONGA
03 isiXHOSA	07 sePEDI	11 isiNDEBELE
04 isiZULU	08 siSWATI	12 OTHER

SUPERVISOR

______ NAME

[ ][ ][ ][ ] NUMBER

INTRODUCTION AND CONSENT

Hello. My name is ______________________. I am working with Statistics South Africa. We are conducting a survey about health and other topics all over South Africa. The information we collect will help the government to plan health services. Your household was selected for the survey. I would like to talk to you about (CHILD'S NAME FROM COVERSHEET)'s health and well-being. The questions usually take about 10-15 minutes. All of the answers you give will be confidential and will not be shared with anyone other than members of our survey team. You don't have to be in the survey, but we hope you will agree to answer the questions since your views are important. If I ask you any question you don't want to answer, just let me know and I will go on to the next question or you can stop the interview at any time.

In case you need more information about the survey, you may contact the person listed on the card that has already been given to your household.

Do you have any questions?
May I begin the interview now?

SIGNATURE OF INTERVIEWER ______________________ DATE ______________

RESPONDENT AGREES TO BE INTERVIEWED . . 1 ↓

RESPONDENT DOES NOT AGREE TO BE INTERVIEWED . . 2 → END

SECTION 1.CHILD'S BACKGROUND

NO.	QUESTIONS AND FILTERS	CODING CATEGORIES	SKIP
101	RECORD THE TIME.	HOURS . . . . [ ][ ]<br>MINUTES . . . . [ ][ ]	
102	RECORD NAME AND LINE NUMBER OF CHILD.	NAME ______________<br>LINE NUMBER . . . . [ ][ ]	
103	Is (NAME) a boy or a girl?	BOY . . . . 1<br>GIRL 2	
104	On what day, month, and year was (NAME FROM 102) born?<br><br>PROBE: What is (NAME)'s birthday?	DAY . . . . [ ][ ]<br>DON'T KNOW DAY . . . . 98<br>MONTH . . . . [ ][ ]<br>DON'T KNOW MONTH . . . . 98<br>YEAR . . . . [ ][ ][ ][ ]	
105	How old was (NAME) at (NAME)'s last birthday?<br><br>COMPARE AND CORRECT 104 AND/OR 105 IF INCONSISTENT.	AGE IN COMPLETED YEARS . . . . [ ][ ]	
106	Which population group does (NAME) belong to: black, white, coloured, Indian or something else?	BLACK/AFRICAN . . . . 1<br>WHITE . . . . 2<br>COLOURED . . . . 3<br>INDIAN/ASIAN . . . . 4<br>OTHER ______________ 6<br>(SPECIFY)	

SECTION 1.CHILD'S BACKGROUND

NO.	QUESTIONS AND FILTERS	CODING CATEGORIES	SKIP
107	What is your relationship to (NAME)?	BIOLOGICAL FATHER . . . . . 11<br>ADOPTIVE PARENT . . . . . 12<br>FOSTER PARENT . . . . . 13<br>STEP PARENT . . . . . 14<br>GRANDPARENT . . . . . 15<br>BROTHER/SISTER . . . . . 16<br>OTHER RELATIVE . . . . . 17<br>UNRELATED GUARDIAN/CAREGIVER . . . . . 18<br>OTHER ______ 96<br>(SPECIFY)	
108	Why is (NAME)'s biological mother not living with (NAME)?	MOTHER DIED . . . . . 1<br>MOTHER WORKS ELSEWHERE/<br>SEEKS EMPLOYMENT ELSEWHERE . . . . . 2<br>MOTHER SENT CHILD HERE FOR CARE . . . . . 3<br>MOTHER IN HOSPITAL . . . . . 4<br>OTHER ______ 6<br>(SPECIFY)	

SECTION 2. CHILD IMMUNISATION

NO.	QUESTIONS AND FILTERS	CODING CATEGORIES		SKIP
501A	CHECK 104: BORN IN 2013-2016? YES ☐ (continue)	NO ☐		→ 602
504A	Do you have a Road to Health booklet/card or other document where (NAME)'s vaccinations are written down?	YES, HAS ONLY A BOOKLET	1	→ 507A
		YES, HAS ONLY ANOTHER DOCUMENT	2	
		YES, HAS BOOKLET AND OTHER DOCUMENT	3	→ 507A
		NO, NO BOOKLET AND NO OTHER DOCUMENT	4	
505A	Did you ever have a Road to Health booklet for (NAME)?	YES	1	
		NO	2	→ 505A2
505A1	What happened to (NAME)'s Road to Health booklet?	BOOKLET WITH RELATIVE/CHILD'S MOTHER	1	→ 506A
		BOOKLET MISPLACED OR LOST	2	→ 506A
		BOOKLET STOLEN	3	→ 506A
		BOOKLET HELD AS COLLATERAL/RANSOM . .	4	→ 506A
		BOOKLET DESTROYED	5	→ 506A
		OTHER ________ (SPECIFY)	6	→ 506A
505A2	Why don't you have a Road to Health booklet for (NAME)?	NONE AVAILABLE AT HEALTH FACILITY	1	
		FOREIGNERS NOT GIVEN ONE	2	
		REQUIRED TO PAY FOR IT	3	
		TOO BUSY TO GET ONE	4	
		BOOKLET WITH RELATIVE/CHILD'S MOTHER . .	5	
		OTHER ________ (SPECIFY)	6	
506A	CHECK 504A: CODE '2' CIRCLED ☐ (continue)	CODE '4' CIRCLED ☐		→ 511A
506A1	May I see the document where (NAME)'s vaccinations are written down?	YES, OTHER DOCUMENT SEEN	1	→ 508A
		NO DOCUMENT SEEN	2	→ 511A
507A	May I see the Road to Health booklet or other document where (NAME)'s vaccinations are written down?	YES, ONLY BOOKLET SEEN	1	→ 508A
		YES, ONLY OTHER DOCUMENT SEEN	2	
		YES, BOOKLET AND OTHER DOCUMENT SEEN	3	→ 508A
		NO BOOKLET AND NO OTHER DOCUMENT SEEN	4	
507A1	Where is (NAME)'s Road to Health booklet?	BOOKLET WITH RELATIVE/CHILD'S MOTHER	1	
		BOOKLET MISPLACED OR LOST	2	
		BOOKLET STOLEN	3	
		BOOKLET HELD AS COLLATERAL/RANSOM . .	4	
		BOOKLET AT HEALTH FACILITY	5	
		OTHER ________ (SPECIFY)	6	
507A2	CHECK 507A: CODE '2' CIRCLED ☐ (continue)	CODE '4' CIRCLED ☐		→ 511A

508A

PHOTOGRAPH VACCINATION PAGE OF BOOKLET OR OTHER DOCUMENT WHERE VACCINATIONS ARE WRITTEN.

COPY DATES FROM THE BOOKLET.
WRITE '44' IN 'DAY' COLUMN IF BOOKLET SHOWS THAT A DOSE WAS GIVEN, BUT NO DATE IS RECORDED.

	DAY		MONTH		YEAR			
BCG								
ORAL POLIO VACCINE (OPV) 0 (BIRTH DOSE)								
ORAL POLIO VACCINE (OPV) 1								
ROTAVIRUS (RV) 1								
DTAP-IPV-HIB 1								
HEPATITIS B (HEP B) 1								
PNEUMOCOCCAL CONJUGATE VACCINE (PCV) 1								
DTAP-IPV-HIB 2								
HEPATITIS B (HEP B) 2								
DTAP-IPV-HIB 3								
HEPATITIS B (HEP B) 3								
PNEUMOCOCCAL CONJUGATE VACCINE (PCV) 2								
ROTAVIRUS (RV) 2								
MEASLES 1								
PNEUMOCOCCAL CONJUGATE VACCINE (PCV) 3								
DTAP-IPV-HIB 4								
MEASLES 2								
VITAMIN A (MOST RECENT)								

509A

CHECK 508A: 'BCG' TO 'MEASLES 2' ALL RECORDED?

NO ☐ YES ☐ → 525A

510A

In addition to what is recorded on (this document/these documents), did (NAME) receive any other vaccinations, including vaccinations received in immunisation campaigns?

RECORD 'YES' ONLY IF THE RESPONDENT MENTIONS AT LEAST ONE OF THE VACCINATIONS IN 508A THAT ARE NOT RECORDED AS HAVING BEEN GIVEN.

YES 1
(PROBE FOR VACCINATIONS AND WRITE '66' IN THE CORRESPONDING DAY COLUMN IN 508A THEN WRITE '00' IN THE CORRESPONDING DAY COLUMN FOR ALL VACCINATIONS NOT GIVEN)
(THEN SKIP TO 525A)

NO 2
DON'T KNOW 8
(WRITE '00' IN THE CORRESPONDING DAY COLUMN FOR ALL VACCINATIONS NOT GIVEN)
(THEN SKIP TO 525A)

SECTION 2. CHILD IMMUNISATION

NO.	QUESTIONS AND FILTERS	CODING CATEGORIES	SKIP
511A	Did (NAME) ever receive any vaccinations to prevent (NAME) from getting diseases, including vaccinations received in immunisation campaigns?	YES . . . . 1<br>NO . . . . 2<br>DON'T KNOW 8	 2 → 526A<br>8 → 602
512A	Has (NAME) ever received a BCG vaccination against tuberculosis, that is, an injection in the arm or shoulder that usually causes a scar?	YES . . . . 1<br>NO . . . . 2<br>DON'T KNOW 8	
514A	Has (NAME) ever received oral polio vaccine, that is, about two drops in the mouth to prevent polio?	YES . . . . 1<br>NO . . . . 2<br>DON'T KNOW 8	2, 8 → 517A
515A	Did (NAME) receive the first oral polio vaccine in the first two weeks after birth or later?	FIRST TWO WEEKS . . . . 1<br>LATER 2	
516A	How many times did (NAME) receive the oral polio vaccine?	NUMBER OF TIMES . . . . ☐	
517A	Has (NAME) ever received a DTP-combination vaccination, also known as a pentavalent vaccination? That is, an injection given in the left thigh or left arm to prevent diphtheria, tetanus, and whooping cough?	YES . . . . 1<br>NO . . . . 2<br>DON'T KNOW 8	2, 8 → 518A1
518A	How many times did (NAME) receive the DTP-combination vaccine?	NUMBER OF TIMES . . . . ☐	
518A1	Has (NAME) ever received a hepatitis B vaccination, that is, an injection given in the right thigh to prevent hepatitis B?	YES . . . . 1<br>NO . . . . 2<br>DON'T KNOW 8	2, 8 → 519A
518A2	How many times did (NAME) receive the hepatitis B vaccine?	NUMBER OF TIMES . . . . ☐	
519A	Has (NAME) ever received a pneumococcal vaccination, that is, an injection in the right thigh to prevent pneumonia?	YES . . . . 1<br>NO . . . . 2<br>DON'T KNOW 8	2, 8 → 521A
520A	How many times did (NAME) receive the pneumococcal vaccine?	NUMBER OF TIMES . . . . ☐	
521A	Has (NAME) ever received a rotavirus vaccination, that is, syrup in the mouth to prevent diarrhoea?	YES . . . . 1<br>NO . . . . 2<br>DON'T KNOW 8	2, 8 → 523A
522A	How many times did (NAME) receive the rotavirus vaccine?	NUMBER OF TIMES . . . . ☐	
523A	Has (NAME) ever received a measles vaccination, that is, an injection in the left thigh or right arm to prevent measles?	YES . . . . 1<br>NO . . . . 2<br>DON'T KNOW 8	2, 8 → 525A
524A	How many times did (NAME) receive the measles vaccine?	NUMBER OF TIMES . . . . ☐	
525A	Did (NAME) ever miss getting a vaccination or get a vaccination late?	YES . . . . 1<br>NO . . . . 2<br>DON'T KNOW 8	2, 8 → 602
526A	CHECK 508A AND 511A:<br>CHILD RECEIVED AT LEAST ONE VACCINATION ☐ ↓<br>a) What was the reason for (NAME) missing the vaccination or getting it late?<br>PROBE: Any other reason?<br>CHILD RECEIVED RECEIVED NO VACCINATIONS ☐ ↓<br>b) What is the reason (NAME) has not received any vaccinations?<br>PROBE: Any other reason?	CLINIC OUT OF STOCK . . . . A<br>NOT AWARE OF NEED FOR A VACCINATION B<br>FEAR OF SIDE EFFECTS . . . . C<br>DID NOT KNOW WHERE TO GO . . . . D<br>TOO BUSY TO TAKE CHILD . . . . E<br>NO MONEY FOR TRANSPORT . . . . F<br>CHILD WAS ILL . . . . G<br>RESPONDENT WAS ILL . . . . H<br>OTHER ______ X<br>(SPECIFY)<br>DON'T KNOW . . . . Z	

NO.	QUESTIONS AND FILTERS	CODING CATEGORIES	SKIP
602	Now I would like to ask some questions about (NAME)'s health.		
605	In the last six months, was (NAME) given a vitamin A dose like [this/any of these]?<br><br>SHOW COMMON TYPES OF AMPULES/CAPSULES/SYRUPS.	YES . . . . . 1<br>NO . . . . . 2<br>DON'T KNOW 8	
607	Was (NAME) given any drug for intestinal worms in the last six months?<br><br>IF RESPONDENT SAYS NO, CHECK ROAD TO HEALTH BOOKLET.	YES . . . . . 1<br>NO . . . . . 2<br>DON'T KNOW 8	
608	Has (NAME) had diarrhoea/loose stools in the last 2 weeks?	YES . . . . . 1<br>NO . . . . . 2<br>DON'T KNOW 8	 NO, DON'T KNOW → 618
609	Now I would like to know how much (NAME) was given to drink during the diarrhoea. Was (NAME) given less than usual to drink, about the same amount, or more than usual to drink?<br><br>IF LESS, PROBE: Was (NAME) given much less than usual to drink or somewhat less?	MUCH LESS . . . . . 1<br>SOMEWHAT LESS . . . . . 2<br>ABOUT THE SAME . . . . . 3<br>MORE . . . . . 4<br>NOTHING TO DRINK . . . . . 5<br>DON'T KNOW 8	
610	When (NAME) had diarrhoea, was (NAME) given less than usual to eat, about the same amount, more than usual, or nothing to eat?<br><br>IF LESS, PROBE: Was (NAME) given much less than usual to eat or somewhat less?	MUCH LESS . . . . . 1<br>SOMEWHAT LESS . . . . . 2<br>ABOUT THE SAME . . . . . 3<br>MORE . . . . . 4<br>STOPPED FOOD . . . . . 5<br>NEVER GAVE FOOD . . . . . 6<br>DON'T KNOW 8	
611	Did you seek advice or treatment for the diarrhoea from any source?	YES . . . . . 1<br>NO 2	 NO → 615

NO.	QUESTIONS AND FILTERS	CODING CATEGORIES	SKIP
612	Where did you seek advice or treatment?<br><br>Anywhere else?<br><br>PROBE TO IDENTIFY THE TYPE OF SOURCE.<br><br>IF UNABLE TO DETERMINE IF PUBLIC OR PRIVATE SECTOR, RECORD 'X' AND WRITE THE NAME OF THE PLACE(S).	**PUBLIC SECTOR**<br>GOVERNMENT HOSPITAL . . . . . . A<br>GOVERNMENT CLINIC/COMM. HEALTH CENTRE B<br>MOBILE CLINIC . . . . . . C<br>COMMUNITY HEALTH WORKER . . . . . . D<br>OTHER PUBLIC SECTOR<br>__________ E<br>(SPECIFY)<br><br>**PRIVATE MEDICAL SECTOR**<br>PRIVATE HOSPITAL/CLINIC . . . . . . F<br>CHEMIST/PHARMACY . . . . . . G<br>PRIVATE DOCTOR . . . . . . H<br>OTHER PRIVATE MEDICAL SECTOR<br>__________ I<br>(SPECIFY)<br>**OTHER SOURCE**<br>SUPERMARKET/SHOP . . . . . . J<br>TRADITIONAL HEALER . . . . . . K<br>MARKET . . . . . . L<br><br>OTHER __________ X<br>(SPECIFY)	
613	CHECK 612: TWO OR MORE CODES CIRCLED ☐ ↓	ONLY ONE CODE CIRCLED ☐ →	615
614	Where did you first seek advice or treatment?<br><br>USE LETTER CODE FROM 612.	FIRST PLACE . . . . . . ☐	
615	Was (NAME) given any of the following at any time since (NAME) started having the diarrhoea:<br><br>a) A fluid made from a special packet called Sorol or Rehidrat?<br>b) A clinic-recommended sugar-salt solution?<br>c) Zinc tablets or syrup?	YES NO DK<br><br>a) FLUID FROM ORS PACKET . . 1 2 8<br>b) CLINIC RECOMMENDED FLUID 1 2 8<br>c) ZINC 1 2 8	
616	CHECK 615<br>ANY 'YES' ☐ ↓ | ALL 'NO' OR 'DK' ☐ ↓<br>a) Was anything else given to treat the diarrhoea? | b) Was anything given to treat the diarrhoea?	YES . . . . . . 1<br>NO . . . . . . 2<br>DON'T KNOW 8	2, 8 → 618
617	CHECK 615<br>ANY 'YES' ☐ ↓ | ALL 'NO' OR 'DK' ☐ ↓<br>a) What else was given to treat the diarrhoea?<br>Anything else? | b) What was given to treat the diarrhoea?<br>Anything else?<br><br>RECORD ALL TREATMENTS GIVEN.	**PILL OR SYRUP**<br>ANTIBIOTIC . . . . . . A<br>ANTIMOTILITY . . . . . . B<br>OTHER (NOT ANTIBIOTIC OR ANTIMOTILITY) C<br>UNKNOWN PILL OR SYRUP . . . . . . D<br><br>**INJECTION**<br>ANTIBIOTIC . . . . . . E<br>NON-ANTIBIOTIC . . . . . . F<br>UNKNOWN INJECTION . . . . . . G<br><br>(IV) INTRAVENOUS . . . . . . H<br><br>HOME REMEDY/HERBAL MEDICINE . . . . . . I<br><br>OTHER __________ X<br>(SPECIFY)	
618	Has (NAME) been ill with a fever at any time in the last 2 weeks?	YES . . . . . . 1<br>NO . . . . . . 2<br>DON'T KNOW 8	

NO.	QUESTIONS AND FILTERS	CODING CATEGORIES	SKIP
620	Has (NAME) had an illness with a cough at any time in the last 2 weeks?	YES . . . . 1<br>NO . . . . 2<br>DON'T KNOW 8	
621	Has (NAME) had fast, short, rapid breaths or difficulty breathing at any time in the last 2 weeks?	YES . . . . 1<br>NO . . . . 2<br>DON'T KNOW 8	(NO, DON'T KNOW) → 623
622	Was the fast or difficult breathing due to a problem in the chest or to a blocked or runny nose?	CHEST ONLY . . . . 1<br>NOSE ONLY . . . . 2<br>BOTH . . . . 3<br>OTHER ________ 6<br>SPECIFY<br>DON'T KNOW 8	(1, 2, 3, 6, 8) → 624
623	CHECK 618: HAD FEVER<br>YES ☐ ↓ NO OR DON'T KNOW ☐ → 631		631
624	Did you seek advice or treatment for the illness from any source?	YES . . . . 1<br>NO 2	(NO) → 631
625	Where did you seek advice or treatment?<br><br>Anywhere else?<br><br>PROBE TO IDENTIFY THE TYPE OF SOURCE.<br><br>IF UNABLE TO DETERMINE IF PUBLIC OR PRIVATE SECTOR, RECORD 'X' AND WRITE THE NAME OF THE PLACE(S).	**PUBLIC SECTOR**<br>GOVERNMENT HOSPITAL . . . . A<br>GOVERNMENT CLINIC/COMM. HEALTH CENTRE B<br>MOBILE CLINIC . . . . C<br>COMMUNITY HEALTH WORKER . . . . D<br>OTHER PUBLIC SECTOR<br>________ E<br>(SPECIFY)<br>**PRIVATE MEDICAL SECTOR**<br>PRIVATE HOSPITAL/CLINIC . . . . F<br>CHEMIST/PHARMACY . . . . G<br>PRIVATE DOCTOR . . . . H<br>OTHER PRIVATE MEDICAL SECTOR<br>________ I<br>(SPECIFY)<br>**OTHER SOURCE**<br>SUPERMARKET/SHOP . . . . J<br>TRADITIONAL HEALER . . . . K<br>MARKET . . . . L<br><br>OTHER ________ X<br>(SPECIFY)	
626	CHECK 625: TWO OR MORE CODES CIRCLED ☐ ↓ ONLY ONE CODE CIRCLED ☐ → 628		628
627	Where did you first seek advice or treatment?<br><br>USE LETTER CODE FROM 625.	FIRST PLACE . . . . [ ]	
628	How many days after the illness began did you first seek advice or treatment for (NAME)?<br><br>IF THE SAME DAY RECORD '00'.	DAYS . . . . [ ][ ]	

NO.	QUESTIONS AND FILTERS	CODING CATEGORIES	SKIP
629	At any time during the illness, did (NAME) take any drugs for the illness?	YES . . . 1<br>NO . . . 2<br>DON'T KNOW . . . 8	2, 8 → 631
630	What drugs did (NAME) take?<br><br>Any other drugs?<br><br>RECORD ALL MENTIONED.	**ANTIMALARIAL DRUGS**<br>COARTEM/ARTEMISININ COMBINATION THERAPY (ACT) . . . A<br>OTHER ANTIMALARIAL<br>__________ B<br>(SPECIFY)<br><br>**ANTIBIOTIC DRUGS**<br>PILL/SYRUP . . . C<br>INJECTION/IV . . . D<br><br>**OTHER DRUGS**<br>ASPIRIN . . . E<br>PARACETAMOL/PANADO . . . F<br>BRUFEN . . . G<br>PONSTAN . . . H<br><br>OTHER __________ X<br>(SPECIFY)<br><br>DON'T KNOW . . . Z	
631	CHECK 104: CHILD BORN IN 2014-2016?<br>YES ☐ ↓	NO ☐ →	655
654	The last time (NAME) passed stools, what was done to dispose of the stools?	CHILD USED TOILET OR LATRINE . . . 01<br>PUT/RINSED INTO TOILET OR LATRINE . . . 02<br>PUT/RINSED INTO DRAIN, DITCH RIVER OR STREAM . . . 03<br>THROWN INTO GARBAGE . . . 04<br>BURIED . . . 05<br>LEFT IN THE OPEN . . . 06<br><br>OTHER __________ 96<br>(SPECIFY)	
655	I do not want to know if (NAME) has HIV, I just want to know if you know (NAME)'s HIV status. Do you know (NAME)'s HIV status?	YES . . . 1<br>NO . . . 2<br>DON'T KNOW . . . 8	
656	RECORD THE TIME.	HOURS . . . ☐☐<br>MINUTES . . . ☐☐	

INTERVIEWER'S OBSERVATIONS

TO BE FILLED IN AFTER COMPLETING BIOMARKERS

SUPERVISOR'S OBSERVATIONS

13 June 2016

2016 SOUTH AFRICA DEMOGRAPHIC AND HEALTH SURVEY
FIELDWORKER QUESTIONNAIRE

STATS SA
SA MRC

LANGUAGE OF QUESTIONNAIRE ENGLISH

NO.	QUESTIONS AND FILTERS	CODING CATEGORIES	SKIP
100	What is your name?	NAME ____________	
101	RECORD FIELDWORKER NUMBER	NUMBER . . . . [ ][ ][ ][ ]	

INSTRUCTIONS

We are collecting information on the SADHS field staff. Please fill in the information below. The information will be part of the survey data files. Your name will not be in the data files; your information will remain anonymous. If there is any question you do not want to answer you may skip it and go to the next question.

AGREE . . . . 1 ↓ DO NOT AGREE . . . . 2 → END

NO.	QUESTIONS AND FILTERS	CODING CATEGORIES	SKIP
102	In what province do you live?	WESTERN CAPE . . . . . 01<br>EASTERN CAPE . . . . . 02<br>NORTHERN CAPE . . . . . 03<br>FREE STATE . . . . . 04<br>KWAZULU-NATAL . . . . . 05<br>NORTH WEST . . . . . 06<br>GAUTENG . . . . . 07<br>MPUMALANGA . . . . . 08<br>LIMPOPO 09	
103	Do you live in a city, town, or rural area, a farm, a tribal area, or an informal settlement?	CITY . . . . . 1<br>TOWN . . . . . 2<br>RURAL AREA . . . . . 3<br>FARM . . . . . 4<br>TRIBAL AREA . . . . . 5<br>INFORMAL SETTLEMENT 6	
104	How old are you?<br>RECORD AGE IN COMPLETED YEARS.	AGE . . . . . [ ][ ]	
105	Are you male or female?	MALE . . . . . 1<br>FEMALE 2	
106	What is your current marital status?	CURRENTLY MARRIED . . . . . 1<br>LIVING WITH A MAN/WOMAN . . . . . 2<br>WIDOWED . . . . . 3<br>DIVORCED . . . . . 4<br>SEPARATED . . . . . 5<br>NEVER MARRIED OR LIVED WITH A MAN/WOMAN 6	
107	How many living children do you have?<br>INCLUDE ONLY CHILDREN WHO ARE YOUR BIOLOGICAL CHILDREN.	LIVING CHILDREN . . . . . [ ][ ]	
108	Have you ever had a child who died?	YES . . . . . 1<br>NO 2	

NO.	QUESTIONS AND FILTERS	CODING CATEGORIES	SKIP
109	What is the highest level of school you attended: primary, secondary, or higher than secondary?	PRIMARY . . . . 1<br>SECONDARY . . . . 2<br>HIGHER THAN SECONDARY 3	
110	What is the highest grade or form you completed at that level?	**PRIMARY SCHOOL**<br>LESS THAN 1 YEAR COMPLETED . . . . 00<br>GRADE 1/SUB A/CLASS 1 . . . . 11<br>GRADE 2/SUB B/CLASS 2 . . . . 12<br>GRADE 3/STANDARD 1/<br>AET 1 (KHA RI GUDE, SANLI) . . . . 13<br>GRADE 4/STANDARD 2 . . . . 14<br>GRADE 5/STANDARD 3/AET 2 . . . . 15<br>GRADE 6 /STANDARD 4 . . . . 16<br>GRADE 7/STANDARD 5/AET 3 . . . . 17<br><br>**SECONDARY SCHOOL**<br>LESS THAN 1 YEAR COMPLETED . . . . 20<br>GRADE 8/STANDARD 6/FORM 1/NTC 1/<br>N1/NC (V) LEVEL 2 . . . . 21<br>GRADE 9/STANDARD 7/FORM 2/AET 4/NTC 2/<br>N2/NC (V) LEVEL 3 . . . . 22<br>GRADE 10/STANDARD 8/FORM 3/NTC 3/<br>N3/NC (V) LEVEL 4 . . . . 23<br>GRADE 11/STANDARD 9/FORM 4 . . . . 24<br>CERTIFICATE OR DIPLOMA WITH LESS THAN<br>GRADE 12/STANDARD 10 COMPLETED . . 25<br>GRADE 12/STANDARD 10/FORM 5/MATRIC . . 26<br>N4/NTC4 . . . . 27<br>N5/NTC5 . . . . 28<br>N6/NTC6 . . . . 29<br><br>**HIGHER EDUCATION**<br>FURTHER STUDIES INCOMPLETE OR ONGOII 30<br>CERTIFICATE OR DIPLOMA WITH GRADE 12/<br>STANDARD 10 COMPLETED . . . . 31<br>HIGHER DIPLOMA (TECHNIKON/<br>U. OF TECHNOLOGY) . . . . 32<br>POST HIGHER DIPLOMA (TECHNIKON/<br>U. TECHNOLOGY MASTERS, DOCTORAL) 33<br>BACHELORS DEGREE/BACHELORS DEGREE<br>AND POST GRADUATE DIPLOMA . . . . . 34<br>HONOURS DEGREE . . . . 35<br>HIGHER DEGREE (MASTERS, DOCTORATE) 36	
112	Which population group do you consider yourself: black, white, coloured, Indian or something else?	BLACK/AFRICAN . . . . 1<br>WHITE . . . . 2<br>COLOURED . . . . 3<br>INDIAN/ASIAN . . . . 4<br><br>OTHER ______________ 6<br>(SPECIFY)	
113	What languages can you speak?<br><br>RECORD ALL LANGUAGES YOU CAN SPEAK.	ENGLISH . . . . A<br>AFRIKAANS . . . . B<br>ISIXHOSA . . . . C<br>ISIZULU . . . . D<br>SESOTHO . . . . E<br>SETSWANA . . . . F<br>SEPEDI . . . . G<br>SISWATI . . . . H<br>TSHIVENDA . . . . I<br>XITSONGA . . . . J<br>ISINDEBELE . . . . K<br><br>OTHER ______________ X<br>(SPECIFY)	

NO.	QUESTIONS AND FILTERS	CODING CATEGORIES	SKIP
114	What is your mother tongue/native language (language spoken at home growing up)?	ENGLISH . . . . . 01<br>AFRIKAANS . . . . . 02<br>ISIXHOSA . . . . . 03<br>ISIZULU . . . . . 04<br>SESOTHO . . . . . 05<br>SETSWANA . . . . . 06<br>SEPEDI . . . . . 07<br>SISWATI . . . . . 08<br>TSHIVENDA . . . . . 09<br>XITSONGA . . . . . 10<br>ISINDEBELE . . . . . 11<br><br>OTHER ______ 96<br>(SPECIFY)	
115	Have you ever worked on a SADHS survey prior to this one?	YES . . . . . 1<br>NO 2	
116	Have you ever worked on any other survey prior to this one (not a SADHS)?	YES . . . . . 1<br>NO 2	
117	Were you already working for STATS SA or SA MRC at the time you were employed to work on this SADHS?	YES, STATS SA . . . . . 1<br>YES, SA MRC . . . . . 2<br><br>NO 3	 <br><br>→ 119
118	Are you a permanent or temporary employee of STATS SA or SA MRC?	PERMANENT . . . . . 1<br>TEMPORARY 2	
119	If you have comments, please write them here.		

ADDITIONAL DHS PROGRAM RESOURCES

**The DHS Program Website** – Download free DHS reports, standard documentation, key indicator data, and training tools, and view announcements.	DHSprogram.com
**STATcompiler** – Build custom tables, graphs, and maps with data from 90 countries and thousands of indicators.	Statcompiler.com
**DHS Program Mobile App** – Access key DHS indicators for 90 countries on your mobile device (Apple, Android, or Windows).	Search DHS Program in your iTunes or Google Play store
**DHS Program User Forum** – Post questions about DHS data, and search our archive of FAQs.	userforum.DHSprogram.com
**Tutorial Videos** – Watch interviews with experts and learn DHS basics, such as sampling and weighting, downloading datasets, and how to read DHS tables.	www.youtube.com/DHSProgram
**Datasets** – Download DHS datasets for analysis.	DHSprogram.com/Data
**Spatial Data Repository** – Download geographically-linked health and demographic data for mapping in a geographic information system (GIS).	spatialdata.DHSprogram.com

Social Media – Follow The DHS Program and join the conversation. Stay up to date through:

**Facebook** www.facebook.com/DHSprogram	**Twitter** www.twitter.com/DHSprogram
**Pinterest** www.pinterest.com/DHSprogram	**LinkedIn** www.linkedin.com/company/dhs-program
**YouTube** www.youtube.com/DHSprogram	**Blog** Blog.DHSprogram.com
